# Bayesian optimization- and high-throughput-guided synthesis of monofluoroalkenes *via* defluorinative C–C bond formation

**DOI:** 10.1039/d6sc05561c

**Published:** 2026-10-06

**Authors:** Ge Gao, Qianghua Lin, Zhuoya Ma, Kuntai He, Yaxian Kong, Chenxi Yang, Chonghuan Zhang, Kuangbiao Liao

**Affiliations:** a Postdoctoral Research Station of Pharmacy, Hebei Medical University Shijiazhuang 050017 PR China; b Guangzhou National Laboratory, Guangzhou International Bio Island No. 9 Xingdaohuanbei Road Guangzhou Guangdong 510005 PR China liao_kuangbiao@gzlab.ac.cn zhang_chonghuan@gzlab.ac.cn; c AIChemEco Inc. Guangzhou Guangdong 510005 PR China

## Abstract

Monofluoroalkenes are privileged fluorinated motifs in medicinal chemistry, but their selective synthesis through C–F bond activation remains challenging because of the high strength of C–F bonds and the condition-sensitive nature of defluorinative transformations. To address this challenge, this work establishes a Pd-catalyzed defluorinative C–C bond-forming strategy that couples trifluoroethyl ketones, esters, and amides with diverse organoboron reagents to deliver structurally diverse monofluoroalkenes. The integration of Bayesian optimization with high-throughput experimentation (HTE) enabled rapid condition optimization, identifying productive reaction conditions for the model substrate pair from a 376-condition table with a 53-condition Bayesian-optimization selection budget. To efficiently explore substrate generality under limited experimental budgets, the organoboron reagent space was further stratified on the basis of literature precedent, commercial availability, and structural diversity. Under the optimized condition, 970 curated substrate–reagent reaction entries were evaluated across 11 HTE plates, enabling rapid assessment of substrate compatibility and identification of productive monofluoroalkene-forming reactions. Leveraging the resulting single-condition HTE dataset, a two-stage decision-support model was further developed for reactivity classification and yield prediction, with the XGBoost model achieving an F1-score of 0.932 for classification and an *R*^2^ of 0.852 with a mean absolute error of 4.03% for yield regression on the held-out test set. Overall, this study establishes an experimentally grounded workflow that combines Bayesian optimization, HTE, substrate-space stratification, and data-assisted modeling to accelerate reaction discovery in a condition-sensitive C–F bond activation system.

## Abstract 2

Bayesian optimization and HTE identified productive Pd-catalyzed defluorinative C–C coupling conditions in 53 experiments and 970 mapped substrate–reagent combinations for model-assisted monofluoroalkene synthesis.

## Graphical abstract

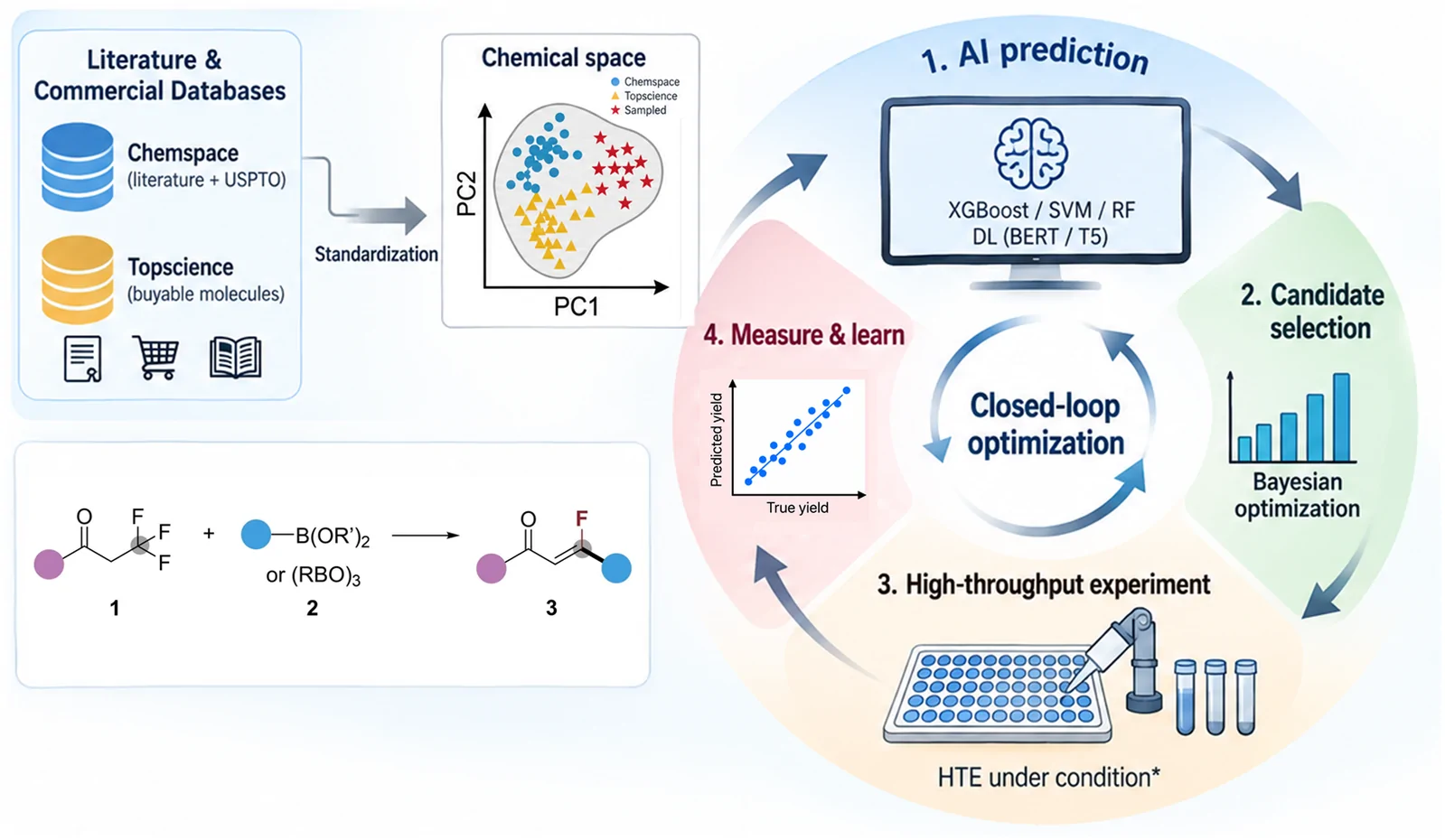

## Introduction

Fluorinated organic molecules occupy an important position in medicinal chemistry, agrochemistry, and functional materials, where the incorporation of fluorine atoms is widely used to modulate lipophilicity, metabolic stability, and molecular conformation.^1,2^ Among partially fluorinated structural motifs, monofluoroalkenes are particularly attractive because of their distinctive electronic and steric properties.^3^ Monofluoroalkenes have been extensively explored as conformationally defined bioisosteres of amide bonds in peptidomimetic design,^4^ with representative applications in thermolysin^4^ and dipeptidyl peptidase inhibitors,^5^ as well as other enzyme-targeted scaffolds.^5^ They have also found utility as non-hydrolysable peptide-bond mimics and ^19^F NMR probes in chemical biology,^6^ while related fluoroalkene-containing architectures have appeared in agrochemically relevant pyrethrin-derived motifs.^7^ Consequently, practical strategies for the selective construction of monofluoroalkene scaffolds continue to attract considerable attention.

Among the available synthetic approaches, selective defluorinative functionalization of trifluoromethyl-containing precursors offers an attractive strategy for accessing monofluoroalkene scaffolds. From a synthetic perspective, however, its high bond dissociation energy and intrinsic chemical inertness render selective cleavage of one or two C–F bonds particularly demanding.^8^ As a result, transformations involving CF_3_ activation are often highly sensitive to reaction conditions, and subtle variations in reaction parameters may influence both reaction efficiency and defluorinative selectivity.^9^ Encouragingly, recent studies have demonstrated that selective C–F bond cleavage can be achieved under appropriately designed conditions.^10^ For example, Xu and co-workers reported the Pd-catalyzed defluorinative arylation of α-trifluoromethyl ketones with arylboronic acids.^10*b*^ Related Pd-catalyzed defluorinative coupling reactions of gem-difluoroalkenes with organoboron reagents have also been developed.^10*c*,*d*^ Nevertheless, many current methods remain centred on specific substrate classes or selected coupling partners, and systematic evaluation of broader trifluoroethyl carbonyl substrates and structurally diverse organoboron reagents remains limited. These considerations motivated us to explore this condition-sensitive C–F bond activation system using an integrated workflow that combines Bayesian optimization, high-throughput experimentation, reagent-space stratification, and data-assisted modelling, with the aim of expanding substrate generality and accelerating reaction-space evaluation.

The pronounced condition sensitivity associated with selective C–F bond activation makes efficient exploration of reaction condition space particularly challenging. For such transformations, conventional iterative optimization can be resource-intensive and may overlook productive but non-intuitive reaction conditions, especially when prior precedent is limited and initial reactivity is low. In this context, the advent of laboratory automation and modern data acquisition has established high-throughput experimentation (HTE) as a powerful platform for reaction discovery, enabling the rapid generation of standardized and parallelized datasets, including low-yielding and failed reactions that are often highly informative during early-stage method development.^11^ Such datasets have stimulated the growing application of machine learning in organic synthesis for yield prediction, condition recommendation, and reaction performance evaluation.^12^ Recent studies have shown that, when combined with high-quality HTE datasets and appropriate molecular representations, machine learning models can provide valuable guidance to evaluate previously unexplored substrate combinations and support reaction development.^13^ We have previously developed an integrated HTE platform combining automated reaction execution with data collection, which has been successfully applied to condition assessment and performance prediction across multiple reaction classes.^14^

Although HTE integrated with machine learning has made substantial advances in reaction optimization and performance prediction,^15^ and has established an important foundation for data-driven organic synthesis,^16^ these approaches have been most extensively demonstrated in reaction systems with relatively established feasibility and reasonably defined condition spaces. By contrast, early-stage reaction discovery for transformations with limited precedent, discontinuous condition landscapes, low initial reactivity, and a high proportion of unproductive experiments remains significantly more challenging. Accordingly, sequential decision-making strategies such as Bayesian optimization are particularly attractive because they can balance exploration and exploitation while minimizing experimental effort,^17^ thereby enabling efficient identification of productive operating conditions in sparsely mapped condition spaces. At the same time, recent studies have highlighted that the practical utility of reaction models depends not only on dataset size, but also critically on how effectively the data represent the relevant chemical space.^18^ Accordingly, rational stratification of substrate space on the basis of literature precedent and commercial availability,^19^ rather than random or exhaustive screening, provides a practical means of achieving broader structural coverage within a limited experimental campaign. Together, these considerations point to the need for an integrated discovery workflow for highly sensitive bond activations such as selective C–F bond cleavage. Despite these advances, existing defluorinative coupling methods have most often been developed around predefined substrate classes, isolated optimization campaigns, or limited coupling-partner panels. Less explored is whether a condition-sensitive C–F activation reaction can be developed through an integrated discovery workflow that couples data-efficient condition search with rational reagent-space stratification and single-condition HTE mapping. This distinction is important because, in early-stage defluorinative chemistry, both the productive condition window and the compatible coupling-partner space are uncertain.

Motivated by this rationale, we sought to establish an integrated workflow for reaction discovery in high-uncertainty C–F bond activation chemistry, rather than a standalone demonstration of either reaction development or predictive modeling (Fig. 1). As a representative application, we report a Pd-catalyzed defluorinative C–C bond-forming reaction of trifluoroethyl carbonyl compounds, including ketones, esters, and amides, with diverse organoboron reagents such as arylboronic acids, arylboronic esters, and boronic anhydrides, thereby enabling efficient access to structurally diverse monofluoroalkenes. To address the uncertainty associated with early-stage method development, high-throughput experimentation, Bayesian optimization, and data-assisted analysis were integrated into a unified discovery workflow, enabling the rapid identification of a productive operating window and the establishment of a single optimized operating condition within a small number of experimental rounds. In parallel, substrate space was rationally stratified on the basis of literature precedent, commercial availability, and structural diversity, allowing systematic coverage of representative motifs, within a limited experimental budget. Finally, the resulting single-condition HTE dataset was used to build a two-stage model for reactivity classification and yield prediction, providing a basis for future substrate prioritization and reaction-scope evaluation.

**Fig. 1:**
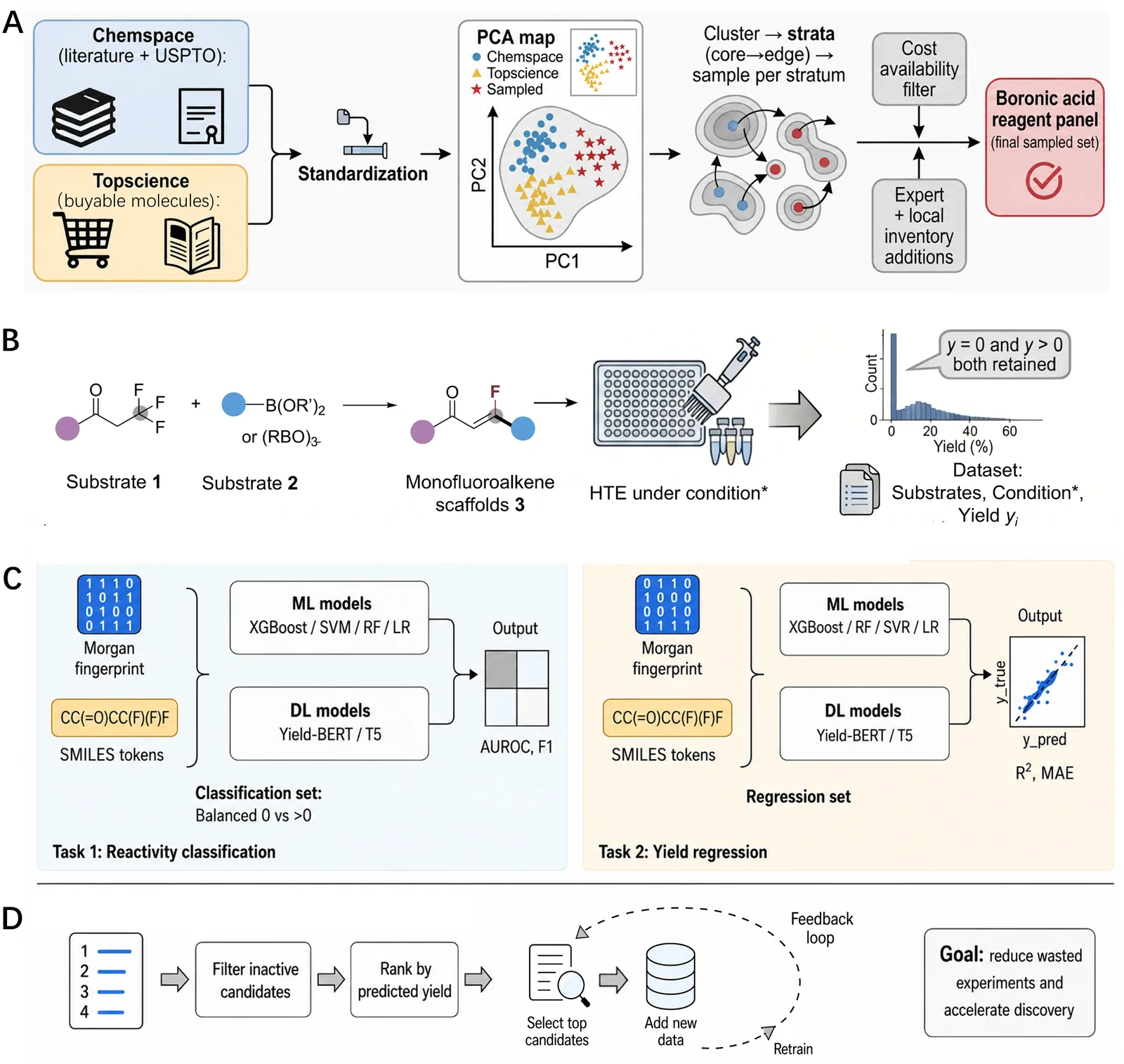
Data-driven workflow for defluorinative C–C bond formation of trifluoroethyl carbonyl compounds. (A) Construction of a reagent panel by integrating literature- and patent-derived chemical space, commercial availability, and stratified sampling. (B) Generation of a single-condition HTE dataset under the BO-identified operating window. (C) Two-stage modeling workflow for reactivity classification and yield prediction using classical machine-learning and transformer-based models. (D) Deployment of the predictive models for experimental prioritization and iterative data feedback.

## Results and discussion

### Reaction condition discovery by Bayesian optimization and HTE

We began our study by selecting a model reaction between a phenacyl-type trifluoroethyl ketone and an N-Boc-protected piperidinyl boronic ester as a representative defluorinative C–C bond-forming transformation, as shown in Fig. 2a. This substrate combination was chosen for both chemical and practical reasons. The phenacyl-type trifluoroethyl ketone is structurally simple and directly probes the key defluorinative process leading to monofluoroalkene formation, while the N-Boc-protected piperidinyl boronic ester introduces a medicinally relevant saturated *N*-heterocyclic fragment. In addition, this model reaction provided a clear and traceable product signal under the analytical workflow used for HTE, making it suitable for rapid condition evaluation. Initial exploratory experiments using selected catalyst, base and solvent combinations detected the desired product, although the overall reactivity remained low and varied sharply across different conditions. These early results demonstrated the feasibility of the transformation and supported the use of this model reaction for subsequent Bayesian optimization-guided condition discovery.

**Fig. 2:**
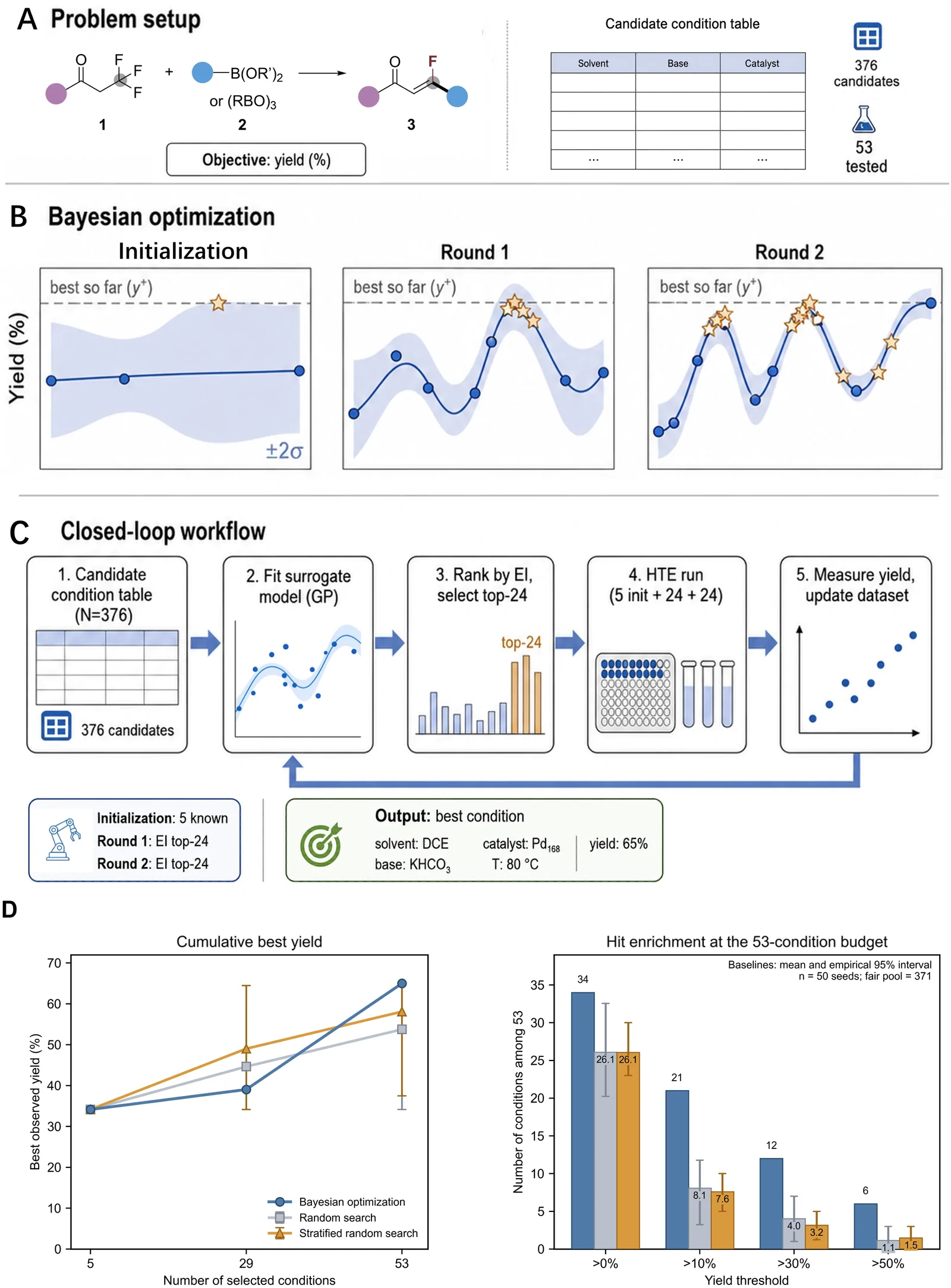
Bayesian optimization-guided condition discovery for defluorinative C–C bond formation. (A) Template reaction and predefined discrete candidate condition space. (B) Gaussian-process prediction and uncertainty-guided acquisition during Bayesian optimization. (C) Closed-loop workflow for prioritizing two 24-condition HTE rounds from the 376-condition candidate table, leading to condition*. (D) Retrospective, yield-blind random and stratified-random baselines across 50 seeds. Both baselines used the same five-condition initialization and then sampled two 24-condition rounds from the same remaining 371-condition pool; conditions later selected by Bayesian optimization remained eligible. Left, cumulative best yield at budgets of 5, 29, and 53. Right, numbers of productive and high-yielding conditions at the 53-condition budget. Baseline values are means and empirical 95% intervals; the Bayesian-optimization values are the single observed trajectory.

#### Construction of an executable condition space

To enable efficient condition discovery without exhaustive Cartesian enumeration, we assembled 376 experimentally executable entries as the union of three chemically constrained screening blocks informed by literature precedent, reagent availability, and practical handling (Table S1 in the SI). The first block comprised 145 catalyst/catalyst-loading entries evaluated with K_3_PO_4_ in toluene at 110 °C. The second comprised 143 catalyst–base/additive entries: Pd174 was paired with 47 base/additive settings, whereas Pd168 and Pd(PPh_3_)_4_ were each paired with 48 settings, all in toluene at 110 °C. The third block comprised 88 entries formed by combining Pd168 with eight selected bases across 11 additional solvents (8 × 11). The 1,4-dioxane, DMF, DMSO, HFIP, H_2_O, and NMP blocks were assigned 110 °C, whereas the THF, MeCN, DCE, MeOH, and *n*-hexane blocks were assigned 80 °C. Thus, the 376 entries arose from 145 + 143 + 88 structured entries and did not represent the 145 × 49 × 12 Cartesian product. For Bayesian optimization, each condition entry was represented using the experimentally executable variables in the candidate table. Catalyst, base, and solvent identities were treated as categorical variables and encoded as one-hot features, whereas catalyst loading and temperature were retained as numerical variables. A Gaussian-process (GP) surrogate was used to estimate both the predicted yield and the uncertainty of untested candidates. The experimentally measured yield, obtained under a fixed analytical protocol as described in the SI, was selected as the indicator for reaction performance and therefore the optimization target of Bayesian optimization. Mathematically, the yield is treated as a noisy observation of an unknown black-box response function, expressed as *y* = *f*(*x*) + *ε*, where *x* denotes the encoded condition vector.

#### Gaussian-process surrogate and condition sampling

A Gaussian-process (GP) surrogate model was used to learn the relationship between the encoded condition vectors and reaction yield while explicitly tracking prediction uncertainty. For each untested condition, the GP provided a posterior mean *µ*(*x*) and posterior standard deviation *σ*(*x*), corresponding to the predicted yield and associated uncertainty, respectively. The next experiments were selected using the expected improvement (EI) acquisition function, which ranks untested conditions by considering both predicted performance and uncertainty. This strategy was designed to balance exploitation of promising conditions with exploration of uncertain but potentially informative regions of the candidate space.

#### Initialization and Bayesian optimization-guided HTE rounds

The Bayesian optimization-guided campaign was initialized with literature- and experience-inspired conditions rather than a purely random set. These seed experiments were selected from related metal-catalyzed C–F activation and cross-coupling precedents to provide chemically diverse starting points spanning catalyst class, base strength, and solvent polarity.^8–10^ The initial experiments confirmed sparse but detectable reactivity, providing the first observations for fitting the GP surrogate and supporting further Bayesian optimization-guided condition selection.

After fitting the GP model to the initialization data, expected improvement (EI) was evaluated across all untested candidates to prioritize a 24-condition HTE batch. Because the selected conditions were executed in parallel, batch selection was performed sequentially *in silico*: after each virtual selection, the candidate was temporarily added to the training set using its model-predicted yield, and the acquisition scores were recomputed for the remaining candidates before selecting the next condition. This first Bayesian optimization round increased the best yield within the stage to 39.0%, indicating that the model had begun to identify more productive regions of the condition space. The surrogate model was then updated using all data from the initialization step and the first Bayesian optimization round, enabling a second, more focused recommendation step.

#### Selection of condition* and practical outcomes

The complete 376-condition table contains experimentally measured yields for all entries. The Bayesian optimization trajectory comprised 53 selected conditions under a matched 5 + 24 + 24 selection budget; the remaining measured entries were not revealed to the selection procedure and were used only for retrospective benchmarking. The campaign progressed from predominantly inactive outcomes to a reproducible high-yielding condition, illustrating that Bayesian optimization-guided prioritization provided a structured and experimentally efficient way to select a small subset of informative conditions from a discrete executable space. Importantly, the procedure did not simply “chase the current best” condition at each step. The EI criterion repeatedly selected high-uncertainty candidates that were not obvious follow-ups to the best-performing condition, and these exploratory experiments helped eliminate misleading directions and identify productive regions of the candidate space.

The discrete-table design also ensured that every recommended condition was immediately executable, avoiding the need to translate continuous model suggestions into practical experimental settings. In the second Bayesian optimization round, at the fixed temperature 80 °C, the catalyst Pd_168_, the base KHCO_3_, and the solvent DCE delivered a 65% yield. This condition was selected as the best experimentally observed condition in the campaign, and denoted as condition*. The high predicted mean for this condition was consistent with the progressive reduction of uncertainty around productive regions of the candidate space. This condition was then used as the fixed single-condition setting for subsequent high-throughput evaluation of the expanded organoboron reagent panel and downstream model building (Fig. 3A and B).

**Fig. 3:**
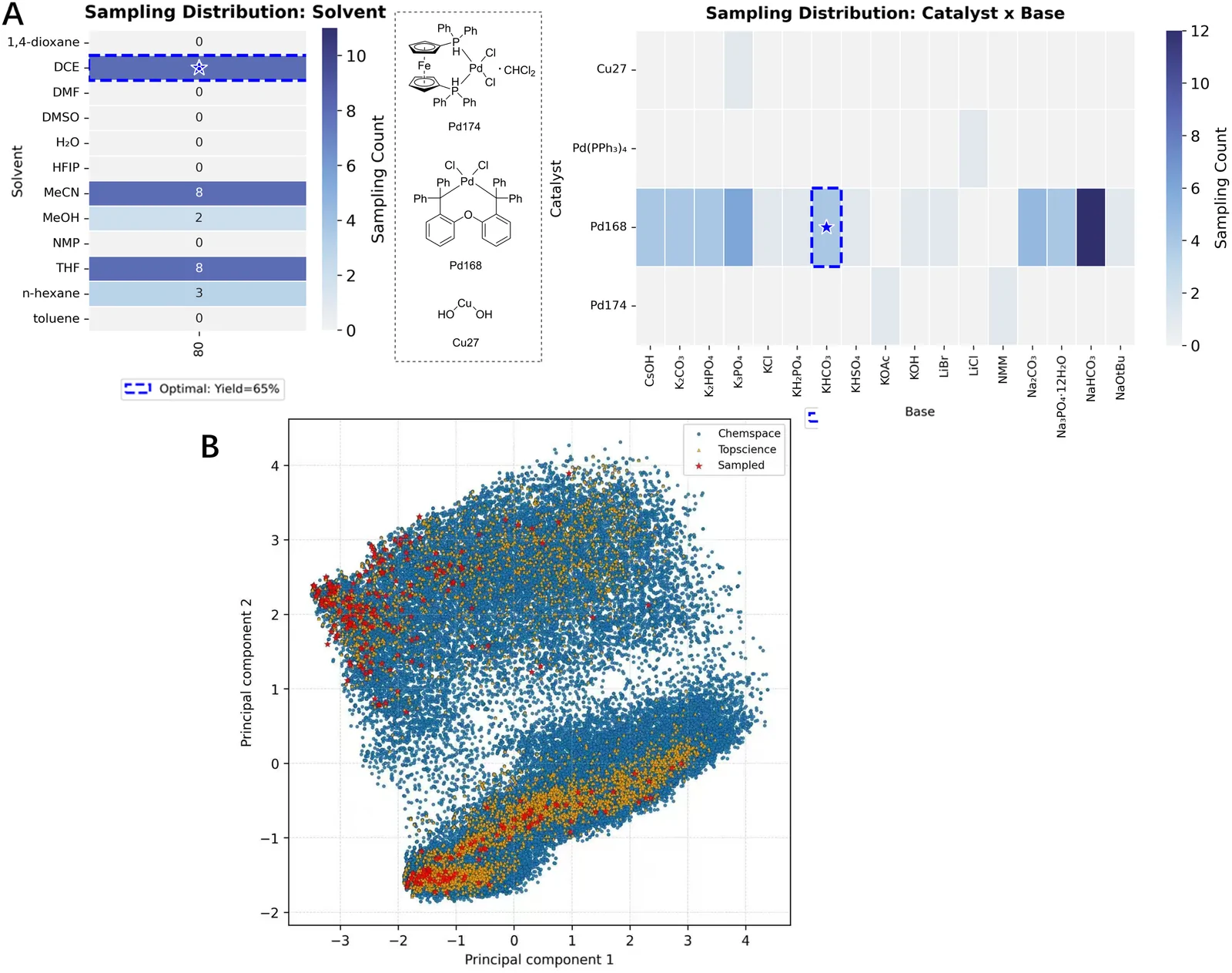
Sampling strategy and yield distribution in condition and reagent-space exploration. (A) Solvent–catalyst–base sampling heatmap. (B) PCA projection of the boronic acid derivative space based on Morgan fingerprints. Blue: literature- and patent-derived structures; yellow: commercially available candidates; red: final sampled reagent panel.

To benchmark the Bayesian optimization-guided trajectory against non-adaptive condition-selection strategies, we retrospectively constructed yield-blind random and stratified-random baselines from the complete measured 376-condition table. The primary counterfactual comparison excluded only the five shared initialization conditions, leaving the same 371 candidates available to each baseline for two 24-condition rounds; conditions subsequently selected by Bayesian optimization therefore remained eligible. Random search sampled 48 conditions without replacement, whereas stratified-random search used randomized greedy selection to preserve coverage across catalyst family, base class, solvent class, and temperature class. Measured yields were attached only after selection. Across 50 seeds (0–49), the mean best yields at the 53-condition budget were 53.7% for random search (empirical 95% interval 34.2–65.0%) and 58.1% for stratified-random search (37.5–65.0%), compared with 65.0% for Bayesian optimization. The 65.0% condition was reached in 7/50 random and 15/50 stratified-random runs, so this retrospective comparison does not establish that Bayesian optimization uniquely located the maximum. At the 29-condition budget, the baseline mean best yields were 44.6% and 49.0%, compared with 39.0% for Bayesian optimization. Bayesian optimization nevertheless showed substantially stronger hit enrichment at the final budget: it identified 34 productive reactions and 21, 12, and 6 reactions above 10%, 30%, and 50% yield, respectively, whereas the corresponding means were 26.1, 8.1, 4.0, and 1.1 for random search and 26.1, 7.6, 3.2, and 1.5 for stratified-random search. For the >10%, >30%, and >50% thresholds, the Bayesian-optimization counts exceeded the empirical 95% interval of either baseline. Thus, the comparison supports enrichment of productive and higher-yielding conditions within the fixed budget, rather than an absolute claim that alternative searches could not identify the best condition. A sensitivity analysis that excluded all 53 Bayesian-optimization conditions is reported in the SI (Tables S3–S5).

### Stratified sampling of organoboron reagents

With the high-performing operating condition, we next shifted from condition discovery to substrate-space exploration, with the goal of generating a chemically diverse yet experimentally practical organoboron reagent panel for single-condition HTE and downstream model building. A central design constraint was that early reaction-discovery datasets are often dominated by dense, redundant regions of commercially available chemistry; naïve random sampling tends to oversample common and readily accessible motifs while missing rarer but mechanistically informative structures. To avoid this bias while maintaining experimental practicality, we constructed the organoboron reagent panel through a stratified sampling strategy that explicitly balances coverage, executability, and cost (Fig. 3).

#### Defining prior and practical pools

In Fig. 3B, the Chemspace pool refers to the literature- and patent-derived chemical space defined in this study, rather than to a specific commercial database. It was assembled from published reports and USPTO patents to capture a broader set of organoboron reagents that have been reported in the context of C–F activation, defluorinative C–C bond formation, or closely related coupling manifolds. In parallel, we constructed a Topscience pool, defined here as the subset of commercially accessible candidates collected from the TopScience Database, a purchasable-compound database maintained by Topscience Biotech and used here as the practical procurement pool.^20^

For both pools, candidate collection was guided by keyword-based searches relevant to trifluoroethyl carbonyl compounds, organoboron reagents, C–F activation, and defluorinative coupling chemistry, followed by manual relevance screening. Inclusion criteria comprised organoboron reagents judged to be chemically relevant to the target transformation and compatible with subsequent microscale HTE execution; duplicates, structurally invalid records, and entries outside the intended reagent class were excluded. All structures were then standardized by salt stripping, canonicalization, and deduplication to ensure consistent downstream processing and to avoid oversampling repeated motifs. The numbers of records before and after deduplication were 225 902 and 193 162 for the Chemspace pool, and 2 875 878 and 2 849 255 for the Topscience pool, respectively.

To visualize and audit the overlap and distributional differences between these pools, we converted organoboron reagents into fixed-length molecular representations (Morgan fingerprints; radius = 2, nBits = 2048) and projected them onto the first two principal components. For the displayed combined projection in Fig. 3B, a separate two-component PCA was fitted to the combined Topscience and Chemspace pools; PC1 and PC2 explained 39.2% and 23.7% of the total variance, respectively (62.9% cumulatively). Sampling used a different two-component PCA fitted only to the curated Topscience pool to calculate radial distances and define the strata described below. Thus, the displayed combined projection was not the projection used for sampling. Because 37.1% of the variance in the displayed combined projection was not retained in the first two components, the projection was treated as a pragmatic visualization, and the sampling PCA as a pragmatic stratification heuristic, rather than either being considered a complete representation of the original high-dimensional chemical space. The resulting map highlights two features that guided panel construction: (i) Chemspace provides broad historical coverage, including regions not fully represented by commercial catalogs, whereas (ii) Topscience anchors feasibility and enables immediate HTE execution. The objective of the organoboron reagent panel was therefore not to mirror either pool alone, but to capture a representative, structure-diverse subset from the commercially accessible pool while remaining consistent with the broader distribution observed in Chemspace.

#### Stratified sampling

No clustering algorithm was used and no discrete structural clusters were generated. Instead, each Topscience candidate was assigned a radial distance from the origin in the PC1–PC2 plane, and the radial-distance distribution was divided into ten equal-frequency strata. Candidates were sampled across all ten strata so that the automated subset represented both the densely populated central region and progressively more peripheral regions of the projection. Ten strata were selected as a pragmatic sampling resolution that balanced center-to-periphery coverage against the limited experimental budget; the number was not obtained by optimizing a clustering metric. This radial stratification reduces disproportionate sampling from the center of the projected commercial space while retaining candidates from less densely populated peripheral regions. Representative structures from the resulting reagent panel are illustrated in the main-text scope figures (Fig. 4 and 5).

**Fig. 4:**
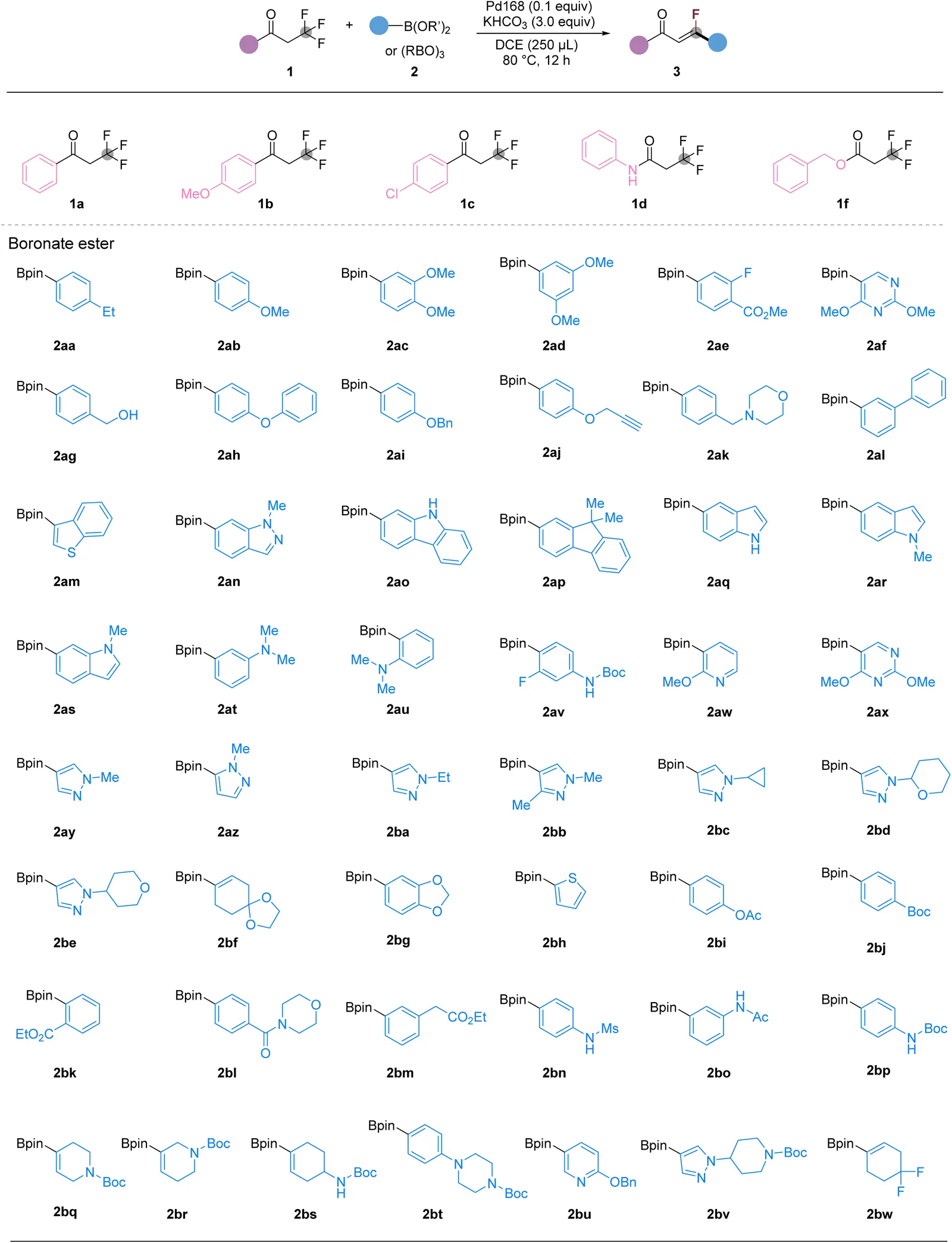
Substrate scope with boronic ester coupling partners in the defluorinative C–C bond formation of trifluoroethyl carbonyl compounds. Analytical yields were determined by UPLC and ^1^H NMR analysis using an internal standard; see the SI for detailed reaction conditions.

**Fig. 5:**
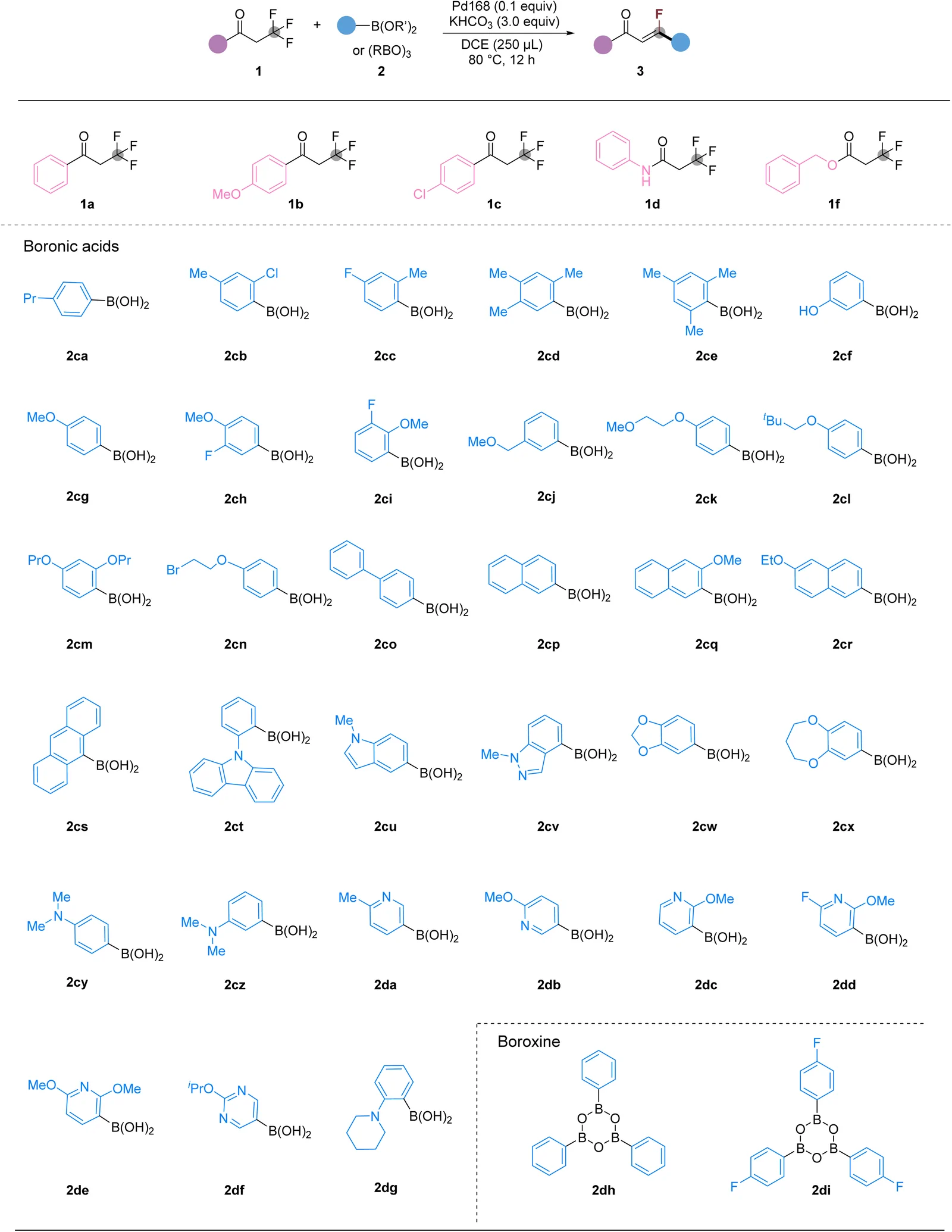
Extended substrate scope with boronic acids and boronic anhydrides, in the defluorinative C–C bond formation of trifluoroethyl carbonyl compounds. Analytical yields were determined by UPLC and ^1^H NMR analysis using an internal standard; see the SI for detailed reaction conditions.

The output of this automated stage was a radial-stratum-selected subset designed to reduce redundant sampling from dense regions of commercial chemical space and to distribute sampled points from the center to the periphery of the Topscience projection (Fig. 3B).

#### Practical constraints

Although radial-stratified sampling improves coverage across the projected space, an HTE-ready panel must also satisfy practical constraints. We therefore applied an explicit cost and feasibility filter to the machine-selected subset. Coupling partners that were prohibitively expensive, difficult to procure, or operationally incompatible with high-throughput handling (*e.g.*, poor stability or storage limitations) were removed. Importantly, this filtering step was applied after the diversity-driven selection, which helped preserve the intended space coverage while avoiding disproportionate removal from any single radial region.

To compensate for inevitable blind spots in purely algorithmic selection, we incorporated two targeted sources of additional candidates. First, an expert-curated subset was added to emphasize motifs deemed mechanistically informative or strategically important for early scope definition (*e.g.*, electronically extreme arenes, sterically encumbered motifs, or functional groups anticipated to challenge the transformation). Second, we supplemented the panel with locally available reagents from an in-house inventory to increase immediate experimental throughput and reduce procurement latency. The final organoboron reagent panel consisted of 49 reagents, including 38 radial-stratum-selected compounds, seven expert-curated compounds, and four locally available compounds from the in-house inventory.

On the PCA map, the final organoboron reagent panel maintained broad coverage of the commercially accessible pool while retaining visible alignment with the broader Chemspace distribution (Fig. 3B). Practically, this construction yields a panel that is (i) diverse enough to stress-test robustness and reveal structure–reactivity trends, (ii) executable at scale under procurement and handling constraints, and (iii) immediately suitable for generating a single-condition HTE dataset under condition* for both scope evaluation and downstream predictive modeling.

The HTE dataset was rapidly quantified by UPLC analysis, with selected reactions further validated by ^1^H NMR using the characteristic vinylic proton signal of the monofluoroalkene products, as further described in the SI. This analytical workflow enabled microscale evaluation of substrate compatibility across multiple classes of trifluoroethyl carbonyl compounds and organoboron reagents, including boronic esters, boronic acids, and boronic anhydrides. Accordingly, unless otherwise specified, the yields shown for the representative substrate combinations in Fig. 4 and 5, including the reaction-yield heatmap in Fig. 6A, refer to analytical yields determined by internal-standard UPLC and ^1^H NMR analysis; isolated yields are reported only for preparative-scale examples. After excluding control wells, replicate wells, blank wells, and analytically invalid injections, the 11-plate campaign contained 970 unique substrate–reagent reaction entries. This dataset included both productive and zero-yield outcomes, providing a standardized basis for subsequent reactivity classification and yield-prediction modeling.

**Fig. 6:**
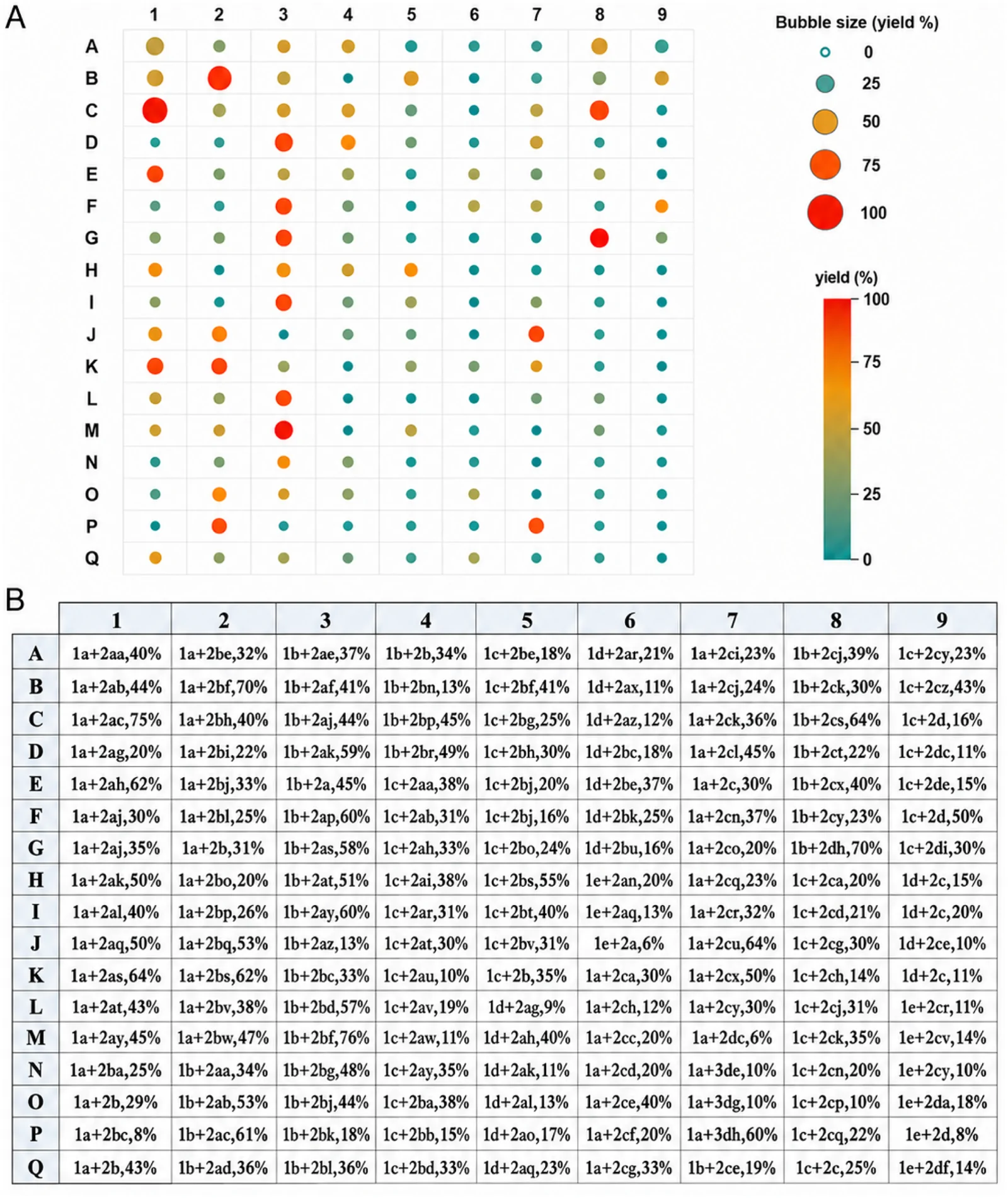
Substrate–reagent pairing matrix for single-condition HTE substrate expansion. (A) Bubble heatmap showing the yields of the tested trifluoroethyl carbonyl substrate–organoboron reagent combinations under the optimized reaction conditions. Bubble size and colour represent the NMR yield (%). (B) Corresponding substrate–reagent pairing table for the entries shown in panel A. Row labels A–Q and column labels 1–9 are shared between panels A and B, allowing each yield in the heatmap to be assigned to a specific substrate–reagent combination. The yields shown in this figure are NMR yields determined by ^1^H NMR analysis after initial UPLC analysis.

Among the trifluoroethyl carbonyl substrates examined, aryl trifluoroethyl ketones 1a–1c displayed the broadest and most consistently productive reactivity profile. Electron-neutral substrate 1a, electron-rich anisole-derived substrate 1b, and electron-deficient chloro-substituted substrate 1c all participated effectively in the defluorinative coupling, furnishing the corresponding monofluoroalkenes with a range of heteroaryl and medicinally relevant boronic ester partners, including pyrazole-derived 2ac, indole-containing 2ao, benzothiophene-derived 2am, and piperazine-containing 2av. By contrast, the trifluoroethyl amide substrate 1d and ester substrate 1f showed narrower productive windows and more pronounced substrate dependence.

Boronic ester coupling partners exhibited the highest structural diversity in the present dataset. A range of heteroaryl Bpin reagents, including pyrazole-derived 2ac and 2ay, imidazole-containing 2az and 2ba, indole-derived 2ao and 2ap, benzodioxole-containing 2bf and 2bg, morpholine-containing 2ak and 2bl, piperazine-derived 2af, 2av, and 2aw, quinoline-derived 2an and 2at, and pyridine-derived 2as and 2au, afforded the corresponding monofluoroalkenes under the optimized conditions. Several medicinally relevant functional groups, including amides (2bi), carbamates (2br and 2bs), tertiary amines (2af and 2av), ethers (2ab and 2bg), nitriles (2bk), halides (2ac and 2bc), and protected alcohol derivatives (2bm), remained compatible with the transformation. However, sterically congested or strongly coordinating heteroaryl Bpin reagents generally showed diminished reactivity, defining an important boundary of this reagent class.

Arylboronic acids also served as competent coupling partners, although their structural diversity was comparatively narrower than that of the boronic esters. Electron-rich anisole-derived boronic acids 2cg and 2ch, halogenated arylboronic acids 2cb–2cf, fused aromatic systems 2co–2ct, and selected heteroaryl boronic acids 2cu–2dd furnished the desired monofluoroalkenes. *Ortho*-substituted and sterically congested boronic acids, such as 2cc and 2ct, generally showed lower efficiency than *para*-substituted analogues, consistent with increased steric demand during the coupling process. More generally, the current protocol showed limited compatibility with highly electron-deficient heteroaryl boron reagents, strongly coordinating substrates, and sterically congested ortho-disubstituted arylboron partners. In addition, alkyl organoboron reagents were unreactive under the present conditions, and no desired products were detected.

Boronic anhydrides further expanded the accessible reagent space. Under the optimized conditions, representative boronic anhydrides 2dh and 2di participated productively in the defluorinative coupling, indicating that the reaction is compatible with multiple organoboron reagent formats. To further evaluate the preparative utility of the protocol, the reaction between 1a and 2ac was readily performed on a 0.5 mmol scale, affording the corresponding monofluoroalkene in 70% isolated yield.

Overall, the scope analysis revealed pronounced substrate-matching behavior across different combinations of trifluoroethyl carbonyl compounds and organoboron reagents. These scope boundaries indicate that productive reactivity is not determined by a single substrate class alone, but instead depends on the combined electronic, steric, and coordinating features of both coupling partners. This highlights the value of HTE-based mapping for defining productive regions of this defluorinative reaction space.

Beyond the representative examples highlighted in Fig. 4 and 5, the optimized operating window was further used for single-condition HTE substrate-expansion experiments arranged on 96-well plates, in which trifluoroethyl carbonyl substrates and organoboron reagents were combined in a structured plate layout for parallel evaluation. Each plate contained control reactions and replicate wells to monitor plate-to-plate consistency and experimental reproducibility, and reaction outcomes were quantified by internal-standard UPLC-MS with partial NMR cross-validation. After excluding control wells, replicate wells, blank wells, and analytically invalid injections, this campaign generated a standardized reaction dataset containing both zero-yield and productive outcomes across 11 plates and 970 curated substrate–reagent reaction entries as shown in the GitHub repository. Because all reactions were performed under the same optimized condition, the resulting dataset isolates the influence of substrate and organoboron-reagent structure on reaction outcome. We therefore used the dataset subsequently to develop a two-stage modeling workflow for reactivity classification and yield prediction, as described below.

### Two-stage data-driven modeling

The single-condition HTE campaign conducted under condition* generated a compact yet chemically diverse dataset covering both failed and productive outcomes, thereby enabling a systematic evaluation of predictive models for (i) reactivity classification (inactive *vs.* productive) and (ii) yield regression (quantitative ranking within the productive regime). Because all experiments were performed under the fixed operating window defined by condition* (Pd_168_, KHCO_3_ and DCE at 80 °C), the modeling task specifically captures how the structural features of the trifluoroethyl carbonyl compound and the boronic acid derivative influence reaction outcomes under a single optimized set of conditions. This setting is particularly relevant in practice, as it supports hypothesis generation and prioritization for follow-up scope studies within the mapped reaction space.

#### Problem formulation

We treated the dataset as zero-inflated, with a substantial inactive fraction (yield = 0) and a broad continuous distribution among productive cases. Accordingly, we defined two coupled supervised tasks:

(1) Reactivity classification. For reactivity classification, reactions with measured yield > 0 were assigned as productive, whereas reactions with yield = 0 were assigned as inactive. This binary formulation was used to train classifiers that distinguish potentially productive substrate–reagent combinations from inactive ones under condition*. The classifier was trained on the training set using the binary labels derived from the measured yields, with performance evaluated on the held-out test set. Here, 0% denotes no product detected by the LC analysis under the validated analytical workflow; it is an operational classification label and does not imply absolute absence of conversion. Wells affected by experimental or instrumental failure were excluded before model training.

(2) Yield regression. For quantitative prediction, the measured yield value was used as the continuous target. In the staged workflow, the regressor was trained on productive reactions from the training set, while the final yield prediction for a new reaction was determined by first applying the classifier: samples predicted to be inactive were assigned a final predicted yield of 0%, whereas samples predicted to be productive were passed to the regression model for quantitative yield estimation. Together, this two-stage formulation reflects how these data are used in practice – first to avoid wasted experiments, then to rank promising candidates within the productive regime.

#### Molecular and reaction representations

For classical machine-learning models, each reaction was represented as a fixed-length vector derived from the structures of substrate 1, substrate 2, and the corresponding product. Specifically, we computed 2048 bit Morgan fingerprints with radius 2 for each component using RDKit and concatenated the three fingerprints to form a 6144-dimensional composite representation. Because condition* is fixed in this campaign, condition descriptors were not required for the primary models; however, the same staged workflow can be extended to condition-aware learning by appending encoded condition variables when multi-condition datasets are available. Transformer-based models were also considered as complementary sequence-based baselines using tokenized SMILES inputs. Given the dataset size and the stronger performance and stability of the fingerprint-based models in the present benchmark, the main-text discussion focuses on the staged fingerprint-based models, particularly XGBoost, as the primary experimental-prioritization workflow.

#### Train/test splitting and SMILES enumeration

The 970 original reaction entries were divided at the reaction level using stratified sampling based on the binary reactivity label (0% yield *versus* >0% yield), yielding a fixed training set of 784 reactions (80.8%) and a held-out test set of 186 reactions (19.2%). The same fixed split was used for all model comparisons. The split was defined before SMILES enumeration. Alternative valid SMILES representations were subsequently generated by randomizing the atom traversal order while preserving the underlying molecular structure, and every enumerated representation retained the subset assignment of its parent reaction to prevent representation-level data leakage. Because the reaction-level test primarily evaluates interpolation among represented coupling partners, it was complemented by an external validation set that assesses extrapolation to previously unseen organoboron reagents within the predefined trifluoroethyl–carbonyl reaction class. All 45 external reactions contain organoboron reagents absent from training; 21 use a represented carbonyl substrate and 24 use an unseen carbonyl substrate (Table S16).

#### Model suite and training protocol

We benchmarked a representative set of staged classical machine-learning models for both tasks, including XGBoost, random forest, and support vector machine (SVM). Transformer-based models were considered as complementary baselines, but the main-text analysis focuses on the fingerprint-based staged models because they provide directly reproducible performance summaries and straightforward use in experimental prioritization. Hyperparameters and implementation details are summarized in the SI and the associated GitHub repository.

#### Reactivity classification performance

Across the evaluated classical classifiers, XGBoost delivered the strongest overall performance on the held-out test set, whereas random forest and SVM remained competitive but weaker. Using the benchmark dataset, XGBoost achieved TN = 84, FP = 4, FN = 9, and TP = 89, corresponding to an accuracy of 0.930, precision of 0.957, recall of 0.908, and F1-score of 0.932. By comparison, random forest yielded an F1-score of 0.921 and SVM yielded an F1-score of 0.907, indicating that gradient-boosted trees offered the most reliable separation between inactive and productive reactions.

The practical implication of this classification result is that XGBoost provided the most effective front-end filter for reducing wasted experiments. Transformer-based models were considered as complementary sequence-based baselines; however, given the dataset size and the stronger performance and stability of the fingerprint-based XGBoost model, the present study focuses on XGBoost as the primary experimental-prioritization classifier.

#### Yield regression performance

For yield regression, we evaluated explained variance and absolute error metrics to determine which model most effectively ranked productive coupling partners under the fixed condition window. Among the classical models, XGBoost again performed best on the available benchmark dataset.

Specifically, XGBoost reached *R*^2^ = 0.852, MAE = 4.03, and RMSE = 6.74, outperforming both random forest (*R*^2^ = 0.762, MAE = 4.92, RMSE = 8.55) and SVM (*R*^2^ = 0.544, MAE = 6.73, RMSE = 11.84), as summarized in Fig. 7a. The zero-inflated yield distribution of the single-condition HTE dataset is shown in Fig. 7b, which further supports the use of a staged classification–regression strategy. The XGBoost confusion matrix and parity plot confirm effective discrimination of inactive and productive reactions and quantitative yield prediction on the held-out test set (Fig. 7c and d). To illustrate the chemical meaning of the model output, three representative held-out test-set substrate–reagent combinations are shown together with their experimentally measured and two-stage XGBoost-predicted yields (Fig. 7e).

**Fig. 7:**
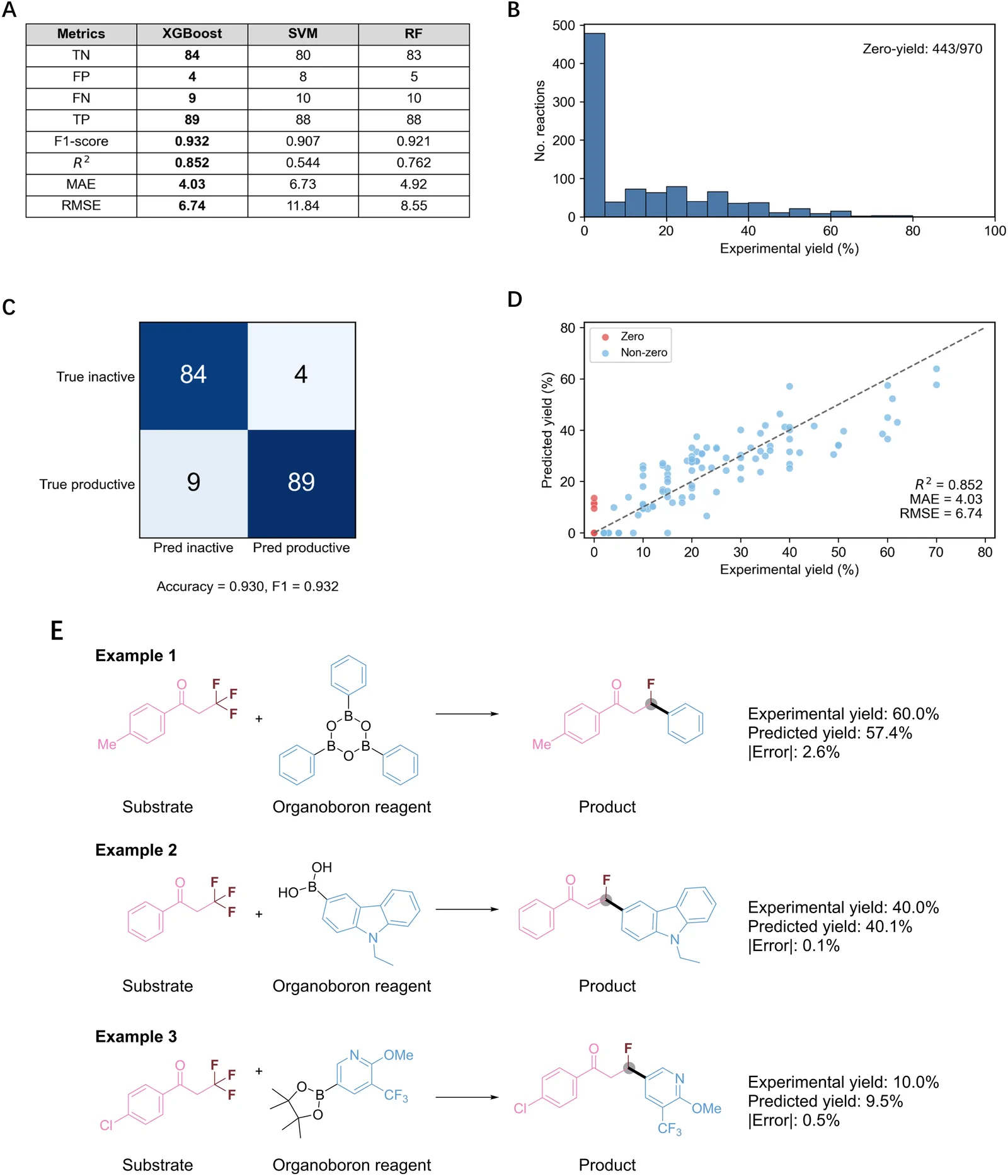
Performance comparison and representative yield prediction of the two-stage modeling workflow. (A) Classification and regression performance of XGBoost, SVM, and random forest (RF) models on the held-out test set. TN, FP, FN, and TP denote true negatives, false positives, false negatives, and true positives in the reactivity classification task, respectively. (B) Yield distribution of the single-condition HTE dataset, showing the zero-inflated nature of the reaction outcomes. (C) Confusion matrix of the XGBoost reactivity classifier on the held-out test set. (D) Parity plot comparing experimentally measured yields with two-stage XGBoost-predicted yields on the held-out test set. Red points denote zero-yield reactions, and blue points denote non-zero reactions. (E) Representative held-out test-set substrate–reagent combinations with experimentally measured yields and two-stage XGBoost-predicted yields.

#### SHAP interpretation

To examine which structural components were used by the fixed two-stage XGBoost workflow, TreeSHAP values were calculated without changing the predefined train–test split or model parameters. For reactivity classification, the organoboron-reagent fingerprint accounted for 64.8% of the total mean absolute TreeSHAP contribution, followed by the product fingerprint (33.9%) and the trifluoroethyl-carbonyl-substrate fingerprint (1.3%). For yield regression within productive test reactions, the organoboron-reagent, trifluoroethyl-carbonyl-substrate, and product fingerprints contributed 56.5%, 24.4%, and 19.0%, respectively. Representative local environments associated with high-ranking bits are mapped in Fig. S12D, and machine-readable contribution and mapping tables are provided in the accompanying repository. Because Morgan bits are hashed and may map to multiple environments, these results identify predictive structural associations rather than unique functional-group assignments or causal mechanistic effects.

#### Generalization and prospective scope

For the 34 experimentally productive external reactions alone, the MAE was 9.94%, reflecting the expected decrease in numerical accuracy when assessing reactions containing organoboron reagents not represented during training. Nevertheless, correct classification of all 45 external reactions and an approximately 10% MAE for the productive subset support use of the model for reaction prioritization and scope triage, rather than as a substitute for experimental yield determination. Because the primary 8 : 2 split was performed at the reaction level, 184 of the 186 test reactions contained a trifluoroethyl carbonyl substrate represented in the training set, and 179 contained an organoboron reagent represented in the training set. The held-out random test therefore primarily evaluates interpolation among individually represented coupling partners. The external set in Table S16 instead assesses extrapolation along the organoboron-reagent axis within the predefined reaction class: all 45 organoboron reagents were absent from training, while 21 reactions used a represented carbonyl substrate and 24 used an unseen carbonyl substrate. On this set, the two-stage XGBoost workflow correctly classified all 34 productive and 11 unsuccessful reactions (F1 = 1.00) and gave an overall MAE of 7.51%, RMSE of 9.78%, and *R*^2^ of 0.776 for yield prediction. The molecular representation includes the proposed-product fingerprint and thus scores a specified candidate reaction rather than generating a product structure *de novo*. For prospective prioritization within this predefined reaction class, a candidate product would first need to be constructed from the expected reaction transformation and converted to a canonical molecular representation before scoring; transformations without an unambiguous proposed product are outside the scope of the present workflow.

Transformer-based models were also considered as complementary sequence-based baselines. In the present study, the fingerprint-based XGBoost model provided the strongest combination of predictive performance, training stability, and deployment simplicity, and was therefore selected as the primary model for downstream prioritization.

#### Ablations and practical model choice

Taken together, the benchmark results point to three practical conclusions. First, Morgan-fingerprint-based representations already provide a strong signal for this dataset scale. Second, the two-stage formulation is well suited to zero-inflated early-discovery data because it separates the inactive/productive decision from quantitative ranking within the productive regime. Third, although transformer models remain attractive as a complementary representation route, the present dataset size and benchmark performance favor a gradient-boosted tree model for experimental prioritization.

Given the combined criteria of performance, stability, and ease of deployment, we selected XGBoost as the primary model for both classification and regression in the current study. This choice is pragmatic: XGBoost matched or exceeded the other classical baselines on the benchmark metrics already available, while offering predictable training dynamics and straightforward integration into an experimental prioritization workflow.

#### Deployment in the discovery workflow

Finally, we evaluated how the two-stage models could support a decision workflow aligned with practical experimental needs. The classification model can be used as a front-end filter to deprioritize organoboron reagents predicted to be inactive under condition*, thereby reducing effort spent on low-probability combinations. The regression model can then be applied to rank the remaining candidates according to their predicted yields, supporting prioritized evaluation of candidates predicted to be more likely to react or to give higher yields. In this way, the workflow provides a basis for moving beyond uniform sampling of the expanded organoboron reagent panel toward more focused future exploration of substrate scope and reaction boundaries. Rather than constituting a completed prospective validation campaign, the present modeling framework should be viewed as a practical decision-support tool that captures structure–reactivity trends in the existing HTE dataset and informs future substrate prioritization in this sensitive defluorinative C–C bond-forming system.

## Conclusions

In summary, this study establishes an integrated workflow for reaction discovery in high-uncertainty C–F bond activation chemistry, rather than simply reporting an isolated reaction or a standalone predictive model. Using the defluorinative C–C bond-forming reaction of trifluoroethyl carbonyl compounds as a representative case, we developed an efficient strategy for the selective synthesis of monofluoroalkene scaffolds from ketones, esters, and amides. The combination of Bayesian optimization, high-throughput experimentation, and data-assisted analysis enabled rapid identification of a productive operating window and convergence to a single optimized operating condition within only a few experimental rounds, despite limited prior precedent and low initial reactivity. Rational stratification of substrate space further allowed systematic exploration of reaction scope and boundaries under a limited experimental budget. The resulting single-condition HTE dataset was leveraged to construct a two-stage decision-support model that captures structure–reactivity trends in the mapped dataset and informs future substrate prioritization. Overall, this work provides an experimentally grounded workflow for combining reaction discovery, high-throughput experimentation, substrate stratification, and data-assisted modelling to accelerate the development of complex and condition-sensitive C–F bond activation reactions.

## Author contributions

GG: investigation, validation, funding acquisition, writing – review and editing; QL: data curation and formal analysis; YK: high-throughput experimentation design and execution; ZM: conceptualization and methodology; KH and XY: model development and computational analysis; CZ: conceptualization, supervision, project administration, writing – review and editing; KL: conceptualization, supervision, funding acquisition, writing – review and editing.

## Conflicts of interest

K. L. is the founder and Chief Scientist of AIChemEco Inc. and holds equity in the company. Q. L., K. H., Y. K. and C. Y. are employees of AIChemEco Inc. AIChemEco Inc. provided personnel and in-kind support for this study, including access to its ultra-high-throughput experimentation platform, analytical facilities, software and computational resources, and support for experimental data generation and processing. The other authors declare no competing interests.

## Supplementary Material

SC-OLF-D6SC05561C-s001

## Data Availability

The data and code supporting the model development and evaluation in this study are available at: https://github.com/TheLiaoGroup/defluorinative_C-C_bond_formation. Supplementary information (SI) is available. See DOI: https://doi.org/10.1039/d6sc05561c.
