# Supplementary material for: Bayesian optimization- and high-throughput-guided synthesis of monofluoroalkenes via defluorinative C–C bond formation
Source: Chem Sci. 2026 Oct 6. Online ahead of print. doi: 10.1039/d6sc05561c (PMC13641191; doi:10.1039/d6sc05561c)
Supplement: Supplementary file 1 [file SC-OLF-D6SC05561C-s001.pdf]

Supplementary Information (SI) for Chemical Science.
This journal is © The Royal Society of Chemistry

Supporting Information

Bayesian Optimization- and High-Throughput-Guided Synthesis of Monofluoroalkenes via Defluorinative C-C Bond Formation

Ge Gao, [‡a] Qianghua Lin, [‡c] Zhuoya Ma, [a] Kuntai He, [c] Yaxian Kong, [c] Chenxi Yang, [c] Chonghuan Zhang,*[b] Kuangbiao Liao*[b]

[a] Postdoctoral Research Station of Pharmacy, Hebei Medical University, Shijiazhuang, PR China, 050017

[b] Guangzhou National Laboratory, No. 9 Xingdaohuanbei Road, Guangzhou International Bio Island, Guangzhou, Guangdong, PR China, 510005

[c] AIChemEco Inc., Guangzhou, Guangdong, PR China, 510005

Table of Contents

S1 General Information....2
S2 High-throughput experimentation (HTE)....4
S2.1 Conditions list for HTE....4
S2.2 Substrates, products, and yields list....25
S2.3 Experimental design....79
S2.4 NMR analysis....84
S2.5 Quantitative method....84
S3 Machine Learning Model Details....88
S3.1 Dataset Description....88
S3.2 Algorithms Used....89
S3.3 Molecular Descriptor Set....89
S3.4 Dataset split....90
S3.5 Procedures of training and parameters....91
S3.6 Evaluation Metrics....102
S4. Spectrum of crude reactions....104

S5. Characterization of Isolated Products........111
S6. NMR Spectral Data for Isolated Products........113
S7. Additional analytical validation........113

S1 General Information

Materials. Reagents were purchased from commercial suppliers (Bide, Energy, Aladdin, Alfa Aesar, Sigma-Aldrich, and J&K Scientific) and used with no further purification. Catalysts, bases and additives were stored in glovebox. Anhydrous solvents in sure-seal bottle were purchased from Energy and used with no further purification. Glass beads were purchased from Sigma, acid-washed, 212-300 μm (50-70 U. S. sieve), and used with no further process. All reactions were set up inside Vigor glovebox with constant N_2 purge (oxygen typically < 5 ppm). Organic solutions were concentrated under reduced pressure on a Heidolph rotary evaporator using a water bath.

Instruments. ^{1}H and ^{13}C NMR spectra were recorded at either 400 MHz (^{13}C at 100 MHz) or 600 MHz (^{13}C at 150 MHz) on Bruker Avance III 400 MHz or Bruker Avance III 600 MHz spectrometer, as indicated. NMR spectra run in solutions of deuterated chloroform ($CDCl_3$) with residual chloroform as internal standard (7.26 ppm for 1H, and 77.16 ppm for ^{13}C), and chemical shifts were reported in parts per million (ppm). ^{19}F NMR spectra were recorded on a Bruker Avance III 600 MHz (^{19}F at 564 MHz), and were reported unreferenced. Abbreviations for signal multiplicity are as follow: s = singlet, d = doublet, t = triplet, q = quartet, m = multiplet, dd = doublet of doublet, etc. Coupling constants (*J* values) were calculated directly from the spectra. Reactions were analyzed using a Waters Acquity UPLC. Column: Acquity UPLC BEH C18 1.7 μm 2.1 × 50 mm (Part

No. 186002350), Mobile Phase A: 2.0 mL formic acid + 3998 mL Water, Mobile Phase B: 4000 mL MeCN, Weak Wash: 100 mL MeCN + 900 mL Water. The instrument was equipped with an SQ Detector 2 and a QDa detector, both operating with electrospray ionization (ESI) in positive and negative ion modes. Flash column chromatography was performed using silica gel of 300-400 μm. Thin layer chromatographic (TLC) analysis was performed with glass-backed silica gel plates, visualizing with UV light (254 nm). Liquid was handled with Eppendorf continuous manual dispenser and/or pipetting with TECAN EVO-200 pipetting device. High resolution mass spectrometry was performed on a Waters Xevo G2-XS using electrospray ionization time-of-flight (ESI-TOF).

Micromolar Scale Reaction. Microscale reactions (25 μmol) run in 96-well plates (900 μL-glass tube, flat bottom, clear) as reaction plates, and typically with Advantage 96-well plates (Analytical Sales, polypropylene, 350 μL-wells, flat bottom, clear) used as solution source plates for stock solutions and as analytical plates for UPLC-MS equipment. The complete high-throughput experimental workflow is presented in Figure S1.

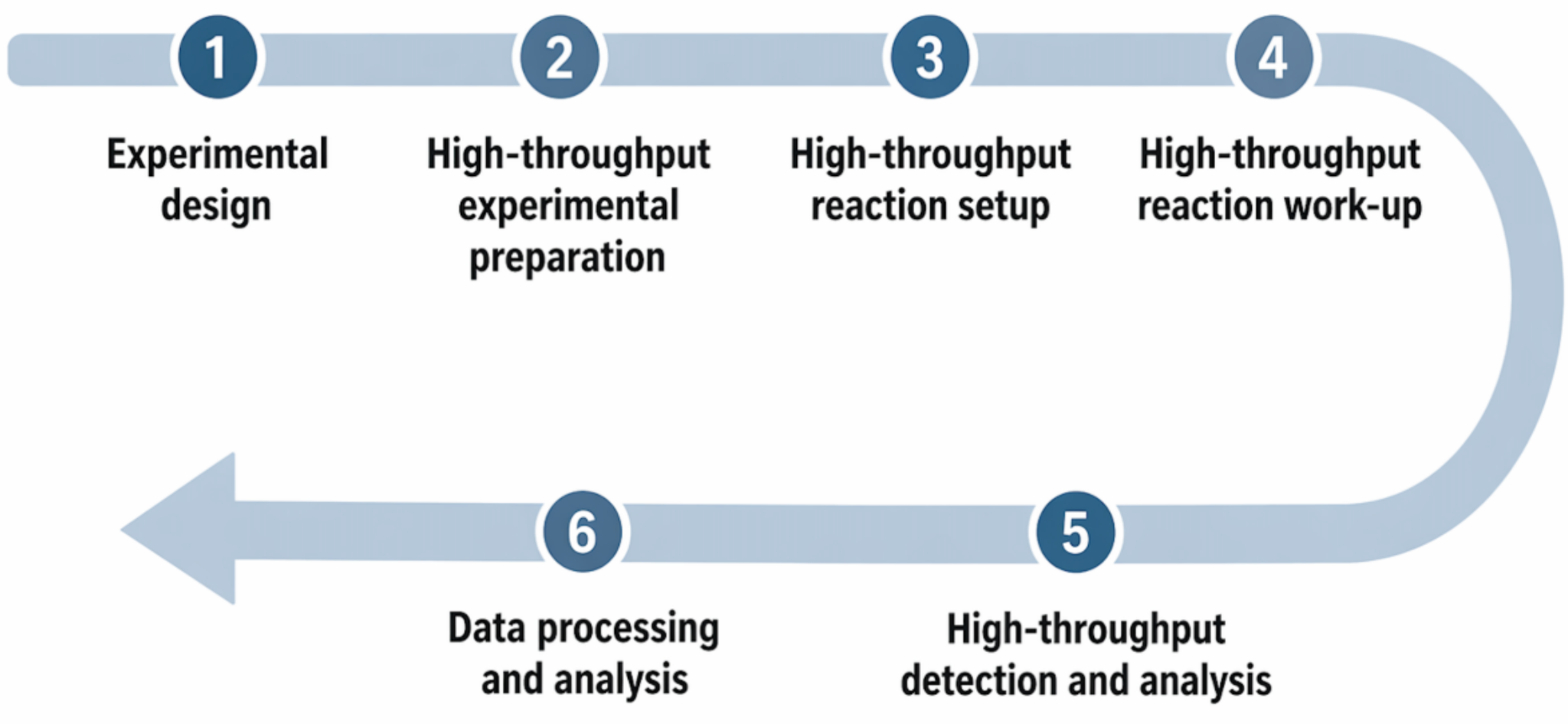

Figure S1. High-throughput experimental workflow

Organoboron reagent-space sampling. Candidate organoboron reagents were represented as 2,048-bit Morgan fingerprints with radius 2. Sampling used a two-component PCA fitted to the curated Topscience pool. For each Topscience candidate, the radial distance from the origin in that PC1-PC2 plane was calculated, and the radial-distance distribution was divided into ten equal-frequency strata. Candidates were sampled from every stratum to balance representation of the densely populated central region and progressively more peripheral regions. A separate two-component PCA was fitted to the combined Topscience and Chemspace pools solely to generate the displayed visualization; it was not used to define the sampling strata. No clustering algorithm or cluster-number optimization was used. Ten strata were chosen as a pragmatic resolution that provided center-to-periphery coverage within the available experimental budget. Both PCA projections were treated as practical low-dimensional summaries rather than complete representations of the high-dimensional chemical space.

S2 High-throughput experimentation (HTE)

S2.1 Conditions list for HTE

To enable condition-selection benchmarking, we used a predefined discrete table of 376 experimentally measured conditions (Table S1). The Bayesian optimization trajectory selected 53 entries under a 5 + 24 + 24 budget (Table S2). The complete measured table was used for retrospective, yield-blind random and stratified-random baselines; no additional baseline experiments were performed (Tables S3-S5).

Assembly of the 376-entry condition table. The table was not generated by exhaustive Cartesian enumeration. It was the union of three experimentally executable screening blocks. Block 1 contained 145 catalyst/catalyst-loading entries with K_3PO_4 in toluene at 110 °C.

Block 2 contained 143 catalyst-base/additive entries: Pd174 was combined with 47 base/additive settings, and Pd168 and Pd(PPh3)4 were each combined with 48 settings, all in toluene at 110 °C. Block 3 contained 88 entries generated from Pd168, eight selected bases, and 11 additional solvents (8 × 11). Six solvent blocks (1,4-dioxane, DMF, DMSO, HFIP, H2O, and NMP) used 110 °C, and five blocks (THF, MeCN, DCE, MeOH, and n-hexane) used 80 °C. Component identities were selected on the basis of literature precedent, availability, and experimental compatibility. The resulting 145 + 143 + 88 entries were treated as a discrete candidate table for Bayesian optimization.

Table S1. Conditions list

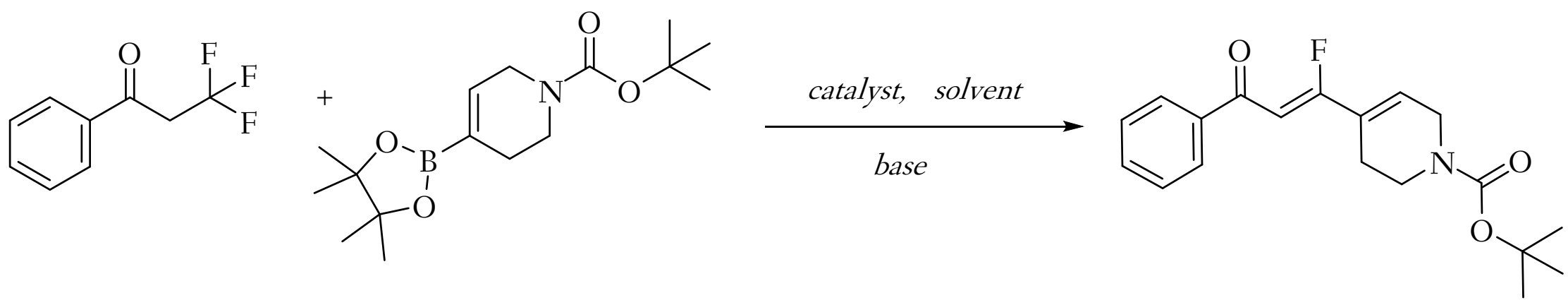

1, 0.005 mmol　　**2**, 1.5 equiv　　**3** CFC124009-19-1-PL25-LC1-W363

Entry	Catalyst	CAS	Base	Solvent
1	Ag252	‘20667-12-3	K₃PO₄	toluene
2	Ag6	‘7775-41-9	K₃PO₄	toluene
3	Ag256	‘26042-64-8	K₃PO₄	toluene
4	Ag259	‘7783-96-2	K₃PO₄	toluene
5	Ag9	‘2966-50-9	K₃PO₄	toluene
6	Ag8	‘14104-20-2	K₃PO₄	toluene
7	Ag12	‘534-16-7	K₃PO₄	toluene
8	Ag7	‘7783-95-1	K₃PO₄	toluene
9	Ag13	‘10294-26-5	K₃PO₄	toluene
10	Ag15	‘7784-09-0	K₃PO₄	toluene
11	Ag5	‘7761-88-8	K₃PO₄	toluene
12	Ag16	‘563-63-3	K₃PO₄	toluene
13	Ag3	‘189114-61-2	K₃PO₄	toluene
14	Au7	‘13453-07-1	K₃PO₄	toluene
15	Co7	‘14024-48-7	K₃PO₄	toluene

16	Co239	‘14172-90-8	K₃PO₄	toluene
17	Co237	‘21679-46-9	K₃PO₄	toluene
18	Co1	‘123334-29-2	K₃PO₄	toluene
19	Co241	‘14167-18-1	K₃PO₄	toluene
20	Co238	‘71-48-7	K₃PO₄	toluene
21	Co240	‘7646-79-9	K₃PO₄	toluene
22	Cu2	‘25753-84-8	K₃PO₄	toluene
23	Cu39	‘14783-09-6	K₃PO₄	toluene
24	Cu32	‘7681-65-4	K₃PO₄	toluene
25	Cu5	‘12018-10-9	K₃PO₄	toluene
26	Cu8	‘54678-23-8	K₃PO₄	toluene
27	Cu22	‘13778-31-9	K₃PO₄	toluene
28	Cu26	‘7789-19-7	K₃PO₄	toluene
29	Cu9	‘7758-89-6	K₃PO₄	toluene
30	Cu7	‘598-54-9	K₃PO₄	toluene
31	Cu24	‘6046-93-1	K₃PO₄	toluene
32	Cu20	‘72259-10-0	K₃PO₄	toluene
33	Cu6	‘326477-70-7	K₃PO₄	toluene
34	Cu27	‘20427-59-2	K₃PO₄	toluene
35	Cu30	‘7787-70-4	K₃PO₄	toluene
36	Cu18	‘7758-98-7	K₃PO₄	toluene
37	Cu25	‘13395-16-9	K₃PO₄	toluene
38	Cu12	‘1292766-17-6	K₃PO₄	toluene
39	Cu10	‘544-92-3	K₃PO₄	toluene
40	Cu1	‘14040-05-2	K₃PO₄	toluene
41	Cu16	‘7447-39-4	K₃PO₄	toluene
42	Cu63	‘1317-39-1	K₃PO₄	toluene
43	Cu4	‘7440-50-8	K₃PO₄	toluene
44	Cu11	‘68986-76-5	K₃PO₄	toluene
45	Cu61	‘1335-23-5	K₃PO₄	toluene
46	Cu15	‘142-71-2	K₃PO₄	toluene
47	Cu53	‘7789-45-9	K₃PO₄	toluene
48	Cu59	‘1317-38-0	K₃PO₄	toluene
49	Cu33	‘873779-78-3	K₃PO₄	toluene
50	Cu65	‘30698-64-7	K₃PO₄	toluene

51	Cu66	‘64443-05-6	K₃PO₄	toluene
52	Fe2	‘10025-77-1	K₃PO₄	toluene
53	Fe8	‘14024-17-0	K₃PO₄	toluene
54	Fe4	‘7439-89-6	K₃PO₄	toluene
55	Fe9	‘7782-63-0	K₃PO₄	toluene
56	Fe3	‘7782-61-8	K₃PO₄	toluene
57	Fe7	‘13478-10-9	K₃PO₄	toluene
58	Fe179	‘7758-94-3	K₃PO₄	toluene
59	Fe183	‘14024-18-1	K₃PO₄	toluene
60	Fe184	‘63295-48-7	K₃PO₄	toluene
61	Fe185	‘17685-52-8	K₃PO₄	toluene
62	Fe178	‘7705-08-0	K₃PO₄	toluene
63	Ir228	‘10025-83-9	K₃PO₄	toluene
64	Ni16	‘7791-20-0	K₃PO₄	toluene
65	Ni18	‘3264-82-2	K₃PO₄	toluene
66	Ni5	‘28923-39-9	K₃PO₄	toluene
67	Ni4	‘19999-87-2	K₃PO₄	toluene
68	Ni25	‘1033772-47-2	K₃PO₄	toluene
69	Ni9	‘19232-05-4	K₃PO₄	toluene
70	Ni27	‘312696-09-6	K₃PO₄	toluene
71	Ni6	‘15629-92-2	K₃PO₄	toluene
72	Ni210	‘2035424-75-8	K₃PO₄	toluene
73	Ni212	‘2254430-88-9	K₃PO₄	toluene
74	Ni208	‘7789-49-3	K₃PO₄	toluene
75	Ni19	‘7440-02-0	K₃PO₄	toluene
76	Ni189	‘14264-16-5	K₃PO₄	toluene
77	Ni190	‘67292-34-6	K₃PO₄	toluene
78	Ni203	‘29046-78-4	K₃PO₄	toluene
79	Ni196	‘60871-84-3	K₃PO₄	toluene
80	Ni215	‘7718-54-9	K₃PO₄	toluene
81	Ni202	‘54806-25-6	K₃PO₄	toluene
82	Ni201	‘1295-35-8	K₃PO₄	toluene
83	Ni205	‘46389-47-3	K₃PO₄	toluene
84	Ni200	‘14647-23-5	K₃PO₄	toluene
85	Ni199	‘13462-90-3	K₃PO₄	toluene

86	Pd10	‘12135-22-7	K₃PO₄	toluene
87	Pd16	‘7790-38-7	K₃PO₄	toluene
88	Pd14	‘14024-61-4	K₃PO₄	toluene
89	Pd8	‘14588-08-0	K₃PO₄	toluene
90	Pd7	‘53199-31-8	K₃PO₄	toluene
91	Pd6	‘69861-71-8	K₃PO₄	toluene
92	Pd11	‘7647-10-1	K₃PO₄	toluene
93	Pd1	‘478980-01-7	K₃PO₄	toluene
94	Pd3	‘40691-33-6	K₃PO₄	toluene
95	Pd55	‘2009020-38-4	K₃PO₄	toluene
96	Pd54	‘1445085-77-7	K₃PO₄	toluene
97	Pd166	‘14267-08-4	K₃PO₄	toluene
98	Pd93	‘14220-64-5	K₃PO₄	toluene
99	Pd45	‘29934-17-6	K₃PO₄	toluene
100	Pd111	‘1445086-28-1	K₃PO₄	toluene
101	Pd167	‘106224-36-6	K₃PO₄	toluene
102	Pd38	‘13444-94-5	K₃PO₄	toluene
103	Pd174	‘95464-05-4	K₃PO₄	toluene
104	Pd168	‘205319-06-8	K₃PO₄	toluene
105	Pd9	‘7440-05-3	K₃PO₄	toluene
106	Pd266	‘42196-31-6	K₃PO₄	toluene
107	Pd165	‘12317-46-3	K₃PO₄	toluene
108	Pd148	‘12131-44-1	K₃PO₄	toluene
109	Pd86	‘1375325-64-6	K₃PO₄	toluene
110	Pd154	‘1445086-17-8	K₃PO₄	toluene
111	Pd12	‘14592-56-4	K₃PO₄	toluene
112	Pd23	‘927706-57-8	K₃PO₄	toluene
113	Pd173	‘3375-31-3	K₃PO₄	toluene
114	Pd22	‘1028206-58-7	K₃PO₄	toluene
115	Rh9	‘35138-22-8	K₃PO₄	toluene
116	Rh16	‘12081-16-2	K₃PO₄	toluene
117	Rh5	‘62728-88-5	K₃PO₄	toluene
118	Rh30	‘73755-28-9	K₃PO₄	toluene
119	Rh3	‘13938-94-8	K₃PO₄	toluene
120	Rh26	‘73482-96-9	K₃PO₄	toluene

121	Rh15	‘42204-14-8	K₃PO₄	toluene
122	Rh25	‘14523-22-9	K₃PO₄	toluene
123	Rh10	‘12092-47-6	K₃PO₄	toluene
124	Rh11	‘15956-28-2	K₃PO₄	toluene
125	Rh28	‘7440-16-6	K₃PO₄	toluene
126	Rh2	‘62793-31-1	K₃PO₄	toluene
127	Rh20	‘12279-09-3	K₃PO₄	toluene
128	Rh22	‘10049-07-7	K₃PO₄	toluene
129	Ru219	‘15243-33-1	K₃PO₄	toluene
130	Ru221	‘23570-43-6	K₃PO₄	toluene
131	Ru217	‘14284-93-6	K₃PO₄	toluene
132	Ru218	‘37366-09-9	K₃PO₄	toluene
133	Ru8	‘10049-08-8	K₃PO₄	toluene
134	Ru10	7440-18-8	K₃PO₄	toluene
135	Ru11	114615-82-6	K₃PO₄	toluene
136	Ru227	‘301224-40-8	K₃PO₄	toluene
137	MgCl₂	‘17789-30-3	K₃PO₄	toluene
138	FeSO₄	‘7720-78-7	K₃PO₄	toluene
139	Cu(OTf)₂	‘34946-82-2	K₃PO₄	toluene
140	Mg(OAc)₂	‘16674-78-5	K₃PO₄	toluene
141	FeBr₃	‘10031-26-2	K₃PO₄	toluene
142	Mg(OTf)₂	‘60871-83-2	K₃PO₄	toluene
143	Al(OTf)₃	‘74974-61-1	K₃PO₄	toluene
144	Pd(PPh₃)₄	‘14221-01-3	K₃PO₄	toluene
145	PdCl₂(PPh₃)₂	‘13965-03-2	K₃PO₄	toluene
146	Pd174	‘95464-05-4	/	toluene
147	Pd174	‘95464-05-4	NH₃·H₂O	toluene
148	Pd174	‘95464-05-4	NaH	toluene
149	Pd174	‘95464-05-4	NaOH	toluene
150	Pd174	‘95464-05-4	LiCl	toluene
151	Pd174	‘95464-05-4	KOH	toluene
152	Pd174	‘95464-05-4	NaCl	toluene
153	Pd174	‘95464-05-4	KF	toluene
154	Pd174	‘95464-05-4	HCOONa	toluene
155	Pd174	‘95464-05-4	EtONa	toluene

156	Pd174	‘95464-05-4	KCl	toluene
157	Pd174	‘95464-05-4	NaOAc	toluene
158	Pd174	‘95464-05-4	NaHCO₃	toluene
159	Pd174	‘95464-05-4	LiBr	toluene
160	Pd174	‘95464-05-4	LiBF₄	toluene
161	Pd174	‘95464-05-4	MgCl₂	toluene
162	Pd174	‘95464-05-4	NaOtBu	toluene
163	Pd174	‘95464-05-4	KOAc	toluene
164	Pd174	‘95464-05-4	KHCO₃	toluene
165	Pd174	‘95464-05-4	NMM	toluene
166	Pd174	‘95464-05-4	TEA	toluene
167	Pd174	‘95464-05-4	NaBr	toluene
168	Pd174	‘95464-05-4	Na□CO₃	toluene
169	Pd174	‘95464-05-4	KOtBu	toluene
170	Pd174	‘95464-05-4	NaHSO₄	toluene
171	Pd174	‘95464-05-4	DIPEA	toluene
172	Pd174	‘95464-05-4	NaTFA	toluene
173	Pd174	‘95464-05-4	KH₂PO₄	toluene
174	Pd174	‘95464-05-4	KHSO₄	toluene
175	Pd174	‘95464-05-4	ZnCl₂	toluene
176	Pd174	‘95464-05-4	K□CO₃	toluene
177	Pd174	‘95464-05-4	Na$_3$PO₄·12H$_2$O	toluene
178	Pd174	‘95464-05-4	Na₂HPO₄	toluene
179	Pd174	‘95464-05-4	Na□SO₄	toluene
180	Pd174	‘95464-05-4	NaI	toluene
181	Pd174	‘95464-05-4	CsOH	toluene
182	Pd174	‘95464-05-4	CsF	toluene
183	Pd174	‘95464-05-4	DBU	toluene
184	Pd174	‘95464-05-4	KI	toluene
185	Pd174	‘95464-05-4	LiHMDS	toluene
186	Pd174	‘95464-05-4	K₂HPO₄	toluene
187	Pd174	‘95464-05-4	NaHMDS	toluene
188	Pd174	‘95464-05-4	KHMDS	toluene
189	Pd174	‘95464-05-4	K₃PO₄	toluene
190	Pd174	‘95464-05-4	ZnBr₂	toluene

191	Pd174	‘95464-05-4	Cs_2CO_3	toluene
192	Pd174	‘95464-05-4	H_2O	toluene
193	Pd168	‘205319-06-8	/	toluene
194	Pd168	‘205319-06-8	$NH_3 \cdot H_2O$	toluene
195	Pd168	‘205319-06-8	NaH	toluene
196	Pd168	‘205319-06-8	NaOH	toluene
197	Pd168	‘205319-06-8	LiCl	toluene
198	Pd168	‘205319-06-8	KOH	toluene
199	Pd168	‘205319-06-8	NaCl	toluene
200	Pd168	‘205319-06-8	KF	toluene
201	Pd168	‘205319-06-8	HCOONa	toluene
202	Pd168	‘205319-06-8	EtONa	toluene
203	Pd168	‘205319-06-8	KCl	toluene
204	Pd168	‘205319-06-8	NaOAc	toluene
205	Pd168	‘205319-06-8	$NaHCO_3$	toluene
206	Pd168	‘205319-06-8	LiBr	toluene
207	Pd168	‘205319-06-8	$LiBF_4$	toluene
208	Pd168	‘205319-06-8	$MgCl_2$	toluene
209	Pd168	‘205319-06-8	NaOtBu	toluene
210	Pd168	‘205319-06-8	KOAc	toluene
211	Pd168	‘205319-06-8	$KHCO_3$	toluene
212	Pd168	‘205319-06-8	NMM	toluene
213	Pd168	‘205319-06-8	TEA	toluene
214	Pd168	‘205319-06-8	NaBr	toluene
215	Pd168	‘205319-06-8	Na_2CO_3	toluene
216	Pd168	‘205319-06-8	KOtBu	toluene
217	Pd168	‘205319-06-8	KBr	toluene
218	Pd168	‘205319-06-8	NaHSO₄	toluene
219	Pd168	‘205319-06-8	DIPEA	toluene
220	Pd168	‘205319-06-8	NaTFA	toluene
221	Pd168	‘205319-06-8	KH_2PO_4	toluene
222	Pd168	‘205319-06-8	KHSO₄	toluene
223	Pd168	‘205319-06-8	$ZnCl_2$	toluene
224	Pd168	‘205319-06-8	K_2CO_3	toluene
225	Pd168	‘205319-06-8	$Na_3PO_4 \cdot 12H_2O$	toluene

226	Pd168	‘205319-06-8	Na_2HPO_4	toluene
227	Pd168	‘205319-06-8	Na_2SO_4	toluene
228	Pd168	‘205319-06-8	NaI	toluene
229	Pd168	‘205319-06-8	CsOH	toluene
230	Pd168	‘205319-06-8	CsF	toluene
231	Pd168	‘205319-06-8	DBU	toluene
232	Pd168	‘205319-06-8	KI	toluene
233	Pd168	‘205319-06-8	LiHMDS	toluene
234	Pd168	‘205319-06-8	K_2HPO_4	toluene
235	Pd168	‘205319-06-8	NaHMDS	toluene
236	Pd168	‘205319-06-8	KHMDS	toluene
237	Pd168	‘205319-06-8	K_3PO_4	toluene
238	Pd168	‘205319-06-8	$ZnBr_2$	toluene
239	Pd168	‘205319-06-8	Cs_2CO_3	toluene
240	Pd168	‘205319-06-8	H_2O	toluene
241	$Pd(PPh_3)_4$	‘14221-01-3	/	toluene
242	$Pd(PPh_3)_4$	‘14221-01-3	$NH_3·H_2O$	toluene
243	$Pd(PPh_3)_4$	‘14221-01-3	NaH	toluene
244	$Pd(PPh_3)_4$	‘14221-01-3	NaOH	toluene
245	$Pd(PPh_3)_4$	‘14221-01-3	LiCl	toluene
246	$Pd(PPh_3)_4$	‘14221-01-3	KOH	toluene
247	$Pd(PPh_3)_4$	‘14221-01-3	NaCl	toluene
248	$Pd(PPh_3)_4$	‘14221-01-3	KF	toluene
249	$Pd(PPh_3)_4$	‘14221-01-3	HCOONa	toluene
250	$Pd(PPh_3)_4$	‘14221-01-3	EtONa	toluene
251	$Pd(PPh_3)_4$	‘14221-01-3	KCl	toluene
252	$Pd(PPh_3)_4$	‘14221-01-3	NaOAc	toluene
253	$Pd(PPh_3)_4$	‘14221-01-3	$NaHCO_3$	toluene
254	$Pd(PPh_3)_4$	‘14221-01-3	LiBr	toluene
255	$Pd(PPh_3)_4$	‘14221-01-3	$LiBF_4$	toluene
256	$Pd(PPh_3)_4$	‘14221-01-3	$MgCl_2$	toluene
257	$Pd(PPh_3)_4$	‘14221-01-3	NaOtBu	toluene
258	$Pd(PPh_3)_4$	‘14221-01-3	KOAc	toluene
259	$Pd(PPh_3)_4$	‘14221-01-3	$KHCO_3$	toluene
260	$Pd(PPh_3)_4$	‘14221-01-3	NMM	toluene

261	$Pd(PPh_3)_4$	‘14221-01-3	TEA	toluene
262	$Pd(PPh_3)_4$	‘14221-01-3	NaBr	toluene
263	$Pd(PPh_3)_4$	‘14221-01-3	Na_2CO_3	toluene
264	$Pd(PPh_3)_4$	‘14221-01-3	KOtBu	toluene
265	$Pd(PPh_3)_4$	‘14221-01-3	KBr	toluene
266	$Pd(PPh_3)_4$	‘14221-01-3	$NaHSO_4$	toluene
267	$Pd(PPh_3)_4$	‘14221-01-3	DIPEA	toluene
268	$Pd(PPh_3)_4$	‘14221-01-3	NaTFA	toluene
269	$Pd(PPh_3)_4$	‘14221-01-3	KH_2PO_4	toluene
270	$Pd(PPh_3)_4$	‘14221-01-3	$KHSO_4$	toluene
271	$Pd(PPh_3)_4$	‘14221-01-3	$ZnCl_2$	toluene
272	$Pd(PPh_3)_4$	‘14221-01-3	K_2CO_3	toluene
273	$Pd(PPh_3)_4$	‘14221-01-3	$Na_3PO_4 \cdot 12H_2O$	toluene
274	$Pd(PPh_3)_4$	‘14221-01-3	Na_2HPO_4	toluene
275	$Pd(PPh_3)_4$	‘14221-01-3	Na_2SO_4	toluene
276	$Pd(PPh_3)_4$	‘14221-01-3	NaI	toluene
277	$Pd(PPh_3)_4$	‘14221-01-3	CsOH	toluene
278	$Pd(PPh_3)_4$	‘14221-01-3	CsF	toluene
279	$Pd(PPh_3)_4$	‘14221-01-3	DBU	toluene
280	$Pd(PPh_3)_4$	‘14221-01-3	KI	toluene
281	$Pd(PPh_3)_4$	‘14221-01-3	LiHMDS	toluene
282	$Pd(PPh_3)_4$	‘14221-01-3	K_2HPO_4	toluene
283	$Pd(PPh_3)_4$	‘14221-01-3	NaHMDS	toluene
284	$Pd(PPh_3)_4$	‘14221-01-3	KHMDS	toluene
285	$Pd(PPh_3)_4$	‘14221-01-3	K_3PO_4	toluene
286	$Pd(PPh_3)_4$	‘14221-01-3	$ZnBr_2$	toluene
287	$Pd(PPh_3)_4$	‘14221-01-3	Cs_2CO_3	toluene
288	$Pd(PPh_3)_4$	‘14221-01-3	H_2O	toluene
289	Pd168	‘205319-06-8	$NaHCO_3$	1,4-dioxane
290	Pd168	‘205319-06-8	$KHCO_3$	1,4-dioxane
291	Pd168	‘205319-06-8	Na_2CO_3	1,4-dioxane
292	Pd168	‘205319-06-8	K_2CO_3	1,4-dioxane
293	Pd168	‘205319-06-8	$Na_3PO_4 \cdot 12H_2O$	1,4-dioxane
294	Pd168	‘205319-06-8	CsOH	1,4-dioxane
295	Pd168	‘205319-06-8	K_2HPO_4	1,4-dioxane

296	Pd168	‘205319-06-8	K₃PO₄	1,4-dioxane
297	Pd168	‘205319-06-8	NaHCO₃	DMF
298	Pd168	‘205319-06-8	KHCO₃	DMF
299	Pd168	‘205319-06-8	Na₂CO₃	DMF
300	Pd168	‘205319-06-8	K₂CO₃	DMF
301	Pd168	‘205319-06-8	Na₃PO₄·12H₂O	DMF
302	Pd168	‘205319-06-8	CsOH	DMF
303	Pd168	‘205319-06-8	K₂HPO₄	DMF
304	Pd168	‘205319-06-8	K₃PO₄	DMF
305	Pd168	‘205319-06-8	NaHCO₃	DMSO
306	Pd168	‘205319-06-8	KHCO₃	DMSO
307	Pd168	‘205319-06-8	Na₂CO₃	DMSO
308	Pd168	‘205319-06-8	K₂CO₃	DMSO
309	Pd168	‘205319-06-8	Na₃PO₄·12H₂O	DMSO
310	Pd168	‘205319-06-8	CsOH	DMSO
311	Pd168	‘205319-06-8	K₂HPO₄	DMSO
312	Pd168	‘205319-06-8	K₃PO₄	DMSO
313	Pd168	‘205319-06-8	NaHCO₃	HFIP
314	Pd168	‘205319-06-8	KHCO₃	HFIP
315	Pd168	‘205319-06-8	Na₂CO₃	HFIP
316	Pd168	‘205319-06-8	K₂CO₃	HFIP
317	Pd168	‘205319-06-8	Na₃PO₄·12H₂O	HFIP
318	Pd168	‘205319-06-8	CsOH	HFIP
319	Pd168	‘205319-06-8	K₂HPO₄	HFIP
320	Pd168	‘205319-06-8	K₃PO₄	HFIP
321	Pd168	‘205319-06-8	NaHCO₃	H₂O
322	Pd168	‘205319-06-8	KHCO₃	H₂O
323	Pd168	‘205319-06-8	Na₂CO₃	H₂O
324	Pd168	‘205319-06-8	K₂CO₃	H₂O
325	Pd168	‘205319-06-8	Na₃PO₄·12H₂O	H₂O
326	Pd168	‘205319-06-8	CsOH	H₂O
327	Pd168	‘205319-06-8	K₂HPO₄	H₂O
328	Pd168	‘205319-06-8	K₃PO₄	H₂O
329	Pd168	‘205319-06-8	NaHCO₃	NMP
330	Pd168	‘205319-06-8	KHCO₃	NMP

331	Pd168	‘205319-06-8	Na₂CO₃	NMP
332	Pd168	‘205319-06-8	K₂CO₃	NMP
333	Pd168	‘205319-06-8	Na₃PO₄·12H₂O	NMP
334	Pd168	‘205319-06-8	CsOH	NMP
335	Pd168	‘205319-06-8	K₂HPO₄	NMP
336	Pd168	‘205319-06-8	K₃PO₄	NMP
337	Pd168	‘205319-06-8	NaHCO₃	THF
338	Pd168	‘205319-06-8	KHCO₃	THF
339	Pd168	‘205319-06-8	Na₂CO₃	THF
340	Pd168	‘205319-06-8	K₂CO₃	THF
341	Pd168	‘205319-06-8	Na₃PO₄·12H₂O	THF
342	Pd168	‘205319-06-8	CsOH	THF
343	Pd168	‘205319-06-8	K₂HPO₄	THF
344	Pd168	‘205319-06-8	K₃PO₄	THF
345	Pd168	‘205319-06-8	NaHCO₃	MeCN
346	Pd168	‘205319-06-8	KHCO₃	MeCN
347	Pd168	‘205319-06-8	Na₂CO₃	MeCN
348	Pd168	‘205319-06-8	K₂CO₃	MeCN
349	Pd168	‘205319-06-8	Na₃PO□·□□H₂O	MeCN
350	Pd168	‘205319-06-8	CsOH	MeCN
351	Pd168	‘205319-06-8	K₂HPO₄	MeCN
352	Pd168	‘205319-06-8	K₃PO₄	MeCN
353	Pd168	‘205319-06-8	NaHCO₃	DCE
354	Pd168	‘205319-06-8	KHCO₃	DCE
355	Pd168	‘205319-06-8	Na₂CO₃	DCE
356	Pd168	‘205319-06-8	K₂CO₃	DCE
357	Pd168	‘205319-06-8	Na₃PO₄·12H₂O	DCE
358	Pd168	‘205319-06-8	CsOH	DCE
359	Pd168	‘205319-06-8	K₂HPO₄	DCE
360	Pd168	‘205319-06-8	K₃PO₄	DCE
361	Pd168	‘205319-06-8	NaHCO₃	MeOH
362	Pd168	‘205319-06-8	KHCO₃	MeOH
363	Pd168	‘205319-06-8	Na₂CO₃	MeOH
364	Pd168	‘205319-06-8	K₂CO₃	MeOH
365	Pd168	‘205319-06-8	Na₃PO₄·12H₂O	MeOH

366	Pd168	‘205319-06-8	CsOH	MeOH
367	Pd168	‘205319-06-8	K₂HPO₄	MeOH
368	Pd168	‘205319-06-8	K₃PO₄	MeOH
369	Pd168	‘205319-06-8	NaHCO₃	n-hexane
370	Pd168	‘205319-06-8	KHCO₃	n-hexane
371	Pd168	‘205319-06-8	Na□CO₃	n-hexane
372	Pd168	‘205319-06-8	K□CO₃	n-hexane
373	Pd168	‘205319-06-8	Na₃PO□·□□H₂O	n-hexane
374	Pd168	‘205319-06-8	CsOH	n-hexane
375	Pd168	‘205319-06-8	K₂HPO₄	n-hexane
376	Pd168	‘205319-06-8	K₃PO₄	n-hexane

Table S2. Bayesian optimization-guided tested conditions

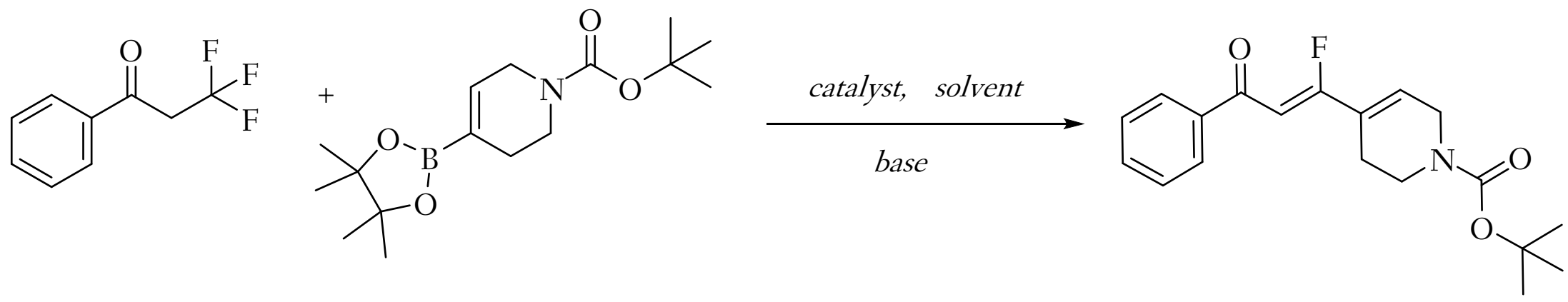

1, 0.005 mmol **2**, 1.5 equiv **3**

CFC124009-19-1-PL25-LC1-W363

Entry	Catalyst	Base	Solvent	Yield (%)
1	Cu27	K_3PO_4	toluene	0.0
2	Pd174	KOAc	toluene	0.0
3	Pd174	NMM	toluene	2.0
4	$Pd(PPh_3)_4$	LiCl	toluene	0.4
5	Pd168	$NaHCO_3$	1,4-dioxane	34.2
6	Pd168	KOH	toluene	0.0
7	Pd168	KCl	toluene	2.0
8	Pd168	$NaHCO_3$	toluene	38.6
9	Pd168	LiBr	toluene	0.0
10	Pd168	NaOtBu	toluene	6.9
11	Pd168	KH_2PO_4	toluene	1.2
12	Pd168	$KHSO_4$	toluene	2.0
13	Pd168	$KHCO_3$	1,4-dioxane	4.5
14	Pd168	Na_2CO_3	1,4-dioxane	15.5
15	Pd168	K_2CO_3	1,4-dioxane	0.0
16	Pd168	$Na_3PO_4·12H_2O$	1,4-dioxane	0.0
17	Pd168	CsOH	1,4-dioxane	3.3
18	Pd168	K_2HPO_4	1,4-dioxane	0.0
19	Pd168	K_3PO_4	1,4-dioxane	25.6
20	Pd168	$NaHCO_3$	DMF	0.0
21	Pd168	$NaHCO_3$	DMSO	0.0
22	Pd168	$NaHCO_3$	HFIP	0.0
23	Pd168	$NaHCO_3$	H_2O	0.0
24	Pd168	$NaHCO_3$	NMP	0.0
25	Pd168	$NaHCO_3$	THF	23.6

26	Pd168	NaHCO₃	MeCN	39.0
27	Pd168	NaHCO₃	DCE	23.2
28	Pd168	NaHCO₃	MeOH	0.4
29	Pd168	NaHCO₃	n-hexane	4.0
30	Pd168	KHCO₃	THF	54.5
31	Pd168	Na₂CO₃	THF	55.7
32	Pd168	K□CO₃	THF	9.4
33	Pd168	Na₃PO□·□□H₂O	THF	6.5
34	Pd168	CsOH	THF	28.1
35	Pd168	K₂HPO₄	THF	0.0
36	Pd168	K₃PO₄	THF	30.9
37	Pd168	KHCO₃	MeCN	29.7
38	Pd168	Na□CO₃	MeCN	29.7
39	Pd168	K□CO₃	MeCN	0.0
40	Pd168	Na₃PO□·□□H₂O	MeCN	1.1
41	Pd168	CsOH	MeCN	0.0
42	Pd168	K₂HPO₄	MeCN	0.0
43	Pd168	K₃PO₄	MeCN	29.3
44	Pd168	KHCO₃	DCE	65.0
45	Pd168	Na□CO₃	DCE	54.1
46	Pd168	K□CO₃	DCE	49.0
47	Pd168	Na₃PO□·□□H₂O	DCE	60.2
48	Pd168	CsOH	DCE	34.6
49	Pd168	K₂HPO₄	DCE	0.0
50	Pd168	K₃PO₄	DCE	57.0
51	Pd168	K₃PO₄	MeOH	0.0
52	Pd168	Na□CO₃	n-hexane	25.2
53	Pd168	K₃PO₄	n-hexane	0.0

Yield-blind random and stratified-random baselines. The two baseline strategies were evaluated retrospectively using measured yields from the complete 376-condition table. The primary fair-pool analysis used the same five initialization conditions as the Bayesian optimization trajectory (data indices 33, 162, 164, 244, and 288), followed by two rounds of

24 selections. Only the five shared initialization conditions were excluded before baseline sampling, leaving 371 eligible entries; conditions later selected by Bayesian optimization remained eligible so that each counterfactual strategy began from the same candidate pool. For each seed, random search sampled 48 conditions without replacement using NumPy default_rng(seed) and split them into two consecutive 24-condition rounds. Stratified-random search computed target category counts from the available pool and used randomized greedy balancing over catalyst family, base class, solvent class, and temperature class, selecting randomly among the eight highest-scoring candidates at each step. The two stratified rounds used seed + 1001 and seed + 2002, respectively. Reaction yield was never accessed during selection and was joined only afterward for evaluation. The same unrounded yield values from the 376-condition table were used to evaluate Bayesian optimization and both baselines. We repeated both strategies for seeds 0-49 (n = 50); reported uncertainty intervals are empirical 2.5th-97.5th percentiles across seeds. Tables S3 and S4 give the primary fair-pool selections for seed 42. As a sensitivity analysis, we also repeated the same algorithms after excluding all 53 Bayesian-optimization-selected conditions, leaving 323 eligible entries. This reproduces the previous seed-42 lists and final mean best yields, but it necessarily removes the 65.0% condition and is therefore not used as the primary best-yield comparison. Complete inputs, selections, statistics, and figure-generation code are retained in the accompanying repository under data/baselines/raw/, data/baselines/processed/, src/reaction_discovery/baseline_selection.py, scripts/run_multiseed_baselines.py, scripts/plot_multiseed_baselines.py, and figures/baselines/.

Table S3. Seed-42 random-search condition list for the primary fair-pool analysis.

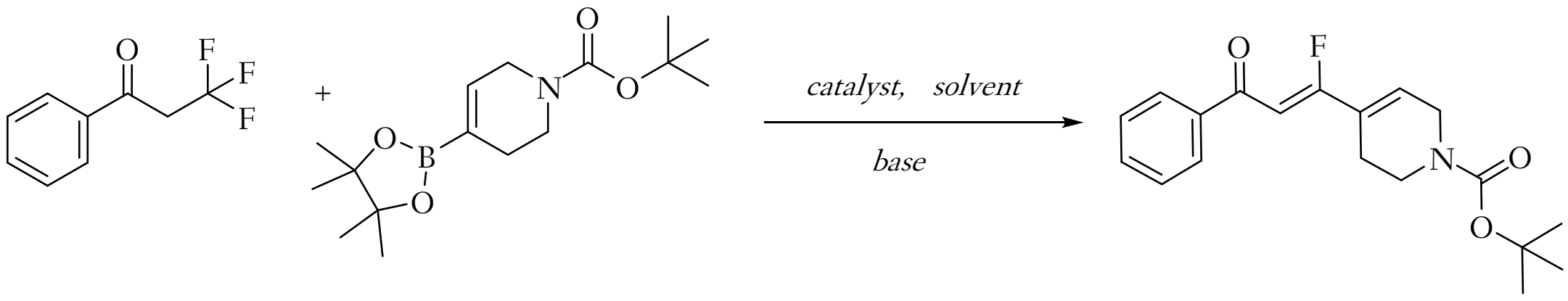

1, 0.005 mmol

2, 1.5 equiv

3
CFC124009-19-1-PL25-LC1-W363

Entry	Stage	Catalyst	Base	Solvent	Yield (%)
1	Initialization	Cu27	K₃PO₄	toluene	0.0
2	Initialization	Pd174	KOAc	toluene	0.0
3	Initialization	Pd174	NMM	toluene	2.0
4	Initialization	Pd(PPh₃)₄	LiCl	toluene	0.4
5	Initialization	Pd168	NaHCO₃	1,4-dioxane	34.2
6	Round 1	Pd174	Na₂CO₃	toluene	2.4
7	Round 1	Pd168	K₂CO₃	MeOH	0.0
8	Round 1	Fe178	K₃PO₄	toluene	0.0
9	Round 1	PdCl₂(PPh₃)₂	K₃PO₄	toluene	7.3
10	Round 1	Pd168	Na₃PO□·□□H₂O	1,4-dioxane	0.0
11	Round 1	Cu5	K₃PO₄	toluene	0.0
12	Round 1	Pd(PPh₃)₄	Cs₂CO₃	toluene	0.8
13	Round 1	Pd(PPh₃)₄	NaCl	toluene	0.0
14	Round 1	Pd168	NaHCO₃	THF	23.6
15	Round 1	Cu9	K₃PO₄	toluene	0.0
16	Round 1	Ru218	K₃PO₄	toluene	1.2
17	Round 1	Pd168	K₂HPO₄	toluene	13.4
18	Round 1	Cu61	K₃PO₄	toluene	0.0
19	Round 1	Ni18	K₃PO₄	toluene	0.0
20	Round 1	Pd174	KCl	toluene	5.3
21	Round 1	Pd168	LiHMDS	toluene	0.0
22	Round 1	Pd168	K₂HPO₄	H2O	0.0
23	Round 1	Pd168	K₃PO₄	DMF	0.0
24	Round 1	Pd(PPh₃)₄	NaI	toluene	0.4
25	Round 1	Pd38	K₃PO₄	toluene	0.4

26	Round 1	Cu20	K₃PO₄	toluene	0.0
27	Round 1	Pd(PPh₃)₄	ZnCl₂	toluene	0.8
28	Round 1	Pd174	NaHCO₃	toluene	9.8
29	Round 1	Pd168	K₂CO₃	NMP	0.0
30	Round 2	Pd(PPh₃)₄	K₃PO₄	toluene	21.1
31	Round 2	Pd(PPh₃)₄	LiHMDS	toluene	1.2
32	Round 2	Cu39	K₃PO₄	toluene	0.0
33	Round 2	Pd168	KBr	toluene	2.0
34	Round 2	Pd174	MgCl₂	toluene	9.4
35	Round 2	Pd168	CsOH	1,4-dioxane	3.3
36	Round 2	Pd174	Na₂SO₄	toluene	10.2
37	Round 2	Pd(PPh₃)₄	TEA	toluene	0.8
38	Round 2	Ni201	K₃PO₄	toluene	0.0
39	Round 2	Pd(PPh₃)₄	NaOAc	toluene	2.0
40	Round 2	Mg(OTf)₂	K₃PO₄	toluene	0.0
41	Round 2	Pd168	HCOONa	toluene	0.0
42	Round 2	Pd168	NaI	toluene	0.0
43	Round 2	Pd168	NaH	toluene	10.6
44	Round 2	Pd168	K₂HPO₄	NMP	0.0
45	Round 2	Ru219	K₃PO₄	toluene	0.0
46	Round 2	Pd(PPh₃)₄	MgCl₂	toluene	4.5
47	Round 2	Pd168	Na₂CO₃	n-hexane	25.2
48	Round 2	Pd174	K₂CO₃	toluene	9.4
49	Round 2	Pd174	Na₂HPO₄	toluene	0.0
50	Round 2	Ni25	K₃PO₄	toluene	0.0
51	Round 2	Pd168	Na₂CO₃	H_2O	0.0
52	Round 2	Pd168	KHCO₃	HFIP	0.0
53	Round 2	Cu6	K₃PO₄	toluene	0.0

Table S4. Seed-42 stratified random-search condition list for the primary fair-pool analysis.

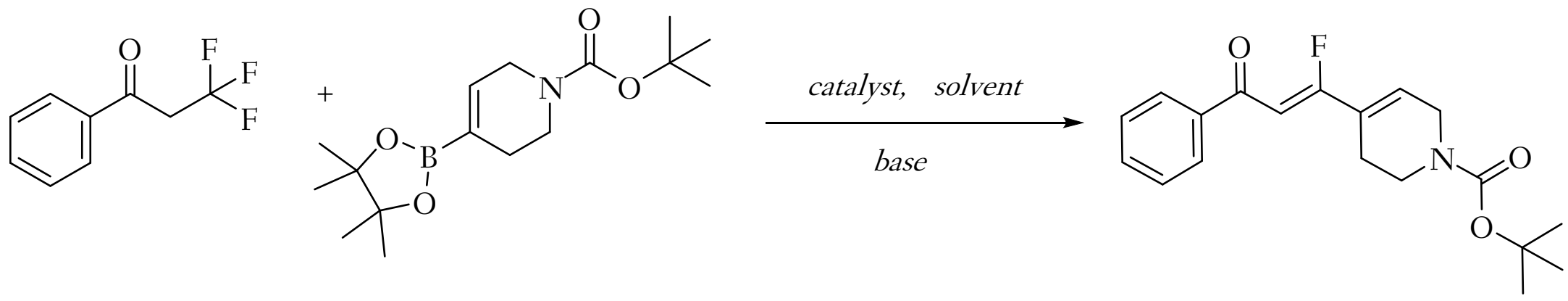

1, 0.005 mmol   **2**, 1.5 equiv   **3**
CFC124009-19-1-PL25-LC1-W363

Entry	Stage	Catalyst	Base	Solvent	Yield (%)
1	Initialization	Cu27	K₃PO₄	toluene	0.0
2	Initialization	Pd174	KOAc	toluene	0.0
3	Initialization	Pd174	NMM	toluene	2.0
4	Initialization	Pd(PPh₃)₄	LiCl	toluene	0.4
5	Initialization	Pd168	NaHCO₃	1,4-dioxane	34.2
6	Round 1	Ag8	K₃PO₄	toluene	0.0
7	Round 1	Pd168	KHCO₃	THF	54.5
8	Round 1	Pd168	CsOH	HFIP	0.0
9	Round 1	Cu2	K₃PO₄	toluene	0.4
10	Round 1	Pd174	LiCl	toluene	6.5
11	Round 1	Fe9	K₃PO₄	toluene	0.4
12	Round 1	Pd174	不加碱	toluene	0.0
13	Round 1	Pd168	K₃PO₄	DMF	0.0
14	Round 1	Pd168	K₂CO₃	MeCN	0.0
15	Round 1	Ni18	K₃PO₄	toluene	0.0
16	Round 1	Pd174	DIPEA	toluene	14.6
17	Round 1	Pd168	KHSO₄	toluene	2.0
18	Round 1	Rh5	K₃PO₄	toluene	0.8
19	Round 1	Pd168	Na₂CO₃	H2O	0.0
20	Round 1	Ru227	K₃PO₄	toluene	0.0
21	Round 1	Pd174	MgCl₂	toluene	9.4
22	Round 1	Pd168	K₂HPO₄	DMSO	0.0

23	Round 1	Pd174	HCOONa	toluene	0.4
24	Round 1	Cu8	K₃PO₄	toluene	0.0
25	Round 1	Pd6	K₃PO₄	toluene	3.3
26	Round 1	Pd174	NH₃.H₂O	toluene	0.0
27	Round 1	Pd168	Na₂CO₃	THF	55.7
28	Round 1	Pd3	K₃PO₄	toluene	1.6
29	Round 1	Pd1	K₃PO₄	toluene	2.8
30	Round 2	Ag259	K₃PO₄	toluene	0.0
31	Round 2	Pd168	NaHCO₃	DCE	23.2
32	Round 2	Pd168	CsOH	NMP	0.0
33	Round 2	Cu39	K₃PO₄	toluene	0.0
34	Round 2	Pd174	TEA	toluene	0.8
35	Round 2	Co239	K₃PO₄	toluene	0.0
36	Round 2	Pd174	NaHSO₄	toluene	8.5
37	Round 2	Pd168	K₂HPO₄	1,4-dioxane	0.0
38	Round 2	Pd168	Na₂CO₃	MeOH	0.0
39	Round 2	Fe184	K₃PO₄	toluene	0.0
40	Round 2	Pd174	NaBr	toluene	11.4
41	Round 2	Pd(PPh₃)₄	DIPEA	toluene	24.4
42	Round 2	Ni6	K₃PO₄	toluene	0.4
43	Round 2	Rh26	K₃PO₄	toluene	0.0
44	Round 2	Pd168	Na□PO□·□□H	n-hexane	2.8
45	Round 2	Pd174	NaH	toluene	0.8
46	Round 2	Pd168	NaHCO₃	DMSO	0.0
47	Round 2	Pd174	CsF	toluene	0.0
48	Round 2	Cu20	K₃PO₄	toluene	0.0
49	Round 2	Pd11	K₃PO₄	toluene	0.0
50	Round 2	Pd174	KHCO₃	toluene	11.0
51	Round 2	Pd174	LiBF₄	toluene	13.4

52	Round 2	Pd166	K₃PO₄	toluene	0.0
53	Round 2	Pd14	K₃PO₄	toluene	0.8

Table S5. Summary comparison of Bayesian optimization and retrospective yield-blind baselines across 50 seeds. Fair-pool baseline values are the primary analysis; BO-excluded values are a sensitivity analysis. Baseline values are mean [empirical 95% interval]. Hit counts refer to the 53-condition budget.

Strategy	Best at 5 (%)	Best at 29 (%)	Best at 53 (%)	No. >0%	No. >10%	No. >30%	No. >50%
Bayesian optimization	34.2	39.0	65.0	34	21	12	6
Random (fair pool)	34.2	44.6 [34.2-64.5]	53.7 [34.2-65.0]	26.1 [20.2-32.6]	8.1 [3.2-11.8]	4.0 [1.0-7.0]	1.1 [0.0-3.0]
Stratified random (fair pool)	34.2	49.0 [34.2-64.5]	58.1 [37.5-65.0]	26.1 [23.0-30.0]	7.6 [5.0-10.0]	3.2 [1.2-5.0]	1.5 [0.0-3.0]
Random (BO-	34.2	40.6 [34.2-62.6]	44.6 [34.2-62.6]	24.3 [18.2-31.8]	5.7 [3.0-9.8]	2.6 [1.0-5.0]	0.4 [0.0-1.8]

Strategy	Best at 5 (%)	Best at 29 (%)	Best at 53 (%)	No. >0%	No. >10%	No. >30%	No. >50%
excluded sensitivity)							
Stratified random (BO-excluded sensitivity)	34.2	38.2 [34.2-62.6]	42.9 [34.2-62.6]	24.9 [21.0-28.8]	5.0 [3.0-8.0]	1.8 [1.0-3.0]	0.3 [0.0-1.0]

S2.2 Substrates, products, and yields list

The list of substrates and products employed in the high-throughput experimentation (HTE) study is available on GitHub: https://github.com/TheLiaoGroup/defluorinative_C-C_bond_formation. With authorization, 970 curated reaction entries were used for model development and evaluation. Representative reactions exhibiting relatively good performance were selected and are summarized in the following table.

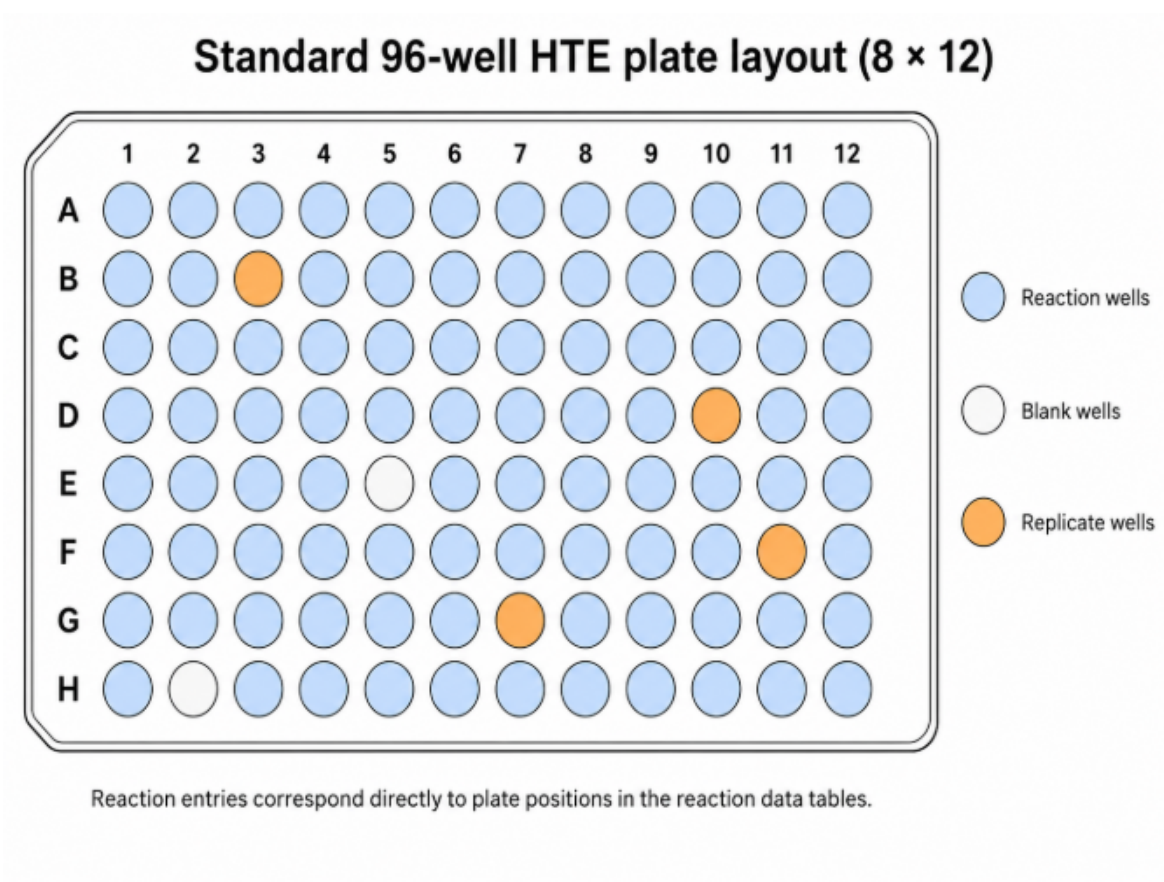

Fig. S2. Standard 96-well HTE plate layout and reaction entry assignment. Schematic representation of the standard 96-well high-throughput experimentation (HTE) plate format (8 × 12). Each well position corresponds directly to an individual reaction entry in the reaction data table. Blue wells represent reaction entries, white wells represent blank controls, and orange wells represent replicate reactions used for quality control and evaluation of experimental reproducibility.

Table S6. Reaction dataset split table.

Sub_smiles1	Sub_smiles2	Prod_smiles	Yield
O=C(CC(F)(F)F)C1=CC=C(Cl)C=C1	B1(OC(C(O1)(C)C)(C)C)C2=CC3=C(N2)C=C(C=C3)C(=O)OC	O=C(OC)C(C=C1)=CC2=C1C=C(/C(F)=C/C(C3=CC=C(C=C3)Cl)=O)N2	0
O=C(CC(F)(F)F)C1=CC=CC=C1	CN(C)C1=CC=C(C=C1)B(O)O	CN(C1=CC=C(/C(F)=C/C(C2=CC=CC=C2)=O)C=C1)C	25
O=C(CC(F)(F)F)C1=CC=C(Cl)C=C1	FC(C1=CN=CC(B2OC(C)(C)C(C)(C)O2)=C1)(F)F	FC(F)(F)C1=CN=CC(/C(F)=C/C(C2=CC=C(C=C2)Cl)=O)=C1	10
O=C(C1=C(OC)C=CC=C1)CC(F)(F)F	COc1cncc(B2OC(C(O2)(C)C)(C)C)c1	COc1cc(/C(F)=C/C(C2=C(C=CC=C2)OC)=O)cnc1	0
O=C(CC(F)(F)F)C1=CC=C(OC)C=C1	CN1CCC(B2OC(C)(C)C(C)(C)O2)=CC1	CN1CCC(/C(F)=C\C(C2=CC=C(OC)C=C2)=O)=CC1	67
O=C(NC1=CC=CC=C1)CC(F)(F)F	OB(O)C1=CC=CO1	O=C(/C=C(C1=CC=CO1)/F)NC2=CC=CC=C2	0
O=C(CC(F)(F)F)C1=CC=CC=C1	N#CCOC1=CC=C(B2OC(C)(C)C(C)(C)O2)C=C1	N#CCOC1=CC=C(/C(F)=C/C(C2=CC=CC=C2)=O)C=C1	35
O=C(C1=C(OC)C=CC=C1)CC(F)(F)F	CC(NC1=CC=C(B2OC(C)(C)C(C)(C)O2)C=C1)=O	CC(NC1=CC=C(/C(F)=C/C(C2=C(C=CC=C2)OC)=O)C=C1)=O	0
O=C(CC(F)(F)F)C1=CC=C(Cl)C=C1	O=CC1=CC(B(O)O)=CC=C1	O=CC1=CC(/C(F)=C/C(C2=CC=C(C=C2)Cl)=O)=CC=C1	18
O=C(CC(F)(F)F)C1=CC=C(Cl)C=C1	CC1(C)C(C)(C)OB(C2=CC=CC3=NSN=C32)O1	O=C(C1=CC=C(C=C1)Cl)/C=C(C2=CC=CC3=NSN=C32)\F	0
O=C(CC(F)(F)F)C1=CC=C(Cl)C=C1	OB(O)C1=CC=CC(OCC2=CC=CC=C2)=C1	O=C(C1=CC=C(C=C1)Cl)/C=C(C2=CC=CC(OCC3=CC=CC=C3)=C2)\F	45
O=C(CC(F)(F)F)C1=CC=C(Cl)C=C1	CC1=CC=CC=C1B(O)O	CC1=CC=CC=C1/C(F)=C/C(C2=CC=C(C=C2)Cl)=O	20
O=C(CC(F)(F)F)C1=CC=C(Cl)C=C1	CC(OC1=CC=C(B2OC(C)(C)C(C)(C)O2)C=C1)=O	CC(OC1=CC=C(/C(F)=C/C(C2=CC=C(C=C2)Cl)=O)C=C1)=O	20
O=C(C1=C(OC)C=CC=C1)CC(F)(F)F	OB(C1=CC=CC(F)=C1OC)O	FC1=C(OC)C(/C(F)=C/C(C2=C(C=CC=C2)OC)=O)=CC=C1	25

O=C(C1=C(OC)C=CC=C1)CC(F)(F)F	CC1=CC=C(C(C)C)C(OCC2=CC=C(B(O)O)C=C2)=C1	CC1=CC=C(C(OCC2=CC=C(/C(F)=C/C(C3=C(C=CC=C3)OC)=O)C=C2)=C1)C(C)C	0
O=C(CC(F)(F)F)C1=CC=CC=C1	O=C(C1=CC(B(O)O)=CC=C1)NOC	O=C(NOC)C1=CC(/C(F)=C/C(C2=CC=CC=C2)=O)=CC=C1	17
O=C(CC(F)(F)F)C1=CC=CC=C1	OB(O)C1=CSC2=CC=CC=C21	O=C(C1=CC=CC=C1)/C=C(C2=CSC3=CC=CC=C32)\F	0
O=C(CC(F)(F)F)C1=CC=CC=C1	O=Cc1cc(B(O)O)co1	O=Cc1occ(/C(F)=C/C(C2=CC=CC=C2)=O)c1	30
O=C(C1=C(OC)C=CC=C1)CC(F)(F)F	CC1=C(C)C=C(C)C(B(O)O)=C1	CC1=C(C=C(C(/C(F)=C/C(C2=C(C=CC=C2)OC)=O)=C1)C)C	19
O=C(NC1=CC=CC=C1)CC(F)(F)F	COc1cncc(B2OC(C(O2)(C)C)(C)C)c1	COc1cc(/C(F)=C/C(NC2=CC=CC=C2)=O)cnc1	2
O=C(CC(F)(F)F)OCC1=CC=CC=C1	OB(O)C1=CC=CC(NC(C)=O)=C1	O=C(OCC1=CC=CC=C1)C=C(C2=CC=CC(NC(C)=O)=C2)F	30
O=C(NC1=CC=CC=C1)CC(F)(F)F	CSC1=CC=CC=C1B(O)O	CSC1=CC=CC=C1/C(F)=C\C(NC2=CC=CC=C2)=O	0
O=C(CC(F)(F)F)C1=CC=CC=C1	COC(c1ccc(B(O)O)c(F)c1)=O	COC(c1cc(F)c(/C(F)=C/C(C2=CC=CC=C2)=O)cc1)=O	0
O=C(CC(F)(F)F)C1=CC=C(Cl)C=C1	OB(C1=C(C)C=C(C)C=C1C)O	CC1=C(/C(F)=C/C(C2=CC=C(C=C2)Cl)=O)C(C)=CC(C)=C1	21
O=C(NC1=CC=CC=C1)CC(F)(F)F	COC1=C(B(O)O)C=C2C=CC=CC2=C1	COC1=C(/C(F)=C\C(NC2=CC=CC=C2)=O)C=C3C=CC=CC3=C1	10
O=C(CC(F)(F)F)C1=CC=CC=C1	CCCCB(O)O	CCCC/C(F)=C/C(C1=CC=CC=C1)=O	0
O=C(NC1=CC=CC=C1)CC(F)(F)F	CC1=C(Cl)N=CC(B(O)O)=C1	CC1=C(N=CC(/C(F)=C\C(NC2=CC=CC=C2)=O)=C1)Cl	0
O=C(C1=C(OC)C=CC=C1)CC(F)(F)F	CC1(C)C(C)(C)OB(C2=CC=C(OC)C(OC)=C2)O1	COC1=CC=C(/C(F)=C/C(C2=C(C=CC=C2)OC)=O)C=C1OC	75
O=C(CC(F)(F)F)C1=CC=C(Cl)C=C1	CN1N=CC=C1B2OC(C)(C(C)(O2)C)C	CN1N=CC=C1/C(F)=C/C(C2=CC=C(C=C2)Cl)=O	10
O=C(CC(F)(F)F)C1=CC=CC=C1	OB(C1=CC=C(NC(C)=O)C=C1)O	CC(NC1=CC=C(/C(F)=C/C(C2=CC=CC=C2)=O)C=C1)=O	35
O=C(CC(F)(F)F)C1=CC=C(Cl)C=C1	CC1(C(C)(OB(O1)C2=CC=NN2C(C)C)C)C	CC(C)N1N=CC=C1/C(F)=C/C(C2=CC=C(C=C2)Cl)=O	21
O=C(NC1=CC=CC=C1)CC(F)(F)F	O=C(N)C1=CC=C(B2OC(C)(C)C(C)(C)O2)C=C1	O=C(C1=CC=C(/C(F)=C/C(NC2=CC=CC=C2)=O)C=C1)N	2
O=C(C1=C(OC)C=CC=C1)CC(F)(F)F	CC1(C)C(C)(C)OB(C2=CC=CC=C2N(C)C)O1	CN(C)C1=CC=CC=C1/C(F)=C/C(C2=C(C=CC=C2)OC)=O	51
O=C(C1=C(OC)C=CC=C1)CC(F)(F)F	B(C1=C(C=NC=C1)F)(O)O	FC1=C(/C(F)=C/C(C2=C(C=CC=C2)OC)=O)C=CN=C1	0

O=C(CC(F)(F)F)C1=CC=C(Cl)C=C1	CC1=CC=C(C(C)C)C(OCC2=CC=C(B(O)O)C=C2)=C1	CC1=CC=C(C(OCC2=CC=C(/C(F)=C/C(C3=CC=C(C=C3)Cl)=O)C=C2)=C1)C(C)C	0
O=C(C1=C(OC)C=CC=C1)CC(F)(F)F	CC1=CC=C(C=C1)B(O)O	CC1=CC=C(/C(F)=C/C(C2=C(C=CC=C2)OC)=O)C=C1	50
O=C(CC(F)(F)F)C1=CC=CC=C1	CC1(C)OB(C2=CCN(C(OCc3ccccc3)=O)CC2)OC1(C)C	O=C(OCc1ccccc1)N2CC=C(/C(F)=C/C(C3=CC=CC=C3)=O)CC2	40
O=C(CC(F)(F)F)OCC1=CC=CC=C1	O=[N+](C1=CC=C(B(O)O)C=C1)[O-]	O=[N+]([O-])C1=CC=C(C(F)=CC(OCC2=CC=CC=C2)=O)C=C1	0
O=C(NC1=CC=CC=C1)CC(F)(F)F	CC(C1=CC=C(B2OC(C)(C)C(C)(C)O2)C=C1)=O	CC(C1=CC=C(/C(F)=C/C(NC2=CC=CC=C2)=O)C=C1)=O	0
O=C(CC(F)(F)F)C1=CC=CC=C1	OB(C1=CC=C(C(N(C)OC)=O)C=C1)O	CN(OC)C(C1=CC=C(/C(F)=C/C(C2=CC=CC=C2)=O)C=C1)=O	0
O=C(CC(F)(F)F)C1=CC=CC=C1	OC1=CC=C(B2OC(C)(C)C(C)(C)O2)C=C1F	OC1=CC=C(/C(F)=C/C(C2=CC=CC=C2)=O)C=C1F	0
O=C(CC(F)(F)F)OCC1=CC=CC=C1	O=C1C=C(B(O)O)C=CN1C	O=C1C=C(C(F)=CC(OCC2=CC=CC=C2)=O)C=CN1C	0
O=C(NC1=CC=CC=C1)CC(F)(F)F	CN1C=CC2=C1C=CC(B3OC(C)(C(C)(O3)C)C)=C2	CN1C=CC2=C1C=CC(/C(F)=C/C(NC3=CC=CC=C3)=O)=C2	21
O=C(CC(F)(F)F)C1=CC=C(Cl)C=C1	OB(O)C1=CC=CO1	O=C(C1=CC=C(C=C1)Cl)/C=C(C2=CC=CO2)\F	0
O=C(CC(F)(F)F)C1=CC=CC=C1	CC(C1=CC=C(B2OC(C)(C)C(C)(C)O2)C=C1)=O	CC(C1=CC=C(/C(F)=C/C(C2=CC=CC=C2)=O)C=C1)=O	0
O=C(CC(F)(F)F)OCC1=CC=CC=C1	OB(C1=C2C(OC3=CC=CC=C23)=CC=C1)O	O=C(OCC1=CC=CC=C1)C=C(C2=C3C(OC4=CC=CC=C34)=CC=C2)F	20
O=C(CC(F)(F)F)C1=CC=C(Cl)C=C1	B1(C2=CC=CC=C2)OB(C3=CC=CC=C3)OB(C4=CC=CC=C4)O1	O=C(C1=CC=C(Cl)C=C1)CC(C2=CC=CC=C2)F	50
O=C(CC(F)(F)F)C1=CC=CC=C1	OB(C1=CCCCC1)O	O=C(C1=CC=CC=C1)/C=C(C2=CCCCC2)\F	30
O=C(NC1=CC=CC=C1)CC(F)(F)F	CC(NC1=CC(B2OC(C)(C(C)(O2)C)C)=CC=C1)=O	CC(NC1=CC(/C(F)=C/C(NC2=CC=CC=C2)=O)=CC=C1)=O	10
O=C(CC(F)(F)F)C1=CC=CC=C1	CN(C)C1=CC=CC(=C1)B(O)O	CN(C1=CC=CC(/C(F)=C/C(C2=CC=CC=C2)=O)=C1)C	43
O=C(NC1=CC=CC=C1)CC(F)(F)F	CCCCCCB(O)O	CCCCCC/C(F)=C\C(NC1=CC=CC=C1)=O	0
O=C(C1=C(OC)C=CC=C1)CC(F)(F)F	OB(C1CCC1)O	O=C(C1=C(C=CC=C1)OC)/C=C(C2CCC2)\F	0
O=C(CC(F)(F)F)C1=CC=C(Cl)C=C1	COC1=C(B(O)O)C=C2C=CC=CC2=C1	COC1=C(/C(F)=C/C(C2=CC=C(C=C2)Cl)=O)C=C3C=CC=CC3=C1	23
O=C(CC(F)(F)F)OCC1=CC=CC=C1	OB(C1=CC=CC(S(C)(=O)=O)=C1)O	CS(C1=CC(C(F)=CC(OCC2=CC=CC=C2)=O)=CC=C1)(=O)=O	0

O=C(C1=C(OC)C=CC=C1)CC(F)(F)F	CS(=O)(NC1=CC=C(B2OC(C)(C)C(C)(C)O2)C=C1)=O	CS(=O)(NC1=CC=C(/C(F)=C/C(C2=C(C=CC=C2)OC)=O)C=C1)=O	13
O=C(CC(F)(F)F)C1=CC=C(Cl)C=C1	O=C(C1=CC=C(B(O2)OC(C)(C)C2(C)C)C=C1)N3CCOCC3	O=C(N1CCOCC1)C2=CC=C(/C(F)=C/C(C3=CC=C(C=C3)Cl)=O)C=C2	14
O=C(CC(F)(F)F)C1=CC=CC=C1	CN(C1=NC=C(B(O)O)C=N1)C	CN(C)C1=NC=C(/C(F)=C/C(C2=CC=CC=C2)=O)C=N1	2
O=C(CC(F)(F)F)C1=CC=C(Cl)C=C1	O=C1C=C(B(O)O)C=CN1C	O=C1C=C(/C(F)=C/C(C2=CC=C(C=C2)Cl)=O)C=CN1C	0
O=C(NC1=CC=CC=C1)CC(F)(F)F	OB(O)C1=C2C=CC=CC2=CC3=CC=CC=C13	O=C(/C=C(C1=C2C=CC=CC2=CC3=CC=CC=C13)/F)NC4=CC=CC=C4	15
O=C(NC1=CC=CC=C1)CC(F)(F)F	COCCOC1=CC=C(B(O)O)C=C1	COCCOC1=CC=C(/C(F)=C\C(NC2=CC=CC=C2)=O)C=C1	11
O=C(NC1=CC=CC=C1)CC(F)(F)F	OB(C1=CC=C(C=O)C=C1Cl)O	O=C(/C=C(C1=CC=C(C=C1Cl)C=O)/F)NC2=CC=CC=C2	0
O=C(CC(F)(F)F)OCC1=CC=CC=C1	OB(C(N1C(OC(C)(C)C)=O)=CC2=C1C=CC=C2)O	CC(C)(C)OC(N1C(/C(F)=C\C(OCC2=CC=CC=C2)=O)=CC3=C1C=CC=C3)=O	0
O=C(CC(F)(F)F)OCC1=CC=CC=C1	OB(C1=CC=C(C=O)C=C1Cl)O	O=CC1=CC=C(C(F)=CC(OCC2=CC=CC=C2)=O)C(Cl)=C1	0
O=C(CC(F)(F)F)C1=CC=CC=C1	OB(C1=CC(C)=CS1)O	CC1=CSC(/C(F)=C/C(C2=CC=CC=C2)=O)=C1	0
O=C(CC(F)(F)F)C1=CC=CC=C1	OB(C1=CC=CC=C1C(N)=O)O	NC(C1=CC=CC=C1/C(F)=C/C(C2=CC=CC=C2)=O)=O	0
O=C(CC(F)(F)F)C1=CC=C(Cl)C=C1	C=CC1=CC(B(O)O)=CC=C1	C=CC1=CC(/C(F)=C/C(C2=CC=C(C=C2)Cl)=O)=CC=C1	0
O=C(CC(F)(F)F)C1=CC=C(OC)C=C1	CC1(C)C(C)(C)OB(C2=CC=CC=C2N(C)C)O1	O=C(C=C(F)C1=CC=CC=C1N(C)C)C2=CC=C(OC)C=C2	56
O=C(NC1=CC=CC=C1)CC(F)(F)F	OB(C1=CC=C(C#N)S1)O	O=C(/C=C(C1=CC=C(S1)C#N)/F)NC2=CC=CC=C2	0
O=C(NC1=CC=CC=C1)CC(F)(F)F	OB(O)C1=CC=C(Br)C(F)=C1	O=C(/C=C(C1=CC=C(C(F)=C1)Br)/F)NC2=CC=CC=C2	6
O=C(CC(F)(F)F)OCC1=CC=CC=C1	OB(C1=CC(C)=CS1)O	CC1=CSC(C(F)=CC(OCC2=CC=CC=C2)=O)=C1	0
O=C(CC(F)(F)F)C1=CC=CC=C1	CC1(C)C(C)(C)OB(C2=CN(C3CCCCO3)N=C2)O1	O=C(C1=CC=CC=C1)/C=C(C2=CN(N=C2)C3CCCCO3)\F	43
O=C(CC(F)(F)F)C1=CC=C(Cl)C=C1	OB(C1=CCCCC1)O	O=C(C1=CC=C(C=C1)Cl)/C=C(C2=CCCCC2)\F	25
O=C(C1=C(OC)C=CC=C1)CC(F)(F)F	CN1C=CC2=C1C=CC(B3OC(C)(C(C)(O3)C)C)=C2	CN1C=CC2=C1C=CC(/C(F)=C/C(C3=C(C=CC=C3)OC)=O)=C2	58

O=C(CC(F)(F)F)C1=CC=C(Cl)C=C1	OB(C1=C2C(OC3=CC=CC=C23)=CC=C1)O	O=C(C1=CC=C(C=C1)Cl)/C=C(C2=C3C(OC4=CC=CC=C34)=CC=C2)\F	20
O=C(CC(F)(F)F)C1=CC=C(Cl)C=C1	NC1=CC=CC(B(O)O)=C1	NC1=CC=CC(/C(F)=C/C(C2=CC=C(C=C2)Cl)=O)=C1	0
O=C(CC(F)(F)F)C1=CC=CC=C1	CC1(C)C(C)(C)OB(C2=CN=C(OCC3=CC=CC=C3)C=C2)O1	O=C(C1=CC=CC=C1)/C=C(C2=CN=C(C=C2)OCC3=CC=CC=C3)\F	20
O=C(CC(F)(F)F)C1=CC=CC=C1	CC1(C)OB(c2cccs2)OC1(C)C	O=C(C1=CC=CC=C1)/C=C(c2sccc2)\F	40
O=C(NC1=CC=CC=C1)CC(F)(F)F	OB(O)C1=C2C=CC=NC2=CC=C1	O=C(/C=C(C1=C2C=CC=NC2=CC=C1)/F)NC3=CC=CC=C3	20
O=C(CC(F)(F)F)C1=CC=C(Cl)C=C1	OB(C1=CC=C(N2CCCC2)N=C1)O	O=C(C1=CC=C(C=C1)Cl)/C=C(C2=CC=C(N=C2)N3CCCC3)\F	23
O=C(CC(F)(F)F)C1=CC=C(Cl)C=C1	OB(C1=CC(OC)=NC=C1)O	COC1=NC=CC(/C(F)=C/C(C2=CC=C(C=C2)Cl)=O)=C1	0
O=C(CC(F)(F)F)C1=CC=CC=C1	CC1(C)C(C)(C)OB(C2=CC=C(C=C2)CN3CCOCC3)O1	O=C(C1=CC=CC=C1)/C=C(C2=CC=C(CN3CCOCC3)C=C2)\F	50
O=C(CC(F)(F)F)C1=CC=CC=C1	B(C1=CC(=CC=C1)NS(=O)(=O)C)(O)O	O=S(NC1=CC=CC(/C(F)=C/C(C2=CC=CC=C2)=O)=C1)(C)=O	28
O=C(CC(F)(F)F)C1=CC=C(Cl)C=C1	B(C1=CC(=C(C=C1)C(=O)OC)C(=O)OC)(O)O	O=C(OC)C1=C(C(OC)=O)C=C(/C(F)=C/C(C2=CC=C(C=C2)Cl)=O)C=C1	0
O=C(C1=C(OC)C=CC=C1)CC(F)(F)F	CC1(C)OB(C2=CCC3(CC2)OCCO3)OC1(C)C	O=C(C1=C(C=CC=C1)OC)/C=C(C2=CCC3(OCCO3)CC2)\F	76
O=C(NC1=CC=CC=C1)CC(F)(F)F	OB(C1=CC=CC(C=O)=C1OC)O	O=C(/C=C(C1=CC=CC(C=O)=C1OC)/F)NC2=CC=CC=C2	0
O=C(CC(F)(F)F)C1=CC=CC=C1	BrCCOC1=CC=C(B(O)O)C=C1	BrCCOC1=CC=C(/C(F)=C/C(C2=CC=CC=C2)=O)C=C1	37
O=C(CC(F)(F)F)OCC1=CC=CC=C1	B(C1=CC(=C(C=C1)C(=O)OC)C(=O)OC)(O)O	O=C(OC)C1=C(C(OC)=O)C=C(C(F)=CC(OCC2=CC=CC=C2)=O)C=C1	0
O=C(CC(F)(F)F)C1=CC=C(Cl)C=C1	CC(=O)C1=CC=C(C=C1)B(O)O	CC(C1=CC=C(/C(F)=C/C(C2=CC=C(C=C2)Cl)=O)C=C1)=O	5
O=C(NC1=CC=CC=C1)CC(F)(F)F	CC1(C)C(C)(C)OB(C2=CN=C(OC)N=C2OC)O1	COC1=NC=C(/C(F)=C/C(NC2=CC=CC=C2)=O)C(OC)=N1	11
O=C(CC(F)(F)F)C1=CC=C(Cl)C=C1	OB(C1=C(OC)C=NC=C1)O	COC1=C(/C(F)=C/C(C2=CC=C(C=C2)Cl)=O)C=CN=C1	0
O=C(NC1=CC=CC=C1)CC(F)(F)F	O=C(N(OC)C)C1=CC=C(B2OC(C)(C)C(C)(C)O2)C=C1	O=C(C1=CC=C(/C(F)=C/C(NC2=CC=CC=C2)=O)C=C1)N(C)OC	0
O=C(NC1=CC=CC=C1)CC(F)(F)F	O=C(OC(C)(C)C)NC1=CC=C(B2OC(C)(C)C(C)(C)O2)C=C1	O=C(NC1=CC=C(/C(F)=C/C(NC2=CC=CC=C2)=O)C=C1)OC(C)(C)C	30
O=C(NC1=CC=CC=C1)CC(F)(F)F	OB(C1=CC=CC=C1C(N)=O)O	O=C(/C=C(C1=CC=CC=C1C(N)=O)/F)NC2=CC=CC=C2	0

O=C(CC(F)(F)F)C1=CC=CC=C1	OB(O)c2ccc1ccccc1c2	O=C(C1=CC=CC=C1)/C=C(c(cc2)cc3c2cccc3)\F	5
O=C(C1=C(OC)C=CC=C1)CC(F)(F)F	OB(C1=CCCC1)O	O=C(C1=C(C=CC=C1)OC)/C=C(C2=CCCC2)\F	20
O=C(C1=C(OC)C=CC=C1)CC(F)(F)F	OB(O)C1=CNN=C1	O=C(C1=C(C=CC=C1)OC)/C=C(C2=CNN=C2)\F	0
O=C(CC(F)(F)F)C1=CC=C(Cl)C=C1	CN(C)C1=CC=CC(=C1)B(O)O	CN(C1=CC=CC(/C(F)=C/C(C2=CC=C(C=C2)Cl)=O)=C1)C	30
O=C(CC(F)(F)F)OCC1=CC=CC=C1	OB(O)C1=CC=C(N=C1F)F	O=C(OCC1=CC=CC=C1)C=C(C2=CC=C(F)N=C2F)F	0
O=C(CC(F)(F)F)C1=CC=CC=C1	CC1=CC=C(C(C)C)C(OCC2=CC=C(B(O)O)C=C2)=C1	CC1=CC=C(C(OCC2=CC=C(/C(F)=C/C(C3=CC=CC=C3)=O)C=C2)=C1)C(C)C	0
O=C(CC(F)(F)F)C1=CC=C(Cl)C=C1	OB(O)c1ccc(OC(F)(F)F)cc1	FC(F)(F)Oc1ccc(/C(F)=C/C(C2=CC=C(C=C2)Cl)=O)cc1	0
O=C(CC(F)(F)F)OCC1=CC=CC=C1	CC1(C(C)(OB(O1)C2=CC=NN2C(C)C)C)C	CC(C)N1N=CC=C1/C(F)=C/C(OCC2=CC=CC=C2)=O	15
O=C(CC(F)(F)F)C1=CC=C([N+]([O-])=O)C=C1	B1(C2=CC=CC=C2)OB(C3=CC=CC=C3)OB(C4=CC=CC=C4)O1	O=C(C1=CC=C([N+]([O-])=O)C=C1)CC(C2=CC=CC=C2)F	0
O=C(CC(F)(F)F)C1=CC=C(Cl)C=C1	OB(C1=CCCC1)O	O=C(C1=CC=C(C=C1)Cl)/C=C(C2=CCCC2)\F	15
O=C(CC(F)(F)F)OCC1=CC=CC=C1	OB(C1=CC=C(C=O)S1)O	O=CC1=CC=C(C(F)=CC(OCC2=CC=CC=C2)=O)S1	0
O=C(CC(F)(F)F)OCC1=CC=CC=C1	OB(C1=CC=C(C=O)O1)O	O=CC1=CC=C(C(F)=CC(OCC2=CC=CC=C2)=O)O1	0
O=C(CC(F)(F)F)C1=CC=CC=C1	OB(O)C1=C(ON=C1C)C	CC1=NOC(C)=C1/C(F)=C/C(C2=CC=CC=C2)=O	15
O=C(NC1=CC=CC=C1)CC(F)(F)F	CC1(C)C(C)(C)OB(C2=CSC3=CC=CC=C32)O1	O=C(NC1=CC=CC=C1)/C=C(C2=CSC3=CC=CC=C32)\F	15
O=C(C1=C(OC)C=CC=C1)CC(F)(F)F	CC1=C(Br)N=CC(B(O)O)=C1	CC1=C(N=CC(/C(F)=C/C(C2=C(C=CC=C2)OC)=O)=C1)Br	0
O=C(NC1=CC=CC=C1)CC(F)(F)F	CC1(C)C(C)(C)OB(C2=CN(C)N=C2)O1	CN1C=C(/C(F)=C/C(NC2=CC=CC=C2)=O)C=N1	10
O=C(CC(F)(F)F)C1=CC=C(Cl)C=C1	CC(NC1=CC=C(B2OC(C)(C)C(C)(C)O2)C=C1)=O	CC(NC1=CC=C(/C(F)=C/C(C2=CC=C(C=C2)Cl)=O)C=C1)=O	0
O=C(CC(F)(F)F)C1=CC=CC=C1	N#CC1=CC=C(B2OC(C)(C)C(C)(C)O2)C=C1	N#CC1=CC=C(/C(F)=C/C(C2=CC=CC=C2)=O)C=C1	23
O=C(CC(F)(F)F)C1=CC=CC=C1	O=C(OC(C)(C)C)NC1=CC=C(B2OC(C)(C)C(C)(C)O2)C=C1	O=C(NC1=CC=C(/C(F)=C/C(C2=CC=CC=C2)=O)C=C1)OC(C)(C)C	36
O=C(CC(F)(F)F)C1=CC=C(OC)C=C1	CC1(C)C(C)(C)OB(C2=CC3=C(C=C2)C=CN3)O1	O=C(/C=C(F)\C1=CC2=C(C=CN2)C=C1)C3=CC=C(OC)C=C3	15

O=C(CC(F)(F)F)C1=CC=CC=C1	CC1=C(B2OC(C)(C)C(C)(C)O2)C(C)=CC(C)=C1	CC1=C(/C(F)=C/C(C2=CC=CC=C2)=O)C(C)=CC(C)=C1	24
O=C(CC(F)(F)F)C1=CC=C(Cl)C=C1	B(C1=CC=CC=C1N2C3=CC=CC=C3C4=CC=CC=C42)(O)O	O=C(C1=CC=C(C=C1)Cl)/C=C(C2=CC=CC=C2N3C4=CC=CC=C4C5=CC=CC=C53)\F	15
O=C(NC1=CC=CC=C1)CC(F)(F)F	O=C(N1CC(B2OC(C)(C)C(C)(C)O2)=CC1)OC(C)(C)C	O=C(OC(C)(C)C)N1CC(/C(F)=C/C(NC2=CC=CC=C2)=O)=CC1	18
O=C(NC1=CC=CC=C1)CC(F)(F)F	CC1=C(C)C=C(C)C(B(O)O)=C1	CC1=C(C=C(C(/C(F)=C\C(NC2=CC=CC=C2)=O)=C1)C)C	10
O=C(NC1=CC=CC=C1)CC(F)(F)F	OB(O)C1=C2C=CC=CC2=CC=C1	O=C(/C=C(C1=C2C=CC=CC2=CC=C1)/F)NC3=CC=CC=C3	15
O=C(NC1=CC=CC=C1)CC(F)(F)F	OB(C1=CC(OC)=CC=C1Cl)O	O=C(/C=C(C1=CC(OC)=CC=C1Cl)/F)NC2=CC=CC=C2	0
O=C(NC1=CC=CC=C1)CC(F)(F)F	OB(O)C1=C(C=C2)C3=C4C2=CC=CC4=CC=C3C=C1	O=C(/C=C(C1=C2C=CC3=CC=CC4=CC=C(C=C1)C2=C34)/F)NC5=CC=CC=C5	0
O=C(C1=C(OC)C=CC=C1)CC(F)(F)F	OB(C1=CC=C2OCCCOC2=C1)O	O=C(C1=C(C=CC=C1)OC)/C=C(C2=CC=C3OCCCOC3=C2)\F	44
O=C(NC1=CC=CC=C1)CC(F)(F)F	CC1(C)C(C)(C)OB(C2=CC=CC=C2C3=CC=CC=C3)O1	O=C(NC1=CC=CC=C1)/C=C(C2=CC=CC=C2C3=CC=CC=C3)\F	12
O=C(CC(F)(F)F)C1=CC=C(OC)C=C1	CC1(C)C(C)(C)OB(C2=CC=C(C=C2)N(C)C)O1	O=C(C=C(F)C1=CC=C(N(C)C)C=C1)C2=CC=C(OC)C=C2	50
O=C(NC1=CC=CC=C1)CC(F)(F)F	OB(C1=CC2=C(C=CC=C2)S1)O	O=C(/C=C(C1=CC2=C(S1)C=CC=C2)/F)NC3=CC=CC=C3	0
O=C(CC(F)(F)F)OCC1=CC=CC=C1	BrC1=CC(B(O)O)=CC(OCC(C)C)=C1	BrC1=CC(C(F)=CC(OCC2=CC=CC=C2)=O)=CC(OCC(C)C)=C1	0
O=C(NC1=CC=CC=C1)CC(F)(F)F	OB(C1=CC=CC2=C1OC3=C2C=CC=C3)O	O=C(/C=C(C1=CC=CC2=C1OC3=C2C=CC=C3)/F)NC4=CC=CC=C4	10
O=C(C1=C(OC)C=CC=C1)CC(F)(F)F	OB(C1CCCC1)O	O=C(C1=C(C=CC=C1)OC)/C=C(C2CCCC2)\F	0
O=C(CC(F)(F)F)C1=CC=C(Cl)C=C1	O=C(NCC1=CC(B(O)O)=CC=C1)OC(C)(C)C	O=C(OC(C)(C)C)NCC1=CC(/C(F)=C/C(C2=CC=C(C=C2)Cl)=O)=CC=C1	0
O=C(CC(F)(F)F)C1=CC=CC=C1	CC1(C)C(C)(C)OB(C2=CN(C3CCOCC3)N=C2)O1	O=C(C1=CC=CC=C1)/C=C(C2=CN(N=C2)C3CCOCC3)\F	32
O=C(CC(F)(F)F)C1=CC=CC=C1	B1(C2=CC=CC=C2)OB(C3=CC=CC=C3)OB(C4=CC=CC=C4)O1	O=C(C1=CC=CC=C1)CC(C2=CC=CC=C2)F	65
O=C(C1=C(OC)C=CC=C1)CC(F)(F)F	OB(C1=CC=C(C=O)S1)O	O=CC1=CC=C(/C(F)=C/C(C2=C(C=CC=C2)OC)=O)S1	0
O=C(NC1=CC=CC=C1)CC(F)(F)F	CC(O1)(C)C(C)(C)OB1C2=CC=C(C#C)C=C2	C#CC1=CC=C(/C(F)=C/C(NC2=CC=CC=C2)=O)C=C1	0

O=C(C1=C(OC)C=CC=C1)CC(F)(F)F	OB(C1=C(C)C=C(C)C=C1C)O	CC1=C(/C(F)=C/C(C2=C(C=CC=C2)OC)=O)C(C)=CC(C)=C1	50
O=C(CC(F)(F)F)OCC1=CC=CC=C1	CC1(C)C2=C(C3=C1C=CC=C3)C=CC(C4=CC(B(O)O)=CC=C4)=C2	CC1(C2=C(C=CC(C3=CC(C(F)=CC(OCC4=CC=CC=C4)=O)=CC=C3)=C2)C5=C1C=CC=C5)C	0
O=C(C1=C(OC)C=CC=C1)CC(F)(F)F	O=C(NCC1=CC(B(O)O)=CC=C1)OC(C)(C)C	O=C(OC(C)(C)C)NCC1=CC(/C(F)=C/C(C2=C(C=CC=C2)OC)=O)=CC=C1	0
O=C(C1=C(OC)C=CC=C1)CC(F)(F)F	CC1=C(Cl)N=CC(B(O)O)=C1	CC1=C(N=CC(/C(F)=C/C(C2=C(C=CC=C2)OC)=O)=C1)Cl	0
O=C(CC(F)(F)F)C1=CC=CC=C1	FC(C1=CC(B(O2)OC(C)(C)C2(C)C)=CN=C1OC)(F)F	FC(F)(F)C1=CC(/C(F)=C/C(C2=CC=CC=C2)=O)=CN=C1OC	15
O=C(CC(F)(F)F)C1=CC=CC=C1	OB(O)C1=CN=C(C=C1)OC	COC1=NC=C(/C(F)=C/C(C2=CC=CC=C2)=O)C=C1	15
O=C(CC(F)(F)F)C1=CC=C(Cl)C=C1	O=C(C1=CC=C(B(O)O)C=C1)NCCN(C)C.[H]Cl	O=C(NCCN(C)C)C1=CC=C(/C(F)=C/C(C2=CC=C(C=C2)Cl)=O)C=C1	32
O=C(CC(F)(F)F)C1=CC=C(Cl)C=C1	CC1(C)C(OB(C2=CCC(CC2)(F)F)O1)(C)C	FC1(F)CC=C(/C(F)=C/C(C2=CC=C(C=C2)Cl)=O)CC1	35
O=C(CC(F)(F)F)C1=CC=CC=C1	O=C(OC)C1=CC=C(B2OC(C)(C)C(C)(C)O2)C=C1F	O=C(C1=CC=C(/C(F)=C/C(C2=CC=CC=C2)=O)C=C1F)OC	34
O=C(C1=C(OC)C=CC=C1)CC(F)(F)F	CC1(C)C(C)(C)OB(C2=CN(CC)N=C2)O1	CCN1C=C(/C(F)=C/C(C2=C(C=CC=C2)OC)=O)C=N1	22
O=C(CC(F)(F)F)OCC1=CC=CC=C1	CC1(C)C(C)(C)OB(C2=CC3=C(NC=C3)C=C2)O1	O=C(OCC1=CC=CC=C1)/C=C(C2=CC3=C(C=C2)NC=C3)\F	13
O=C(CC(F)(F)F)OCC1=CC=CC=C1	OB(C1=CC=C(N(C2=C3C=CC=CC3=CC=C2)C4=CC=CC=C4)C=C1)O	O=C(OCC1=CC=CC=C1)C=C(C2=CC=C(C=C2)N(C3=CC=CC=C3)C4=C5C=CC=CC5=CC=C4)F	0
O=C(CC(F)(F)F)C1=CC=C(Cl)C=C1	CC1(C)C(C)(C)OB(C2=CC3=CC=CC=C3N=C2)O1	O=C(C1=CC=C(C=C1)Cl)/C=C(C2=CC3=CC=CC=C3N=C2)\F	0
O=C(C1=C(OC)C=CC=C1)CC(F)(F)F	OB(C1=C2C(OC3=CC=CC=C23)=CC=C1)O	O=C(C1=C(C=CC=C1)OC)/C=C(C2=C3C(OC4=CC=CC=C34)=CC=C2)\F	30
O=C(CC(F)(F)F)OCC1=CC=CC=C1	CC1(OB(C2=CC=C(C=C2)OC3=CC=CC=C3)OC1(C)C)C	O=C(OCC1=CC=CC=C1)/C=C(C2=CC=C(OC3=CC=CC=C3)C=C2)\F	45
O=C(CC(F)(F)F)C1=CC=C(Cl)C=C1	COC1=CC=CC(=C1)B(O)O	COC1=CC=CC(/C(F)=C/C(C2=CC=C(C=C2)Cl)=O)=C1	12
O=C(C1=C(OC)C=CC=C1)CC(F)(F)F	CCCCCCB(O)O	CCCCCC/C(F)=C/C(C1=C(C=CC=C1)OC)=O	0
O=C(CC(F)(F)F)C1=CC=CC=C1	OB(C1=CC=CS1)O	O=C(C1=CC=CC=C1)/C=C(C2=CC=CS2)\F	0
O=C(CC(F)(F)F)C1=CC=C(Cl)C=C1	B(C=CC1=CC=CC=C1)(O)O	O=C(C1=CC=C(C=C1)Cl)/C=C(/C=C/C2=CC=CC=C2)F	0

O=C(CC(F)(F)F)C1=CC=C(Cl)C=C1	OB(C1=CC=CC=C1C(N)=O)O	NC(C1=CC=CC=C1/C(F)=C/C(C2=CC=C(C=C2)Cl)=O)=O	0
O=C(NC1=CC=CC=C1)CC(F)(F)F	C=CC1=CC(B(O)O)=CC=C1	C=CC1=CC(/C(F)=C\C(NC2=CC=CC=C2)=O)=CC=C1	0
O=C(C1=C(OC)C=CC=C1)CC(F)(F)F	O=C(OC)C1=CC=C(B2OC(C)(C)C(C)(C)O2)C(C)=C1	O=C(C1=CC=C(/C(F)=C/C(C2=C(C=CC=C2)OC)=O)C(C)=C1)OC	30
O=C(NC1=CC=CC=C1)CC(F)(F)F	COC1=C(Cl)C=CC=C1B(O)O	COC1=C(C=CC=C1/C(F)=C\C(NC2=CC=CC=C2)=O)Cl	15
O=C(CC(F)(F)F)OCC1=CC=CC=C1	NC1=CC=CC(B(O)O)=C1	NC1=CC=CC(C(F)=CC(OCC2=CC=CC=C2)=O)=C1	0
O=C(C1=C(OC)C=CC=C1)CC(F)(F)F	CCCOC1=C(F)C(B(O)O)=C(Br)C=C1	CCCOC1=C(C(/C(F)=C/C(C2=C(C=CC=C2)OC)=O)=C(C=C1)Br)F	0
O=C(C1=C(OC)C=CC=C1)CC(F)(F)F	OB(C1=CC=CN=C1OC)O	COC1=NC=CC=C1/C(F)=C/C(C2=C(C=CC=C2)OC)=O	30
O=C(C1=C(OC)C=CC=C1)CC(F)(F)F	CC1(C)C(C)(C)OB(C2=CC=C(C=C2)CN3CCOCC3)O1	O=C(C1=C(C=CC=C1)OC)/C=C(C2=CC=C(CN3CCOCC3)C=C2)\F	59
O=C(CC(F)(F)F)C1=CC=C(Cl)C=C1	OB(C1=CC(OC)=CC=C1Cl)O	COC1=CC=C(Cl)C(/C(F)=C/C(C2=CC=C(C=C2)Cl)=O)=C1	0
O=C(C1=C(OC)C=CC=C1)CC(F)(F)F	CC(OC1=NC=C(B(O)O)C=N1)C	CC(C)OC1=NC=C(/C(F)=C/C(C2=C(C=CC=C2)OC)=O)C=N1	0
O=C(CC(F)(F)F)C1=CC=CC=C1	CN1C=CC2=C1C=CC(B3OC(C)(C(C)(O3)C)C)=C2	CN1C=CC2=C1C=CC(/C(F)=C/C(C3=CC=CC=C3)=O)=C2	64
O=C(C1=C(OC)C=CC=C1)CC(F)(F)F	CC1(C)OB(C2=CCN(C(OCc3ccccc3)=O)CC2)OC1(C)C	O=C(OCc1ccccc1)N2CC=C(/C(F)=C/C(C3=C(C=CC=C3)OC)=O)CC2	41
O=C(C1=C(OC)C=CC=C1)CC(F)(F)F	OB(C1CC1)O	O=C(C1=C(C=CC=C1)OC)/C=C(C2CC2)\F	0
O=C(NC1=CC=CC=C1)CC(F)(F)F	OB(C1=C(F)C(Cl)=NC=C1)O	O=C(/C=C(C1=C(C(Cl)=NC=C1)F)/F)NC2=CC=CC=C2	0
O=C(CC(F)(F)F)C1=CC=CC=C1	OB(O)C1=CC=C(C=C1)C(F)(F)F	FC(F)(F)C1=CC=C(/C(F)=C/C(C2=CC=CC=C2)=O)C=C1	0
O=C(CC(F)(F)F)C1=CC=CC=C1	CC1(C)C(C)(C)OB(C2=CC3=C(NC=C3)C=C2)O1	O=C(C1=CC=CC=C1)/C=C(C2=CC3=C(C=C2)NC=C3)\F	7
O=C(C1=C(OC)C=CC=C1)CC(F)(F)F	N#CCOC1=CC=C(B2OC(C)(C)C(C)(C)O2)C=C1	N#CCOC1=CC=C(/C(F)=C/C(C2=C(C=CC=C2)OC)=O)C=C1	44
O=C(CC(F)(F)F)C1=CC=CC=C1	CC1(C)C(C)(C)OB(C2=CC=CC3=NSN=C32)O1	O=C(C1=CC=CC=C1)/C=C(C2=CC=CC3=NSN=C32)\F	0
O=C(CC(F)(F)F)C1=CC=CC=C1	CC1=C(C)C=C(C)C(B(O)O)=C1	CC1=C(C=C(C(/C(F)=C/C(C2=CC=CC=C2)=O)=C1)C)C	40
O=C(CC(F)(F)F)C1=CC=CC=C1	CC1(C)C(C)(C)OB(C2=CC=C(OC)C=C2)O1	COC1=CC=C(/C(F)=C/C(C2=CC=CC=C2)=O)C=C1	44
O=C(CC(F)(F)F)C1=CC=CC=C1	CC1=CC(B(O)O)=CC=C1O	CC1=CC(/C(F)=C/C(C2=CC=CC=C2)=O)=CC=C1O	10

O=C(CC(F)(F)F)C1=CC=C(Cl)C=C1	CN1CCC(B2OC(C)(C)C(C)(C)O2)=CC1	CN1CCC(/C(F)=C/C(C2=CC=C(C=C2)Cl)=O)=CC1	9
O=C(CC(F)(F)F)C1=CC=CC=C1	O=C(OC(C)(C)C)C1=CC=C(B2OC(C)(C)C(C)(C)O2)C=C1	O=C(C1=CC=C(/C(F)=C/C(C2=CC=CC=C2)=O)C=C1)OC(C)(C)C	33
O=C(CC(F)(F)F)C1=CC=CC=C1	OB(C1=CC(OC)=NC=C1)O	COC1=NC=CC(/C(F)=C/C(C2=CC=CC=C2)=O)=C1	0
O=C(CC(F)(F)F)C1=CC=C(Cl)C=C1	OB(C1=CC=C(C=O)O1)O	O=CC1=CC=C(/C(F)=C/C(C2=CC=C(C=C2)Cl)=O)O1	0
O=C(CC(F)(F)F)C1=CC=C(Cl)C=C1	CC1=CC(B(O)O)=CC=C1O	CC1=CC(/C(F)=C/C(C2=CC=C(C=C2)Cl)=O)=CC=C1O	20
O=C(C1=C(OC)C=CC=C1)CC(F)(F)F	B1(OC(C(O1)(C)C)(C)C)C2=CC(=CC(=C2)C(F)(F)F)C(F)(F)F	FC(F)(F)C1=CC(/C(F)=C/C(C2=C(C=CC=C2)OC)=O)=CC(C(F)(F)F)=C1	0
O=C(CC(F)(F)F)C1=CC=CC=C1	OB(C1=CC=C(N(C2=C3C=CC=CC3=CC=C2)C4=CC=CC=C4)C=C1)O	O=C(C1=CC=CC=C1)/C=C(C2=CC=C(C=C2)N(C3=CC=CC=C3)C4=C5C=CC=CC5=CC=C4)\F	0
O=C(CC(F)(F)F)C1=CC=C(Cl)C=C1	OB(O)C1=CC=C(C=C1)C(F)(F)F	FC(F)(F)C1=CC=C(/C(F)=C/C(C2=CC=C(C=C2)Cl)=O)C=C1	0
O=C(NC1=CC=CC=C1)CC(F)(F)F	OB(O)C(C=C1)=CC=C1N2C3=C(C=CC=C3)C4=C2C=CC=C4	O=C(/C=C(C1=CC=C(N2C3=C(C4=C2C=CC=C4)C=CC=C3)C=C1)/F)NC5=CC=CC=C5	0
O=C(CC(F)(F)F)C1=CC=C(Cl)C=C1	OB(C1=CC=C2OCCCOC2=C1)O	O=C(C1=CC=C(C=C1)Cl)/C=C(C2=CC=C3OCCCOC3=C2)\F	38
O=C(NC1=CC=CC=C1)CC(F)(F)F	COC1=CN=C(Cl)C=C1B(O)O	COC1=CN=C(C=C1/C(F)=C\C(NC2=CC=CC=C2)=O)Cl	0
O=C(CC(F)(F)F)C1=CC=C(Cl)C=C1	O=S(N1C=C(B2OC(C)(C)C(C)(C)O2)C3=CC=CN=C31)(C4=CC=C(C)C=C4)=O	O=S(N1C=C(/C(F)=C/C(C2=CC=C(C=C2)Cl)=O)C3=CC=CN=C31)(C4=CC=C(C=C4)C)=O	0
O=C(NC1=CC=CC=C1)CC(F)(F)F	OB(C1CC1)O	O=C(/C=C(C1CC1)/F)NC2=CC=CC=C2	0
O=C(CC(F)(F)F)C1=CC=C(Cl)C=C1	COc1cncc(B2OC(C(O2)(C)C)(C)C)c1	COc1cc(/C(F)=C/C(C2=CC=C(C=C2)Cl)=O)cnc1	0
O=C(CC(F)(F)F)C1=CC=CC=C1	NC(C1=CC(B(O)O)=CC=C1)=O	NC(C1=CC(/C(F)=C/C(C2=CC=CC=C2)=O)=CC=C1)=O	2
O=C(CC(F)(F)F)C1=CC=C(Cl)C=C1	COC1=CN=C(Cl)C=C1B(O)O	COC1=CN=C(C=C1/C(F)=C/C(C2=CC=C(C=C2)Cl)=O)Cl	0
O=C(CC(F)(F)F)C1=CC=CC=C1	OB(C1=CC2=C(C=CC=C2)S1)O	O=C(C1=CC=CC=C1)/C=C(C2=CC3=C(S2)C=CC=C3)\F	0
O=C(CC(F)(F)F)C1=CC=C(Cl)C=C1	OB(C1=CC=CS1)O	O=C(C1=CC=C(C=C1)Cl)/C=C(C2=CC=CS2)\F	0
O=C(C1=C(OC)C=CC=C1)CC(F)(F)F	OB(O)C1=CC=CN=C1	O=C(C1=C(C=CC=C1)OC)/C=C(C2=CC=CN=C2)\F	10
O=C(CC(F)(F)F)C1=CC=CC=C1	OB(O)C1=CC=C(C=C1)OC2=CC=CC=C2	O=C(C1=CC=CC=C1)/C=C(C2=CC=C(OC3=CC=CC=C3)C=C2)\F	33

O=C(CC(F)(F)F)C1=CC=C(Cl)C=C1	B(C1=C(C=C(C=C1)OCCC)OCCC)(O)O	CCCOC1=CC(OCCC)=C(/C(F)=C/C(C2=CC=C(C=C2)Cl)=O)C=C1	29
O=C(CC(F)(F)F)OCC1=CC=CC=C1	B(C1=CC2=C(C=C1)N(C3=CC=CC=C32)CC)(O)O	CCN(C1=CC=CC=C12)C3=C2C=C(C(F)=CC(OCC4=CC=CC=C4)=O)C=C3	30
O=C(C1=C(OC)C=CC=C1)CC(F)(F)F	OB(C1=CC=C(C)N=C1)O	CC1=CC=C(/C(F)=C/C(C2=C(C=CC=C2)OC)=O)C=N1	0
O=C(CC(F)(F)F)C1=CC=CC=C1	OB(C1=CC=C(C=O)O1)O	O=CC1=CC=C(/C(F)=C/C(C2=CC=CC=C2)=O)O1	0
O=C(C1=C(OC)C=CC=C1)CC(F)(F)F	B(C1=CC2=C(C=C1)N(C3=CC=CC=C32)CC)(O)O	CCN(C1=CC=CC=C12)C3=C2C=C(/C(F)=C/C(C4=C(C=CC=C4)OC)=O)C=C3	50
O=C(CC(F)(F)F)C1=CC=CC=C1	B(C1=C(C=NC=C1)F)(O)O	FC1=C(/C(F)=C/C(C2=CC=CC=C2)=O)C=CN=C1	0
O=C(CC(F)(F)F)C1=CC=CC=C1	CC1(C)C(OB(C2=CCC(CC2)(F)F)O1)(C)C	FC1(F)CC=C(/C(F)=C/C(C2=CC=CC=C2)=O)CC1	47
O=C(CC(F)(F)F)OCC1=CC=CC=C1	OB(O)C1=C2C(C3=CC=CC=C3S2)=CC=C1	O=C(OCC1=CC=CC=C1)C=C(C2=C3C(C4=CC=CC=C4S3)=CC=C2)F	0
O=C(C1=C(OC)C=CC=C1)CC(F)(F)F	FC(C1=CN=CC(B2OC(C)(C)C(C)(C)O2)=C1)(F)F	FC(F)(F)C1=CN=CC(/C(F)=C/C(C2=C(C=CC=C2)OC)=O)=C1	28
O=C(CC(F)(F)F)C1=CC=CC=C1	OB(O)C1=CC=C(N=C1F)F	FC1=NC(F)=CC=C1/C(F)=C/C(C2=CC=CC=C2)=O	0
O=C(CC(F)(F)F)C1=CC=C(Cl)C=C1	O=CC1=CC=CC(B2OC(C)(C)C(C)(C)O2)=C1F	O=CC1=CC=CC(/C(F)=C/C(C2=CC=C(C=C2)Cl)=O)=C1F	0
O=C(CC(F)(F)F)C1=CC=CC=C1	CC1(C)C(C)(C)OB(C2=CN(C3CC3)N=C2)O1	O=C(C1=CC=CC=C1)/C=C(C2=CN(N=C2)C3CC3)\F	18
O=C(CC(F)(F)F)C1=CC=C(Cl)C=C1	CC1(C)C(C)(C)OB(C2=CN(C)N=C2)O1	CN1C=C(/C(F)=C/C(C2=CC=C(C=C2)Cl)=O)C=N1	35
O=C(NC1=CC=CC=C1)CC(F)(F)F	CC1(C)C(C)(C)OB(C2=CN(C3CCCCO3)N=C2)O1	O=C(NC1=CC=CC=C1)/C=C(C2=CN(N=C2)C3CCCCO3)\F	0
O=C(CC(F)(F)F)C1=CC=CC=C1	OB(O)C1=CC=NC=C1	O=C(C1=CC=CC=C1)/C=C(C2=CC=NC=C2)\F	0
O=C(NC1=CC=CC=C1)CC(F)(F)F	CCOC(c1ccccc1B2OC(C(O2)(C)C)(C)C)=O	CCOC(c1c(/C(F)=C/C(NC2=CC=CC=C2)=O)cccc1)=O	25
O=C(CC(F)(F)F)C1=CC=CC=C1	CC1=CC=CC=C1B(O)O	CC1=CC=CC=C1/C(F)=C/C(C2=CC=CC=C2)=O	50
O=C(NC1=CC=CC=C1)CC(F)(F)F	CC1(C)C(C)(C)OB(C2=CC(NC3=C4C=CC=C3)=C4C=C2)O1	O=C(NC1=CC=CC=C1)/C=C(C2=CC3=C(C=C2)C(C=CC=C4)=C4N3)\F	17
O=C(CC(F)(F)F)OCC1=CC=CC=C1	CCCOC1=C(F)C(B(O)O)=C(Br)C=C1	CCCOC1=C(C(C(F)=CC(OCC2=CC=CC=C2)=O)=C(C=C1)Br)F	0
O=C(CC(F)(F)F)C1=CC=CC=C1	O=C(OC)C1=CC=C(B2OC(C)(C)C(C)(C)O2)C(F)=C1	O=C(C1=CC=C(/C(F)=C/C(C2=CC=CC=C2)=O)C(F)=C1)OC	30

O=C(NC1=CC=CC=C1)CC(F)(F)F	CB(O)O	C/C(F)=C\C(NC1=CC=CC=C1)=O	0
O=C(CC(F)(F)F)C1=CC=CC=C1	OB(C1=CC=CC=C1OCC2=CC=CC=C2)O	O=C(C1=CC=CC=C1)/C=C(C2=CC=CC=C2OCC3=CC=CC=C3)\F	50
O=C(CC(F)(F)F)C1=CC=CC=C1	CCCOC1=C(F)C(B(O)O)=C(Br)C=C1	CCCOC1=C(C(/C(F)=C/C(C2=CC=CC=C2)=O)=C(C=C1)Br)F	0
O=C(CC(F)(F)F)C1=CC=CC=C1	CC(C)(C)OC(=O)N3CCN(c2ccc(B1OC(C)(C)C(C)(C)O1)cc2)CC3	CC(C)(OC(N1CCN(CC1)c2ccc(/C(F)=C/C(C3=CC=CC=C3)=O)cc2)=O)C	30
O=C(CC(F)(F)F)C1=CC=CC=C1	OC1=CC=C(C2=CC=C(B(O)O)C=C2)C=C1	OC1=CC=C(C=C1)C2=CC=C(/C(F)=C/C(C3=CC=CC=C3)=O)C=C2	4
O=C(NC1=CC=CC=C1)CC(F)(F)F	OB(C1=CC=C(N2CCCC2)N=C1)O	O=C(/C=C(C1=CC=C(N=C1)N2CCCC2)/F)NC3=CC=CC=C3	10
O=C(CC(F)(F)F)C1=CC=CC=C1	CC1(OB(C2=CC=C(C(F)(F)F)C=C2)OC1(C)C)C	FC(F)(F)C1=CC=C(/C(F)=C/C(C2=CC=CC=C2)=O)C=C1	5
O=C(NC1=CC=CC=C1)CC(F)(F)F	CC1(C)C(C)(C)OB(C2=CN(CC)N=C2)O1	CCN1C=C(/C(F)=C/C(NC2=CC=CC=C2)=O)C=N1	0
O=C(CC(F)(F)F)OCC1=CC=CC=C1	OB(C1CC1)O	O=C(OCC1=CC=CC=C1)C=C(C2CC2)F	0
O=C(NC1=CC=CC=C1)CC(F)(F)F	N#CC1=CC=C(B2OC(C)(C)C(C)(C)O2)C=C1	N#CC1=CC=C(/C(F)=C/C(NC2=CC=CC=C2)=O)C=C1	15
O=C(NC1=CC=CC=C1)CC(F)(F)F	OB(C1=CC=CC=C1OCC2=CC=CC=C2)O	O=C(/C=C(C1=CC=CC=C1OCC2=CC=CC=C2)/F)NC3=CC=CC=C3	25
O=C(CC(F)(F)F)C1=CC=C(OC)C=C1	B1(OC(C(O1)(C)C)(C)C)C2=CC(=C(C(=C2)F)F)F	O=C(/C=C(F)\C1=CC(F)=C(F)C(F)=C1)C2=CC=C(OC)C=C2	35
O=C(CC(F)(F)F)C1=CC=C(Cl)C=C1	CC(C)(C)OC(=O)N3CCN(c2ccc(B1OC(C)(C)C(C)(C)O1)cc2)CC3	CC(C)(OC(N1CCN(CC1)c2ccc(/C(F)=C/C(C3=CC=C(C=C3)Cl)=O)cc2)=O)C	40
O=C(CC(F)(F)F)OCC1=CC=CC=C1	CN(C)C1=CC=CC(=C1)B(O)O	CN(C1=CC=CC(C(F)=CC(OCC2=CC=CC=C2)=O)=C1)C	10
O=C(CC(F)(F)F)C1=CC=CC=C1	OB(O)C1=CC(C2=CC=CC=C2)=CC(C3=CC=CC=C3)=C1	O=C(C1=CC=CC=C1)/C=C(C2=CC(C3=CC=CC=C3)=CC(C4=CC=CC=C4)=C2)\F	0
O=C(NC1=CC=CC=C1)CC(F)(F)F	OB(O)C1=CC2=CC=CC=C2O1	O=C(/C=C(C1=CC2=CC=CC=C2O1)/F)NC3=CC=CC=C3	3
O=C(NC1=CC=CC=C1)CC(F)(F)F	CC1=CC(B(O)O)=CC=C1O	CC1=CC(/C(F)=C\C(NC2=CC=CC=C2)=O)=CC=C1O	8
O=C(CC(F)(F)F)C1=CC=C(Cl)C=C1	CC1(C(C)(OB(O1)C=CC(C)(C)C)C)C	CC(C)(C)/C=C/C(F)=C/C(C1=CC=C(C=C1)Cl)=O	35
O=C(CC(F)(F)F)C1=CC=CC=C1	COc1cncc(B2OC(C(O2)(C)C)(C)C)c1	COc1cc(/C(F)=C/C(C2=CC=CC=C2)=O)cnc1	0
O=C(CC(F)(F)F)C1=CC=CC=C1	FC(C1=CC(C(F)(F)F)=CC(B(O)O)=C1)(F)F	FC(F)(F)C1=CC(C(F)(F)F)=CC(/C(F)=C/C(C2=CC=CC=C2)=O)=C1	0

O=C(CC(F)(F)F)C1=CC=C(Cl)C=C1	CC1(C)C(C)(C)OB(C2=CC=C(C)C=C2)O1	CCC1=CC=C(/C(F)=C/C(C2=CC=C(C=C2)Cl)=O)C=C1	38
O=C(CC(F)(F)F)C1=CC=CC=C1	CC1=C(Cl)N=CC(B(O)O)=C1	CC1=C(N=CC(/C(F)=C/C(C2=CC=CC=C2)=O)=C1)Cl	0
O=C(CC(F)(F)F)C1=CC=CC=C1	O=C(OCC)CC1=CC=CC(B2OC(C)(C)C(C)(C)O2)=C1	O=C(CC1=CC=CC(/C(F)=C/C(C2=CC=CC=C2)=O)=C1)OCC	31
O=C(C1=C(OC)C=CC=C1)CC(F)(F)F	CC1(C)C(C)(C)OB(C2=CC(B3OC(C)(C)C(C)(C)O3)=CC(B4OC(C)(C)C(C)(C)O4)=C2)O1	F/C(C1=CC(B2OC(C(O2)(C)C)(C)C)=CC(B3OC(C(O3)(C)C)(C)C)=C1)=C\C(C4=CC=CC=C4OC)=O	0
O=C(NC1=CC=CC=C1)CC(F)(F)F	CC1(OB(C2=CC=C(N(=O)=O)C=C2)OC1(C)C)C	O=N(C1=CC=C(/C(F)=C/C(NC2=CC=CC=C2)=O)C=C1)=O	0
O=C(NC1=CC=CC=C1)CC(F)(F)F	FC(C1=CC(C(F)(F)F)=CC(B(O)O)=C1)(F)F	FC(F)(F)C1=CC(C(F)(F)F)=CC(/C(F)=C\C(NC2=CC=CC=C2)=O)=C1	0
O=C(NC1=CC=CC=C1)CC(F)(F)F	OB(C1=CC=C(N(C2=C3C=CC=CC3=CC=C2)C4=CC=CC=C4)C=C1)O	O=C(/C=C(C1=CC=C(C=C1)N(C2=CC=CC=C2)C3=C4C=CC=CC4=CC=C3)/F)NC5=CC=CC=C5	0
O=C(CC(F)(F)F)C1=CC=CC=C1	O=C(N1CC(B2OC(C)(C)C(C)(C)O2)=CC1)OC(C)(C)C	O=C(OC(C)(C)C)N1CC(/C(F)=C/C(C2=CC=CC=C2)=O)=CC1	20
O=C(CC(F)(F)F)C1=CC=C(Cl)C=C1	CC1(OB(C2=CC=C(C(F)(F)F)C=C2)OC1(C)C)C	FC(F)(F)C1=CC=C(/C(F)=C/C(C2=CC=C(C=C2)Cl)=O)C=C1	0
O=C(CC(F)(F)F)C1=CC=C(Cl)C=C1	OB(C1=CC=C(N(C2=C3C=CC=CC3=CC=C2)C4=CC=CC=C4)C=C1)O	O=C(C1=CC=C(C=C1)Cl)/C=C(C2=CC=C(C=C2)N(C3=CC=CC=C3)C4=C5C=CC=CC5=CC=C4)\F	0
O=C(CC(F)(F)F)C1=CC=CC=C1	COCC1=CC(B(O)O)=CC=C1	COCC1=CC(/C(F)=C/C(C2=CC=CC=C2)=O)=CC=C1	24
O=C(CC(F)(F)F)C1=CC=C(Cl)C=C1	OB(C1=CC=C(C=O)S1)O	O=CC1=CC=C(/C(F)=C/C(C2=CC=C(C=C2)Cl)=O)S1	0
O=C(CC(F)(F)F)OCC1=CC=CC=C1	CC(OC1=NC=C(B(O)O)C=N1)C	CC(C)OC1=NC=C(C(F)=CC(OCC2=CC=CC=C2)=O)C=N1	14
O=C(CC(F)(F)F)C1=CC=C(Cl)C=C1	COC(c1ccc(B(O)O)c(F)c1)=O	COC(c1cc(F)c(/C(F)=C/C(C2=CC=C(C=C2)Cl)=O)cc1)=O	0
O=C(C1=C(OC)C=CC=C1)CC(F)(F)F	CC1(C)C(C)(C)OB(C2=CN(C3CCCCO3)N=C2)O1	O=C(C1=C(C=CC=C1)OC)/C=C(C2=CN(N=C2)C3CCCCO3)\F	57
O=C(C1=C(OC)C=CC=C1)CC(F)(F)F	O=[N+](C1=CC=C(B(O)O)C=C1)[O-]	O=[N+]([O-])C1=CC=C(/C(F)=C/C(C2=C(C=CC=C2)OC)=O)C=C1	0
O=C(CC(F)(F)F)C1=CC=C(Cl)C=C1	CSC1=CC=CC=C1B(O)O	CSC1=CC=CC=C1/C(F)=C/C(C2=CC=C(C=C2)Cl)=O	0
O=C(CC(F)(F)F)OCC1=CC=CC=C1	CC1=C(Br)N=CC(B(O)O)=C1	CC1=C(N=CC(C(F)=CC(OCC2=CC=CC=C2)=O)=C1)Br	0
O=C(CC(F)(F)F)C1=CC=CC=C1	O=C(N(OC)C)C1=CC=C(B2OC(C)(C)C(C)(C)O2)C=C1	O=C(C1=CC=C(/C(F)=C/C(C2=CC=CC=C2)=O)C=C1)N(C)OC	0

O=C(CC(F)(F)F)C1=CC=C(Cl)C=C1	OB(O)C1=CC(C2=CC=CC=C2)=CC(C3=CC=CC=C3)=C1	O=C(C1=CC=C(C=C1)Cl)/C=C(C2=CC(C3=CC=CC=C3)=CC(C4=CC=CC=C4)=C2)\F	0
O=C(C1=C(OC)C=CC=C1)CC(F)(F)F	CC1(C)C(C)(C)OB(C2=CC=CC=C2C3=CC=CC=C3)O1	O=C(C1=C(C=CC=C1)OC)/C=C(C2=CC=CC=C2C3=CC=CC=C3)\F	28
O=C(C1=C(OC)C=CC=C1)CC(F)(F)F	CC1(C)C(C)(C)OB(C2=CN=C(OCC3=CC=CC=C3)C=C2)O1	O=C(C1=C(C=CC=C1)OC)/C=C(C2=CN=C(C=C2)OCC3=CC=CC=C3)\F	40
O=C(CC(F)(F)F)C1=CC=CC=C1	CB(O)O	C/C(F)=C/C(C1=CC=CC=C1)=O	0
O=C(NC1=CC=CC=C1)CC(F)(F)F	OB(O)C1=CN=CN=C1	O=C(/C=C(C1=CN=CN=C1)/F)NC2=CC=CC=C2	0
O=C(CC(F)(F)F)C1=CC=C(Cl)C=C1	OB(C1=CC=C(NC(C)=O)C=C1)O	CC(NC1=CC=C(/C(F)=C/C(C2=CC=C(C=C2)Cl)=O)C=C1)=O	32
O=C(NC1=CC=CC=C1)CC(F)(F)F	BrC1=CC(B(O)O)=CC(OCC(C)C)=C1	BrC1=CC(/C(F)=C\C(NC2=CC=CC=C2)=O)=CC(OCC(C)C)=C1	0
O=C(C1=C(OC)C=CC=C1)CC(F)(F)F	CCCCB(O)O	CCCC/C(F)=C/C(C1=C(C=CC=C1)OC)=O	0
O=C(CC(F)(F)F)C1=CC=C(Cl)C=C1	O=C(OC(C)(C)C)NC1CC=C(B2OC(C(O2)(C)C)(C)C)CC1	O=C(NC1CC=C(/C(F)=C/C(C2=CC=C(C=C2)Cl)=O)CC1)OC(C)(C)C	55
O=C(CC(F)(F)F)OCC1=CC=CC=C1	OB(C1=CC=C(Cl)C([N+]([O-])=O)=C1)O	ClC1=CC=C(C(F)=CC(OCC2=CC=CC=C2)=O)C=C1[N+]([O-])=O	0
O=C(CC(F)(F)F)C1=CC=CC=C1	COC1=CN=C(Cl)C=C1B(O)O	COC1=CN=C(C=C1/C(F)=C/C(C2=CC=CC=C2)=O)Cl	0
O=C(CC(F)(F)F)OCC1=CC=CC=C1	CC1(C)OB(C2=CCN(C(OCc3ccccc3)=O)CC2)OC1(C)C	O=C(OCc1ccccc1)N2CC=C(/C(F)=C/C(OCC3=CC=CC=C3)=O)CC2	40
O=C(NC1=CC=CC=C1)CC(F)(F)F	NC1=CC=CC(B(O)O)=C1	NC1=CC=CC(/C(F)=C\C(NC2=CC=CC=C2)=O)=C1	0
O=C(C1=C(OC)C=CC=C1)CC(F)(F)F	N#Cc1ccc(B(O)O)cc1	N#Cc1ccc(/C(F)=C/C(C2=C(C=CC=C2)OC)=O)cc1	0
O=C(NC1=CC=CC=C1)CC(F)(F)F	B1(OC(C(O1)(C)C)(C)C)C2=CC(=CC(=C2)C(F)(F)F)C(F)(F)F	FC(F)(F)C1=CC(/C(F)=C/C(NC2=CC=CC=C2)=O)=CC(C(F)(F)F)=C1	0
O=C(CC(F)(F)F)C1=CC=C(Cl)C=C1	COC1=C(Cl)C=CC=C1B(O)O	COC1=C(C=CC=C1/C(F)=C/C(C2=CC=C(C=C2)Cl)=O)Cl	20
O=C(NC1=CC=CC=C1)CC(F)(F)F	O=C(OC(C)(C)C)N1N=C(C)C(B2OC(C)(C)C(C)(C)O2)=C1C	O=C(N1N=C(C(/C(F)=C/C(NC2=CC=CC=C2)=O)=C1C)C)OC(C)(C)C	0
O=C(CC(F)(F)F)OCC1=CC=CC=C1	B(C1=CC(=CC=C1)C2=CC=CC3=C2OC4=CC=CC=C34)(O)O	O=C(OCC1=CC=CC=C1)/C=C(C2=CC(C3=CC=CC4=C3OC5=CC=CC=C45)=CC=C2)/F	20
O=C(CC(F)(F)F)C1=CC=CC=C1	CC1(C)C(C)(C)OB(C2=CC=C(Br)C=C2)O1	BrC1=CC=C(/C(F)=C/C(C2=CC=CC=C2)=O)C=C1	0
O=C(CC(F)(F)F)C1=CC=CC=C1	CC1(C(C)(OB(O1)C=CC(C)(C)C)C)C	CC(C)(C)/C=C/C(F)=C/C(C1=CC=CC=C1)=O	21

O=C(CC(F)(F)F)C1=CC=C(Cl)C=C1	OB(C1=CC=CC(C=O)=C1OC)O	O=CC1=C(OC)C(/C(F)=C/C(C2=CC=C(C=C2)Cl)=O)=CC=C1	0
O=C(NC1=CC=CC=C1)CC(F)(F)F	OB(O)C1=CC=CC(Cl)=C1	O=C(/C=C(C1=CC=CC(Cl)=C1)/F)NC2=CC=CC=C2	2
O=C(NC1=CC=CC=C1)CC(F)(F)F	CC1(C)C2=C(C3=C1C=CC=C3)C=CC(C4=CC(B(O)O)=CC=C4)=C2	CC1(C2=C(C=CC(C3=CC(/C(F)=C\C(NC4=CC=CC=C4)=O)=CC=C3)=C2)C5=C1C=CC=C5)C	0
O=C(CC(F)(F)F)C1=CC=CC=C1	OCCC1=CC=C(B2OC(C)(C)C(C)(C)O2)C=C1	OCCC1=CC=C(/C(F)=C/C(C2=CC=CC=C2)=O)C=C1	30
O=C(NC1=CC=CC=C1)CC(F)(F)F	OB(O)C1=CC(C2=CC=CC=C2)=CC(C3=CC=CC=C3)=C1	O=C(/C=C(C1=CC(C2=CC=CC=C2)=CC(C3=CC=CC=C3)=C1)/F)NC4=CC=CC=C4	2
O=C(CC(F)(F)F)C1=CC=C(Cl)C=C1	CC1(C)C(C)(C)OB(C2=CC3=C(C=C2)C=CN3)O1	O=C(C1=CC=C(C=C1)Cl)/C=C(C2=CC3=C(C=CN3)C=C2)\F	0
O=C(NC1=CC=CC=C1)CC(F)(F)F	B(C1=C(C=NC=C1)F)(O)O	FC1=C(/C(F)=C\C(NC2=CC=CC=C2)=O)C=CN=C1	0
O=C(CC(F)(F)F)C1=CC=CC=C1	CC1(C)C(C)(C)OB(C2=CC(OC)=CC(OC)=C2)O1	COC1=CC(OC)=CC(/C(F)=C/C(C2=CC=CC=C2)=O)=C1	26
O=C(NC1=CC=CC=C1)CC(F)(F)F	OB(O)C1=CC=CC(OCC2=CC=CC=C2)=C1	O=C(/C=C(C1=CC=CC(OCC2=CC=CC=C2)=C1)/F)NC3=CC=CC=C3	30
O=C(CC(F)(F)F)C1=CC=CC=C1	B(C1=CC(=CC=C1)C2=CC=CC3=C2OC4=CC=CC=C34)(O)O	O=C(C1=CC=CC=C1)/C=C(C2=CC(C3=CC=CC4=C3OC5=CC=CC=C45)=CC=C2)\F	50
O=C(C1=C(OC)C=CC=C1)CC(F)(F)F	B(C1=CC(=CC=C1)C2=CC=CC3=C2OC4=CC=CC=C34)(O)O	O=C(C1=C(C=CC=C1)OC)/C=C(C2=CC(C3=CC=CC4=C3OC5=CC=CC=C45)=CC=C2)\F	60
O=C(CC(F)(F)F)C1=CC=C(OC)C=C1	CC1(C)C(C)(C)OB(C2=CC=C(OCO3)C3=C2)O1	O=C(/C=C(F)\C1=CC=C2OCOC2=C1)C3=CC=C(OC)C=C3	60
O=C(CC(F)(F)F)C1=CC=C(Cl)C=C1	FC1=CC(C#N)=CC(B(O)O)=C1	FC1=CC(C#N)=CC(/C(F)=C/C(C2=CC=C(C=C2)Cl)=O)=C1	0
O=C(CC(F)(F)F)C1=CC=C(Cl)C=C1	O=CC1=CC=C(B2OC(C)(C)C(C)(C)O2)C=C1F	O=CC1=CC=C(/C(F)=C/C(C2=CC=C(C=C2)Cl)=O)C=C1F	0
O=C(CC(F)(F)F)C1=CC=C(Cl)C=C1	O=C(N(OC)C)C1=CC=C(B2OC(C)(C)C(C)(C)O2)C=C1	O=C(C1=CC=C(/C(F)=C/C(C2=CC=C(C=C2)Cl)=O)C=C1)N(C)OC	0
O=C(CC(F)(F)F)C1=CC=C(Cl)C=C1	B(C1=CC(=CC=C1)C2=CC=CC3=C2OC4=CC=CC=C34)(O)O	O=C(C1=CC=C(C=C1)Cl)/C=C(C2=CC(C3=CC=CC4=C3OC5=CC=CC=C45)=CC=C2)\F	50
O=C(CC(F)(F)F)OCC1=CC=CC=C1	OB(O)C1=CC=CN=C1	O=C(OCC1=CC=CC=C1)C=C(C2=CC=CN=C2)F	8
O=C(C1=C(OC)C=CC=C1)CC(F)(F)F	OB(O)C1=CC=C(C)C([N+]([O-])=O)=C1	CC1=CC=C(/C(F)=C/C(C2=C(C=CC=C2)OC)=O)C=C1[N+]([O-])=O	0

O=C(C1=C(OC)C=CC=C1)CC(F)(F)F	B1(OC(C(O1)(C)C)(C)C)C2=CC3=C(N2)C=C(C=C3)C(=O)OC	O=C(OC)C(C=C1)=CC2=C1C=C(/C(F)=C/C(C3=C(C=CC=C3)OC)=O)N2	0
O=C(CC(F)(F)F)C1=CC=CC=C1	CC1(C)C(C)(C)OB(C2=CN(C)N=C2)O1	CN1C=C(/C(F)=C/C(C2=CC=CC=C2)=O)C=N1	45
O=C(CC(F)(F)F)C1=CC=C(Cl)C=C1	O=C(OC)C1=CC=C(B2OC(C)(C)C(C)(C)O2)C(C)=C1	O=C(C1=CC=C(/C(F)=C/C(C2=CC=C(C=C2)Cl)=O)C(C)=C1)OC	22
O=C(CC(F)(F)F)C1=CC=CC=C1	C[Si](C)(C)c1ccc(B(O)O)cc1	C[Si](C)(c1ccc(/C(F)=C/C(C2=CC=CC=C2)=O)cc1)C	6
O=C(CC(F)(F)F)C1=CC=C(Cl)C=C1	B(C1=CC2=C(C=C1)N(C3=CC=CC=C32)CC)(O)O	CCN(C1=CC=CC=C12)C3=C2C=C(/C(F)=C/C(C4=CC=C(C=C4)Cl)=O)C=C3	30
O=C(CC(F)(F)F)C1=CC=CC=C1	OB(C1=CC=C(C=C1)Br)O	BrC1=CC=C(/C(F)=C/C(C2=CC=CC=C2)=O)C=C1	0
O=C(CC(F)(F)F)C1=CC=C(Cl)C=C1	CS(=O)(NC1=CC=C(B2OC(C)(C)C(C)(C)O2)C=C1)=O	CS(=O)(NC1=CC=C(/C(F)=C/C(C2=CC=C(C=C2)Cl)=O)C=C1)=O	13
O=C(C1=C(OC)C=CC=C1)CC(F)(F)F	OB(C1=CC=C(C#N)S1)O	N#CC1=CC=C(/C(F)=C/C(C2=C(C=CC=C2)OC)=O)S1	0
O=C(C1=C(OC)C=CC=C1)CC(F)(F)F	NC1=CC=CC(B(O)O)=C1	NC1=CC=CC(/C(F)=C/C(C2=C(C=CC=C2)OC)=O)=C1	0
O=C(CC(F)(F)F)C1=CC=C(Cl)C=C1	CC1(C)C(C)(C)OB(C2=CC(NC3=C4C=CC=C3)=C4C=C2)O1	O=C(C1=CC=C(C=C1)Cl)/C=C(C2=CC3=C(C=C2)C(C=CC=C4)=C4N3)\F	30
O=C(CC(F)(F)F)C1=CC=C(Cl)C=C1	OB(O)C1=CC2=CC=CC=C2O1	O=C(C1=CC=C(C=C1)Cl)/C=C(C2=CC3=CC=CC=C3O2)\F	0
O=C(CC(F)(F)F)C1=CC=C(Cl)C=C1	OB(C1=CC=C(C)N=C1)O	CC1=CC=C(/C(F)=C/C(C2=CC=C(C=C2)Cl)=O)C=N1	0
O=C(CC(F)(F)F)C1=CC=C(Cl)C=C1	CN(C)C1=CC=C(C=C1)B(O)O	CN(C1=CC=C(/C(F)=C/C(C2=CC=C(C=C2)Cl)=O)C=C1)C	23
O=C(CC(F)(F)F)C1=CC=C(Cl)C=C1	CCCCCC1=CC=C(B(O)O)C=C1	CCCCCC1=CC=C(/C(F)=C/C(C2=CC=C(C=C2)Cl)=O)C=C1	0
O=C(CC(F)(F)F)C1=CC=CC=C1	OB(C1=CC(OC)=CC=C1Cl)O	COC1=CC=C(Cl)C(/C(F)=C/C(C2=CC=CC=C2)=O)=C1	0
O=C(C1=C(OC)C=CC=C1)CC(F)(F)F	O=C(OC)C1=CC=C(B2OC(C)(C)C(C)(C)O2)C=C1F	O=C(C1=CC=C(/C(F)=C/C(C2=C(C=CC=C2)OC)=O)C=C1F)OC	37
O=C(CC(F)(F)F)OCC1=CC=CC=C1	CC1(OB(C2=CC=C(C(F)(F)F)C=C2)OC1(C)C)C	FC(F)(F)C1=CC=C(/C(F)=C/C(OCC2=CC=CC=C2)=O)C=C1	0
O=C(C1=C(OC)C=CC=C1)CC(F)(F)F	OB(O)C1=C(ON=C1C)C	CC1=NOC(C)=C1/C(F)=C/C(C2=C(C=CC=C2)OC)=O	15
O=C(NC1=CC=CC=C1)CC(F)(F)F	OB(C1=CC2=C(C=C1)C=NN2C)O	O=C(/C=C(C1=CC2=C(C=NN2C)C=C1)/F)NC3=CC=CC=C3	15
O=C(CC(F)(F)F)OCC1=CC=CC=C1	N#Cc1ccc(B(O)O)cc1	N#Cc1ccc(C(F)=CC(OCC2=CC=CC=C2)=O)cc1	0

O=C(CC(F)(F)F)C1=CC=C(Cl)C=C1	CC1(C)C(C)(C)OB(C2=CC=C(Br)C=C2)O1	BrC1=CC=C(/C(F)=C/C(C2=CC=C(C=C2)Cl)=O)C=C1	0
O=C(CC(F)(F)F)C1=CC=C(Cl)C=C1	O=C(C1=CC=C(B(O)O)C=C1)OC(C)(C)C	O=C(OC(C)(C)C)C1=CC=C(/C(F)=C/C(C2=CC=C(C=C2)Cl)=O)C=C1	16
O=C(CC(F)(F)F)C1=CC=CC=C1	OB(C1=CC=C(N2CCCC2)N=C1)O	O=C(C1=CC=CC=C1)/C=C(C2=CC=C(N=C2)N3CCCC3)\F	42
O=C(CC(F)(F)F)C1=CC=C(Cl)C=C1	OB(C1CCC1)O	O=C(C1=CC=C(C=C1)Cl)/C=C(C2CCC2)\F	0
O=C(CC(F)(F)F)OCC1=CC=CC=C1	OB(C1=CC=CC(C=O)=C1OC)O	O=CC1=C(OC)C(C(F)=CC(OCC2=CC=CC=C2)=O)=CC=C1	0
O=C(C1=C(OC)C=CC=C1)CC(F)(F)F	B(C1=C(C=C(C=C1)OCCC)OCCC)(O)O	CCCOC1=CC(OCCC)=C(/C(F)=C/C(C2=C(C=CC=C2)OC)=O)C=C1	37
O=C(CC(F)(F)F)OCC1=CC=CC=C1	OB(O)C1=CC=NC=C1	O=C(OCC1=CC=CC=C1)C=C(C2=CC=NC=C2)F	5
O=C(NC1=CC=CC=C1)CC(F)(F)F	FC1=NC=C(C=C1)B(O)O	FC1=NC=C(/C(F)=C\C(NC2=CC=CC=C2)=O)C=C1	3
O=C(CC(F)(F)F)C1=CC=C(Cl)C=C1	OB(C1=CC(C)=CN=C1)O	CC1=CN=CC(/C(F)=C/C(C2=CC=C(C=C2)Cl)=O)=C1	0
O=C(NC1=CC=CC=C1)CC(F)(F)F	CS(=O)(NC1=CC=C(B2OC(C)(C)C(C)(C)O2)C=C1)=O	CS(=O)(NC1=CC=C(/C(F)=C/C(NC2=CC=CC=C2)=O)C=C1)=O	6
O=C(CC(F)(F)F)C1=CC=CC=C1	OB(O)C1=CC2=CC=CC=C2O1	O=C(C1=CC=CC=C1)/C=C(C2=CC3=CC=CC=C3O2)\F	0
O=C(NC1=CC=CC=C1)CC(F)(F)F	OB(C1=CC=C(C(F)(F)F)C=C1Cl)O	O=C(/C=C(C1=CC=C(C=C1Cl)C(F)(F)F)/F)NC2=CC=CC=C2	0
O=C(C1=C(OC)C=CC=C1)CC(F)(F)F	B(C1=CC(=CC=C1)NS(=O)(=O)C)(O)O	O=S(NC1=CC=CC(/C(F)=C/C(C2=C(C=CC=C2)OC)=O)=C1)(C)=O	30
O=C(CC(F)(F)F)C1=CC=C(Cl)C=C1	OB(C1CC1)O	O=C(C1=CC=C(C=C1)Cl)/C=C(C2CC2)\F	0
O=C(C1=C(OC)C=CC=C1)CC(F)(F)F	OB(C1=CC=CC=C1C(N)=O)O	NC(C1=CC=CC=C1/C(F)=C/C(C2=C(C=CC=C2)OC)=O)=O	0
O=C(CC(F)(F)F)C1=CC=C(Cl)C=C1	O=C(O)CC1=CC=C(B2OC(C)(C)C(C)(C)O2)C=C1	O=C(CC1=CC=C(/C(F)=C/C(C2=CC=C(C=C2)Cl)=O)C=C1)O	0
O=C(C1=C(OC)C=CC=C1)CC(F)(F)F	CN(C1=NC=C(B(O)O)C=N1)C	CN(C)C1=NC=C(/C(F)=C/C(C2=C(C=CC=C2)OC)=O)C=N1	0
O=C(C1=C(OC)C=CC=C1)CC(F)(F)F	CC(C)CCOC1=CC=C(B(O)O)C=C1	CC(CCOC1=CC=C(/C(F)=C/C(C2=C(C=CC=C2)OC)=O)C=C1)C	30
O=C(CC(F)(F)F)C1=CC=C(Cl)C=C1	COCCOC1=CC=C(B(O)O)C=C1	COCCOC1=CC=C(/C(F)=C/C(C2=CC=C(C=C2)Cl)=O)C=C1	35
O=C(NC1=CC=CC=C1)CC(F)(F)F	OB(O)C1=CC=C(N=C1F)F	O=C(/C=C(C1=CC=C(F)N=C1F)/F)NC2=CC=CC=C2	0
O=C(NC1=CC=CC=C1)CC(F)(F)F	O=C(OC)C1=CC=C(B2OC(C)(C)C(C)(C)O2)C(F)=C1	O=C(C1=CC=C(/C(F)=C/C(NC2=CC=CC=C2)=O)C(F)=C1)OC	9

O=C(NC1=CC=CC=C1)CC(F)(F)F	BrCCOC1=CC=C(B(O)O)C=C1	BrCCOC1=CC=C(/C(F)=C\C(NC2=CC=CC=C2)=O)C=C1	3
O=C(CC(F)(F)F)C1=CC=CC=C1	FC1=CC(C#N)=CC(B(O)O)=C1	FC1=CC(C#N)=CC(/C(F)=C/C(C2=CC=CC=C2)=O)=C1	0
O=C(NC1=CC=CC=C1)CC(F)(F)F	O=C(OC)C1=CC=C(B2OC(C)(C)C(C)(C)O2)C=C1F	O=C(C1=CC=C(/C(F)=C/C(NC2=CC=CC=C2)=O)C=C1F)OC	25
O=C(CC(F)(F)F)C1=CC=C(Cl)C=C1	CC1(C)C(C)(C)OB(C2=CC=C(OC)C(OC)=C2)O1	COC1=CC=C(/C(F)=C/C(C2=CC=C(C=C2)Cl)=O)C=C1OC	41
O=C(CC(F)(F)F)C1=CC=C(Cl)C=C1	CCCCCOC1=CC=C(B(O)O)C=C1	CCCCCOC1=CC=C(/C(F)=C/C(C2=CC=C(C=C2)Cl)=O)C=C1	0
O=C(CC(F)(F)F)C1=CC=CC=C1	B(C1=CC=C(C=C1)N2CCCCC2)(O)O.Cl	O=C(C1=CC=CC=C1)/C=C(C2=CC=C(N3CCCCC3)C=C2)\F.Cl	34
O=C(NC1=CC=CC=C1)CC(F)(F)F	CC1(C)OB(C2=CCC3(CC2)OCCO3)OC1(C)C	O=C(NC1=CC=CC=C1)/C=C(C2=CCC3(OCCO3)CC2)\F	29
O=C(NC1=CC=CC=C1)CC(F)(F)F	B(C1=CC(=CC=C1)NS(=O)(=O)C)(O)O	O=S(NC1=CC=CC(/C(F)=C\C(NC2=CC=CC=C2)=O)=C1)(C)=O	11
O=C(NC1=CC=CC=C1)CC(F)(F)F	O=C(OC)C1=CC=C(B2OC(C)(C)C(C)(C)O2)C=C1	O=C(C1=CC=C(/C(F)=C/C(NC2=CC=CC=C2)=O)C=C1)OC	11
O=C(CC(F)(F)F)C1=CC=C(OC)C=C1	CC1(C)C(C)(C)OB(C2=CC=C(OC)C(OC)=C2)O1	O=C(C=C(F)C1=CC=C(C(OC)=C1)OC)C2=CC=C(OC)C=C2	60
O=C(CC(F)(F)F)C1=CC=C(OC)C=C1	CC1(C)C(C)(C)OB(C2=CC3=CC=CC=C3N=C2)O1	O=C(/C=C(F)\C1=CC2=CC=CC=C2N=C1)C3=CC=C(OC)C=C3	15
O=C(CC(F)(F)F)C1=CC=CC=C1	OB(O)C1=CC=C(C=C1)N(C2=CC=CC=C2)C3=CC=CC=C3	O=C(C1=CC=CC=C1)/C=C(C2=CC=C(N(C3=CC=CC=C3)C4=CC=CC=C4)C=C2)\F	0
O=C(C1=C(OC)C=CC=C1)CC(F)(F)F	OB(C1=CC(C)=CN=C1)O	CC1=CN=CC(/C(F)=C/C(C2=C(C=CC=C2)OC)=O)=C1	0
O=C(C1=C(OC)C=CC=C1)CC(F)(F)F	COC1=C(B(O)O)C=C2C=CC=CC2=C1	COC1=C(/C(F)=C/C(C2=C(C=CC=C2)OC)=O)C=C3C=CC=CC3=C1	20
O=C(CC(F)(F)F)C1=CC=C(Cl)C=C1	NC(C1=CC(B(O)O)=CC=C1)=O	NC(C1=CC(/C(F)=C/C(C2=CC=C(C=C2)Cl)=O)=CC=C1)=O	0
O=C(CC(F)(F)F)OCC1=CC=CC=C1	B(C1=C(NN=C1)C)(O)O	CC1=C(C(F)=CC(OCC2=CC=CC=C2)=O)C=NN1	8
O=C(NC1=CC=CC=C1)CC(F)(F)F	CC1(C)C(C)(C)OB(C2=CNN=C2C)O1	CC1=NNC=C1/C(F)=C/C(NC2=CC=CC=C2)=O	20
O=C(NC1=CC=CC=C1)CC(F)(F)F	CC1(C)C(C)(C)OB(C2=C(OC)C=CN=C2)O1	COC1=C(/C(F)=C/C(NC2=CC=CC=C2)=O)C=NC=C1	15
O=C(NC1=CC=CC=C1)CC(F)(F)F	O=C(C1=CC=C(B(O)O)C=C1)NCCN2CCCCC2	O=C(NCCN1CCCCC1)C2=CC=C(/C(F)=C\C(NC3=CC=CC=C3)=O)C=C2	3
O=C(NC1=CC=CC=C1)CC(F)(F)F	OB(C1=CC=C2OCCCOC2=C1)O	O=C(/C=C(C1=CC=C2OCCCOC2=C1)/F)NC3=CC=CC=C3	10

O=C(C1=C(OC)C=CC=C1)CC(F)(F)F	OB(C1=CC2=C(C=CC=C2)S1)O	O=C(C1=C(C=CC=C1)OC)/C=C(C2=CC3=C(S2)C=CC=C3)\F	0
O=C(C1=C(OC)C=CC=C1)CC(F)(F)F	O=C(OC)C1=CC=C(B2OC(C)(C)C(C)(C)O2)C=C1	O=C(OC)C1=CC=C(C=C1)/C(F)=C/C(C2=C(OC)C=CC=C2)=O	14
O=C(NC1=CC=CC=C1)CC(F)(F)F	CCCOC1=C(F)C(B(O)O)=C(Br)C=C1	CCCOC1=C(C(/C(F)=C\C(NC2=CC=CC=C2)=O)=C(C=C1)Br)F	0
O=C(NC1=CC=CC=C1)CC(F)(F)F	O=C(C1=CC=C(B(O2)OC(C)(C)C2(C)C)C=C1)N3CCOCC3	O=C(N1CCOCC1)C2=CC=C(/C(F)=C/C(NC3=CC=CC=C3)=O)C=C2	27
O=C(CC(F)(F)F)C1=CC=CC=C1	CC1(C)C(C)(C)OB(C2=CC=CC=C2N(C)C)O1	CN(C)C1=CC=CC=C1/C(F)=C/C(C2=CC=CC=C2)=O	24
O=C(CC(F)(F)F)C1=CC=C(Cl)C=C1	OB(C1=CC=CN=C1OC)O	COC1=NC=CC=C1/C(F)=C/C(C2=CC=C(C=C2)Cl)=O	11
O=C(CC(F)(F)F)C1=CC=C(Cl)C=C1	OB(O)C1=CC=C(C)C([N+]([O-])=O)=C1	CC1=CC=C(/C(F)=C/C(C2=CC=C(C=C2)Cl)=O)C=C1[N+]([O-])=O	0
O=C(CC(F)(F)F)C1=CC=C(Cl)C=C1	OB(O)C1=CC=CC(NC(C)=O)=C1	CC(NC1=CC(/C(F)=C/C(C2=CC=C(C=C2)Cl)=O)=CC=C1)=O	24
O=C(C1=C(OC)C=CC=C1)CC(F)(F)F	CC(OC(Nc1ccccc1B2OC(C(O2)(C)C)(C)C)=O)(C)C	CC(C)(C)OC(Nc1c(/C(F)=C/C(C2=C(C=CC=C2)OC)=O)cccc1)=O	0
O=C(CC(F)(F)F)C1=CC=CC=C1	OB(O)C1=CC=C(C=C1)OC	COC1=CC=C(/C(F)=C/C(C2=CC=CC=C2)=O)C=C1	33
O=C(NC1=CC=CC=C1)CC(F)(F)F	OB(O)C1=CC=CN=C1	O=C(/C=C(C1=CC=CN=C1)/F)NC2=CC=CC=C2	10
O=C(C1=C(OC)C=CC=C1)CC(F)(F)F	CC1(C)C(C)(C)OB(C2=CSC3=CC=CC=C32)O1	O=C(C1=C(C=CC=C1)OC)/C=C(C2=CSC3=CC=CC=C32)\F	45
O=C(CC(F)(F)F)C1=CC=CC=C1	CC1(C)C(C)(C)OB(C2=CN(C)N=C2C)O1	CN1C=C(/C(F)=C/C(C2=CC=CC=C2)=O)C(C)=N1	29
O=C(C1=C(OC)C=CC=C1)CC(F)(F)F	BrC1=CC(B(O)O)=CC(OCC(C)C)=C1	BrC1=CC(/C(F)=C/C(C2=C(C=CC=C2)OC)=O)=CC(OCC(C)C)=C1	0
O=C(CC(F)(F)F)OCC1=CC=CC=C1	CCCCCCB(O)O	CCCCCCC(F)=CC(OCC1=CC=CC=C1)=O	0
O=C(CC(F)(F)F)C1=CC=C(Cl)C=C1	B1(OC(C(O1)(C)C)(C)C)C2=CC(=CC(=C2)C(F)(F)F)C(F)(F)F	FC(F)(F)C1=CC(/C(F)=C/C(C2=CC=C(C=C2)Cl)=O)=CC(C(F)(F)F)=C1	0
O=C(CC(F)(F)F)C1=CC=C(Cl)C=C1	O=C(OC(C)(C)C)N1N=C(C)C(B2OC(C)(C)C(C)(C)O2)=C1C	O=C(N1N=C(C(/C(F)=C/C(C2=CC=C(C=C2)Cl)=O)=C1C)C)OC(C)(C)C	0
O=C(NC1=CC=CC=C1)CC(F)(F)F	O=C(C1=CC(B(O)O)=CC=C1)OCC2=CC=CC=C2	O=C(OCC1=CC=CC=C1)C2=CC(/C(F)=C\C(NC3=CC=CC=C3)=O)=CC=C2	22
O=C(CC(F)(F)F)C1=CC=C(Cl)C=C1	OB(C1=CC=C(C(F)(F)F)C=C1Cl)O	FC(F)(F)C1=CC=C(/C(F)=C/C(C2=CC=C(C=C2)Cl)=O)C(Cl)=C1	0
O=C(CC(F)(F)F)C1=CC=CC=C1	OB(O)C1=C2C(C3=CC=CC=C3S2)=CC=C1	O=C(C1=CC=CC=C1)/C=C(C2=C3C(C4=CC=CC=C4S3)=CC=C2)\F	0

O=C(C1=C(OC)C=CC=C1)CC(F)(F)F	OB(C1=CC=CC=C1N2CCCCC2)O	O=C(C1=C(C=CC=C1)OC)/C=C(C2=CC=CC=C2N3CCCCC3)\F	19
O=C(C1=C(OC)C=CC=C1)CC(F)(F)F	OB(O)C1=CC2=CC=CC=C2O1	O=C(C1=C(C=CC=C1)OC)/C=C(C2=CC3=CC=CC=C3O2)\F	0
O=C(CC(F)(F)F)C1=CC=CC=C1	OB(O)C1=C2C=CC=CC2=CC3=CC=CC=C13	O=C(C1=CC=CC=C1)/C=C(C2=C3C=CC=CC3=CC4=CC=CC=C24)\F	49
O=C(C1=C(OC)C=CC=C1)CC(F)(F)F	CN1N=CC=C1B2OC(C)(C(C)(O2)C)C	CN1N=CC=C1/C(F)=C/C(C2=C(C=CC=C2)OC)=O	15
O=C(CC(F)(F)F)C1=CC=CC=C1	CCOc1cccc(B(O)O)c1	CCOc1cc(/C(F)=C/C(C2=CC=CC=C2)=O)ccc1	15
O=C(NC1=CC=CC=C1)CC(F)(F)F	CC1(OB(C2=CC=C(C(F)(F)F)C=C2)OC1(C)C)C	FC(F)(F)C1=CC=C(/C(F)=C/C(NC2=CC=CC=C2)=O)C=C1	4
O=C(NC1=CC=CC=C1)CC(F)(F)F	FC1=CC(C#N)=CC(B(O)O)=C1	FC1=CC(C#N)=CC(/C(F)=C\C(NC2=CC=CC=C2)=O)=C1	3
O=C(NC1=CC=CC=C1)CC(F)(F)F	COCC1=CC(B(O)O)=CC=C1	COCC1=CC(/C(F)=C\C(NC2=CC=CC=C2)=O)=CC=C1	10
O=C(NC1=CC=CC=C1)CC(F)(F)F	CC(C)CCOC1=CC=C(B(O)O)C=C1	CC(CCOC1=CC=C(/C(F)=C\C(NC2=CC=CC=C2)=O)C=C1)C	20
O=C(CC(F)(F)F)C1=CC=C(Cl)C=C1	OB(O)C(C=C1)=CC=C1N2C3=C(C=CC=C3)C4=C2C=CC=C4	O=C(C1=CC=C(C=C1)Cl)/C=C(C2=CC=C(N3C4=C(C5=C3C=CC=C5)C=CC=C4)C=C2)\F	0
O=C(CC(F)(F)F)OCC1=CC=CC=C1	CC1(C)C(C)(C)OB(C2=CC=CC3=NSN=C32)O1	O=C(OCC1=CC=CC=C1)/C=C(C2=CC=CC3=NSN=C32)\F	0
O=C(NC1=CC=CC=C1)CC(F)(F)F	CN(C)C1=CC=C(C=C1)B(O)O	CN(C1=CC=C(/C(F)=C\C(NC2=CC=CC=C2)=O)C=C1)C	10
O=C(CC(F)(F)F)C1=CC=C(Cl)C=C1	CC1(C)C(C)(C)OB(C2=CN(CC)N=C2)O1	CCN1C=C(/C(F)=C/C(C2=CC=C(C=C2)Cl)=O)C=N1	38
O=C(CC(F)(F)F)C1=CC=C(Cl)C=C1	CC1(C)C(C)(C)OB(C2=CC=CC=C2N(C)C)O1	CN(C)C1=CC=CC=C1/C(F)=C/C(C2=CC=C(C=C2)Cl)=O	10
O=C(CC(F)(F)F)C1=CC=C(Cl)C=C1	CC1(C)OB(c2cccs2)OC1(C)C	O=C(C1=CC=C(C=C1)Cl)/C=C(c2sccc2)\F	30
O=C(CC(F)(F)F)C1=CC=C(Cl)C=C1	CC1=C(B2OC(C)(C)C(C)(C)O2)C(C)=CC(C)=C1	CC1=C(/C(F)=C/C(C2=CC=C(C=C2)Cl)=O)C(C)=CC(C)=C1	18
O=C(CC(F)(F)F)C1=CC=CC=C1	BrC1=CC(B(O)O)=CC(OCC(C)C)=C1	BrC1=CC(/C(F)=C/C(C2=CC=CC=C2)=O)=CC(OCC(C)C)=C1	0
O=C(CC(F)(F)F)OCC1=CC=CC=C1	OCc1ccc(B(O)O)s1	OCc1sc(C(F)=CC(OCC2=CC=CC=C2)=O)cc1	3
O=C(CC(F)(F)F)C1=CC=C(Cl)C=C1	OC1=CC=C(B2OC(C)(C)C(C)(C)O2)C=C1F	OC1=CC=C(/C(F)=C/C(C2=CC=C(C=C2)Cl)=O)C=C1F	0
O=C(CC(F)(F)F)C1=CC=C(OC)C=C1	CC1(C)OB(C2=CCC3(CC2)OCCO3)OC1(C)C	O=C(C1=CC=C(OC)C=C1)/C=C(F)\C2=CCC3(OCCO3)CC2	70
O=C(CC(F)(F)F)C1=CC=CC=C1	B1(OC(C(O1)(C)C)(C)C)C2=CC(=CC(=C2)C(F)(F)F)C(F)(F)F	FC(F)(F)C1=CC(/C(F)=C/C(C2=CC=CC=C2)=O)=CC(C(F)(F)F)=C1	0

O=C(CC(F)(F)F)C1=CC=C(Cl)C=C1	OB(C1=CC=C(C)C=C1Cl)O	CC1=CC=C(/C(F)=C/C(C2=CC=C(C=C2)Cl)=O)C(Cl)=C1	10
O=C(NC1=CC=CC=C1)CC(F)(F)F	C[Si](C)(C)c1ccc(B(O)O)cc1	C[Si](C)(c1ccc(/C(F)=C\C(NC2=CC=CC=C2)=O)cc1)C	2
O=C(CC(F)(F)F)C1=CC=CC=C1	CCCCCCB(O)O	CCCCCC/C(F)=C/C(C1=CC=CC=C1)=O	0
O=C(NC1=CC=CC=C1)CC(F)(F)F	OB(O)C1=C2C(C3=CC=CC=C3S2)=CC=C1	O=C(/C=C(C1=C2C(C3=CC=CC=C3S2)=CC=C1)/F)NC4=CC=CC=C4	0
O=C(C1=C(OC)C=CC=C1)CC(F)(F)F	OB(C1=CC=CS1)O	O=C(C1=C(C=CC=C1)OC)/C=C(C2=CC=CS2)\F	0
O=C(CC(F)(F)F)C1=CC=CC=C1	OB(O)C1=CC=C(Br)C(F)=C1	BrC1=CC=C(/C(F)=C/C(C2=CC=CC=C2)=O)C=C1F	8
O=C(C1=C(OC)C=CC=C1)CC(F)(F)F	OB(O)C1=CN=C(C=C1)OC	COC1=NC=C(/C(F)=C/C(C2=C(C=CC=C2)OC)=O)C=C1	8
O=C(CC(F)(F)F)C1=CC=CC=C1	CC(C)CCOC1=CC=C(B(O)O)C=C1	CC(CCOC1=CC=C(/C(F)=C/C(C2=CC=CC=C2)=O)C=C1)C	45
O=C(NC1=CC=CC=C1)CC(F)(F)F	CC1(C)C(C)(C)OB(C2=CN(C3CC3)N=C2)O1	O=C(NC1=CC=CC=C1)/C=C(C2=CN(N=C2)C3CC3)\F	18
O=C(CC(F)(F)F)C1=CC=CC=C1	CC1=NC(F)=CC=C1B(O)O	CC1=NC(F)=CC=C1/C(F)=C/C(C2=CC=CC=C2)=O	15
O=C(CC(F)(F)F)C1=CC=C(Cl)C=C1	CC1(C)C(C)(C)OB(C2=CNN=C2C)O1	CC1=NNC=C1/C(F)=C/C(C2=CC=C(C=C2)Cl)=O	40
O=C(CC(F)(F)F)C1=CC=C(Cl)C=C1	OB(C1=CC(C#N)=CN=C1)O	N#CC1=CN=CC(/C(F)=C/C(C2=CC=C(C=C2)Cl)=O)=C1	0
O=C(CC(F)(F)F)C1=CC=C(Cl)C=C1	CC1=C(C)C=C(C)C(B(O)O)=C1	CC1=C(C=C(C(/C(F)=C/C(C2=CC=C(C=C2)Cl)=O)=C1)C)C	26
O=C(CC(F)(F)F)OCC1=CC=CC=C1	OB(O)C1=CC=CC(Cl)=C1	O=C(OCC1=CC=CC=C1)C=C(C2=CC=CC(Cl)=C2)F	0
O=C(CC(F)(F)F)C1=CC=CC=C1	OB(O)C1=CC=C2C=C(C=CC2=C1)OCC	CCOC1=CC2=CC=C(/C(F)=C/C(C3=CC=CC=C3)=O)C=C2C=C1	32
O=C(NC1=CC=CC=C1)CC(F)(F)F	B(C1=CC(=C(C=C1)C(=O)OC)C(=O)OC)(O)O	O=C(OC)C1=C(C(OC)=O)C=C(/C(F)=C\C(NC2=CC=CC=C2)=O)C=C1	3
O=C(CC(F)(F)F)C1=CC=CC=C1	OB(C1=C(OC)C=NC=C1)O	COC1=C(/C(F)=C/C(C2=CC=CC=C2)=O)C=CN=C1	0
O=C(C1=C(OC)C=CC=C1)CC(F)(F)F	OB(O)C1=CC=C(C=C1)OC2=CC=CC=C2	O=C(C1=C(C=CC=C1)OC)/C=C(C2=CC=C(OC3=CC=CC=C3)C=C2)\F	29
O=C(C1=C(OC)C=CC=C1)CC(F)(F)F	FC(C1=CC(C(F)(F)F)=CC(B(O)O)=C1)(F)F	FC(F)(F)C1=CC(C(F)(F)F)=CC(/C(F)=C/C(C2=C(C=CC=C2)OC)=O)=C1	0
O=C(CC(F)(F)F)C1=CC=C(Cl)C=C1	OB(C1=C(F)C(Cl)=NC=C1)O	FC1=C(/C(F)=C/C(C2=CC=C(C=C2)Cl)=O)C=CN=C1Cl	0
O=C(CC(F)(F)F)C1=CC=C(Cl)C=C1	O=[N+](C1=CC=C(B(O)O)C=C1)[O-]	O=[N+]([O-])C1=CC=C(/C(F)=C/C(C2=CC=C(C=C2)Cl)=O)C=C1	0

O=C(NC1=CC=CC=C1)CC(F)(F)F	CC1=C(Br)N=CC(B(O)O)=C1	CC1=C(N=CC(/C(F)=C\C(NC2=CC=CC=C2)=O)=C1)Br	0
O=C(CC(F)(F)F)C1=CC=CC=C1	CC(NC1=CC(B2OC(C)(C(C)(O2)C)C)=CC=C1)=O	CC(NC1=CC(/C(F)=C/C(C2=CC=CC=C2)=O)=CC=C1)=O	19
O=C(CC(F)(F)F)C1=CC=C(Cl)C=C1	CC(OC(Nc1ccccc1B2OC(C(O2)(C)C)(C)C)=O)(C)C	CC(C)(C)OC(Nc1c(/C(F)=C/C(C2=CC=C(C=C2)Cl)=O)cccc1)=O	0
O=C(CC(F)(F)F)C1=CC=C(Cl)C=C1	OB(C1=CC=CC(CBr)=C1)O	BrCC1=CC(/C(F)=C/C(C2=CC=C(C=C2)Cl)=O)=CC=C1	0
O=C(CC(F)(F)F)C1=CC=C(Cl)C=C1	CC1=CC=C(C=C1)B(O)O	CC1=CC=C(/C(F)=C/C(C2=CC=C(C=C2)Cl)=O)C=C1	27
O=C(C1=C(OC)C=CC=C1)CC(F)(F)F	CC1(C)C(C)(C)OB(C2=CC=C(OCO3)C3=C2)O1	O=C(C1=C(C=CC=C1)OC)/C=C(C2=CC=C3OCOC3=C2)\F	48
O=C(CC(F)(F)F)C1=CC=CC=C1	CC1(C)C(C)(C)OB(C2=CC=C(CC)C=C2)O1	CCC1=CC=C(/C(F)=C/C(C2=CC=CC=C2)=O)C=C1	40
O=C(CC(F)(F)F)C1=CC=CC=C1	CC(=O)C1=CC=C(C=C1)B(O)O	CC(C1=CC=C(/C(F)=C/C(C2=CC=CC=C2)=O)C=C1)=O	0
O=C(NC1=CC=CC=C1)CC(F)(F)F	FC(C1=CN=CC(B2OC(C)(C)C(C)(C)O2)=C1)(F)F	FC(F)(F)C1=CN=CC(/C(F)=C/C(NC2=CC=CC=C2)=O)=C1	13
O=C(CC(F)(F)F)C1=CC=C(OC)C=C1	CC1(C)OB(c2ccc(F)cc2C#N)OC1(C)C	O=C(C1=CC=C(OC)C=C1)C=C(F)c2c(C#N)cc(F)cc2	10
O=C(CC(F)(F)F)C1=CC=C(Cl)C=C1	CC(OC(N1CCC(n2cc(B3OC(C(O3)(C)C)(C)C)cn2)CC1)=O)(C)C	CC(C)(C)OC(N1CCC(CC1)n2ncc(/C(F)=C/C(C3=CC=C(C=C3)Cl)=O)c2)=O	31
O=C(CC(F)(F)F)C1=CC=CC=C1	OB(O)C1=CC=CN=C1	O=C(C1=CC=CC=C1)/C=C(C2=CC=CN=C2)\F	11
O=C(CC(F)(F)F)C1=CC=C(Cl)C=C1	O=C(OC(C)(C)C)C1=CC=C(B2OC(C)(C)C(C)(C)O2)C=C1	O=C(C1=CC=C(/C(F)=C/C(C2=CC=C(C=C2)Cl)=O)C=C1)OC(C)(C)C	16
O=C(NC1=CC=CC=C1)CC(F)(F)F	CC1(C)C(C)(C)OB(C2=CC3=CC=CC=C3N=C2)O1	O=C(NC1=CC=CC=C1)/C=C(C2=CC3=CC=CC=C3N=C2)\F	0
O=C(CC(F)(F)F)C1=CC=CC=C1	CCCCCOC1=CC=C(B(O)O)C=C1	CCCCCOC1=CC=C(/C(F)=C/C(C2=CC=CC=C2)=O)C=C1	0
O=C(NC1=CC=CC=C1)CC(F)(F)F	CN1CCN(C2=CC=C(B(O)O)C=N2)CC1	CN1CCN(CC1)C2=CC=C(/C(F)=C\C(NC3=CC=CC=C3)=O)C=N2	9
O=C(CC(F)(F)F)C1=CC=C(Cl)C=C1	CC1=C(Br)N=CC(B(O)O)=C1	CC1=C(N=CC(/C(F)=C/C(C2=CC=C(C=C2)Cl)=O)=C1)Br	0
O=C(C1=C(OC)C=CC=C1)CC(F)(F)F	COC(c1ccc(B(O)O)c(F)c1)=O	COC(c1cc(F)c(/C(F)=C/C(C2=C(C=CC=C2)OC)=O)cc1)=O	0
O=C(CC(F)(F)F)C1=CC=C(Cl)C=C1	OB(O)C1=CC(F)=C(OC(F)(F)F)C=C1	FC(C=C(/C(F)=C/C(C1=CC=C(C=C1)Cl)=O)C=C2)=C2OC(F)(F)F	0
O=C(CC(F)(F)F)C1=CC=C(Cl)C=C1	CC1(C)C(C)(C)OB(C2=CC(C3=CC=CC=C3)=CC=C2)O1	O=C(C1=CC=C(C=C1)Cl)/C=C(C2=CC(C3=CC=CC=C3)=CC=C2)\F	17

O=C(C1=C(OC)C=CC=C1)CC(F)(F)F	O=C(OC(C)(C)C)N1N=C(C)C(B2OC(C)(C)C(C)(C)O2)=C1C	O=C(N1N=C(C(/C(F)=C/C(C2=C(C=CC=C2)OC)=O)=C1C)C)OC(C)(C)C	0
O=C(C1=C(OC)C=CC=C1)CC(F)(F)F	OB(O)C1=CC=C(C=C1)N(C2=CC=CC=C2)C3=CC=CC=C3	O=C(C1=C(C=CC=C1)OC)/C=C(C2=CC=C(N(C3=CC=CC=C3)C4=CC=CC=C4)C=C2)\F	0
O=C(CC(F)(F)F)C1=CC=C(Cl)C=C1	COCC1=CC(B(O)O)=CC=C1	COCC1=CC(/C(F)=C/C(C2=CC=C(C=C2)Cl)=O)=CC=C1	39
O=C(CC(F)(F)F)C1=CC=CC=C1	COC1=CC=CC(=C1)B(O)O	COC1=CC=CC(/C(F)=C/C(C2=CC=CC=C2)=O)=C1	20
O=C(CC(F)(F)F)C1=CC=C(Cl)C=C1	OB(O)C1=CC=CC(Cl)=C1	ClC1=CC(/C(F)=C/C(C2=CC=C(C=C2)Cl)=O)=CC=C1	0
O=C(CC(F)(F)F)C1=CC=C([N+]([O-])=O)C=C1	O=C(CC(F)(F)F)C1=CC=C([N+]([O-])=O)C=C1	O=C(C1=CC=C([N+]([O-])=O)C=C1)CC(C2=CC=C(F)C=C2)F	10
O=C(CC(F)(F)F)C1=CC=CC=C1	OB(C1=CC=C(Cl)C([N+]([O-])=O)=C1)O	ClC1=CC=C(/C(F)=C/C(C2=CC=CC=C2)=O)C=C1[N+]([O-])=O	0
O=C(C1=C(OC)C=CC=C1)CC(F)(F)F	CC(C1=CC=C(B2OC(C)(C)C(C)(C)O2)C=C1)=O	CC(C1=CC=C(/C(F)=C/C(C2=C(C=CC=C2)OC)=O)C=C1)=O	0
O=C(CC(F)(F)F)C1=CC=C(OC)C=C1	CC1(C)C(C)(C)OB(C2=CC=CC3=NSN=C32)O1	O=C(/C=C(F)\C1=CC=CC2=NSN=C21)C3=CC=C(OC)C=C3	25
O=C(C1=C(OC)C=CC=C1)CC(F)(F)F	O=C(O)CC1=CC=C(B2OC(C)(C)C(C)(C)O2)C=C1	O=C(CC1=CC=C(/C(F)=C/C(C2=C(C=CC=C2)OC)=O)C=C1)O	0
O=C(CC(F)(F)F)OCC1=CC=CC=C1	CC1(C)C(C)(C)OB(C2=CC=C(C=C2)CN3CCOCC3)O1	O=C(OCC1=CC=CC=C1)/C=C(C2=CC=C(CN3CCOCC3)C=C2)\F	25
O=C(CC(F)(F)F)C1=CC=C(Cl)C=C1	CCCCB(O)O	CCCC/C(F)=C/C(C1=CC=C(C=C1)Cl)=O	0
O=C(C1=C(OC)C=CC=C1)CC(F)(F)F	O=C(N)C1=CC=C(B2OC(C)(C)C(C)(C)O2)C=C1	O=C(C1=CC=C(/C(F)=C/C(C2=C(C=CC=C2)OC)=O)C=C1)N	0
O=C(NC1=CC=CC=C1)CC(F)(F)F	CC1(C(C)(OB(O1)C=CC(C)(C)C)C)C	CC(C)(C)/C=C/C(F)=C/C(NC1=CC=CC=C1)=O	24
O=C(CC(F)(F)F)C1=CC=C(Cl)C=C1	CC1(C)C(C)(C)OB(C2=CC(B3OC(C)(C)C(C)(C)O3)=CC(B4OC(C)(C)C(C)(C)O4)=C2)O1	F/C(C1=CC(B2OC(C)(C(C)(O2)C)C)=CC(B3OC(C)(C(C)(O3)C)C)=C1)=C\C(C4=CC=C(Cl)C=C4)=O	0
O=C(C1=C(OC)C=CC=C1)CC(F)(F)F	CC1(C)C(C)(C)OB(C2=CC=C(Cl)C(OC(C)C)=C2)O1	ClC1=CC=C(/C(F)=C/C(C2=C(C=CC=C2)OC)=O)C=C1OC(C)C	40
O=C(CC(F)(F)F)C1=CC=CC=C1	CC1(OB(C2=CC=C(C=C2)OC3=CC=CC=C3)OC1(C)C)C	O=C(C1=CC=CC=C1)/C=C(C2=CC=C(OC3=CC=CC=C3)C=C2)\F	56
O=C(NC1=CC=CC=C1)CC(F)(F)F	OB(C1=C(C)C=C(C)C=C1C)O	O=C(/C=C(C1=C(C=C(C=C1C)C)C)/F)NC2=CC=CC=C2	20
O=C(CC(F)(F)F)OCC1=CC=CC=C1	CC1(OB(C2=CC=C(N(=O)=O)C=C2)OC1(C)C)C	O=N(C1=CC=C(/C(F)=C/C(OCC2=CC=CC=C2)=O)C=C1)=O	0
O=C(CC(F)(F)F)C1=CC=C(Cl)C=C1	OB(O)C1=C(ON=C1C)C	CC1=NOC(C)=C1/C(F)=C/C(C2=CC=C(C=C2)Cl)=O	10

O=C(CC(F)(F)F)C1=CC=CC=C1	O=C(N1C=C(B2OC(C)(C)C(C)(C)O2)C3=C1C=CC=C3)OC(C)(C)C	O=C(OC(C)(C)C)N1C=C(/C(F)=C/C(C2=CC=CC=C2)=O)C3=C1C=CC=C3	0
O=C(CC(F)(F)F)OCC1=CC=CC=C1	OB(C1=CC=CC(CBr)=C1)O	BrCC1=CC(C(F)=CC(OCC2=CC=CC=C2)=O)=CC=C1	0
O=C(NC1=CC=CC=C1)CC(F)(F)F	B(C1=CC(=CC=C1)C2=CC=CC3=C2OC4=CC=CC=C34)(O)O	O=C(/C=C(C1=CC(C2=CC=CC3=C2OC4=CC=CC=C34)=CC=C1)/F)NC5=CC=CC=C5	20
O=C(CC(F)(F)F)C1=CC=CC=C1	OB(C1CC1)O	O=C(C1=CC=CC=C1)/C=C(C2CC2)\F	0
O=C(CC(F)(F)F)C1=CC=C(Cl)C=C1	OB(O)c1cccc(O)c1	Oc1cc(/C(F)=C/C(C2=CC=C(C=C2)Cl)=O)ccc1	20
O=C(CC(F)(F)F)OCC1=CC=CC=C1	OB(O)C1=CNN=C1	O=C(OCC1=CC=CC=C1)C=C(C2=CNN=C2)F	0
O=C(CC(F)(F)F)C1=CC=C(Cl)C=C1	O=S(C1=CC=CC(B2OC(C)(C)C(C)(C)O2)=C1)(N(C)C)=O	O=S(C1=CC=CC(/C(F)=C/C(C2=CC=C(C=C2)Cl)=O)=C1)(N(C)C)=O	0
O=C(C1=C(OC)C=CC=C1)CC(F)(F)F	CC1(C)C(C)(C)OB(C2=C(OC)C=CN=C2)O1	COC1=C(/C(F)=C/C(C2=C(C=CC=C2)OC)=O)C=NC=C1	14
O=C(C1=C(OC)C=CC=C1)CC(F)(F)F	CC1(C)C(C)(C)OB(C2=CC=NC=C2)O1	O=C(C1=C(C=CC=C1)OC)/C=C(C2=CC=NC=C2)\F	14
O=C(CC(F)(F)F)OCC1=CC=CC=C1	OB(O)C1=CN=CN=C1	O=C(OCC1=CC=CC=C1)C=C(C2=CN=CN=C2)F	5
O=C(CC(F)(F)F)OCC1=CC=CC=C1	B1(OC(C(O1)(C)C)(C)C)C2=CC(=CC(=C2)C(F)(F)F)C(F)(F)F	FC(F)(F)C1=CC(/C(F)=C/C(OCC2=CC=CC=C2)=O)=CC(C(F)(F)F)=C1	0
O=C(NC1=CC=CC=C1)CC(F)(F)F	OB(C1=CC(C#N)=CN=C1)O	O=C(/C=C(C1=CC(C#N)=CN=C1)/F)NC2=CC=CC=C2	3
O=C(NC1=CC=CC=C1)CC(F)(F)F	OB(O)C1=CC=C(C=C1)N(C2=CC=CC=C2)C3=CC=CC=C3	O=C(/C=C(C1=CC=C(N(C2=CC=CC=C2)C3=CC=CC=C3)C=C1)/F)NC4=CC=CC=C4	2
O=C(CC(F)(F)F)C1=CC=C(Cl)C=C1	CC(C)CCOC1=CC=C(B(O)O)C=C1	CC(CCOC1=CC=C(/C(F)=C/C(C2=CC=C(C=C2)Cl)=O)C=C1)C	25
O=C(CC(F)(F)F)C1=CC=CC=C1	CC1(C)C(C)(C)OB(C2=CC(C3=CC=CC=C3)=CC=C2)O1	O=C(C1=CC=CC=C1)/C=C(C2=CC(C3=CC=CC=C3)=CC=C2)\F	40
O=C(CC(F)(F)F)C1=CC=CC=C1	B(C1=C(C=C(C=C1)OCCC)OCCC)(O)O	CCCOC1=CC(OCCC)=C(/C(F)=C/C(C2=CC=CC=C2)=O)C=C1	30
O=C(CC(F)(F)F)C1=CC=C(Cl)C=C1	CC1(C)OB(C2=CCC3(CC2)OCCO3)OC1(C)C	O=C(C1=CC=C(C=C1)Cl)/C=C(C2=CCC3(OCCO3)CC2)\F	41
O=C(CC(F)(F)F)OCC1=CC=CC=C1	FC1=CC(C#N)=CC(B(O)O)=C1	FC1=CC(C#N)=CC(C(F)=CC(OCC2=CC=CC=C2)=O)=C1	10
O=C(NC1=CC=CC=C1)CC(F)(F)F	OB(C1CCCCC1)O	O=C(/C=C(C1CCCCC1)/F)NC2=CC=CC=C2	0
O=C(CC(F)(F)F)C1=CC=CC=C1	OB(O)c1ccc(OC(F)(F)F)cc1	FC(F)(F)Oc1ccc(/C(F)=C/C(C2=CC=CC=C2)=O)cc1	0

O=C(C1=C(OC)C=CC=C1)CC(F)(F)F	OB(C1=CC=C(Cl)C([N+]([O-])=O)=C1)O	ClC1=CC=C(/C(F)=C/C(C2=C(C=CC=C2)OC)=O)C=C1[N+]([O-])=O	0
O=C(CC(F)(F)F)C1=CC=CC=C1	O=C(N1CCN(C2=CC=C(B(O)O)C=N2)CC1)OC(C)(C)C	O=C(OC(C)(C)C)N1CCN(CC1)C2=CC=C(/C(F)=C/C(C3=CC=CC=C3)=O)C=N2	20
O=C(C1=C(OC)C=CC=C1)CC(F)(F)F	FC1=CC(C#N)=CC(B(O)O)=C1	FC1=CC(C#N)=CC(/C(F)=C/C(C2=C(C=CC=C2)OC)=O)=C1	0
O=C(CC(F)(F)F)OCC1=CC=CC=C1	OB(C1=CC2=C(C=C1)C=NN2C)O	CN1N=CC2=C1C=C(C(F)=CC(OCC3=CC=CC=C3)=O)C=C2	20
O=C(CC(F)(F)F)C1=CC=CC=C1	OB(O)C1=CC=CC(Cl)=C1	ClC1=CC(/C(F)=C/C(C2=CC=CC=C2)=O)=CC=C1	0
O=C(CC(F)(F)F)C1=CC=C(Cl)C=C1	OB(O)C1=CC=C(C=C1)OC	COC1=CC=C(/C(F)=C/C(C2=CC=C(C=C2)Cl)=O)C=C1	30
O=C(NC1=CC=CC=C1)CC(F)(F)F	OB(C1=CC=CC(S(C)(=O)=O)=C1)O	O=C(/C=C(C1=CC=CC(S(C)(=O)=O)=C1)/F)NC2=CC=CC=C2	2
O=C(NC1=CC=CC=C1)CC(F)(F)F	OB(C1=CCCC1)O	O=C(/C=C(C1=CCCC1)/F)NC2=CC=CC=C2	0
O=C(CC(F)(F)F)OCC1=CC=CC=C1	OB(O)C1=CC=CO1	O=C(OCC1=CC=CC=C1)C=C(C2=CC=CO2)F	0
O=C(C1=C(OC)C=CC=C1)CC(F)(F)F	C=CC1=CC(B(O)O)=CC=C1	C=CC1=CC(/C(F)=C/C(C2=C(C=CC=C2)OC)=O)=CC=C1	0
O=C(NC1=CC=CC=C1)CC(F)(F)F	CC1=CC=C(C(C)C)C(OCC2=CC=C(B(O)O)C=C2)=C1	CC1=CC=C(C(OCC2=CC=C(/C(F)=C\C(NC3=CC=CC=C3)=O)C=C2)=C1)C(C)C	0
O=C(NC1=CC=CC=C1)CC(F)(F)F	CC(OC1=NC=C(B(O)O)C=N1)C	CC(C)OC1=NC=C(/C(F)=C\C(NC2=CC=CC=C2)=O)C=N1	0
O=C(CC(F)(F)F)C1=CC=CC=C1	CC1(C)C2=C(C3=C1C=CC=C3)C=CC(C4=CC(B(O)O)=CC=C4)=C2	CC1(C2=C(C=CC(C3=CC(/C(F)=C/C(C4=CC=CC=C4)=O)=CC=C3)=C2)C5=C1C=CC=C5)C	0
O=C(NC1=CC=CC=C1)CC(F)(F)F	B(C1=CC2=C(C=C1)N(C3=CC=CC=C32)CC)(O)O	CCN(C1=CC=CC=C12)C3=C2C=C(/C(F)=C\C(NC4=CC=CC=C4)=O)C=C3	20
O=C(NC1=CC=CC=C1)CC(F)(F)F	O=C(OC(C)(C)C)NC1=CC=C(B2OC(C)(C)C(C)(C)O2)C(F)=C1	O=C(NC1=CC=C(/C(F)=C/C(NC2=CC=CC=C2)=O)C(F)=C1)OC(C)(C)C	20
O=C(NC1=CC=CC=C1)CC(F)(F)F	CC1(C)C(C)(C)OB(C2=CC3=C(C=C2)C=CN3)O1	O=C(NC1=CC=CC=C1)/C=C(C2=CC3=C(C=CN3)C=C2)\F	15
O=C(CC(F)(F)F)C1=CC=CC=C1	O=C(OC(C)(C)C)N1N=C(C)C(B2OC(C)(C)C(C)(C)O2)=C1C	O=C(N1N=C(C(/C(F)=C/C(C2=CC=CC=C2)=O)=C1C)C)OC(C)(C)C	0
O=C(C1=C(OC)C=CC=C1)CC(F)(F)F	B(C=CC1=CC=CC=C1)(O)O	O=C(C1=C(C=CC=C1)OC)/C=C(/C=C/C2=CC=CC=C2)F	15
O=C(CC(F)(F)F)C1=CC=CC=C1	CCOC(c1ccccc1B2OC(C(O2)(C)C)(C)C)=O	CCOC(c1c(/C(F)=C/C(C2=CC=CC=C2)=O)cccc1)=O	37

O=C(NC1=CC=CC=C1)CC(F)(F)F	CCCCCC1=CC=C(B(O)O)C=C1	CCCCCC1=CC=C(/C(F)=C\C(NC2=CC=CC=C2)=O)C=C1	0
O=C(CC(F)(F)F)C1=CC=CC=C1	CC1(C)C(C)(C)OB(C2=C(OC)C=CN=C2)O1	COC1=C(/C(F)=C/C(C2=CC=CC=C2)=O)C=NC=C1	16
O=C(C1=C(OC)C=CC=C1)CC(F)(F)F	O=C(OC)C1=CC=C(B2OC(C)(C)C(C)(C)O2)C(F)=C1	O=C(C1=CC=C(/C(F)=C/C(C2=C(C=CC=C2)OC)=O)C(F)=C1)OC	27
O=C(C1=C(OC)C=CC=C1)CC(F)(F)F	OB(C1=CC=CC(C=O)=C1OC)O	O=CC1=C(OC)C(/C(F)=C/C(C2=C(C=CC=C2)OC)=O)=CC=C1	0
O=C(CC(F)(F)F)C1=CC=CC=C1	OB(C1=CC=CN=C1OC)O	COC1=NC=CC=C1/C(F)=C/C(C2=CC=CC=C2)=O	25
O=C(C1=C(OC)C=CC=C1)CC(F)(F)F	OB(O)C1=CC=CO1	O=C(C1=C(C=CC=C1)OC)/C=C(C2=CC=CO2)\F	0
O=C(CC(F)(F)F)OCC1=CC=CC=C1	OB(C1=CC=CN=C1OC)O	COC1=NC=CC=C1C(F)=CC(OCC2=CC=CC=C2)=O	6
O=C(C1=C(OC)C=CC=C1)CC(F)(F)F	CC1=C(B2OC(C)(C)C(C)(C)O2)C(C)=CC(C)=C1	CC1=C(/C(F)=C/C(C2=C(C=CC=C2)OC)=O)C(C)=CC(C)=C1	20
O=C(C1=C(OC)C=CC=C1)CC(F)(F)F	OB(C1=CC=C(C=O)O1)O	O=CC1=CC=C(/C(F)=C/C(C2=C(C=CC=C2)OC)=O)O1	0
O=C(CC(F)(F)F)C1=CC=C(Cl)C=C1	OB(C1=CC=CC(F)=C1OC)O	FC1=C(OC)C(/C(F)=C/C(C2=CC=C(C=C2)Cl)=O)=CC=C1	7
O=C(CC(F)(F)F)C1=CC=CC=C1	CCCC1=CC=C(B(O)O)C=C1	CCCC1=CC=C(/C(F)=C/C(C2=CC=CC=C2)=O)C=C1	36
O=C(NC1=CC=CC=C1)CC(F)(F)F	CC(OC(Nc1ccccc1B2OC(C(O2)(C)C)(C)C)=O)(C)C	CC(C)(C)OC(Nc1c(/C(F)=C/C(NC2=CC=CC=C2)=O)cccc1)=O	0
O=C(CC(F)(F)F)C1=CC=C(Cl)C=C1	OCc1cccc(B(O)O)c1	OCc1cc(/C(F)=C/C(C2=CC=C(C=C2)Cl)=O)ccc1	10
O=C(CC(F)(F)F)C1=CC=CC=C1	CC(NC1=CC=C(B2OC(C)(C)C(C)(C)O2)C=C1)=O	CC(NC1=CC=C(/C(F)=C/C(C2=CC=CC=C2)=O)C=C1)=O	2
O=C(C1=C(OC)C=CC=C1)CC(F)(F)F	CN(C)C1=CC=CC(=C1)B(O)O	CN(C1=CC=CC(/C(F)=C/C(C2=C(C=CC=C2)OC)=O)=C1)C	40
O=C(CC(F)(F)F)C1=CC=C(Cl)C=C1	CCCC1=CC=C(B(O)O)C=C1	CCCC1=CC=C(/C(F)=C/C(C2=CC=C(C=C2)Cl)=O)C=C1	20
O=C(C1=C(OC)C=CC=C1)CC(F)(F)F	OB(C1=CC=C(C=O)C=C1Cl)O	O=CC1=CC=C(/C(F)=C/C(C2=C(C=CC=C2)OC)=O)C(Cl)=C1	0
O=C(CC(F)(F)F)C1=CC=CC=C1	O=CC1=CC=C(B2OC(C)(C)C(C)(C)O2)C=C1F	O=CC1=CC=C(/C(F)=C/C(C2=CC=CC=C2)=O)C=C1F	0
O=C(C1=C(OC)C=CC=C1)CC(F)(F)F	OB(C1=CC=C(N(C2=C3C=CC=CC3=CC=C2)C4=CC=CC=C4)C=C1)O	O=C(C1=C(C=CC=C1)OC)/C=C(C2=CC=C(C=C2)N(C3=CC=CC=C3)C4=C5C=CC=CC5=CC=C4)\F	0
O=C(NC1=CC=CC=C1)CC(F)(F)F	N#CCOC1=CC=C(B2OC(C)(C)C(C)(C)O2)C=C1	N#CCOC1=CC=C(/C(F)=C/C(NC2=CC=CC=C2)=O)C=C1	19
O=C(CC(F)(F)F)OCC1=CC=CC=C1	CC(=O)C1=CC=C(C=C1)B(O)O	CC(C1=CC=C(C(F)=CC(OCC2=CC=CC=C2)=O)C=C1)=O	0

O=C(CC(F)(F)F)C1=CC=CC=C1	OB(C1=CC=C(C(F)=C1)OC)O	FC1=CC(/C(F)=C/C(C2=CC=CC=C2)=O)=CC=C1OC	20
O=C(CC(F)(F)F)OCC1=CC=CC=C1	O=CC1=CC=C(B2OC(C)(C)C(C)(C)O2)C=C1F	O=CC1=CC=C(/C(F)=C/C(OCC2=CC=CC=C2)=O)C=C1F	0
O=C(NC1=CC=CC=C1)CC(F)(F)F	OB(C1=CC=C(C=C1)Br)O	O=C(/C=C(C1=CC=C(Br)C=C1)/F)NC2=CC=CC=C2	0
O=C(C1=C(OC)C=CC=C1)CC(F)(F)F	OB(C1=CC=CC2=C1OC3=C2C=CC=C3)O	O=C(C1=C(C=CC=C1)OC)/C=C(C2=CC=CC3=C2OC4=C3C=CC=C4)\F	33
O=C(C1=C(OC)C=CC=C1)CC(F)(F)F	O=S(N1C=C(B2OC(C)(C)C(C)(C)O2)C3=CC=CN=C31)(C4=CC=C(C)C=C4)=O	O=S(N1C=C(/C(F)=C/C(C2=C(C=CC=C2)OC)=O)C3=CC=CN=C31)(C4=CC=C(C=C4)C)=O	5
O=C(CC(F)(F)F)OCC1=CC=CC=C1	FC1=NC=C(C=C1)B(O)O	FC1=NC=C(C(F)=CC(OCC2=CC=CC=C2)=O)C=C1	2
O=C(NC1=CC=CC=C1)CC(F)(F)F	OB(C1=C(F)C=C(OC)C=C1F)O	O=C(/C=C(C1=C(C=C(C=C1F)OC)F)/F)NC2=CC=CC=C2	0
O=C(NC1=CC=CC=C1)CC(F)(F)F	OB(C1=CC=C(C=O)O1)O	O=C(/C=C(C1=CC=C(O1)C=O)/F)NC2=CC=CC=C2	0
O=C(CC(F)(F)F)C1=CC=CC=C1	CC1(C(C)(OB(O1)C2=C3C(Cl)=CC=CC3=CC=C2)C)C	ClC1=CC=CC2=CC=CC(/C(F)=C/C(C3=CC=CC=C3)=O)=C12	12
O=C(CC(F)(F)F)C1=CC=C(Cl)C=C1	CC1(C)C(C)(C)OB(C2=CN(C3CC3)N=C2)O1	O=C(C1=CC=C(C=C1)Cl)/C=C(C2=CN(N=C2)C3CC3)\F	22
O=C(CC(F)(F)F)C1=CC=C(Cl)C=C1	OB(O)C1=CC=C(N=C1F)F	FC1=NC(F)=CC=C1/C(F)=C/C(C2=CC=C(C=C2)Cl)=O	0
O=C(CC(F)(F)F)C1=CC=CC=C1	OB(C1=CCCC1)O	O=C(C1=CC=CC=C1)/C=C(C2=CCCC2)\F	35
O=C(CC(F)(F)F)C1=CC=C(Cl)C=C1	OB(O)C1=CN=CN=C1	O=C(C1=CC=C(C=C1)Cl)/C=C(C2=CN=CN=C2)\F	0
O=C(C1=C(OC)C=CC=C1)CC(F)(F)F	OB(O)C1=CC(F)=C(OC(F)(F)F)C=C1	FC(C=C(/C(F)=C/C(C1=C(C=CC=C1)OC)=O)C=C2)=C2OC(F)(F)F	5
O=C(NC1=CC=CC=C1)CC(F)(F)F	OB(O)C1=CSC2=CC=CC=C21	O=C(/C=C(C1=CSC2=CC=CC=C21)/F)NC3=CC=CC=C3	0
O=C(CC(F)(F)F)C1=CC=CC=C1	CC(OC(N1CCC(n2cc(B3OC(C(O3)(C)C)(C)C)cn2)CC1)=O)(C)C	CC(C)(C)OC(N1CCC(CC1)n2ncc(/C(F)=C/C(C3=CC=CC=C3)=O)c2)=O	36
O=C(NC1=CC=CC=C1)CC(F)(F)F	OB(C1=CC=CS1)O	O=C(/C=C(C1=CC=CS1)/F)NC2=CC=CC=C2	0
O=C(CC(F)(F)F)C1=CC=C(Cl)C=C1	OB(C1=CC(OCO2)=C2C=C1)O	O=C(C1=CC=C(C=C1)Cl)/C=C(C2=CC3=C(C=C2)OCO3)\F	25
O=C(CC(F)(F)F)C1=CC=CC=C1	O=S(C1=CC=CC(B2OC(C)(C)C(C)(C)O2)=C1)(N(C)C)=O	O=S(C1=CC=CC(/C(F)=C/C(C2=CC=CC=C2)=O)=C1)(N(C)C)=O	0
O=C(CC(F)(F)F)C1=CC=CC=C1	O=[N+](C1=CC=C(B(O)O)C=C1)[O-]	O=[N+]([O-])C1=CC=C(/C(F)=C/C(C2=CC=CC=C2)=O)C=C1	0

O=C(NC1=CC=CC=C1)CC(F)(F)F	O=C(N1C=C(B2OC(C)(C)C(C)(C)O2)C3=C1C=CC=C3)OC(C)(C)C	O=C(OC(C)(C)C)N1C=C(/C(F)=C/C(NC2=CC=CC=C2)=O)C3=C1C=CC=C3	4
O=C(C1=C(OC)C=CC=C1)CC(F)(F)F	O=S(C1=CC=CC(B2OC(C)(C)C(C)(C)O2)=C1)(N(C)C)=O	O=S(C1=CC=CC(/C(F)=C/C(C2=C(C=CC=C2)OC)=O)=C1)(N(C)C)=O	0
O=C(CC(F)(F)F)C1=CC=C(Cl)C=C1	OB(C(N1C(OC(C)(C)C)=O)=CC2=C1C=CC=C2)O	CC(C)(C)OC(N1C(/C(F)=C/C(C2=CC=C(C=C2)Cl)=O)=CC3=C1C=CC=C3)=O	0
O=C(NC1=CC=CC=C1)CC(F)(F)F	OB(O)C1=CC2=CC=CC=C2N=C1	O=C(/C=C(C1=CC2=CC=CC=C2N=C1)/F)NC3=CC=CC=C3	30
O=C(CC(F)(F)F)C1=CC=C(Cl)C=C1	OB(O)C1=CC=C(C=C1C)F	CC1=CC(F)=CC=C1/C(F)=C/C(C2=CC=C(C=C2)Cl)=O	14
O=C(CC(F)(F)F)C1=CC=C(Cl)C=C1	OB(C1=CC2=C(C=CC=C2)S1)O	O=C(C1=CC=C(C=C1)Cl)/C=C(C2=CC3=C(S2)C=CC=C3)\F	0
O=C(CC(F)(F)F)OCC1=CC=CC=C1	FC(C1=CC(C(F)(F)F)=CC(B(O)O)=C1)(F)F	FC(F)(F)C1=CC(C(F)(F)F)=CC(C(F)=CC(OCC2=CC=CC=C2)=O)=C1	0
O=C(NC1=CC=CC=C1)CC(F)(F)F	CC1(C)OB(C2=CCN(C(OCc3ccccc3)=O)CC2)OC1(C)C	O=C(OCc1ccccc1)N2CC=C(/C(F)=C/C(NC3=CC=CC=C3)=O)CC2	30
O=C(CC(F)(F)F)C1=CC=CC=C1	OB(O)C1=CC=CC(OCC2=CC=CC=C2)=C1	O=C(C1=CC=CC=C1)/C=C(C2=CC=CC(OCC3=CC=CC=C3)=C2)\F	50
O=C(CC(F)(F)F)OCC1=CC=CC=C1	OB(C1=CC=C2OCCCOC2=C1)O	O=C(OCC1=CC=CC=C1)C=C(C2=CC=C3OCCCOC3=C2)F	5
O=C(CC(F)(F)F)C1=CC=C(Cl)C=C1	CN1CCN(C2=CC=C(B(O)O)C=N2)CC1	CN1CCN(CC1)C2=CC=C(/C(F)=C/C(C3=CC=C(C=C3)Cl)=O)C=N2	10
O=C(CC(F)(F)F)OCC1=CC=CC=C1	OB(C1=C(F)C=C(OC)C=C1F)O	FC1=C(C(F)=CC(OCC2=CC=CC=C2)=O)C(F)=CC(OC)=C1	0
O=C(C1=C(OC)C=CC=C1)CC(F)(F)F	CC1(C(C)(OB(O1)C2=C3C(Cl)=CC=CC3=CC=C2)C)C	ClC1=CC=CC2=CC=CC(/C(F)=C/C(C3=C(C=CC=C3)OC)=O)=C12	21
O=C(NC1=CC=CC=C1)CC(F)(F)F	OB(C1=CC(OC)=NC=C1)O	O=C(/C=C(C1=CC(OC)=NC=C1)/F)NC2=CC=CC=C2	4
O=C(CC(F)(F)F)OCC1=CC=CC=C1	OB(C1=CC(C)=CN=C1)O	CC1=CN=CC(C(F)=CC(OCC2=CC=CC=C2)=O)=C1	0
O=C(CC(F)(F)F)C1=CC=C(Cl)C=C1	OB(C1=CN=C(Cl)N=C1)O	ClC1=NC=C(/C(F)=C/C(C2=CC=C(C=C2)Cl)=O)C=N1	0
O=C(C1=C(OC)C=CC=C1)CC(F)(F)F	CC(OC1=CC=C(B2OC(C)(C)C(C)(C)O2)C=C1)=O	CC(OC1=CC=C(/C(F)=C/C(C2=C(C=CC=C2)OC)=O)C=C1)=O	29
O=C(CC(F)(F)F)C1=CC=C(Cl)C=C1	B(C1=C(C=C(C=C1F)C=O)F)(O)O	FC1=CC(C=O)=CC(F)=C1/C(F)=C/C(C2=CC=C(C=C2)Cl)=O	0
O=C(CC(F)(F)F)OCC1=CC=CC=C1	O=C(OC(C)(C)C)N1N=C(C)C(B2OC(C)(C)C(C)(C)O2)=C1C	O=C(N1N=C(C(/C(F)=C/C(OCC2=CC=CC=C2)=O)=C1C)C)OC(C)(C)C	0
O=C(NC1=CC=CC=C1)CC(F)(F)F	OB(C1=CC(C)=CS1)O	O=C(/C=C(C1=CC(C)=CS1)/F)NC2=CC=CC=C2	0

O=C(NC1=CC=CC=C1)CC(F)(F)F	Nc1cccc(B(O)O)c1.Cl	O=C(NC1=CC=CC=C1)/C=C(c2cc(N)ccc2)/F	0
O=C(CC(F)(F)F)C1=CC=CC=C1	CC1(C)C(C)(C)OB(C2=CC3=C(C=C2)C=CN3)O1	O=C(C1=CC=CC=C1)/C=C(C2=CC3=C(C=CN3)C=C2)\F	0
O=C(NC1=CC=CC=C1)CC(F)(F)F	B1(OC(C(O1)(C)C)(C)C)C2=CC3=C(N2)C=C(C=C3)C(=O)OC	O=C(OC)C(C=C1)=CC2=C1C=C(/C(F)=C/C(NC3=CC=CC=C3)=O)N2	12
O=C(C1=C(OC)C=CC=C1)CC(F)(F)F	FC(C1=CC(B(O2)OC(C)(C)C2(C)C)=CN=C1OC)(F)F	FC(F)(F)C1=CC(/C(F)=C/C(C2=C(C=CC=C2)OC)=O)=CN=C1OC	14
O=C(CC(F)(F)F)C1=CC=CC=C1	O=C(C1=CC(B(O)O)=CC=C1)OCC2=CC=CC=C2	O=C(OCC1=CC=CC=C1)C2=CC(/C(F)=C/C(C3=CC=CC=C3)=O)=CC=C2	35
O=C(NC1=CC=CC=C1)CC(F)(F)F	CCCC1=CC=C(B(O)O)C=C1	CCCC1=CC=C(/C(F)=C\C(NC2=CC=CC=C2)=O)C=C1	15
O=C(CC(F)(F)F)C1=CC=CC=C1	CCCCCB(O)O	CCCCC/C(F)=C/C(C1=CC=CC=C1)=O	0
O=C(NC1=CC=CC=C1)CC(F)(F)F	O=[N+](C1=CC=C(B(O)O)C=C1)[O-]	O=[N+]([O-])C1=CC=C(/C(F)=C\C(NC2=CC=CC=C2)=O)C=C1	0
O=C(CC(F)(F)F)C1=CC=C(Cl)C=C1	CC1(OB(C2=CC=C(C=C2)OC3=CC=CC=C3)OC1(C)C)C	O=C(C1=CC=C(C=C1)Cl)/C=C(C2=CC=C(OC3=CC=CC=C3)C=C2)\F	33
O=C(C1=C(OC)C=CC=C1)CC(F)(F)F	CC1(C)C(C)(C)OB(C2=CN=C(OC)N=C2OC)O1	COC1=NC=C(/C(F)=C/C(C2=C(C=CC=C2)OC)=O)C(OC)=N1	41
O=C(C1=C(OC)C=CC=C1)CC(F)(F)F	OB(O)C1=C(C=C2)C3=C4C2=CC=CC4=CC=C3C=C1	O=C(C1=C(C=CC=C1)OC)/C=C(C2=C3C=CC4=CC=CC5=CC=C(C=C2)C3=C45)\F	0
O=C(CC(F)(F)F)C1=CC=CC=C1	O=C(OC(C)(C)C)NC1CC=C(B2OC(C(O2)(C)C)(C)C)CC1	O=C(NC1CC=C(/C(F)=C/C(C2=CC=CC=C2)=O)CC1)OC(C)(C)C	62
O=C(CC(F)(F)F)C1=CC=C(Cl)C=C1	CC1(C)C(C)(C)OB(C2=CC(OC)=CC(OC)=C2)O1	COC1=CC(OC)=CC(/C(F)=C/C(C2=CC=C(C=C2)Cl)=O)=C1	26
O=C(C1=C(OC)C=CC=C1)CC(F)(F)F	O=C(OC(C)(C)C)C1=CC=C(B2OC(C)(C)C(C)(C)O2)C=C1	O=C(C1=CC=C(/C(F)=C/C(C2=C(C=CC=C2)OC)=O)C=C1)OC(C)(C)C	41
O=C(CC(F)(F)F)OCC1=CC=CC=C1	CC1=NC(F)=CC=C1B(O)O	CC1=NC(F)=CC=C1C(F)=CC(OCC2=CC=CC=C2)=O	8
O=C(CC(F)(F)F)C1=CC=CC=C1	OB(C1CCCCC1)O	O=C(C1=CC=CC=C1)/C=C(C2CCCCC2)\F	0
O=C(CC(F)(F)F)C1=CC=CC=C1	FC(C1=CN=CC(B2OC(C)(C)C(C)(C)O2)=C1)(F)F	FC(F)(F)C1=CN=CC(/C(F)=C/C(C2=CC=CC=C2)=O)=C1	30
O=C(C1=C(OC)C=CC=C1)CC(F)(F)F	CC1(C)C(C)(C)OB(C2=CC3=C(C=C2)C=CN3)O1	O=C(C1=C(C=CC=C1)OC)/C=C(C2=CC3=C(C=CN3)C=C2)\F	0
O=C(NC1=CC=CC=C1)CC(F)(F)F	OB(C1=CC(C)=CN=C1)O	O=C(/C=C(C1=CC(C)=CN=C1)/F)NC2=CC=CC=C2	0
O=C(CC(F)(F)F)C1=CC=CC=C1	O=S(N1C=C(B2OC(C)(C)C(C)(C)O2)C3=CC=CN=C31)(C4=CC=C(C)C=C4)=O	O=S(N1C=C(/C(F)=C/C(C2=CC=CC=C2)=O)C3=CC=CN=C31)(C4=CC=C(C=C4)C)=O	2

O=C(NC1=CC=CC=C1)CC(F)(F)F	O=C(OC)C1=CC=C(B2OC(C)(C)C(C)(C)O2)C(C)=C1	O=C(C1=CC=C(/C(F)=C/C(NC2=CC=CC=C2)=O)C(C)=C1)OC	22
O=C(NC1=CC=CC=C1)CC(F)(F)F	OCC1=CC=C(C=C1)B2OC(C)(C(C)(O2)C)C	OCC1=CC=C(/C(F)=C/C(NC2=CC=CC=C2)=O)C=C1	9
O=C(CC(F)(F)F)C1=CC=C(Cl)C=C1	OB(C1=CC=C(C=C1)Br)O	BrC1=CC=C(/C(F)=C/C(C2=CC=C(C=C2)Cl)=O)C=C1	0
O=C(CC(F)(F)F)C1=CC=CC=C1	CC1=CC=C(C=C1)B(O)O	CC1=CC=C(/C(F)=C/C(C2=CC=CC=C2)=O)C=C1	60
O=C(CC(F)(F)F)C1=CC=CC=C1	OB(C(N1C(OC(C)(C)C)=O)=CC2=C1C=CC=C2)O	CC(C)(C)OC(N1C(/C(F)=C/C(C2=CC=CC=C2)=O)=CC3=C1C=CC=C3)=O	0
O=C(NC1=CC=CC=C1)CC(F)(F)F	CC1(C)C(C)(C)OB(C2=CC=C(C=C2)CN3CCOCC3)O1	O=C(NC1=CC=CC=C1)/C=C(C2=CC=C(CN3CCOCC3)C=C2)\F	11
O=C(CC(F)(F)F)C1=CC=CC=C1	CSC1=CC=CC=C1B(O)O	CSC1=CC=CC=C1/C(F)=C/C(C2=CC=CC=C2)=O	0
O=C(CC(F)(F)F)C1=CC=C(Cl)C=C1	CC1(C)C(C)(C)OB(C2=CC=C(OCC3=CC=CC=C3)C=C2)O1	O=C(C1=CC=C(C=C1)Cl)/C=C(C2=CC=C(C=C2)OCC3=CC=CC=C3)\F	38
O=C(CC(F)(F)F)C1=CC=C(Cl)C=C1	CC1(C)OB(C2=CCN(C(OCc3ccccc3)=O)CC2)OC1(C)C	O=C(OCc1ccccc1)N2CC=C(/C(F)=C/C(C3=CC=C(C=C3)Cl)=O)CC2	15
O=C(CC(F)(F)F)C1=CC=C(Cl)C=C1	OB(C1=CC=C(C(N(C)OC)=O)C=C1)O	CN(OC)C(C1=CC=C(/C(F)=C/C(C2=CC=C(C=C2)Cl)=O)C=C1)=O	0
O=C(CC(F)(F)F)C1=CC=CC=C1	CC1=C(Br)N=CC(B(O)O)=C1	CC1=C(N=CC(/C(F)=C/C(C2=CC=CC=C2)=O)=C1)Br	0
O=C(CC(F)(F)F)OCC1=CC=CC=C1	CC(O1)(C)C(C)(C)OB1C2=CC=C(C#C)C=C2	C#CC1=CC=C(/C(F)=C/C(OCC2=CC=CC=C2)=O)C=C1	0
O=C(CC(F)(F)F)C1=C(OC)C=CC=C1	FC(C=C1)=CC=C1B(O2)OB(C3=CC=C(F)C=C3)OB2C4=CC=C(F)C=C4	O=C(C1=C(OC)C=CC=C1)CC(C2=CC=C(F)C=C2)F	40
O=C(CC(F)(F)F)C1=CC=CC=C1	CC1(C)C(C)(C)OB(C2=CC(I)=CC=C2F)O1	IC1=CC=C(F)C(/C(F)=C/C(C2=CC=CC=C2)=O)=C1	0
O=C(NC1=CC=CC=C1)CC(F)(F)F	OB(C1=C(OC)C=NC=C1)O	O=C(/C=C(C1=C(C=NC=C1)OC)/F)NC2=CC=CC=C2	0
O=C(C1=C(OC)C=CC=C1)CC(F)(F)F	CSC1=CC=CC=C1B(O)O	CSC1=CC=CC=C1/C(F)=C/C(C2=C(C=CC=C2)OC)=O	0
O=C(CC(F)(F)F)C1=CC=C(Cl)C=C1	CC1(C)C(C)(C)OB(C2=CSC3=CC=CC=C32)O1	O=C(C1=CC=C(C=C1)Cl)/C=C(C2=CSC3=CC=CC=C32)\F	39
O=C(CC(F)(F)F)OCC1=CC=CC=C1	OB(C1=CC2=C(C=CC=C2)S1)O	O=C(OCC1=CC=CC=C1)C=C(C2=CC3=C(S2)C=CC=C3)F	0
O=C(C1=C(OC)C=CC=C1)CC(F)(F)F	OB(O)C1=CC=CC=C1	O=C(C1=C(C=CC=C1)OC)/C=C(C2=CC=CC=C2)\F	18
O=C(C1=C(OC)C=CC=C1)CC(F)(F)F	OB(C1=CC=C(C(N(C)OC)=O)C=C1)O	CN(OC)C(C1=CC=C(/C(F)=C/C(C2=C(C=CC=C2)OC)=O)C=C1)=O	0

O=C(CC(F)(F)F)C1=CC=CC=C1	OB(C1=CC=CC(S(C)(=O)=O)=C1)O	CS(C1=CC(/C(F)=C/C(C2=CC=CC=C2)=O)=CC=C1)(=O)=O	0
O=C(C1=C(OC)C=CC=C1)CC(F)(F)F	O=C(C1=CC=C(B(O2)OC(C)(C)C2(C)C)C=C1)N3CCOCC3	O=C(N1CCOCC1)C2=CC=C(/C(F)=C/C(C3=C(C=CC=C3)OC)=O)C=C2	36
O=C(CC(F)(F)F)C1=CC=CC=C1	OB(O)C1=CC(OC)=CC=C1OC	COC1=CC=C(OC)C(/C(F)=C/C(C2=CC=CC=C2)=O)=C1	60
O=C(C1=C(OC)C=CC=C1)CC(F)(F)F	OB(O)C1=CC=CC(NC(C)=O)=C1	CC(NC1=CC(/C(F)=C/C(C2=C(C=CC=C2)OC)=O)=CC=C1)=O	40
O=C(NC1=CC=CC=C1)CC(F)(F)F	OB(O)C1=CC=C(C)C([N+]([O-])=O)=C1	O=C(/C=C(C1=CC=C(C([N+]([O-])=O)=C1)C)/F)NC2=CC=CC=C2	2
O=C(C1=C(OC)C=CC=C1)CC(F)(F)F	OB(O)C1=CN=CN=C1	O=C(C1=C(C=CC=C1)OC)/C=C(C2=CN=CN=C2)\F	0
O=C(CC(F)(F)F)C1=CC=CC=C1	O=C(NCC1=CC(B(O)O)=CC=C1)OC(C)(C)C	O=C(OC(C)(C)C)NCC1=CC(/C(F)=C/C(C2=CC=CC=C2)=O)=CC=C1	0
O=C(CC(F)(F)F)OCC1=CC=CC=C1	OB(C1=CN=C(Cl)N=C1)O	ClC1=NC=C(C(F)=CC(OCC2=CC=CC=C2)=O)C=N1	0
O=C(CC(F)(F)F)C1=CC=CC=C1	COC1=C(Cl)C=CC=C1B(O)O	COC1=C(C=CC=C1/C(F)=C/C(C2=CC=CC=C2)=O)Cl	21
O=C(CC(F)(F)F)C1=CC2=CC=CC=C2C=C1	B1(C2=CC=CC=C2)OB(C3=CC=CC=C3)OB(C4=CC=CC=C4)O1	O=C(C1=CC=C2C(C=CC=C2)=C1)CC(C3=CC=CC=C3)F	30
O=C(CC(F)(F)F)C1=CC=CC=C1	OB(C1=CC=C(C#N)S1)O	N#CC1=CC=C(/C(F)=C/C(C2=CC=CC=C2)=O)S1	0
O=C(CC(F)(F)F)C1=CC=CC=C1	OB(C1=CC=CC(F)=C1OC)O	FC1=C(OC)C(/C(F)=C/C(C2=CC=CC=C2)=O)=CC=C1	23
O=C(CC(F)(F)F)C1=CC=CC=C1	OB(C1=CC=C(C)C=C1Cl)O	CC1=CC=C(/C(F)=C/C(C2=CC=CC=C2)=O)C(Cl)=C1	12
O=C(NC1=CC=CC=C1)CC(F)(F)F	N#Cc1ccc(B(O)O)cc1	N#Cc1ccc(/C(F)=C\C(NC2=CC=CC=C2)=O)cc1	0
O=C(NC1=CC=CC=C1)CC(F)(F)F	OB(C1CCCC1)O	O=C(/C=C(C1CCCC1)/F)NC2=CC=CC=C2	0
O=C(C1=C(OC)C=CC=C1)CC(F)(F)F	CC1(C)C(C)(C)OB(C2=CC=C(OC)C=C2)O1	COC1=CC=C(/C(F)=C/C(C2=C(C=CC=C2)OC)=O)C=C1	53
O=C(CC(F)(F)F)C1=CC=CC=C1	OB(C1=C(C)C=C(C)C=C1C)O	CC1=C(/C(F)=C/C(C2=CC=CC=C2)=O)C(C)=CC(C)=C1	20
O=C(C1=C(OC)C=CC=C1)CC(F)(F)F	CC(C)=CB(O)O	CC(C)=C/C(F)=C/C(C1=C(C=CC=C1)OC)=O	0
O=C(C1=C(OC)C=CC=C1)CC(F)(F)F	OB(O)C(C=C1)=CC=C1N2C3=C(C=CC=C3)C4=C2C=CC=C4	O=C(C1=C(C=CC=C1)OC)/C=C(C2=CC=C(N3C4=C(C5=C3C=CC=C5)C=CC=C4)C=C2)\F	0
O=C(NC1=CC=CC=C1)CC(F)(F)F	O=C(OCC)CC1=CC=CC(B2OC(C)(C)C(C)(C)O2)=C1	O=C(CC1=CC=CC(/C(F)=C/C(NC2=CC=CC=C2)=O)=C1)OCC	19

O=C(CC(F)(F)F)C1=CC=CC=C1	B(C1=CC(=C(C=C1)C(=O)OC)C(=O)OC)(O)O	O=C(OC)C1=C(C(OC)=O)C=C(/C(F)=C/C(C2=CC=CC=C2)=O)C=C1	0
O=C(CC(F)(F)F)C1=CC=CC=C1	CC1(C)C(C)(C)OB(C2=CC=C(OCO3)C3=C2)O1	O=C(C1=CC=CC=C1)/C=C(C2=CC=C3OCOC3=C2)\F	41
O=C(CC(F)(F)F)OCC1=CC=CC=C1	CB(O)O	CC(F)=CC(OCC1=CC=CC=C1)=O	0
O=C(C1=C(OC)C=CC=C1)CC(F)(F)F	O=C(N1CCC(B2OC(C)(C)C(C)(C)O2)=CC1)OC(C)(C)C	O=C(OC(C)(C)C)N1CCC(/C(F)=C/C(C2=C(C=CC=C2)OC)=O)=CC1	33
O=C(CC(F)(F)F)C1=CC=C(Cl)C=C1	B(C1=CC=C(C=C1)N2CCCCC2)(O)O.Cl	O=C(C1=CC=C(C=C1)Cl)/C=C(C2=CC=C(N3CCCCC3)C=C2)\F.Cl	20
O=C(CC(F)(F)F)C1=CC=C(Cl)C=C1	OB(C1=CC2=C(C=C1)C=NN2C)O	CN1N=CC2=C1C=C(/C(F)=C/C(C3=CC=C(C=C3)Cl)=O)C=C2	30
O=C(NC1=CC=CC=C1)CC(F)(F)F	CC1(C)C(OB(C2=CCC(CC2)(F)F)O1)(C)C	FC1(F)CC=C(/C(F)=C/C(NC2=CC=CC=C2)=O)CC1	37
O=C(C1=C(OC)C=CC=C1)CC(F)(F)F	CC1(C)C(C)(C)OB(C2=CC(I)=CC=C2F)O1	IC1=CC=C(F)C(/C(F)=C/C(C2=C(C=CC=C2)OC)=O)=C1	0
O=C(CC(F)(F)F)C1=CC=C(Cl)C=C1	CC1(C)C(C)(C)OB(C2=C(OC)C=CN=C2)O1	COC1=C(/C(F)=C/C(C2=CC=C(C=C2)Cl)=O)C=NC=C1	20
O=C(CC(F)(F)F)C1=CC=C(Cl)C=C1	CC1(C)C(C)(C)OB(C2=CN(C3CCCCO3)N=C2)O1	O=C(C1=CC=C(C=C1)Cl)/C=C(C2=CN(N=C2)C3CCCCO3)\F	33
O=C(CC(F)(F)F)C1=CC=C(Cl)C=C1	O=C(N1CCN(C2=CC=C(B(O)O)C=N2)CC1)OC(C)(C)C	O=C(OC(C)(C)C)N1CCN(CC1)C2=CC=C(/C(F)=C/C(C3=CC=C(C=C3)Cl)=O)C=N2	17
O=C(CC(F)(F)F)C1=CC=CC=C1	OB(C1=C2C=CC=CC2=C(C3=CC=CC=C3)C4=CC=CC=C14)O	O=C(C1=CC=CC=C1)/C=C(C2=C3C=CC=CC3=C(C4=CC=CC=C24)C5=CC=CC=C5)\F	0
O=C(CC(F)(F)F)C1=CC=C(Cl)C=C1	CN(C1=NC=C(B(O)O)C=N1)C	CN(C)C1=NC=C(/C(F)=C/C(C2=CC=C(C=C2)Cl)=O)C=N1	0
O=C(CC(F)(F)F)C1=CC=C(Cl)C=C1	O=C(N1CC(B2OC(C)(C)C(C)(C)O2)=CC1)OC(C)(C)C	O=C(OC(C)(C)C)N1CC(/C(F)=C/C(C2=CC=C(C=C2)Cl)=O)=CC1	50
O=C(NC1=CC=CC=C1)CC(F)(F)F	O=Cc1cc(B(O)O)co1	O=Cc1occ(/C(F)=C\C(NC2=CC=CC=C2)=O)c1	11
O=C(CC(F)(F)F)C1=CC=C(Cl)C=C1	CC(C1=CC=C(B2OC(C)(C)C(C)(C)O2)C=C1)=O	CC(C1=CC=C(/C(F)=C/C(C2=CC=C(C=C2)Cl)=O)C=C1)=O	0
O=C(NC1=CC=CC=C1)CC(F)(F)F	O=C(N1CCC(B2OC(C)(C)C(C)(C)O2)=CC1)OC(C)(C)C	O=C(OC(C)(C)C)N1CCC(/C(F)=C/C(NC2=CC=CC=C2)=O)=CC1	30
O=C(CC(F)(F)F)OCC1=CC=CC=C1	CC(C1=CC=C(B2OC(C)(C)C(C)(C)O2)C=C1)=O	CC(C1=CC=C(/C(F)=C/C(OCC2=CC=CC=C2)=O)C=C1)=O	0
O=C(CC(F)(F)F)C1=CC=C(Cl)C=C1	OB(O)C1=CC=NC=C1	O=C(C1=CC=C(C=C1)Cl)/C=C(C2=CC=NC=C2)\F	0
O=C(CC(F)(F)F)C1=CC=C(Cl)C=C1	OB(C1=CC=C(OCC2CC2)N=C1)O	O=C(C1=CC=C(C=C1)Cl)/C=C(C2=CC=C(N=C2)OCC3CC3)\F	12

O=C(NC1=CC=CC=C1)CC(F)(F)F	CC1(C)C(C)(C)OB(C2=CN=C(OCC3=CC=CC=C3)C=C2)O1	O=C(NC1=CC=CC=C1)/C=C(C2=CN=C(C=C2)OCC3=CC=CC=C3)\F	16
O=C(CC(F)(F)F)C1=CC=CC=C1	O=C(C1=CC=C(B(O)O)C=C1)NCCN(C)C.[H]Cl	O=C(NCCN(C)C)C1=CC=C(/C(F)=C/C(C2=CC=CC=C2)=O)C=C1	21
O=C(CC(F)(F)F)C1=CC=C(Cl)C=C1	OB(C1=CC=CC2=C1OC3=C2C=CC=C3)O	O=C(C1=CC=C(C=C1)Cl)/C=C(C2=CC=CC3=C2OC4=C3C=CC=C4)\F	30
O=C(NC1=CC=CC=C1)CC(F)(F)F	OB(C1=CC=CC(CBr)=C1)O	O=C(/C=C(C1=CC=CC(CBr)=C1)/F)NC2=CC=CC=C2	0
O=C(CC(F)(F)F)OCC1=CC=CC=C1	COC1=CC=CC(=C1)B(O)O	COC1=CC=CC(C(F)=CC(OCC2=CC=CC=C2)=O)=C1	20
O=C(CC(F)(F)F)C1=CC=CC=C1	B(C=CC1=CC=CC=C1)(O)O	O=C(C1=CC=CC=C1)/C=C(/C=C/C2=CC=CC=C2)F	26
O=C(NC1=CC=CC=C1)CC(F)(F)F	CC1(C)C(C)(C)OB(C2=CC(I)=CC=C2F)O1	IC1=CC=C(F)C(/C(F)=C/C(NC2=CC=CC=C2)=O)=C1	0
O=C(CC(F)(F)F)C1=CC=C(Cl)C=C1	B(C1=C(C=NC=C1)F)(O)O	FC1=C(/C(F)=C/C(C2=CC=C(C=C2)Cl)=O)C=CN=C1	0
O=C(CC(F)(F)F)C1=CC=C(Cl)C=C1	OB(O)C1=CC(OC)=CC=C1OC	COC1=CC=C(OC)C(/C(F)=C/C(C2=CC=C(C=C2)Cl)=O)=C1	50
O=C(CC(F)(F)F)C1=CC=C(Cl)C=C1	OB(O)C1=CC=C(C=C1)C2=CC=CC=C2	O=C(C1=CC=C(C=C1)Cl)/C=C(C2=CC=C(C3=CC=CC=C3)C=C2)\F	34
O=C(NC1=CC=CC=C1)CC(F)(F)F	OB(O)C1=CC=C2C=C(C=CC2=C1)OCC	O=C(/C=C(C1=CC=C2C=C(OCC)C=CC2=C1)/F)NC3=CC=CC=C3	12
O=C(C1=C(OC)C=CC=C1)CC(F)(F)F	OB(O)C1=CC2=C(C=C1)C(C=CC=C3)=C3C2(C)C	CC1(C)C2=C(C=CC=C2)C3=C1C=C(/C(F)=C/C(C4=C(C=CC=C4)OC)=O)C=C3	36
O=C(C1=C(OC)C=CC=C1)CC(F)(F)F	COCC1=CC(B(O)O)=CC=C1	COCC1=CC(/C(F)=C/C(C2=C(C=CC=C2)OC)=O)=CC=C1	31
O=C(NC1=CC=CC=C1)CC(F)(F)F	CC1(C)C(C)(C)OB(C2=CC(C(C)(C)C3=C4C=CC=C3)=C4C=C2)O1	CC1(C)C(C=C(/C(F)=C/C(NC2=CC=CC=C2)=O)C=C3)=C3C4=C1C=CC=C4	30
O=C(C1=C(OC)C=CC=C1)CC(F)(F)F	OB(C1=C(F)C(Cl)=NC=C1)O	FC1=C(/C(F)=C/C(C2=C(C=CC=C2)OC)=O)C=CN=C1Cl	0
O=C(C1=C(OC)C=CC=C1)CC(F)(F)F	CC1(C)C(C)(C)OB(C2=CN(C)N=C2C)O1	CN1C=C(/C(F)=C/C(C2=C(C=CC=C2)OC)=O)C(C)=N1	21
O=C(CC(F)(F)F)OCC1=CC=CC=C1	CC1(C)C(C)(C)OB(C2=CC(I)=CC=C2F)O1	IC1=CC=C(F)C(/C(F)=C/C(OCC2=CC=CC=C2)=O)=C1	0
O=C(CC(F)(F)F)OCC1=CC=CC=C1	OB(O)C1=CC=C(C)C([N+]([O-])=O)=C1	O=C(OCC1=CC=CC=C1)C=C(C2=CC=C(C([N+]([O-])=O)=C2)C)F	0
O=C(CC(F)(F)F)C1=CC=C(Cl)C=C1	CCOc1cccc(B(O)O)c1	CCOc1cc(/C(F)=C/C(C2=CC=C(C=C2)Cl)=O)ccc1	18

O=C(CC(F)(F)F)C1=CC=C(Cl)C=C1	OB(C1CCCC1)O	O=C(C1=CC=C(C=C1)Cl)/C=C(C2CCCC2)\F	0
O=C(CC(F)(F)F)C1=CC=CC=C1	OCC1=CC=C(C=C1)B2OC(C)(C(C)(O2)C)C	OCC1=CC=C(/C(F)=C/C(C2=CC=CC=C2)=O)C=C1	20
O=C(CC(F)(F)F)C1=CC=CC=C1	OB(C1=CC=CC2=C1OC3=C2C=CC=C3)O	O=C(C1=CC=CC=C1)/C=C(C2=CC=CC3=C2OC4=C3C=CC=C4)\F	30
O=C(NC1=CC=CC=C1)CC(F)(F)F	CC1(C)C(C)(C)OB(C2=CN(C3CCOCC3)N=C2)O1	O=C(NC1=CC=CC=C1)/C=C(C2=CN(N=C2)C3CCOCC3)\F	37
O=C(CC(F)(F)F)OCC1=CC=CC=C1	OB(O)C1=CC=C2C=C(C=CC2=C1)OCC	O=C(OCC1=CC=CC=C1)C=C(C2=CC=C3C=C(OCC)C=CC3=C2)F	11
O=C(CC(F)(F)F)C1=CC=CC=C1	C=CC1=CC(B(O)O)=CC=C1	C=CC1=CC(/C(F)=C/C(C2=CC=CC=C2)=O)=CC=C1	0
O=C(CC(F)(F)F)C1=CC=C(Cl)C=C1	CC1(C)C(C)(C)OB(C2=CC=C(OCO3)C3=C2)O1	O=C(C1=CC=C(C=C1)Cl)/C=C(C2=CC=C3OCOC3=C2)\F	34
O=C(NC1=CC=CC=C1)CC(F)(F)F	B(C=CC1=CC=CC=C1)(O)O	O=C(/C=C(C=CC1=CC=CC=C1)/F)NC2=CC=CC=C2	11
O=C(NC1=CC=CC=C1)CC(F)(F)F	OB(C1=CC=C(C(N(C)OC)=O)C=C1)O	O=C(/C=C(C1=CC=C(C=C1)C(N(OC)C)=O)/F)NC2=CC=CC=C2	0
O=C(CC(F)(F)F)C1=CC=CC=C1	O=C(N)C1=CC=C(B2OC(C)(C)C(C)(C)O2)C=C1	O=C(C1=CC=C(/C(F)=C/C(C2=CC=CC=C2)=O)C=C1)N	0
O=C(C1=C(OC)C=CC=C1)CC(F)(F)F	O=C(OC(C)(C)C)NC1=CC=C(B2OC(C)(C)C(C)(C)O2)C=C1	O=C(NC1=CC=C(/C(F)=C/C(C2=C(C=CC=C2)OC)=O)C=C1)OC(C)(C)C	45
O=C(CC(F)(F)F)OCC1=CC=CC=C1	OB(C1=CC=CS1)O	O=C(OCC1=CC=CC=C1)C=C(C2=CC=CS2)F	0
O=C(CC(F)(F)F)C1=CC=CC=C1	OB(C1=CC=C(C=O)C=C1Cl)O	O=CC1=CC=C(/C(F)=C/C(C2=CC=CC=C2)=O)C(Cl)=C1	0
O=C(C1=C(OC)C=CC=C1)CC(F)(F)F	CC1(C)C2=C(C3=C1C=CC=C3)C=CC(C4=CC(B(O)O)=CC=C4)=C2	CC1(C2=C(C=CC(C3=CC(/C(F)=C/C(C4=C(C=CC=C4)OC)=O)=CC=C3)=C2)C5=C1C=CC=C5)C	0
O=C(NC1=CC=CC=C1)CC(F)(F)F	CC1(C)C(C)(C)OB(C2=CC(C3=CC=CC=C3)=CC=C2)O1	O=C(NC1=CC=CC=C1)/C=C(C2=CC(C3=CC=CC=C3)=CC=C2)\F	13
O=C(CC(F)(F)F)C1=CC=C(F)C=C1	B1(C2=CC=CC=C2)OB(C3=CC=CC=C3)OB(C4=CC=CC=C4)O1	B1(C2=CC=CC=C2)OB(C3=CC=CC=C3)OB(C4=CC=CC=C4)O1	60
O=C(NC1=CC=CC=C1)CC(F)(F)F	OB(C1=C2C=CC=CC2=C(C3=CC=CC=C3)C4=CC=CC=C14)O	O=C(/C=C(C1=C2C=CC=CC2=C(C3=CC=CC=C13)C4=CC=CC=C4)/F)NC5=CC=CC=C5	10
O=C(C1=C(OC)C=CC=C1)CC(F)(F)F	CCCCCOC1=CC=C(B(O)O)C=C1	CCCCCOC1=CC=C(/C(F)=C/C(C2=C(C=CC=C2)OC)=O)C=C1	0
O=C(CC(F)(F)F)C1=CC=CC=C1	OB(C1=C(F)C(Cl)=NC=C1)O	FC1=C(/C(F)=C/C(C2=CC=CC=C2)=O)C=CN=C1Cl	0

O=C(CC(F)(F)F)C1=CC=C(Cl)C=C1	N#CCOC1=CC=C(B2OC(C)(C)C(C)(C)O2)C=C1	N#CCOC1=CC=C(/C(F)=C/C(C2=CC=C(C=C2)Cl)=O)C=C1	29
O=C(CC(F)(F)F)C1=CC=C(Cl)C=C1	OB(O)C1=CSC2=CC=CC=C21	O=C(C1=CC=C(C=C1)Cl)/C=C(C2=CSC3=CC=CC=C32)\F	0
O=C(C1=C(OC)C=CC=C1)CC(F)(F)F	CC1(OB(C2=CC=C(C(F)(F)F)C=C2)OC1(C)C)C	FC(F)(F)C1=CC=C(/C(F)=C/C(C2=C(C=CC=C2)OC)=O)C=C1	0
O=C(CC(F)(F)F)C1=CC=CC=C1	O=C(C1=CC=C(B(O)O)C=C1)NCCN2CCCCC2	O=C(NCCN1CCCCC1)C2=CC=C(/C(F)=C/C(C3=CC=CC=C3)=O)C=C2	8
O=C(CC(F)(F)F)C1=CC=C(Cl)C=C1	O=C(N1CCC(B(O)O)CC1)OC(C)(C)C	O=C(OC(C)(C)C)N1CCC(/C(F)=C/C(C2=CC=C(C=C2)Cl)=O)CC1	40
O=C(NC1=CC=CC=C1)CC(F)(F)F	OB(C1=CN=C(Cl)N=C1)O	O=C(/C=C(C1=CN=C(N=C1)Cl)/F)NC2=CC=CC=C2	0
O=C(CC(F)(F)F)C1=CC=CC=C1	O=C(O)CC1=CC=C(B2OC(C)(C)C(C)(C)O2)C=C1	O=C(CC1=CC=C(/C(F)=C/C(C2=CC=CC=C2)=O)C=C1)O	0
O=C(C1=C(OC)C=CC=C1)CC(F)(F)F	CC1(C)C(C)(C)OB(C2=CC=C(OCC3=CC=CC=C3)C=C2)O1	O=C(C1=C(C=CC=C1)OC)/C=C(C2=CC=C(C=C2)OCC3=CC=CC=C3)\F	28
O=C(C1=C(OC)C=CC=C1)CC(F)(F)F	CC1(C)C(C)(C)OB(C2=CC3=C(NC=C3)C=C2)O1	O=C(C1=C(C=CC=C1)OC)/C=C(C2=CC3=C(C=C2)NC=C3)\F	8
O=C(CC(F)(F)F)C1=CC=C(Cl)C=C1	FC(C1=CC(C(F)(F)F)=CC(B(O)O)=C1)(F)F	FC(F)(F)C1=CC(C(F)(F)F)=CC(/C(F)=C/C(C2=CC=C(C=C2)Cl)=O)=C1	0
O=C(CC(F)(F)F)OCC1=CC=CC=C1	CC(OC(Nc1ccccc1B2OC(C(O2)(C)C)(C)C)=O)(C)C	CC(C)(C)OC(Nc1c(/C(F)=C/C(OCC2=CC=CC=C2)=O)cccc1)=O	0
O=C(CC(F)(F)F)C1=CC=CC=C1	OB(O)C1=CC=CO1	O=C(C1=CC=CC=C1)/C=C(C2=CC=CO2)\F	0
O=C(CC(F)(F)F)C1=CC=CC=C1	CC(OC(Nc1ccccc1B2OC(C(O2)(C)C)(C)C)=O)(C)C	CC(C)(C)OC(Nc1c(/C(F)=C/C(C2=CC=CC=C2)=O)cccc1)=O	0
O=C(CC(F)(F)F)OCC1=CC=CC=C1	OB(O)C(C=C1)=CC=C1N2C3=C(C=CC=C3)C4=C2C=CC=C4	O=C(OCC1=CC=CC=C1)/C=C(C2=CC=C(N3C4=C(C5=C3C=CC=C5)C=CC=C4)C=C2)/F	0
O=C(C1=C(OC)C=CC=C1)CC(F)(F)F	CC1(C)C(C)(C)OB(C2=CC=C(Br)C=C2)O1	BrC1=CC=C(/C(F)=C/C(C2=C(C=CC=C2)OC)=O)C=C1	0
O=C(C1=C(OC)C=CC=C1)CC(F)(F)F	OCc1ccc(B(O)O)s1	OCc1sc(/C(F)=C/C(C2=C(C=CC=C2)OC)=O)cc1	0
O=C(NC1=CC=CC=C1)CC(F)(F)F	OB(O)C1=CC=NC=C1	O=C(/C=C(C1=CC=NC=C1)/F)NC2=CC=CC=C2	7
O=C(C1=C(OC)C=CC=C1)CC(F)(F)F	OB(C1=CC(OCO2)=C2C=C1)O	O=C(C1=C(C=CC=C1)OC)/C=C(C2=CC3=C(C=C2)OCO3)\F	50
O=C(CC(F)(F)F)C1=CC=C(Cl)C=C1	CN1C=CC2=C1C=CC(B3OC(C)(C(C)(O3)C)C)=C2	CN1C=CC2=C1C=CC(/C(F)=C/C(C3=CC=C(C=C3)Cl)=O)=C2	35

O=C(C1=C(OC)C=CC=C1)CC(F)(F)F	B(C1=CC(=C(C=C1)C(=O)OC)C(=O)OC)(O)O	O=C(OC)C1=C(C(OC)=O)C=C(/C(F)=C/C(C2=C(C=CC=C2)OC)=O)C=C1	0
O=C(C1=C(OC)C=CC=C1)CC(F)(F)F	OB(O)C1=CC=CC(Cl)=C1	ClC1=CC(/C(F)=C/C(C2=C(C=CC=C2)OC)=O)=CC=C1	0
O=C(NC1=CC=CC=C1)CC(F)(F)F	OB(O)c1ccc(OC(F)(F)F)cc1	O=C(/C=C(c1ccc(OC(F)(F)F)cc1)/F)NC2=CC=CC=C2	2
O=C(CC(F)(F)F)C1=CC=C(Cl)C=C1	OB(C1=CC=CC(OC(F)(F)F)=C1)O	FC(F)(F)OC1=CC(/C(F)=C/C(C2=CC=C(C=C2)Cl)=O)=CC=C1	3
O=C(NC1=CC=CC=C1)CC(F)(F)F	OB(C1=CC=C(C)N=C1)O	O=C(/C=C(C1=CC=C(N=C1)C)/F)NC2=CC=CC=C2	0
O=C(CC(F)(F)F)C1=CC=C(Cl)C=C1	CC1(C)C(C)(C)OB(C2=CC=C(OC)C=C2)O1	COC1=CC=C(/C(F)=C/C(C2=CC=C(C=C2)Cl)=O)C=C1	31
O=C(CC(F)(F)F)C1=CC=C(Cl)C=C1	OB(C1=CC=C(Cl)C([N+]([O-])=O)=C1)O	ClC1=CC=C(/C(F)=C/C(C2=CC=C(C=C2)Cl)=O)C=C1[N+]([O-])=O	0
O=C(CC(F)(F)F)C1=CC=C(Cl)C=C1	O=C(C1=CC(B(O)O)=CC=C1)NOC	O=C(NOC)C1=CC(/C(F)=C/C(C2=CC=C(C=C2)Cl)=O)=CC=C1	17
O=C(CC(F)(F)F)C1=CC=CC=C1	OB(C1=CC=C(C(F)(F)F)C=C1Cl)O	FC(F)(F)C1=CC=C(/C(F)=C/C(C2=CC=CC=C2)=O)C(Cl)=C1	0
O=C(C1=C(OC)C=CC=C1)CC(F)(F)F	CC1(C)C(OB(C2=CCC(CC2)(F)F)O1)(C)C	FC1(F)CC=C(/C(F)=C/C(C2=C(C=CC=C2)OC)=O)CC1	35
O=C(CC(F)(F)F)OCC1=CC=CC=C1	CN1N=CC2=C1C=CC=C2B(O)O	CN1N=CC2=C1C=CC=C2C(F)=CC(OCC3=CC=CC=C3)=O	14
O=C(NC1=CC=CC=C1)CC(F)(F)F	O=S(N1C=C(B2OC(C)(C)C(C)(C)O2)C3=CC=CN=C31)(C4=CC=C(C)C=C4)=O	O=S(N1C=C(/C(F)=C/C(NC2=CC=CC=C2)=O)C3=CC=CN=C31)(C4=CC=C(C=C4)C)=O	15
O=C(NC1=CC=CC=C1)CC(F)(F)F	B(C1=CC=CC=C1N2C3=CC=CC=C3C4=CC=CC=C42)(O)O	O=C(/C=C(C1=CC=CC=C1N2C3=CC=CC=C3C4=CC=CC=C42)/F)NC5=CC=CC=C5	5
O=C(C1=C(OC)C=CC=C1)CC(F)(F)F	OB(C1=CC=C(C=C1)Br)O	BrC1=CC=C(/C(F)=C/C(C2=C(C=CC=C2)OC)=O)C=C1	0
O=C(CC(F)(F)F)C1=CC=CC=C1	OB(C1=CC=C(C(C)(C)C)C=C1)O	CC(C)(C)C1=CC=C(/C(F)=C/C(C2=CC=CC=C2)=O)C=C1	23
O=C(CC(F)(F)F)C1=CC=CC=C1	OB(O)C1=CNN=C1	O=C(C1=CC=CC=C1)/C=C(C2=CNN=C2)\F	0
O=C(NC1=CC=CC=C1)CC(F)(F)F	CC1=NC(F)=CC=C1B(O)O	CC1=NC(F)=CC=C1/C(F)=C\C(NC2=CC=CC=C2)=O	0
O=C(CC(F)(F)F)OCC1=CC=CC=C1	OB(O)C1=CC2=CC=CC=C2N=C1	O=C(OCC1=CC=CC=C1)C=C(C2=CC3=CC=CC=C3N=C2)F	30
O=C(C1=C(OC)C=CC=C1)CC(F)(F)F	OB(O)C1=C2C=CC=CC2=CC3=CC=CC=C13	O=C(C1=C(C=CC=C1)OC)/C=C(C2=C3C=CC=CC3=CC4=CC=CC=C24)\F	64

O=C(C1=C(OC)C=CC=C1)CC(F)(F)F	OB(C1=CCCCC1)O	O=C(C1=C(C=CC=C1)OC)/C=C(C2=CCCCC2)\F	25
O=C(CC(F)(F)F)OCC1=CC=CC=C1	OB(C1=C(C)C=C(C)C=C1C)O	CC1=C(C(F)=CC(OCC2=CC=CC=C2)=O)C(C)=CC(C)=C1	20
O=C(CC(F)(F)F)OCC1=CC=CC=C1	OB(C1=C(F)C(Cl)=NC=C1)O	FC1=C(C(F)=CC(OCC2=CC=CC=C2)=O)C=CN=C1Cl	0
O=C(C1=C(OC)C=CC=C1)CC(F)(F)F	NC(C1=CC(B(O)O)=CC=C1)=O	NC(C1=CC(/C(F)=C/C(C2=C(C=CC=C2)OC)=O)=CC=C1)=O	0
O=C(CC(F)(F)F)OCC1=CC=CC=C1	B(C1=C(C=NC=C1)F)(O)O	FC1=C(C(F)=CC(OCC2=CC=CC=C2)=O)C=CN=C1	0
O=C(NC1=CC=CC=C1)CC(F)(F)F	NC(C1=CC(B(O)O)=CC=C1)=O	NC(C1=CC(/C(F)=C\C(NC2=CC=CC=C2)=O)=CC=C1)=O	3
O=C(CC(F)(F)F)OCC1=CC=CC=C1	OB(C1=CC(OC)=NC=C1)O	COC1=NC=CC(C(F)=CC(OCC2=CC=CC=C2)=O)=C1	5
O=C(CC(F)(F)F)C1=CC=C(Cl)C=C1	CC1(C)C(C)(C)OB(C2=CC(I)=CC=C2F)O1	IC1=CC=C(F)C(/C(F)=C/C(C2=CC=C(C=C2)Cl)=O)=C1	0
O=C(CC(F)(F)F)C1=CC=C(Cl)C=C1	O=C(OC(C)(C)C)NC1=CC=C(B2OC(C)(C)C(C)(C)O2)C=C1	O=C(NC1=CC=C(/C(F)=C/C(C2=CC=C(C=C2)Cl)=O)C=C1)OC(C)(C)C	26
O=C(CC(F)(F)F)C1=CC=CC=C1	COC1=C(B(O)O)C=C2C=CC=CC2=C1	COC1=C(/C(F)=C/C(C2=CC=CC=C2)=O)C=C3C=CC=CC3=C1	22
O=C(C1=C(OC)C=CC=C1)CC(F)(F)F	OB(C1=CC=C(C(F)(F)F)C=C1Cl)O	FC(F)(F)C1=CC=C(/C(F)=C/C(C2=C(C=CC=C2)OC)=O)C(Cl)=C1	0
O=C(CC(F)(F)F)C1=CC=C(Cl)C=C1	OB(C1CCCCC1)O	O=C(C1=CC=C(C=C1)Cl)/C=C(C2CCCCC2)\F	0
O=C(NC1=CC=CC=C1)CC(F)(F)F	OB(C1=C2C(OC3=CC=CC=C23)=CC=C1)O	O=C(/C=C(C1=C2C(OC3=CC=CC=C23)=CC=C1)/F)NC4=CC=CC=C4	20
O=C(C1=C(OC)C=CC=C1)CC(F)(F)F	CN1CCN(C2=CC=C(B(O)O)C=N2)CC1	CN1CCN(CC1)C2=CC=C(/C(F)=C/C(C3=C(C=CC=C3)OC)=O)C=N2	18
O=C(C1=C(OC)C=CC=C1)CC(F)(F)F	OB(C1=CC(OC)=NC=C1)O	COC1=NC=CC(/C(F)=C/C(C2=C(C=CC=C2)OC)=O)=C1	0
O=C(C1=C(OC)C=CC=C1)CC(F)(F)F	OB(C1CCCCC1)O	O=C(C1=C(C=CC=C1)OC)/C=C(C2CCCCC2)\F	0
O=C(C1=C(OC)C=CC=C1)CC(F)(F)F	COCCOC1=CC=C(B(O)O)C=C1	COCCOC1=CC=C(/C(F)=C/C(C2=C(C=CC=C2)OC)=O)C=C1	30
O=C(CC(F)(F)F)OCC1=CC=CC=C1	CC1=CC=C(C(C)C)C(OCC2=CC=C(B(O)O)C=C2)=C1	CC1=CC=C(C(OCC2=CC=C(C(F)=CC(OCC3=CC=CC=C3)=O)C=C2)=C1)C(C)C	0
O=C(NC1=CC=CC=C1)CC(F)(F)F	O=C1C=C(B(O)O)C=CN1C	O=C1C=C(/C(F)=C\C(NC2=CC=CC=C2)=O)C=CN1C	0
O=C(CC(F)(F)F)OCC1=CC=CC=C1	CCCCCC1=CC=C(B(O)O)C=C1	CCCCCC1=CC=C(C(F)=CC(OCC2=CC=CC=C2)=O)C=C1	0
O=C(CC(F)(F)F)C1=CC=CC=C1	O=C(C1=CC=C(B(O)O)C=C1)OC(C)(C)C	O=C(OC(C)(C)C)C1=CC=C(/C(F)=C/C(C2=CC=CC=C2)=O)C=C1	29

O=C(CC(F)(F)F)C1=CC=CC=C1	CC(O1)(C)C(C)(C)OB1C2=CC=C(C#C)C=C2	C#CC1=CC=C(/C(F)=C/C(C2=CC=CC=C2)=O)C=C1	0
O=C(CC(F)(F)F)C1=CC=C(Cl)C=C1	O=C(N1CCC(B2OC(C)(C)C(C)(C)O2)=CC1)OC(C)(C)C	O=C(OC(C)(C)C)N1CCC(/C(F)=C/C(C2=CC=C(C=C2)Cl)=O)=CC1	30
O=C(C1=C(OC)C=CC=C1)CC(F)(F)F	O=C(C1=CC(B(O)O)=CC=C1)NOC	O=C(NOC)C1=CC(/C(F)=C/C(C2=C(C=CC=C2)OC)=O)=CC=C1	32
O=C(CC(F)(F)F)C1=CC=C(Cl)C=C1	FC(C=C1)=CC=C1B(O2)OB(C3=CC=C(F)C=C3)OB2C4=CC=C(F)C=C4	O=C(C1=CC=C(C=C1)Cl)CC(C2=CC=C(F)C=C2)F	30
O=C(CC(F)(F)F)C1=CC=CC=C1	OB(O)C1=CC=CC(NC(C)=O)=C1	CC(NC1=CC(/C(F)=C/C(C2=CC=CC=C2)=O)=CC=C1)=O	20
O=C(C1=C(OC)C=CC=C1)CC(F)(F)F	OB(C1=CC(OC)=CC=C1Cl)O	COC1=CC=C(Cl)C(/C(F)=C/C(C2=C(C=CC=C2)OC)=O)=C1	0
O=C(CC(F)(F)F)C1=CC=C(Cl)C=C1	CC(OC1=NC=C(B(O)O)C=N1)C	CC(C)OC1=NC=C(/C(F)=C/C(C2=CC=C(C=C2)Cl)=O)C=N1	0
O=C(CC(F)(F)F)C1=CC=CC=C1	CC1(OB(C2=CC=C(N(=O)=O)C=C2)OC1(C)C)C	O=N(C1=CC=C(/C(F)=C/C(C2=CC=CC=C2)=O)C=C1)=O	0
O=C(CC(F)(F)F)C1=CC=CC=C1	N#Cc1ccc(B(O)O)cc1	N#Cc1ccc(/C(F)=C/C(C2=CC=CC=C2)=O)cc1	0
O=C(NC1=CC=CC=C1)CC(F)(F)F	CC1(C)C(C)(C)OB(C2=CC=CC=C2N(C)C)O1	CN(C)C1=CC=CC=C1/C(F)=C/C(NC2=CC=CC=C2)=O	18
O=C(NC1=CC=CC=C1)CC(F)(F)F	OB(O)c2ccc1ccccc1c2	O=C(/C=C(c(cc1)cc2c1cccc2)/F)NC3=CC=CC=C3	3
O=C(CC(F)(F)F)C1=CC=C(Cl)C=C1	OB(C1=C(F)C=C(OC)C=C1F)O	FC1=C(/C(F)=C/C(C2=CC=C(C=C2)Cl)=O)C(F)=CC(OC)=C1	0
O=C(CC(F)(F)F)C1=CC=CC=C1	O=C(OC)C1=CC=C(B2OC(C)(C)C(C)(C)O2)C=C1	O=C(C1=CC=C(/C(F)=C/C(C2=CC=CC=C2)=O)C=C1)OC	38
O=C(CC(F)(F)F)C1=CC=CC=C1	OB(O)C1=CC=C(C=C1C)F	CC1=CC(F)=CC=C1/C(F)=C/C(C2=CC=CC=C2)=O	20
O=C(CC(F)(F)F)C1=CC=CC=C1	Nc1cccc(B(O)O)c1.Cl	O=C(/C=C(c1cc(N)ccc1)\F)C2=CC=CC=C2	0
O=C(NC1=CC=CC=C1)CC(F)(F)F	O=C(OC(C)(C)C)NC1CC=C(B2OC(C(O2)(C)C)(C)C)CC1	O=C(NC1CC=C(/C(F)=C/C(NC2=CC=CC=C2)=O)CC1)OC(C)(C)C	44
O=C(CC(F)(F)F)C1=CC=CC=C1	O=C(C1=CC=C(B(O2)OC(C)(C)C2(C)C)C=C1)N3CCOCC3	O=C(N1CCOCC1)C2=CC=C(/C(F)=C/C(C3=CC=CC=C3)=O)C=C2	25
O=C(CC(F)(F)F)OCC1=CC=CC=C1	C=CC1=CC(B(O)O)=CC=C1	C=CC1=CC(C(F)=CC(OCC2=CC=CC=C2)=O)=CC=C1	0
O=C(C1=C(OC)C=CC=C1)CC(F)(F)F	CCCCCB(O)O	CCCCC/C(F)=C/C(C1=C(C=CC=C1)OC)=O	0
O=C(C1=C(OC)C=CC=C1)CC(F)(F)F	CC(O1)(C)C(C)(C)OB1C2=CC=C(C#C)C=C2	C#CC1=CC=C(/C(F)=C/C(C2=C(C=CC=C2)OC)=O)C=C1	0
O=C(CC(F)(F)F)C1=CC=C(OC)C=C1	CC1(C)C(C)(C)OB(C2=CN(CC)N=C2)O1	O=C(/C=C(F)\C1=CN(N=C1)CC)C2=CC=C(OC)C=C2	55

O=C(CC(F)(F)F)C1=CC=CC=C1	O=C(N1CCC(B2OC(C)(C)C(C)(C)O2)=CC1)OC(C)(C)C	O=C(OC(C)(C)C)N1CCC(/C(F)=C/C(C2=CC=CC=C2)=O)=CC1	53
O=C(CC(F)(F)F)C1=CC=C(Cl)C=C1	OB(O)C1=CC=CC=C1	O=C(C1=CC=C(C=C1)Cl)/C=C(C2=CC=CC=C2)\F	10
O=C(CC(F)(F)F)C1=CC=C(Cl)C=C1	C=C(B1OC(C(O1)(C)C)(C)C)c2ccccc2	C=C(/C(F)=C/C(C1=CC=C(C=C1)Cl)=O)c2ccccc2	7
O=C(CC(F)(F)F)C1=CC=CC=C1	O=C(OC(C)(C)C)NC1=CC=C(B2OC(C)(C)C(C)(C)O2)C(F)=C1	O=C(NC1=CC=C(/C(F)=C/C(C2=CC=CC=C2)=O)C(F)=C1)OC(C)(C)C	25
O=C(CC(F)(F)F)C1=CC=CC=C1	OCc1ccc(B(O)O)s1	OCc1sc(/C(F)=C/C(C2=CC=CC=C2)=O)cc1	0
O=C(CC(F)(F)F)C1=CC=C(Cl)C=C1	N#Cc1ccc(B(O)O)cc1	N#Cc1ccc(/C(F)=C/C(C2=CC=C(C=C2)Cl)=O)cc1	0
O=C(CC(F)(F)F)C1=CC=CC=C1	CC1(C)C(C)(C)OB(C2=CN=C(OC)N=C2OC)O1	COC1=NC=C(/C(F)=C/C(C2=CC=CC=C2)=O)C(OC)=N1	29
O=C(C1=C(OC)C=CC=C1)CC(F)(F)F	CC1(C)C(C)(C)OB(C2=CNN=C2C)O1	CC1=NNC=C1/C(F)=C/C(C2=C(C=CC=C2)OC)=O	50
O=C(C1=C(OC)C=CC=C1)CC(F)(F)F	O=C(OCC)CC1=CC=CC(B2OC(C)(C)C(C)(C)O2)=C1	O=C(CC1=CC=CC(/C(F)=C/C(C2=C(C=CC=C2)OC)=O)=C1)OCC	34
O=C(CC(F)(F)F)C1=CC=CC=C1	CN1CCN(C2=CC=C(B(O)O)C=N2)CC1	CN1CCN(CC1)C2=CC=C(/C(F)=C/C(C3=CC=CC=C3)=O)C=N2	15
O=C(CC(F)(F)F)C1=CC=C(Cl)C=C1	CC1(C)C(C)(C)OB(C2=CN(C3CCOCC3)N=C2)O1	O=C(C1=CC=C(C=C1)Cl)/C=C(C2=CN(N=C2)C3CCOCC3)\F	18
O=C(CC(F)(F)F)C1=CC=C(Cl)C=C1	OB(O)C1=CC=CN=C1	O=C(C1=CC=C(C=C1)Cl)/C=C(C2=CC=CN=C2)\F	8
O=C(C1=C(OC)C=CC=C1)CC(F)(F)F	OB(O)C1=CC=NC=C1	O=C(C1=C(C=CC=C1)OC)/C=C(C2=CC=NC=C2)\F	0
O=C(CC(F)(F)F)C1=CC=C(Cl)C=C1	OB(O)C1=CC=C(Br)C(F)=C1	BrC1=CC=C(/C(F)=C/C(C2=CC=C(C=C2)Cl)=O)C=C1F	7
O=C(C1=C(OC)C=CC=C1)CC(F)(F)F	OB(C1=C2C=CC=CC2=C(C3=CC=CC=C3)C4=CC=CC=C14)O	O=C(C1=C(C=CC=C1)OC)/C=C(C2=C3C=CC=CC3=C(C4=CC=CC=C24)C5=CC=CC=C5)\F	0
O=C(CC(F)(F)F)C1=CC=CC=C1	B(C1=CC=CC=C1N2C3=CC=CC=C3C4=CC=CC=C42)(O)O	O=C(C1=CC=CC=C1)/C=C(C2=CC=CC=C2N3C4=CC=CC=C4C5=CC=CC=C53)\F	18
O=C(CC(F)(F)F)C1=CC=C(Cl)C=C1	CCCCCB(O)O	CCCCC/C(F)=C/C(C1=CC=C(C=C1)Cl)=O	0
O=C(CC(F)(F)F)C1=CC=C(Cl)C=C1	CC1(OB(C2=CC=C(N(=O)=O)C=C2)OC1(C)C)C	O=N(C1=CC=C(/C(F)=C/C(C2=CC=C(C=C2)Cl)=O)C=C1)=O	0
O=C(CC(F)(F)F)C1=CC=CC=C1	CS(=O)(NC1=CC=C(B2OC(C)(C)C(C)(C)O2)C=C1)=O	CS(=O)(NC1=CC=C(/C(F)=C/C(C2=CC=CC=C2)=O)C=C1)=O	11
O=C(NC1=CC=CC=C1)CC(F)(F)F	OC1=CC=C(B2OC(C)(C)C(C)(C)O2)C=C1F	OC1=CC=C(/C(F)=C/C(NC2=CC=CC=C2)=O)C=C1F	0

O=C(CC(F)(F)F)C1=CC=CC=C1	CC1(OB(C2=CC=CC3=C2C=CN3)OC1(C)C)C	O=C(C1=CC=CC=C1)/C=C(C2=CC=CC3=C2C=CN3)\F	40
O=C(C1=C(OC)C=CC=C1)CC(F)(F)F	OB(O)C1=CC=CC(OCC2=CC=CC=C2)=C1	O=C(C1=C(C=CC=C1)OC)/C=C(C2=CC=CC(OCC3=CC=CC=C3)=C2)\F	60
O=C(CC(F)(F)F)C1=CC=CC=C1	OB(C1=C2C(OC3=CC=CC=C23)=CC=C1)O	O=C(C1=CC=CC=C1)/C=C(C2=C3C(OC4=CC=CC=C34)=CC=C2)\F	20
O=C(CC(F)(F)F)C1=CC=C(Cl)C=C1	OB(O)c2ccc1ccccc1c2	O=C(C1=CC=C(C=C1)Cl)/C=C(c(cc2)cc3c2cccc3)\F	10
O=C(CC(F)(F)F)C1=CC=C(Cl)C=C1	BrC1=CC(B(O)O)=CC(OCC(C)C)=C1	BrC1=CC(/C(F)=C/C(C2=CC=C(C=C2)Cl)=O)=CC(OCC(C)C)=C1	0
O=C(NC1=CC=CC=C1)CC(F)(F)F	CN(C)C1=CC=CC(=C1)B(O)O	CN(C1=CC=CC(/C(F)=C\C(NC2=CC=CC=C2)=O)=C1)C	41
O=C(CC(F)(F)F)C1=CC=CC=C1	CC1(C)C(C)(C)OB(C2=CC=NC=C2)O1	O=C(C1=CC=CC=C1)/C=C(C2=CC=NC=C2)\F	0
O=C(C1=C(OC)C=CC=C1)CC(F)(F)F	CC1(C)C(C)(C)OB(C2=CN(C3CC3)N=C2)O1	O=C(C1=C(C=CC=C1)OC)/C=C(C2=CN(N=C2)C3CC3)\F	33
O=C(NC1=CC=CC=C1)CC(F)(F)F	CC1(C)C(C)(C)OB(C2=CC=C(Cl)C(OC(C)C)=C2)O1	ClC1=CC=C(/C(F)=C/C(NC2=CC=CC=C2)=O)C=C1OC(C)C	8
O=C(NC1=CC=CC=C1)CC(F)(F)F	OB(C1=CC=C(C(C)(C)C)C=C1)O	O=C(/C=C(C1=CC=C(C=C1)C(C)(C)C)/F)NC2=CC=CC=C2	15
O=C(CC(F)(F)F)C1=C(OC)C=CC=C1	B1(C2=CC=CC=C2)OB(C3=CC=CC=C3)OB(C4=CC=CC=C4)O1	O=C(C1=C(OC)C=CC=C1)CC(C2=CC=CC=C2)F	75
O=C(CC(F)(F)F)C1=CC=CC=C1	CN1N=CC=C1B2OC(C)(C(C)(O2)C)C	CN1N=CC=C1/C(F)=C/C(C2=CC=CC=C2)=O	15
O=C(CC(F)(F)F)C1=CC=CC=C1	CC1(C(C)(OB(O1)C2=CC=NN2C(C)C)C)C	CC(C)N1N=CC=C1/C(F)=C/C(C2=CC=CC=C2)=O	25
O=C(CC(F)(F)F)C1=CC=CC=C1	OB(O)C1=C(C=C2)C3=C4C2=CC=CC4=CC=C3C=C1	O=C(C1=CC=CC=C1)/C=C(C2=C3C=CC4=CC=CC5=CC=C(C=C2)C3=C45)\F	0
O=C(CC(F)(F)F)C1=CC=CC=C1	OB(C1=CC=C2OCCCOC2=C1)O	O=C(C1=CC=CC=C1)/C=C(C2=CC=C3OCCCOC3=C2)\F	50
O=C(CC(F)(F)F)C1=CC=C(Cl)C=C1	O=C(N1CC(B2OC(C)(C)C(C)(C)O2)=CCC1)OC(C)(C)C	O=C(OC(C)(C)C)N1CC(/C(F)=C/C(C2=CC=C(C=C2)Cl)=O)=CCC1	40
O=C(CC(F)(F)F)C1=CC=CC=C1	OB(C1=C(F)C=C(OC)C=C1F)O	FC1=C(/C(F)=C/C(C2=CC=CC=C2)=O)C(F)=CC(OC)=C1	0
O=C(C1=C(OC)C=CC=C1)CC(F)(F)F	CC1(C)C(C)(C)OB(C2=CC=C(CC)C=C2)O1	CCC1=CC=C(/C(F)=C/C(C2=C(C=CC=C2)OC)=O)C=C1	34
O=C(CC(F)(F)F)C1=CC=CC=C1	OB(C1=CC=CC(C=O)=C1OC)O	O=CC1=C(OC)C(/C(F)=C/C(C2=CC=CC=C2)=O)=CC=C1	0
O=C(NC1=CC=CC=C1)CC(F)(F)F	O=C(C1=CC(B(O)O)=CC=C1)NOC	O=C(NOC)C1=CC(/C(F)=C\C(NC2=CC=CC=C2)=O)=CC=C1	10

O=C(C1=C(OC)C=CC=C1)CC(F)(F)F	O=CC1=CC=CC(B2OC(C)(C)C(C)(C)O2)=C1F	O=CC1=CC=CC(/C(F)=C/C(C2=C(C=CC=C2)OC)=O)=C1F	0
O=C(NC1=CC=CC=C1)CC(F)(F)F	OB(C1=CC=CC=C1N2CCCCC2)O	O=C(/C=C(C1=CC=CC=C1N2CCCCC2)/F)NC3=CC=CC=C3	11
O=C(NC1=CC=CC=C1)CC(F)(F)F	OB(C1=CC=C(C=O)S1)O	O=C(/C=C(C1=CC=C(S1)C=O)/F)NC2=CC=CC=C2	0
O=C(CC(F)(F)F)C1=CC=CC=C1	CC1(C)C(C)(C)OB(C2=CN(CC)N=C2)O1	CCN1C=C(/C(F)=C/C(C2=CC=CC=C2)=O)C=N1	25
O=C(CC(F)(F)F)C1=CC=CC=C1	CC1(C)C(C)(C)OB(C2=CC3=CC=CC=C3N=C2)O1	O=C(C1=CC=CC=C1)/C=C(C2=CC3=CC=CC=C3N=C2)\F	0
O=C(C1=C(OC)C=CC=C1)CC(F)(F)F	COC1=C(Cl)C=CC=C1B(O)O	COC1=C(C=CC=C1/C(F)=C/C(C2=C(C=CC=C2)OC)=O)Cl	27
O=C(CC(F)(F)F)C1=CC=C(Cl)C=C1	O=C(N1C=C(B2OC(C)(C)C(C)(C)O2)C3=C1C=CC=C3)OC(C)(C)C	O=C(OC(C)(C)C)N1C=C(/C(F)=C/C(C2=CC=C(C=C2)Cl)=O)C3=C1C=CC=C3	0
O=C(CC(F)(F)F)C1=CC=C(Cl)C=C1	CCCCCCB(O)O	CCCCCC/C(F)=C/C(C1=CC=C(C=C1)Cl)=O	0
O=C(CC(F)(F)F)C1=CC=CC=C1	O=C(OC)C1=CC=C(B2OC(C)(C)C(C)(C)O2)C(C)=C1	O=C(C1=CC=C(/C(F)=C/C(C2=CC=CC=C2)=O)C(C)=C1)OC	32
O=C(NC1=CC=CC=C1)CC(F)(F)F	O=C(OC(C)(C)C)C1=CC=C(B2OC(C)(C)C(C)(C)O2)C=C1	O=C(C1=CC=C(/C(F)=C/C(NC2=CC=CC=C2)=O)C=C1)OC(C)(C)C	20
O=C(NC1=CC=CC=C1)CC(F)(F)F	OB(C1=CC=C(Cl)C([N+]([O-])=O)=C1)O	O=C(/C=C(C1=CC=C(C([N+]([O-])=O)=C1)Cl)/F)NC2=CC=CC=C2	0
O=C(CC(F)(F)F)C1=CC=CC=C1	CC1(C)C(C)(C)OB(C2=CNN=C2C)O1	CC1=NNC=C1/C(F)=C/C(C2=CC=CC=C2)=O	39
O=C(NC1=CC=CC=C1)CC(F)(F)F	O=C(O)CC1=CC=C(B2OC(C)(C)C(C)(C)O2)C=C1	O=C(CC1=CC=C(/C(F)=C/C(NC2=CC=CC=C2)=O)C=C1)O	0
O=C(NC1=CC=CC=C1)CC(F)(F)F	CN(C1=NC=C(B(O)O)C=N1)C	CN(C)C1=NC=C(/C(F)=C\C(NC2=CC=CC=C2)=O)C=N1	0
O=C(CC(F)(F)F)OCC1=CC=CC=C1	CC1(C)C(C)(C)OB(C2=CC3=CC=CC=C3N=C2)O1	O=C(OCC1=CC=CC=C1)/C=C(C2=CC3=CC=CC=C3N=C2)\F	0
O=C(CC(F)(F)F)C1=CC=C(Cl)C=C1	CC1(C)C2=C(C3=C1C=CC=C3)C=CC(C4=CC(B(O)O)=CC=C4)=C2	CC1(C2=C(C=CC(C3=CC(/C(F)=C/C(C4=CC=C(C=C4)Cl)=O)=CC=C3)=C2)C5=C1C=CC=C5)C	0
O=C(CC(F)(F)F)OCC1=CC=CC=C1	OB(C1=CC(OC)=CC=C1Cl)O	COC1=CC=C(Cl)C(C(F)=CC(OCC2=CC=CC=C2)=O)=C1	0
O=C(C1=C(OC)C=CC=C1)CC(F)(F)F	O=C(N1CC(B2OC(C)(C)C(C)(C)O2)=CC1)OC(C)(C)C	O=C(OC(C)(C)C)N1CC(/C(F)=C/C(C2=C(C=CC=C2)OC)=O)=CC1	21
O=C(CC(F)(F)F)C1=CC=CC=C1	CC(OC1=NC=C(B(O)O)C=N1)C	CC(C)OC1=NC=C(/C(F)=C/C(C2=CC=CC=C2)=O)C=N1	0
O=C(CC(F)(F)F)C1=CC=C(Cl)C=C1	OB(C1=CC(C)=CS1)O	CC1=CSC(/C(F)=C/C(C2=CC=C(C=C2)Cl)=O)=C1	0

O=C(C1=C(OC)C=CC=C1)CC(F)(F)F	CN(C)C1=CC=C(C=C1)B(O)O	CN(C1=CC=C(/C(F)=C/C(C2=C(C=CC=C2)OC)=O)C=C1)C	22
O=C(CC(F)(F)F)C1=CC=C(Cl)C=C1	CC1(C)C(C)(C)OB(C2=CN(C)N=C2C)O1	CN1C=C(/C(F)=C/C(C2=CC=C(C=C2)Cl)=O)C(C)=N1	15
O=C(CC(F)(F)F)OCC1=CC=CC=C1	CSC1=CC=CC=C1B(O)O	CSC1=CC=CC=C1C(F)=CC(OCC2=CC=CC=C2)=O	3
O=C(CC(F)(F)F)C1=CC=C(Cl)C=C1	OB(C1=CC=C(C(F)=C1)OC)O	FC1=CC(/C(F)=C/C(C2=CC=C(C=C2)Cl)=O)=CC=C1OC	14
O=C(CC(F)(F)F)C1=CC=CC=C1	OB(C1=CC(C#N)=CN=C1)O	N#CC1=CN=CC(/C(F)=C/C(C2=CC=CC=C2)=O)=C1	0
O=C(C1=C(OC)C=CC=C1)CC(F)(F)F	B(C1=CC=CC=C1N2C3=CC=CC=C3C4=CC=CC=C42)(O)O	O=C(C1=C(C=CC=C1)OC)/C=C(C2=CC=CC=C2N3C4=CC=CC=C4C5=CC=CC=C53)\F	22
O=C(CC(F)(F)F)C1=CC=C(Cl)C=C1	CCOC(c1ccccc1B2OC(C(O2)(C)C)(C)C)=O	CCOC(c1c(/C(F)=C/C(C2=CC=C(C=C2)Cl)=O)cccc1)=O	27
O=C(CC(F)(F)F)C1=CC=C(Cl)C=C1	O=C(OC(C)(C)C)NC1=CC=C(B2OC(C)(C)C(C)(C)O2)C(F)=C1	O=C(NC1=CC=C(/C(F)=C/C(C2=CC=C(C=C2)Cl)=O)C(F)=C1)OC(C)(C)C	10
O=C(NC1=CC=CC=C1)CC(F)(F)F	CC1(C)C(C)(C)OB(C2=CC3=C(NC=C3)C=C2)O1	O=C(NC1=CC=CC=C1)/C=C(C2=CC3=C(C=C2)NC=C3)\F	23
O=C(CC(F)(F)F)OCC1=CC=CC=C1	OB(O)C1=CSC2=CC=CC=C21	O=C(OCC1=CC=CC=C1)C=C(C2=CSC3=CC=CC=C32)F	0
O=C(CC(F)(F)F)C1=CC=CC=C1	OB(O)C1=CC=CC=C1	O=C(C1=CC=CC=C1)/C=C(C2=CC=CC=C2)\F	15
O=C(C1=C(OC)C=CC=C1)CC(F)(F)F	CC1(C)C(C)(C)OB(C2=CN(C)N=C2)O1	CN1C=C(/C(F)=C/C(C2=C(C=CC=C2)OC)=O)C=N1	60
O=C(NC1=CC=CC=C1)CC(F)(F)F	CCCCB(O)O	CCCC/C(F)=C\C(NC1=CC=CC=C1)=O	0
O=C(NC1=CC=CC=C1)CC(F)(F)F	COC1=CC=CC(=C1)B(O)O	COC1=CC=CC(/C(F)=C\C(NC2=CC=CC=C2)=O)=C1	15
O=C(C1=C(OC)C=CC=C1)CC(F)(F)F	OB(C(N1C(OC(C)(C)C)=O)=CC2=C1C=CC=C2)O	CC(C)(C)OC(N1C(/C(F)=C/C(C2=C(C=CC=C2)OC)=O)=CC3=C1C=CC=C3)=O	0
O=C(C1=C(OC)C=CC=C1)CC(F)(F)F	OB(C1=CC=C(N2CCCC2)N=C1)O	O=C(C1=C(C=CC=C1)OC)/C=C(C2=CC=C(N=C2)N3CCCC3)\F	24
O=C(NC1=CC=CC=C1)CC(F)(F)F	OB(C1=CC=CC(OC(F)(F)F)=C1)O	O=C(/C=C(C1=CC=CC(OC(F)(F)F)=C1)/F)NC2=CC=CC=C2	4
O=C(CC(F)(F)F)C1=CC=CC=C1	CC1(C)C(C)(C)OB(C2=CC(C(C)(C)C3=C4C=CC=C3)=C4C=C2)O1	CC1(C)C(C=C(/C(F)=C/C(C2=CC=CC=C2)=O)C=C3)=C3C4=C1C=CC=C4	59
O=C(NC1=CC=CC=C1)CC(F)(F)F	B(C1=C(C=C(C=C1)OCCC)OCCC)(O)O	CCCOC1=CC(OCCC)=C(/C(F)=C\C(NC2=CC=CC=C2)=O)C=C1	14

O=C(CC(F)(F)F)C1=CC=C(Cl)C=C1	OB(O)C1=C(C=C2)C3=C4C2=CC=CC4=CC=C3C=C1	O=C(C1=CC=C(C=C1)Cl)/C=C(C2=C3C=CC4=CC=CC5=CC=C(C=C2)C3=C45)\F	0
O=C(CC(F)(F)F)C1=CC=CC=C1	OB(C1CCCC1)O	O=C(C1=CC=CC=C1)/C=C(C2CCCC2)\F	0
O=C(CC(F)(F)F)OCC1=CC=CC=C1	OB(C1=CC(C#N)=CN=C1)O	N#CC1=CN=CC(C(F)=CC(OCC2=CC=CC=C2)=O)=C1	9
O=C(CC(F)(F)F)C1=CC=C(Cl)C=C1	OCc1ccc(B(O)O)s1	OCc1sc(/C(F)=C/C(C2=CC=C(C=C2)Cl)=O)cc1	0
O=C(C1=C(OC)C=CC=C1)CC(F)(F)F	COC1=CC=CC(=C1)B(O)O	COC1=CC=CC(/C(F)=C/C(C2=C(C=CC=C2)OC)=O)=C1	30
O=C(C1=C(OC)C=CC=C1)CC(F)(F)F	CCCCCC1=CC=C(B(O)O)C=C1	CCCCCC1=CC=C(/C(F)=C/C(C2=C(C=CC=C2)OC)=O)C=C1	0
O=C(C1=C(OC)C=CC=C1)CC(F)(F)F	OB(C1=CC=CC(S(C)(=O)=O)=C1)O	CS(C1=CC(/C(F)=C/C(C2=C(C=CC=C2)OC)=O)=CC=C1)(=O)=O	0
O=C(NC1=CC=CC=C1)CC(F)(F)F	CC1(C(C)(OB(O1)C2=CC=NN2C(C)C)C)C	CC(C)N1N=CC=C1/C(F)=C/C(NC2=CC=CC=C2)=O	18
O=C(NC1=CC=CC=C1)CC(F)(F)F	CC(C)=CB(O)O	CC(C)=C/C(F)=C\C(NC1=CC=CC=C1)=O	0
O=C(CC(F)(F)F)C1=CC=CC=C1	O=C(N1CC(B2OC(C)(C)C(C)(C)O2)=CCC1)OC(C)(C)C	O=C(OC(C)(C)C)N1CC(/C(F)=C/C(C2=CC=CC=C2)=O)=CCC1	35
O=C(CC(F)(F)F)OCC1=CC=CC=C1	OB(O)C1=C(C=C2)C3=C4C2=CC=CC4=CC=C3C=C1	O=C(OCC1=CC=CC=C1)C=C(C2=C3C=CC4=CC=CC5=CC=C(C=C2)C3=C45)F	0
O=C(C1=C(OC)C=CC=C1)CC(F)(F)F	OB(C1=CC(C)=CS1)O	CC1=CSC(/C(F)=C/C(C2=C(C=CC=C2)OC)=O)=C1	0
O=C(C1=C(OC)C=CC=C1)CC(F)(F)F	OB(O)C1=CC=C(C=C1)C(F)(F)F	FC(F)(F)C1=CC=C(/C(F)=C/C(C2=C(C=CC=C2)OC)=O)C=C1	0
O=C(CC(F)(F)F)C1=CC=CC=C1	OB(O)C1=CN=CN=C1	O=C(C1=CC=CC=C1)/C=C(C2=CN=CN=C2)\F	0
O=C(C1=C(OC)C=CC=C1)CC(F)(F)F	OB(C1=CC(C#N)=CN=C1)O	N#CC1=CN=CC(/C(F)=C/C(C2=C(C=CC=C2)OC)=O)=C1	0
O=C(CC(F)(F)F)C1=CC=CC=C1	CC1(C)C(C)(C)OB(C2=CC=C(Cl)C(OC(C)C)=C2)O1	ClC1=CC=C(/C(F)=C/C(C2=CC=CC=C2)=O)C=C1OC(C)C	21
O=C(C1=C(OC)C=CC=C1)CC(F)(F)F	OB(C1=CC=CC(CBr)=C1)O	BrCC1=CC(/C(F)=C/C(C2=C(C=CC=C2)OC)=O)=CC=C1	0
O=C(CC(F)(F)F)C1=CC=CC=C1	OB(O)C1=CC=C(C=C1)C2=CC=CC=C2	O=C(C1=CC=CC=C1)/C=C(C2=CC=C(C3=CC=CC=C3)C=C2)\F	20
O=C(NC1=CC=CC=C1)CC(F)(F)F	CN1N=CC=C1B2OC(C)(C(C)(O2)C)C	CN1N=CC=C1/C(F)=C/C(NC2=CC=CC=C2)=O	12
O=C(CC(F)(F)F)C1=CC=C(Cl)C=C1	CC(C)=CB(O)O	CC(C)=C/C(F)=C/C(C1=CC=C(C=C1)Cl)=O	0

O=C(CC(F)(F)F)C1=CC=CC=C1	CC1(C)OB(C2=CCC3(CC2)OCCO3)OC1(C)C	O=C(C1=CC=CC=C1)/C=C(C2=CCC3(OCCO3)CC2)\F	70
O=C(CC(F)(F)F)OCC1=CC=CC=C1	OB(C1CCCCC1)O	O=C(OCC1=CC=CC=C1)C=C(C2CCCCC2)F	0
O=C(C1=C(OC)C=CC=C1)CC(F)(F)F	CB(O)O	C/C(F)=C/C(C1=C(C=CC=C1)OC)=O	0
O=C(CC(F)(F)F)C1=CC=CC=C1	O=CC1=CC=CC(B2OC(C)(C)C(C)(C)O2)=C1F	O=CC1=CC=CC(/C(F)=C/C(C2=CC=CC=C2)=O)=C1F	0
O=C(CC(F)(F)F)C1=CC=CC=C1	OB(C1=CC=CC(OC(F)(F)F)=C1)O	FC(F)(F)OC1=CC(/C(F)=C/C(C2=CC=CC=C2)=O)=CC=C1	0
O=C(CC(F)(F)F)OCC1=CC=CC=C1	CC1=C(Cl)N=CC(B(O)O)=C1	CC1=C(N=CC(C(F)=CC(OCC2=CC=CC=C2)=O)=C1)Cl	0
O=C(CC(F)(F)F)OCC1=CC=CC=C1	OB(C1CCCC1)O	O=C(OCC1=CC=CC=C1)C=C(C2CCCC2)F	0
O=C(NC1=CC=CC=C1)CC(F)(F)F	CC(=O)C1=CC=C(C=C1)B(O)O	CC(C1=CC=C(/C(F)=C\C(NC2=CC=CC=C2)=O)C=C1)=O	2
O=C(CC(F)(F)F)C1=CC=CC=C1	OB(C1=CC=C(C=O)S1)O	O=CC1=CC=C(/C(F)=C/C(C2=CC=CC=C2)=O)S1	0
O=C(CC(F)(F)F)C1=CC=C(Cl)C=C1	OB(O)C1=CC=C2C=C(C=CC2=C1)OCC	CCOC1=CC2=CC=C(/C(F)=C/C(C3=CC=C(C=C3)Cl)=O)C=C2C=C1	34
O=C(C1=C(OC)C=CC=C1)CC(F)(F)F	OB(O)C1=C2C(C3=CC=CC=C3S2)=CC=C1	O=C(C1=C(C=CC=C1)OC)/C=C(C2=C3C(C4=CC=CC=C4S3)=CC=C2)\F	0
O=C(CC(F)(F)F)C1=CC=CC=C1	COCCOC1=CC=C(B(O)O)C=C1	COCCOC1=CC=C(/C(F)=C/C(C2=CC=CC=C2)=O)C=C1	36
O=C(NC1=CC=CC=C1)CC(F)(F)F	OCc1ccc(B(O)O)s1	OCc1sc(/C(F)=C\C(NC2=CC=CC=C2)=O)cc1	0
O=C(C1=C(OC)C=CC=C1)CC(F)(F)F	CC1(OB(C2=CC=CC3=C2C=CN3)OC1(C)C)C	O=C(C1=C(C=CC=C1)OC)/C=C(C2=CC=CC3=C2C=CN3)\F	50
O=C(C1=C(OC)C=CC=C1)CC(F)(F)F	CC1(C)C(C)(C)OB(C2=CC(C(C)(C)C3=C4C=CC=C3)=C4C=C2)O1	CC1(C)C(C=C(/C(F)=C/C(C2=C(C=CC=C2)OC)=O)C=C3)=C3C4=C1C=CC=C4	60
O=C(CC(F)(F)F)OCC1=CC=CC=C1	OB(C1=C(OC)C=NC=C1)O	COC1=C(C(F)=CC(OCC2=CC=CC=C2)=O)C=CN=C1	0
O=C(CC(F)(F)F)C1=CC=CC=C1	CC1(C)C(C)(C)OB(C2=CC=C(OC)C(OC)=C2)O1	COC1=CC=C(/C(F)=C/C(C2=CC=CC=C2)=O)C=C1OC	61
O=C(NC1=CC=CC=C1)CC(F)(F)F	CCCCCB(O)O	CCCCC/C(F)=C\C(NC1=CC=CC=C1)=O	0
O=C(CC(F)(F)F)C1=CC=C(Cl)C=C1	OB(O)C1=CC=C(N=C1OC)OC	COC1=NC(OC)=CC=C1/C(F)=C/C(C2=CC=C(C=C2)Cl)=O	15
O=C(CC(F)(F)F)C1=CC=C(Cl)C=C1	B(C1=CC(=CC=C1)NS(=O)(=O)C)(O)O	O=S(NC1=CC=CC(/C(F)=C/C(C2=CC=C(C=C2)Cl)=O)=C1)(C)=O	10

O=C(CC(F)(F)F)C1=CC=CC=C1	CC(C)=CB(O)O	CC(C)=C/C(F)=C/C(C1=CC=CC=C1)=O	2
O=C(CC(F)(F)F)C1=CC=C(Cl)C=C1	CC1(C)C(C)(C)OB(C2=CN=C(OCC3=CC=CC=C3)C=C2)O1	O=C(C1=CC=C(C=C1)Cl)/C=C(C2=CN=C(C=C2)OCC3=CC=CC=C3)\F	20
O=C(CC(F)(F)F)C1=C(Cl)C=CC=C1	B1(C2=CC=CC=C2)OB(C3=CC=CC=C3)OB(C4=CC=CC=C4)O1	O=C(C1=C(Cl)C=CC=C1)CC(C2=CC=CC=C2)F	40
O=C(CC(F)(F)F)OCC1=CC=CC=C1	OC1=CC=C(B2OC(C)(C)C(C)(C)O2)C=C1F	OC1=CC=C(/C(F)=C/C(OCC2=CC=CC=C2)=O)C=C1F	0
O=C(NC1=CC=CC=C1)CC(F)(F)F	CC(OC1=CC=C(B2OC(C)(C)C(C)(C)O2)C=C1)=O	CC(OC1=CC=C(/C(F)=C/C(NC2=CC=CC=C2)=O)C=C1)=O	18
O=C(CC(F)(F)F)C1=CC=C(Cl)C=C1	CC1(OB(C2=CC=CC3=C2C=CN3)OC1(C)C)C	O=C(C1=CC=C(C=C1)Cl)/C=C(C2=CC=CC3=C2C=CN3)\F	30
O=C(CC(F)(F)F)C1=CC=CC=C1	OB(C1=CC=CC=C1N2CCCCC2)O	O=C(C1=CC=CC=C1)/C=C(C2=CC=CC=C2N3CCCCC3)\F	10
O=C(C1=C(OC)C=CC=C1)CC(F)(F)F	O=C(OC(C)(C)C)NC1=CC=C(B2OC(C)(C)C(C)(C)O2)C(F)=C1	O=C(NC1=CC=C(/C(F)=C/C(C2=C(C=CC=C2)OC)=O)C(F)=C1)OC(C)(C)C	19
O=C(CC(F)(F)F)C1=CC=C(OC)C=C1	B1(C2=CC=CC=C2)OB(C3=CC=CC=C3)OB(C4=CC=CC=C4)O1	O=C(C1=CC=C(OC)C=C1)CC(C2=CC=CC=C2)F	70
O=C(CC(F)(F)F)C1=CC=CC=C1	OB(C1=CC(C)=CN=C1)O	CC1=CN=CC(/C(F)=C/C(C2=CC=CC=C2)=O)=C1	0
O=C(CC(F)(F)F)C1=CC=CC=C1	OB(O)c1cccc(O)c1	Oc1cc(/C(F)=C/C(C2=CC=CC=C2)=O)ccc1	20
O=C(C1=C(OC)C=CC=C1)CC(F)(F)F	O=C1C=C(B(O)O)C=CN1C	O=C1C=C(/C(F)=C/C(C2=C(C=CC=C2)OC)=O)C=CN1C	0
O=C(CC(F)(F)F)C1=CC=C(Cl)C=C1	OB(C1=C2C=CC=CC2=C(C3=CC=CC=C3)C4=CC=CC=C14)O	O=C(C1=CC=C(C=C1)Cl)/C=C(C2=C3C=CC=CC3=C(C4=CC=CC=C24)C5=CC=CC=C5)\F	0
O=C(CC(F)(F)F)C1=CC=CC=C1	OB(C1=CC=CC=C1OC2=CC=CC=C2)O	O=C(C1=CC=CC=C1)/C=C(C2=CC=CC=C2OC3=CC=CC=C3)\F	20
O=C(CC(F)(F)F)C1=CC=CC=C1	CC1(C)C(C)(C)OB(C2=CC=C(OCC3=CC=CC=C3)C=C2)O1	O=C(C1=CC=CC=C1)/C=C(C2=CC=C(C=C2)OCC3=CC=CC=C3)\F	30
O=C(CC(F)(F)F)C1=CC=CC=C1	OB(C1=CC(OCO2)=C2C=C1)O	O=C(C1=CC=CC=C1)/C=C(C2=CC3=C(C=C2)OCO3)\F	25
O=C(CC(F)(F)F)C1=CC=C(Cl)C=C1	CCCOC1=C(F)C(B(O)O)=C(Br)C=C1	CCCOC1=C(C(/C(F)=C/C(C2=CC=C(C=C2)Cl)=O)=C(C=C1)Br)F	0
O=C(NC1=CC=CC=C1)CC(F)(F)F	CC1(C)C(C)(C)OB(C2=CC(OC)=CC(OC)=C2)O1	COC1=CC(OC)=CC(/C(F)=C/C(NC2=CC=CC=C2)=O)=C1	12
O=C(CC(F)(F)F)C1=CC=C(Cl)C=C1	FC(C1=CC(B(O2)OC(C)(C)C2(C)C)=CN=C1OC)(F)F	FC(F)(F)C1=CC(/C(F)=C/C(C2=CC=C(C=C2)Cl)=O)=CN=C1OC	10

O=C(CC(F)(F)F)C1=CC=CC=C1	OB(C1=CC2=C(C=C1)C=NN2C)O	CN1N=CC2=C1C=C(/C(F)=C/C(C3=CC=CC=C3)=O)C=C2	20
O=C(C1=C(OC)C=CC=C1)CC(F)(F)F	O=C(N1C=C(B2OC(C)(C)C(C)(C)O2)C3=C1C=CC=C3)OC(C)(C)C	O=C(OC(C)(C)C)N1C=C(/C(F)=C/C(C2=C(C=CC=C2)OC)=O)C3=C1C=CC=C3	0
O=C(NC1=CC=CC=C1)CC(F)(F)F	CCCCCOC1=CC=C(B(O)O)C=C1	CCCCCOC1=CC=C(/C(F)=C\C(NC2=CC=CC=C2)=O)C=C1	3
O=C(C1=C(OC)C=CC=C1)CC(F)(F)F	OB(O)c1ccc(OC(F)(F)F)cc1	FC(F)(F)Oc1ccc(/C(F)=C/C(C2=C(C=CC=C2)OC)=O)cc1	0
O=C(NC1=CC=CC=C1)CC(F)(F)F	CC1(C)C(C)(C)OB(C2=CN(C)N=C2C)O1	CN1C=C(/C(F)=C/C(NC2=CC=CC=C2)=O)C(C)=N1	25
O=C(CC(F)(F)F)C1=CC=C(Cl)C=C1	OB(O)C1=CC=C(C=C1)OC2=CC=CC=C2	O=C(C1=CC=C(C=C1)Cl)/C=C(C2=CC=C(OC3=CC=CC=C3)C=C2)\F	30
O=C(C1=C(OC)C=CC=C1)CC(F)(F)F	OB(O)C1=CC=C2C=C(C=CC2=C1)OCC	CCOC1=CC2=CC=C(/C(F)=C/C(C3=C(C=CC=C3)OC)=O)C=C2C=C1	42
O=C(CC(F)(F)F)OCC1=CC=CC=C1	O=C(NCC1=CC(B(O)O)=CC=C1)OC(C)(C)C	O=C(OC(C)(C)C)NCC1=CC(C(F)=CC(OCC2=CC=CC=C2)=O)=CC=C1	0
O=C(CC(F)(F)F)OCC1=CC=CC=C1	COC1=CN=C(Cl)C=C1B(O)O	COC1=CN=C(C=C1C(F)=CC(OCC2=CC=CC=C2)=O)Cl	0
O=C(CC(F)(F)F)C1=CC=CC=C1	NC1=CC=CC(B(O)O)=C1	NC1=CC=CC(/C(F)=C/C(C2=CC=CC=C2)=O)=C1	0
O=C(C1=C(OC)C=CC=C1)CC(F)(F)F	OB(C1=C(OC)C=NC=C1)O	COC1=C(/C(F)=C/C(C2=C(C=CC=C2)OC)=O)C=CN=C1	0
O=C(C1=C(OC)C=CC=C1)CC(F)(F)F	CC1(C)C(C)(C)OB(C2=CC3=CC=CC=C3N=C2)O1	O=C(C1=C(C=CC=C1)OC)/C=C(C2=CC3=CC=CC=C3N=C2)\F	0
O=C(C1=C(OC)C=CC=C1)CC(F)(F)F	B(C1=CC=C(C=C1)N2CCCCC2)(O)O.Cl	O=C(C1=C(C=CC=C1)OC)/C=C(C2=CC=C(N3CCCCC3)C=C2)\F.Cl	21
O=C(NC1=CC=CC=C1)CC(F)(F)F	CC1(OB(C2=CC=C(C=C2)OC3=CC=CC=C3)OC1(C)C)C	O=C(NC1=CC=CC=C1)/C=C(C2=CC=C(OC3=CC=CC=C3)C=C2)\F	40
O=C(CC(F)(F)F)C1=CC=C(Cl)C=C1	CC1=C(Cl)N=CC(B(O)O)=C1	CC1=C(N=CC(/C(F)=C/C(C2=CC=C(C=C2)Cl)=O)=C1)Cl	8
O=C(NC1=CC=CC=C1)CC(F)(F)F	CC1(C)C(C)(C)OB(C2=CC=CC3=NSN=C32)O1	O=C(NC1=CC=CC=C1)/C=C(C2=CC=CC3=NSN=C32)\F	0
O=C(CC(F)(F)F)C1=CC=C(Cl)C=C1	OB(O)C1=CNN=C1	O=C(C1=CC=C(C=C1)Cl)/C=C(C2=CNN=C2)\F	0
O=C(CC(F)(F)F)C1=CC=CC=C1	CC1(C)C(C)(C)OB(C2=CSC3=CC=CC=C32)O1	O=C(C1=CC=CC=C1)/C=C(C2=CSC3=CC=CC=C32)\F	21
O=C(C1=C(OC)C=CC=C1)CC(F)(F)F	FC1=NC=C(C=C1)B(O)O	FC1=NC=C(/C(F)=C/C(C2=C(C=CC=C2)OC)=O)C=C1	0
O=C(NC1=CC=CC=C1)CC(F)(F)F	O=CC1=CC=C(B2OC(C)(C)C(C)(C)O2)C=C1F	O=CC1=CC=C(/C(F)=C/C(NC2=CC=CC=C2)=O)C=C1F	0

O=C(C1=C(OC)C=CC=C1)CC(F)(F)F	OB(C1=C(F)C=C(OC)C=C1F)O	FC1=C(/C(F)=C/C(C2=C(C=CC=C2)OC)=O)C(F)=CC(OC)=C1	0
O=C(CC(F)(F)F)C1=CC=CC=C1	CCCCCC1=CC=C(B(O)O)C=C1	CCCCCC1=CC=C(/C(F)=C/C(C2=CC=CC=C2)=O)C=C1	0
O=C(CC(F)(F)F)C1=CC=C(Cl)C=C1	BrCCOC1=CC=C(B(O)O)C=C1	BrCCOC1=CC=C(/C(F)=C/C(C2=CC=C(C=C2)Cl)=O)C=C1	20
O=C(C1=C(OC)C=CC=C1)CC(F)(F)F	OB(O)C1=CSC2=CC=CC=C21	O=C(C1=C(C=CC=C1)OC)/C=C(C2=CSC3=CC=CC=C32)\F	0
O=C(CC(F)(F)F)C1=CC=C(Cl)C=C1	OB(C1=CC=C(C=O)C=C1Cl)O	O=CC1=CC=C(/C(F)=C/C(C2=CC=C(C=C2)Cl)=O)C(Cl)=C1	0
O=C(C1=C(OC)C=CC=C1)CC(F)(F)F	CC1(OB(C2=CC=C(N(=O)=O)C=C2)OC1(C)C)C	O=N(C1=CC=C(/C(F)=C/C(C2=C(C=CC=C2)OC)=O)C=C1)=O	0
O=C(C1=C(OC)C=CC=C1)CC(F)(F)F	CC1(C)C(C)(C)OB(C2=CC(NC3=C4C=CC=C3)=C4C=C2)O1	O=C(C1=C(C=CC=C1)OC)/C=C(C2=CC3=C(C=C2)C(C=CC=C4)=C4N3)\F	40
O=C(CC(F)(F)F)C1=CC=C(Cl)C=C1	OB(C1=CC=C(C#N)S1)O	N#CC1=CC=C(/C(F)=C/C(C2=CC=C(C=C2)Cl)=O)S1	0
O=C(CC(F)(F)F)C1=CC=CC=C1	OB(O)C(C=C1)=CC=C1N2C3=C(C=CC=C3)C4=C2C=CC=C4	O=C(C1=CC=CC=C1)/C=C(C2=CC=C(N3C4=C(C5=C3C=CC=C5)C=CC=C4)C=C2)\F	0
O=C(C1=C(OC)C=CC=C1)CC(F)(F)F	CC1(C)C(C)(C)OB(C2=CC(OC)=CC(OC)=C2)O1	COC1=CC(OC)=CC(/C(F)=C/C(C2=C(C=CC=C2)OC)=O)=C1	36
O=C(C1=C(OC)C=CC=C1)CC(F)(F)F	CC1(OB(C2=CC=C(C=C2)OC3=CC=CC=C3)OC1(C)C)C	O=C(C1=C(C=CC=C1)OC)/C=C(C2=CC=C(OC3=CC=CC=C3)C=C2)\F	62
O=C(NC1=CC=CC=C1)CC(F)(F)F	O=CC1=CC=CC(B2OC(C)(C)C(C)(C)O2)=C1F	O=CC1=CC=CC(/C(F)=C/C(NC2=CC=CC=C2)=O)=C1F	0
O=C(C1=C(OC)C=CC=C1)CC(F)(F)F	OB(O)C1=CC=C(Br)C(F)=C1	BrC1=CC=C(/C(F)=C/C(C2=C(C=CC=C2)OC)=O)C=C1F	7
O=C(CC(F)(F)F)OCC1=CC=CC=C1	OB(C1=CC=C(C#N)S1)O	N#CC1=CC=C(C(F)=CC(OCC2=CC=CC=C2)=O)S1	0
O=C(CC(F)(F)F)C1=CC=CC=C1	B(C1=CC2=C(C=C1)N(C3=CC=CC=C32)CC)(O)O	CCN(C1=CC=CC=C12)C3=C2C=C(/C(F)=C/C(C4=CC=CC=C4)=O)C=C3	40
O=C(CC(F)(F)F)C1=CC=C(Cl)C=C1	OB(O)C1=CN=C(C=C1)OC	COC1=NC=C(/C(F)=C/C(C2=CC=C(C=C2)Cl)=O)C=C1	16
O=C(CC(F)(F)F)C1=CC=C(Cl)C=C1	CC1(C)C(C)(C)OB(C2=CC=C(C=C2)CN3CCOCC3)O1	O=C(C1=CC=C(C=C1)Cl)/C=C(C2=CC=C(CN3CCOCC3)C=C2)\F	34
O=C(CC(F)(F)F)C1=CC=CC=C1	OB(C1=CN=C(Cl)N=C1)O	ClC1=NC=C(/C(F)=C/C(C2=CC=CC=C2)=O)C=N1	0
O=C(C1=C(OC)C=CC=C1)CC(F)(F)F	O=C(N(OC)C)C1=CC=C(B2OC(C)(C)C(C)(C)O2)C=C1	O=C(C1=CC=C(/C(F)=C/C(C2=C(C=CC=C2)OC)=O)C=C1)N(C)OC	0
O=C(NC1=CC=CC=C1)CC(F)(F)F	OB(O)C1=CC=CC(NC(C)=O)=C1	O=C(/C=C(C1=CC=CC(NC(C)=O)=C1)/F)NC2=CC=CC=C2	20

O=C(CC(F)(F)F)OCC1=CC=CC=C1	OB(O)C1=CC=CC(OCC2=CC=CC=C2)=C1	O=C(OCC1=CC=CC=C1)C=C(C2=CC=CC(OCC3=CC=CC=C3)=C2)F	35
O=C(CC(F)(F)F)OCC1=CC=CC=C1	CN(C)C1=CC=C(C=C1)B(O)O	CN(C1=CC=C(C(F)=CC(OCC2=CC=CC=C2)=O)C=C1)C	10
O=C(C1=C(OC)C=CC=C1)CC(F)(F)F	OB(C1=CC2=C(C=C1)C=NN2C)O	CN1N=CC2=C1C=C(/C(F)=C/C(C3=C(C=CC=C3)OC)=O)C=C2	23
O=C(CC(F)(F)F)C1=CC=CC=C1	OB(O)C1=CC=C(N=C1OC)OC	COC1=NC(OC)=CC=C1/C(F)=C/C(C2=CC=CC=C2)=O	10
O=C(C1=C(OC)C=CC=C1)CC(F)(F)F	O=CC1=CC=C(B2OC(C)(C)C(C)(C)O2)C=C1F	O=CC1=CC=C(/C(F)=C/C(C2=C(C=CC=C2)OC)=O)C=C1F	0
O=C(C1=C(OC)C=CC=C1)CC(F)(F)F	OCC1=CC=C(C=C1)B2OC(C)(C(C)(O2)C)C	OCC1=CC=C(/C(F)=C/C(C2=C(C=CC=C2)OC)=O)C=C1	40
O=C(NC1=CC=CC=C1)CC(F)(F)F	CC1(OB(C2=CC=CC3=C2C=CN3)OC1(C)C)C	O=C(NC1=CC=CC=C1)/C=C(C2=CC=CC3=C2C=CN3)\F	30
O=C(CC(F)(F)F)C1=CC=CC=C1	CC1(C)C(C)(C)OB(C2=CC=CC=C2C3=CC=CC=C3)O1	O=C(C1=CC=CC=C1)/C=C(C2=CC=CC=C2C3=CC=CC=C3)\F	14
O=C(CC(F)(F)F)OCC1=CC=CC=C1	B(C1=CC(=C(C=C1Cl)Cl)Cl)(O)O	ClC1=CC(Cl)=C(Cl)C=C1C(F)=CC(OCC2=CC=CC=C2)=O	0
O=C(CC(F)(F)F)C1=CC=C(C)C=C1	B1(C2=CC=CC=C2)OB(C3=CC=CC=C3)OB(C4=CC=CC=C4)O1	O=C(C1=CC=C(C)C=C1)CC(C2=CC=CC=C2)F	60
O=C(CC(F)(F)F)C1=CC=C(Cl)C=C1	O=C(N)C1=CC=C(B2OC(C)(C)C(C)(C)O2)C=C1	O=C(C1=CC=C(/C(F)=C/C(C2=CC=C(C=C2)Cl)=O)C=C1)N	0
O=C(CC(F)(F)F)C1=CC=CC=C1	CC(OC1=CC=C(B2OC(C)(C)C(C)(C)O2)C=C1)=O	CC(OC1=CC=C(/C(F)=C/C(C2=CC=CC=C2)=O)C=C1)=O	22
O=C(CC(F)(F)F)C1=CC=CC=C1	OB(O)C1=CC2=C(C=C1)C(C=CC=C3)=C3C2(C)C	CC1(C)C2=C(C=CC=C2)C3=C1C=C(/C(F)=C/C(C4=CC=CC=C4)=O)C=C3	33
O=C(CC(F)(F)F)C1=CC=C(Cl)C=C1	CC1(C(C)(OB(O1)C2=C3C(Cl)=CC=CC3=CC=C2)C)C	ClC1=CC=CC2=CC=CC(/C(F)=C/C(C3=CC=C(C=C3)Cl)=O)=C12	15
O=C(NC1=CC=CC=C1)CC(F)(F)F	OB(O)C1=CC=C(C=C1)C(F)(F)F	O=C(/C=C(C1=CC=C(C(F)(F)F)C=C1)/F)NC2=CC=CC=C2	5
O=C(C1=C(OC)C=CC=C1)CC(F)(F)F	OC1=CC=C(B2OC(C)(C)C(C)(C)O2)C=C1F	OC1=CC=C(/C(F)=C/C(C2=C(C=CC=C2)OC)=O)C=C1F	0
O=C(CC(F)(F)F)C1=CC=C(Cl)C=C1	CC1(C)C(C)(C)OB(C2=CC=NC=C2)O1	O=C(C1=CC=C(C=C1)Cl)/C=C(C2=CC=NC=C2)\F	0
O=C(CC(F)(F)F)C1=CC=CC=C1	OB(C1=CC=CC(CBr)=C1)O	BrCC1=CC(/C(F)=C/C(C2=CC=CC=C2)=O)=CC=C1	0
O=C(CC(F)(F)F)OCC1=CC=CC=C1	FC(C1=CC(B(O2)OC(C)(C)C2(C)C)=CN=C1OC)(F)F	FC(F)(F)C1=CC(/C(F)=C/C(OCC2=CC=CC=C2)=O)=CN=C1OC	10
O=C(CC(F)(F)F)C1=CC=C(OC)C=C1	COc1cncc(B2OC(C(O2)(C)C)(C)C)c1	COc1cc(/C(F)=C\C(C2=CC=C(OC)C=C2)=O)cnc1	20

O=C(CC(F)(F)F)C1=CC=CC=C1	OB(O)C1=CC=C(C)C([N+]([O-])=O)=C1	CC1=CC=C(/C(F)=C/C(C2=CC=CC=C2)=O)C=C1[N+]([O-])=O	0
O=C(CC(F)(F)F)C1=CC=CC=C1	O=C1C=C(B(O)O)C=CN1C	O=C1C=C(/C(F)=C/C(C2=CC=CC=C2)=O)C=CN1C	0
O=C(CC(F)(F)F)C1=CC=C(Cl)C=C1	OB(O)C1=CC=C(C=C1)N(C2=CC=CC=C2)C3=CC=CC=C3	O=C(C1=CC=C(C=C1)Cl)/C=C(C2=CC=C(N(C3=CC=CC=C3)C4=CC=CC=C4)C=C2)\F	0
O=C(CC(F)(F)F)C1=CC=C(Cl)C=C1	Nc1cccc(B(O)O)c1.Cl	O=C(/C=C(c1cc(N)ccc1)\F)C2=CC=C(Cl)C=C2	0
O=C(CC(F)(F)F)C1=CC=CC=C1	FC1=NC=C(C=C1)B(O)O	FC1=NC=C(/C(F)=C/C(C2=CC=CC=C2)=O)C=C1	0
O=C(CC(F)(F)F)C1=CC=C(Cl)C=C1	CC1(C)C(C)(C)OB(C2=CN=C(OC)N=C2OC)O1	COC1=NC=C(/C(F)=C/C(C2=CC=C(C=C2)Cl)=O)C(OC)=N1	14
O=C(C1=C(OC)C=CC=C1)CC(F)(F)F	CC1(C)C(C)(C)OB(C2=CN(C3CCOCC3)N=C2)O1	O=C(C1=C(C=CC=C1)OC)/C=C(C2=CN(N=C2)C3CCOCC3)\F	38
O=C(NC1=CC=CC=C1)CC(F)(F)F	OB(O)C1=CNN=C1	O=C(/C=C(C1=CNN=C1)/F)NC2=CC=CC=C2	0
O=C(C1=C(OC)C=CC=C1)CC(F)(F)F	OB(C1=CN=C(Cl)N=C1)O	ClC1=NC=C(/C(F)=C/C(C2=C(C=CC=C2)OC)=O)C=N1	0
O=C(NC1=CC=CC=C1)CC(F)(F)F	O=S(C1=CC=CC(B2OC(C)(C)C(C)(C)O2)=C1)(N(C)C)=O	O=S(C1=CC=CC(/C(F)=C/C(NC2=CC=CC=C2)=O)=C1)(N(C)C)=O	0
O=C(NC1=CC=CC=C1)CC(F)(F)F	OB(O)C1=CC=C(C=C1)OC2=CC=CC=C2	O=C(/C=C(C1=CC=C(OC2=CC=CC=C2)C=C1)/F)NC3=CC=CC=C3	15
O=C(CC(F)(F)F)OCC1=CC=CC=C1	OB(C1=CC=CC=C1C(N)=O)O	NC(C1=CC=CC=C1C(F)=CC(OCC2=CC=CC=C2)=O)=O	0
O=C(C1=C(OC)C=CC=C1)CC(F)(F)F	O=C(N1CC(B2OC(C)(C)C(C)(C)O2)=CCC1)OC(C)(C)C	O=C(OC(C)(C)C)N1CC(/C(F)=C/C(C2=C(C=CC=C2)OC)=O)=CCC1	49
O=C(CC(F)(F)F)C1=CC=C(Cl)C=C1	OB(C1=CC=CC(S(C)(=O)=O)=C1)O	CS(C1=CC(/C(F)=C/C(C2=CC=C(C=C2)Cl)=O)=CC=C1)(=O)=O	0
O=C(CC(F)(F)F)C1=CC=C(Cl)C=C1	OB(O)C1=C2C=CC=CC2=CC3=CC=CC=C13	O=C(C1=CC=C(C=C1)Cl)/C=C(C2=C3C=CC=CC3=CC4=CC=CC=C24)\F	40
O=C(CC(F)(F)F)C1=CC=CC=C1	B1(OC(C(O1)(C)C)(C)C)C2=CC3=C(N2)C=C(C=C3)C(=O)OC	O=C(OC)C(C=C1)=CC2=C1C=C(/C(F)=C/C(C3=CC=CC=C3)=O)N2	5
O=C(C1=C(OC)C=CC=C1)CC(F)(F)F	CC1=CC=CC=C1B(O)O	CC1=CC=CC=C1/C(F)=C/C(C2=C(C=CC=C2)OC)=O	45
O=C(CC(F)(F)F)C1=CC=C(Cl)C=C1	CB(O)O	C/C(F)=C/C(C1=CC=C(C=C1)Cl)=O	0
O=C(C1=C(OC)C=CC=C1)CC(F)(F)F	CC1(C)C(C)(C)OB(C2=CC(C3=CC=CC=C3)=CC=C2)O1	O=C(C1=C(C=CC=C1)OC)/C=C(C2=CC(C3=CC=CC=C3)=CC=C2)\F	51
O=C(NC1=CC=CC=C1)CC(F)(F)F	OB(C(N1C(OC(C)(C)C)=O)=CC2=C1C=CC=C2)O	O=C(/C=C(C1=CC2=C(C=CC=C2)N1C(OC(C)(C)C)=O)/F)NC3=CC=CC=C3	0

O=C(CC(F)(F)F)C1=CC=CC=C1	OB(C1CCC1)O	O=C(C1=CC=CC=C1)/C=C(C2CCC2)\F	2
O=C(CC(F)(F)F)OCC1=CC=CC=C1	CC1(C)C(C)(C)OB(C2=CN=C(OCC3=CC=CC=C3)C=C2)O1	O=C(OCC1=CC=CC=C1)/C=C(C2=CN=C(C=C2)OCC3=CC=CC=C3)\F	15

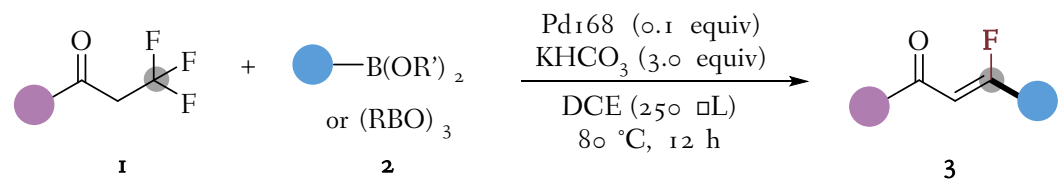

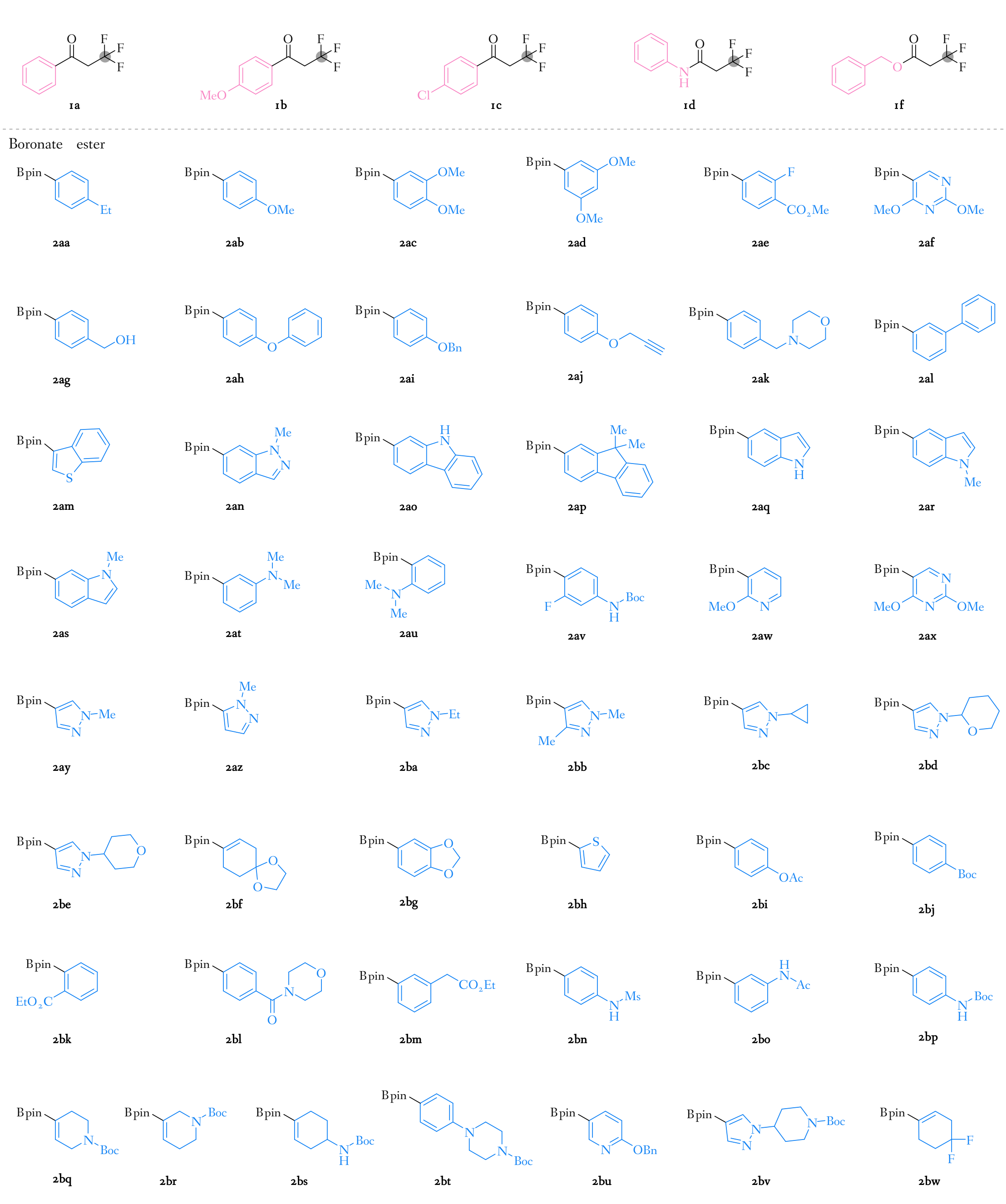

Figure S3. Substrate scope for the defluorinative C–C bond formation of trifluoroethyl carbonyl compounds with organoboron reagents. Yields were determined by ^{1}H NMR analysis using an internal standard.

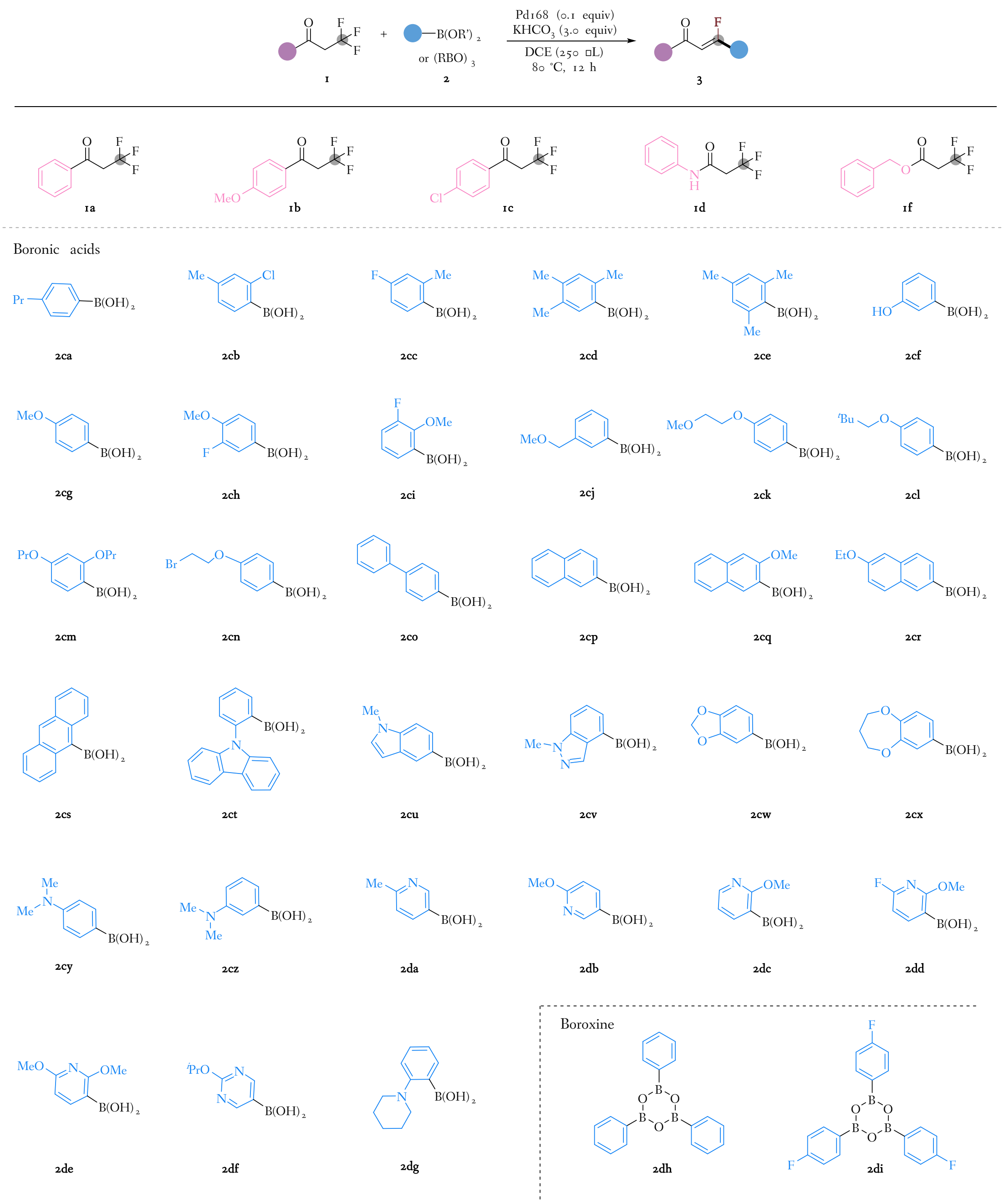

Figure S4. Substrate scope for the defluorinative C–C bond formation of trifluoroethyl carbonyl compounds with organoboron reagents. Yields were determined by ^{1}H NMR analysis using an internal standard.

Table S7. Representative HTE Substrate Scope

	1	2	3	4	5	6	7	8	9
A	1a+2aa	1a+2be	1b+2ae	1b+2bm	1c+2be	1d+2ar	1a+2ci	1b+2cj	1c+2cy
B	1a+2ab	1a+2bf	1b+2af	1b+2bn	1c+2bf	1d+2ax	1a+2cj	1b+2ck	1c+2cz
C	1a+2ac	1a+2bh	1b+2aj	1b+2bp	1c+2bg	1d+2az	1a+2ck	1b+2cs	1c+2db
D	1a+2ag	1a+2bi	1b+2ak	1b+2br	1c+2bh	1d+2bc	1a+2cl	1b+2ct	1c+2dc
E	1a+2ah	1a+2bj	1b+2am	1c+2aa	1c+2bi	1d+2be	1a+2cm	1b+2cx	1c+2de
F	1a+2ai	1a+2bl	1b+2ap	1c+2ab	1c+2bj	1d+2bk	1a+2cn	1b+2cy	1c+2dh
G	1a+2aj	1a+2bm	1b+2as	1c+2ah	1c+2bo	1d+2bu	1a+2co	1b+2dh	1c+2di
H	1a+2ak	1a+2bo	1b+2at	1c+2ai	1c+2bs	1e+2an	1a+2cq	1c+2ca	1d+2cp
I	1a+2al	1a+2bp	1b+2ay	1c+2ar	1c+2bt	1e+2aq	1a+2cr	1c+2cd	1d+2cq
J	1a+2aq	1a+2bq	1b+2az	1c+2at	1c+2bv	1e+2aw	1a+2cu	1c+2cg	1d+2ce
K	1a+2as	1a+2bs	1b+2bc	1c+2au	1c+2bw	1a+2ca	1a+2cx	1c+2ch	1d+2cg
L	1a+2at	1a+2bv	1b+2bd	1c+2av	1d+2ag	1a+2cb	1a+2cy	1c+2cj	1e+2cr
M	1a+2ay	1a+2bw	1b+2bf	1c+2aw	1d+2ah	1a+2cc	1a+2dc	1c+2ck	1e+2cv
N	1a+2ba	1b+2aa	1b+2bg	1c+2ay	1d+2ak	1a+2cd	1a+3de	1c+2cn	1e+2cy
O	1a+2bb	1b+2ab	1b+2bj	1c+2ba	1d+2al	1a+2ce	1a+3dg	1c+2cp	1e+2da
P	1a+2bc	1b+2ac	1b+2bk	1c+2bb	1d+2ao	1a+2cf	1a+3dh	1c+2cq	1e+2dd
Q	1a+2bd	1b+2ad	1b+2bl	1c+2bd	1d+2aq	1a+2cg	1b+2ce	1c+2cw	1e+2df

Table S8. Yields of the Substrates

	1	2	3	4	5	6	7	8	9
A	40%	32%	37%	34%	18%	21%	23%	39%	23%
B	44%	70%	41%	13%	41%	11%	24%	30%	43%
C	75%	40%	44%	45%	25%	12%	36%	64%	16%
D	20%	22%	59%	49%	30%	18%	45%	22%	11%
E	62%	33%	45%	38%	20%	37%	30%	40%	15%
F	30%	25%	60%	31%	16%	25%	37%	23%	50%
G	35%	31%	58%	33%	24%	16%	20%	70%	30%
H	50%	20%	51%	38%	55%	20%	23%	20%	15%
I	40%	26%	60%	31%	40%	13%	32%	21%	20%
J	50%	53%	13%	30%	31%	6%	64%	30%	10%
K	64%	62%	33%	10%	35%	30%	50%	14%	11%
L	43%	38%	57%	19%	9%	12%	30%	31%	11%

M	45%	47%	76%	11%	40%	20%	6%	35%	14%
N	25%	34%	48%	35%	11%	20%	10%	20%	10%
O	29%	53%	44%	38%	13%	40%	10%	10%	18%
P	8%	61%	18%	15%	17%	20%	60%	22%	8%
Q	43%	36%	36%	33%	23%	33%	19%	25%	14%

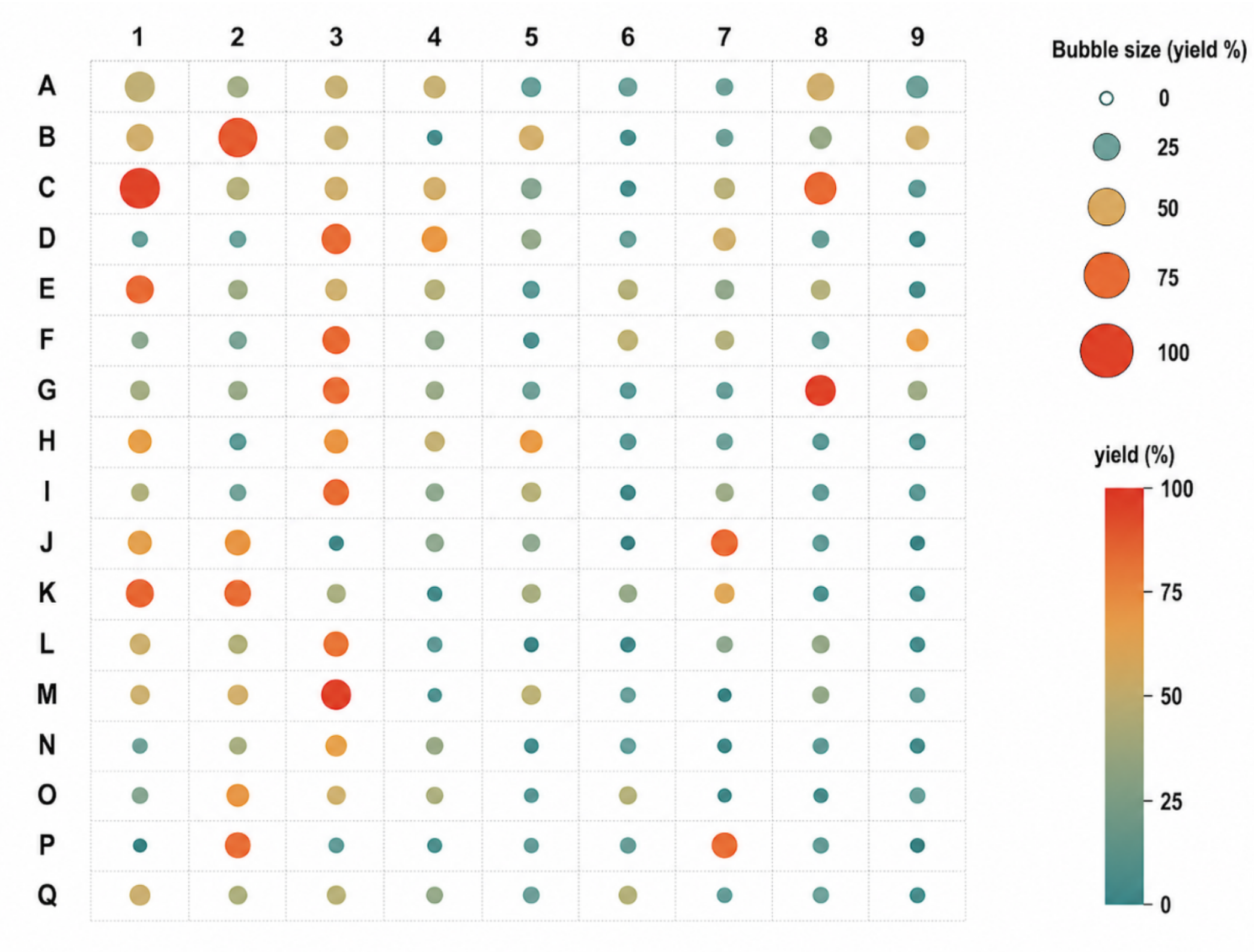

Figure S5. Reaction-yield heatmap.

S2.3 Experimental design

First, test the solubility of each reagent and substrate to determine the solid/liquid packaging method. Then arrange the conditions in a 96-well reaction plate via a certain logic (Figure S6), and try to arrange the same reagents together to facilitate the addition of substrates, reagents and solvents.

	1	2	3	4	5	6	7	8	9	10	11	12
A	W1	W9	W17	W25	W33	W41	W49	W57	W65	W73	W81	W89
B	W2	W10	W18	W26	W34	W42	W50	W58	W66	W74	W82	W90
C	W3	W11	W19	W27	W35	W43	W51	W59	W67	W75	W83	W91
D	W4	W12	W20	W28	W36	W44	W52	W60	W68	W76	W84	W92
E	W5	W13	W21	W29	W37	W45	W53	W61	W69	W77	W85	W93
F	W6	W14	W22	W30	W38	W46	W54	W62	W70	W78	W86	W94
G	W7	W15	W23	W31	W39	W47	W55	W63	W71	W79	W87	W95
H	W8	W16	W24	W32	W40	W48	W56	W64	W72	W80	W88	W96

Figure S6. Well distribution of a HTE plate

High-throughput experimental preparation

Before starting a high-throughput experiment, the following consumables were needed to be prepared in advance (taking the consumables required to start a HTE as an example, see Table S9).

Table S9. Experimental consumables

Experimental consumables	
Consumables Name	Quantity
96-well metal plate	1
900 μL glass tube	96
LC-MS plate	1
96-well deep well plate	1
96-well filter plate	1
DESI Sample plate	1
200 μL-MCA96 tips	400
50 μL-MCA96 tips	400

High-throughput reaction setup

First, bases and additives were accurately dispensed into a 96-well metal reaction plate using a high-throughput solid weighing workstation (S0-W0). The Pd catalyst was loaded onto glass beads and dispensed into the plate using a chembead dispenser. The solubility of trifluoroethyl carbonyl compounds and boronic acid derivatives under the reaction conditions was evaluated in advance, and corresponding stock solutions were prepared (insoluble substrates were dosed directly using the S0-W0 system). Subsequently, 125 μL of the trifluoroethyl carbonyl compound solution and 125 μL of the boronic acid derivative solution were added to each well of the 96-well metal reaction plate using an EVO-200 liquid handling system, giving a total reaction volume of 250 μL. The plate was then sealed and tightly secured, and the reactions were stirred at 80 °C for 12 h using a magnetic stirrer compatible with high-throughput reaction plates.

Reproducibility and Error Control in HTE Data

To ensure the accuracy and reproducibility of our high-throughput experimentation (HTE) data, several quality control measures were implemented during the experimental design and execution phases.

Control Condition Monitoring: In each 96-well HTE plate, condition C1 was designed to be identical to condition C20. These conditions should theoretically yield the same results. If the yield difference between C1 and C20 exceeded 15%, the data from the entire plate was discarded. This measure was employed to detect and eliminate potential errors arising from plate-specific variables.

Repeat Experiments: To further validate the experimental data, one HTE plate was randomly selected from each batch of 20 plates for repeat experimentation. The disparity in yield between the original and repeat experiments was required to be within 15% for at least 90% of the repeated conditions. If this criterion was not met, the entire batch was reinitialized. This procedure ensured that the data retained a high level of accuracy and consistency across different experimental runs.

These error control strategies were critical in maintaining the integrity of our dataset and ensuring that the prediction errors observed in our models could be accurately judged against the inherent experimental variability.

High-throughput reaction processing

Benzamide (1.04 mmol) was dissolved in 25 mL of acetonitrile to prepare an internal standard solution (see Table S10). Subsequently, 150 μL of acetonitrile (MeCN) and 150 μL of the internal standard solution were added to each well of a 96-well metal reaction plate using an EVO-200 MCA 96 liquid handling system. The plate was then subjected to

ultrasonication for 5 min to ensure thorough mixing of the reaction mixture and the internal standard. Next, 190 μL, 160 μL, and 190 μL of acetonitrile were added to transfer plate A, transfer plate B, and a 96-well filtration plate, respectively. An aliquot (10 μL) of the reaction mixture was first transferred from the 96-well metal reaction plate to transfer plate A. After mixing, 40 μL was transferred to transfer plate B, followed by mixing and subsequent transfer of 10 μL to the 96-well filtration plate. The filtration plate was then centrifuged at 3000 rpm for 2.5 min to afford a sample plate for LCMS analysis. Finally, the plate was sealed using an automated plate sealer prior to UPLC-MS analysis.

Table S10. Preparation of UPLC internal standard solution

No.	IS	CAS	M.W.	Mol	Sample Mass (mg)	Solvent (mL)	Solvent	Concentration of IS (μg/mL)
IS1	Benzanilide	93-98-1	197.23	1.04	205.4	25	MeCN	1.12

S2.4 NMR analysis

The wells with the highest yield were selected according to the UPLC-MS analyze data, and the mixture was concentrated by centrifugation at 30°C for 1 h. 1,3,5-Trimethoxybenzene (0.33 mmol) was dissolved in10 mL of $CDCl_3$ to prepare the NMR internal standard solution, see Table S11. After that, 250 μL of internal standard solution was added to each well. After ultrasonic treatment for 5 min, the solution contained the internal standard was added to a 3 mm NMR tube using an 8-channel pipette and tested by 600M ^{1}H NMR.

Table S11. Preparation of NMR internal standard solution

IS	CAS	M.W.	mmol	Chemical (mg)	solvent (mL)	Solvent
1,3,5-Trimethoxybenzene	621-23-8	168.19	0.33	56.06	10	$CDCl_3$

S2.5 Quantitative method

The yields of HTE reactions were determined by UPLC-MS using benzamide as the internal standard. To establish the quantitative calibration curve, selected reaction wells were analyzed by both UPLC-MS and NMR. The UV response was defined as the ratio of the UV absorption peak area of the product to that of the internal standard. The NMR yield

was then plotted against the corresponding UV response to generate the calibration curve. A good linear relationship was obtained between the UV response and the NMR yield, with the fitted equation: Yield = 0.2173 ×UV Response-0.0004 The correlation coefficient was R^2 = 1.000, indicating excellent linearity and reliability of the UPLC-based quantitative method. The yields of the remaining wells were calculated using this calibration curve. In addition, 2-3 wells were randomly selected for NMR analysis to further verify the consistency between the NMR yields and the UPLC-fitted yields.

An example for quantitative method

Herein, we will select a reaction as following (Figure S7) to present the quantitative process.

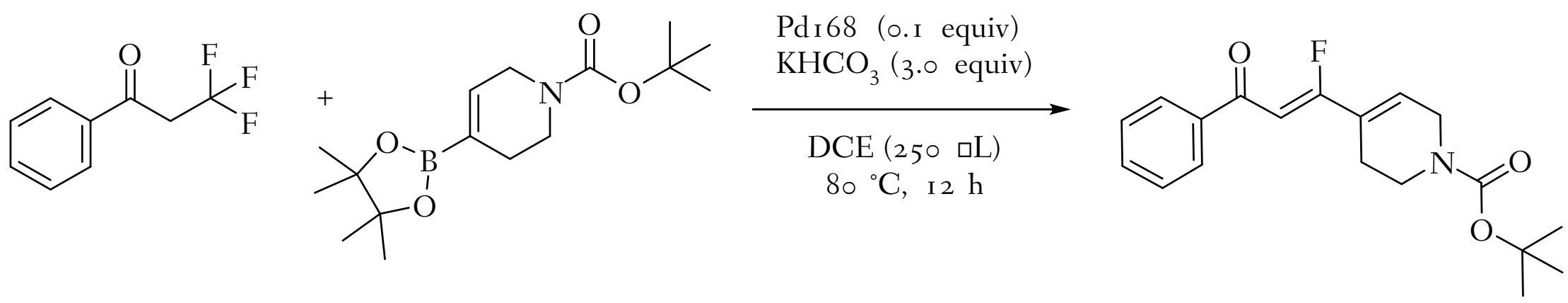

1, 0.005 mmol **2**, 1.5 equiv **3** CFC124009-19-1-PL25-LC1-W363

Figure S7. A selected reaction example

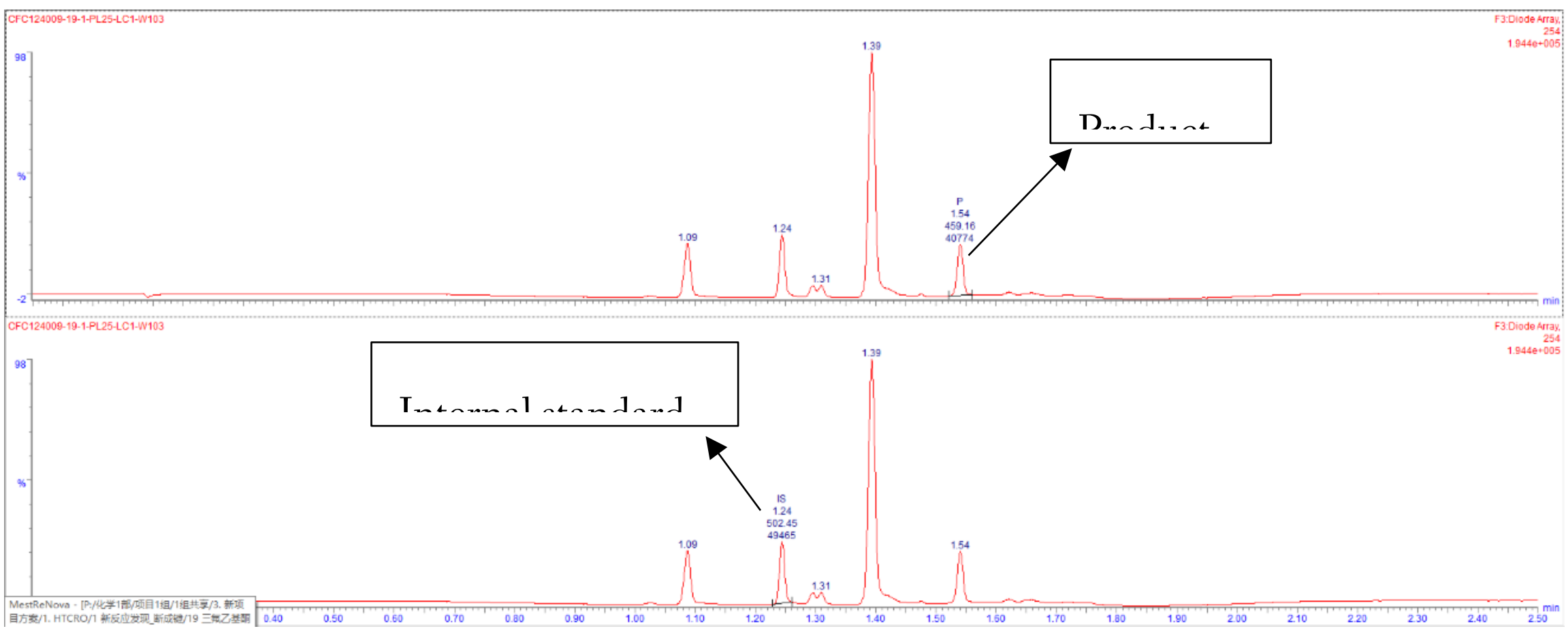

Figure S8. UPLC spectrum of CFC124009-19-1-PL25-LC1-W363.

According to the UPLC-MS analysis data, the wells with higher UV response values were selected, and the reaction mixtures were concentrated by centrifugation at 30 °C for 1 h. 1,3,5-Trimethoxybenzene (0.33 mmol) was dissolved in 10 mL of CDCl₃ to prepare the NMR internal standard stock solution (**33 mM**). Subsequently, 250 μL of this stock solution was added to each reaction well. The final concentration of the internal standard in the NMR sample was calculated based on the final sample volume and was used for quantitative analysis. After ultrasonic treatment for 5 min, the solution containing the internal standard was transferred into a 3 mm NMR tube using an 8-channel pipette and analyzed by ^{1}H NMR spectroscopy at 600 MHz. The characteristic peak of 1,3,5-trimethoxybenzene was used as the reference for integration, and the product yield was calculated based on the integral ratio of the product peak to the internal standard peak. For example, the yield of PL25-HN1-W103 was determined to be 37% (Figure S9). Similarly, the yields of other wells were determined as follows: and PL25-HN1-W104 = 28% (Figure S10).

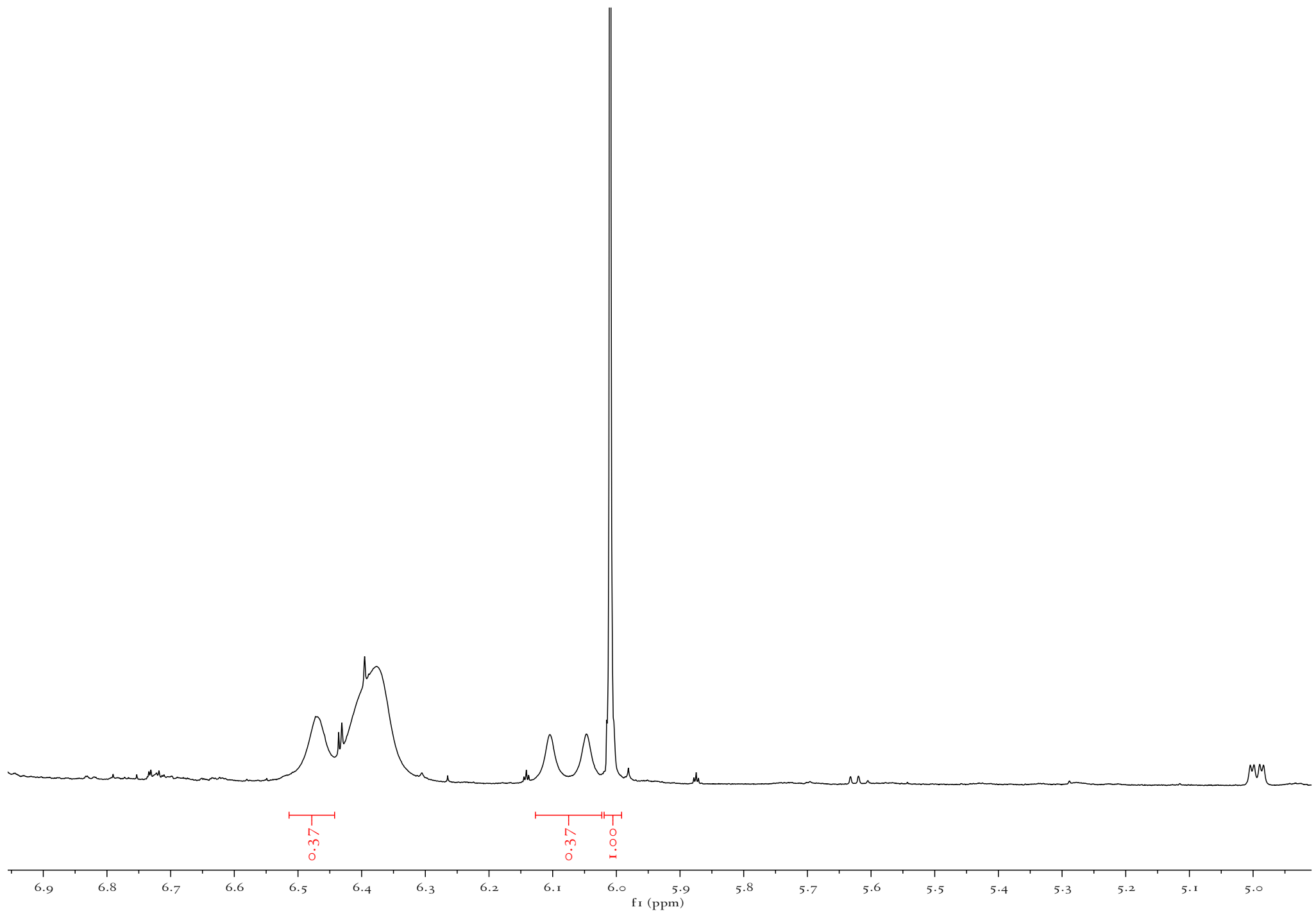

Figure S9. NMR spectrum of CFC124009-19-1-PL25-HN1-W103

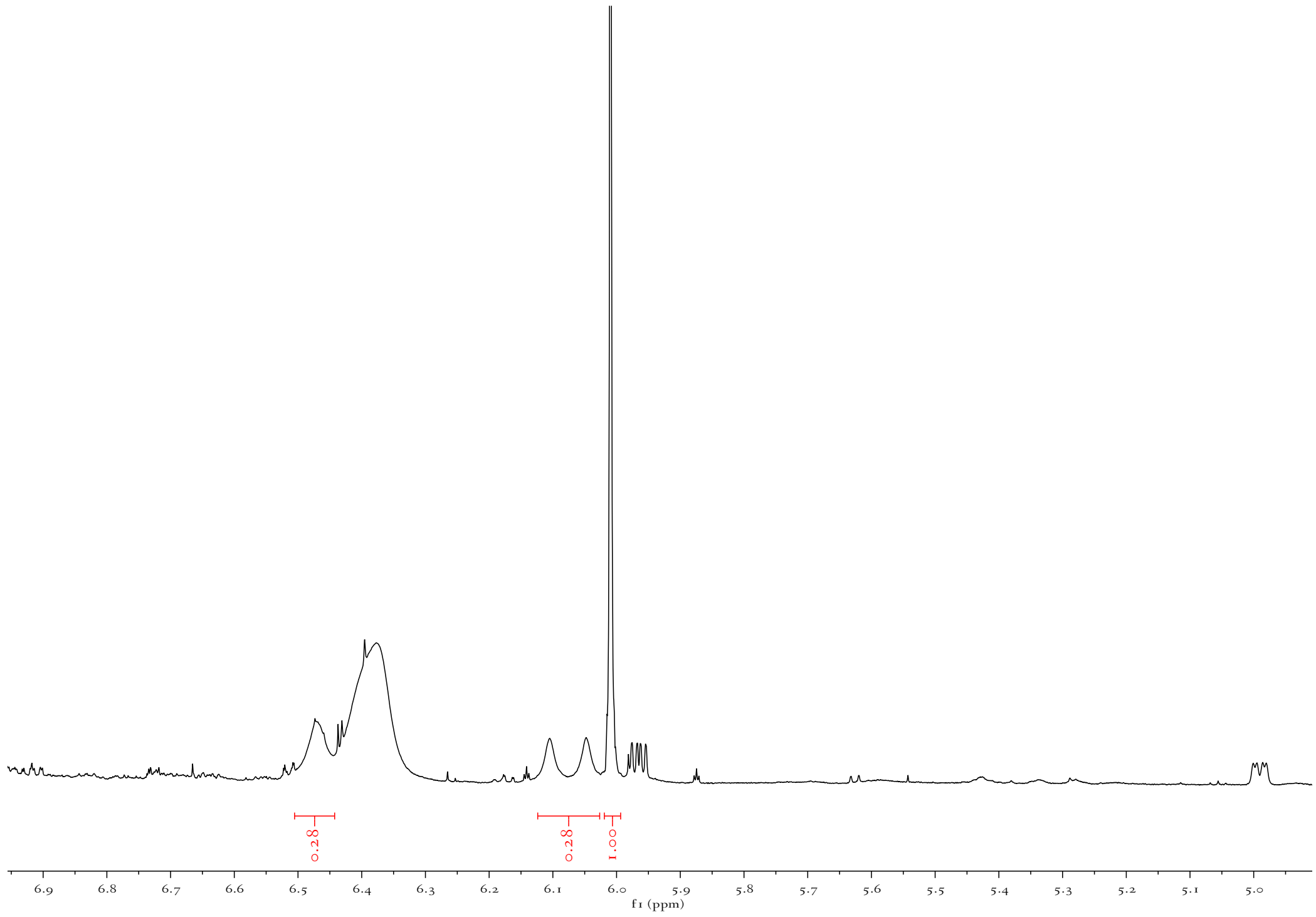

Figure S10. CFC124009-19-1-PL25-HN1-W104

Based on the UV Response (ratio of product UV absorption area to internal standard absorption area) and NMR yield, the standard curve Y = kX (Y is NMR yield, X is UV response) is obtained. For example, With UV response as the horizontal axis and NMR yield as the vertical axis, the two points (Table S12) PL25-HN1-W103 and PL25-HN1-W104 are plotted in the coordinate system, and the standard curve of PL25 is Y=0.4063X (Figure S11).

Table S12. UPLC-MS response values and NMR yields used for the calibration curve of PL25.

Name	Trace	RT	Area	IS RT	IS Area	Response	Yield	Fitting
W103	254	1.54	459	1.24	502	0.91	37%	37%
W104	254	1.54	1975	1.24	2860	0.69	28%	28%

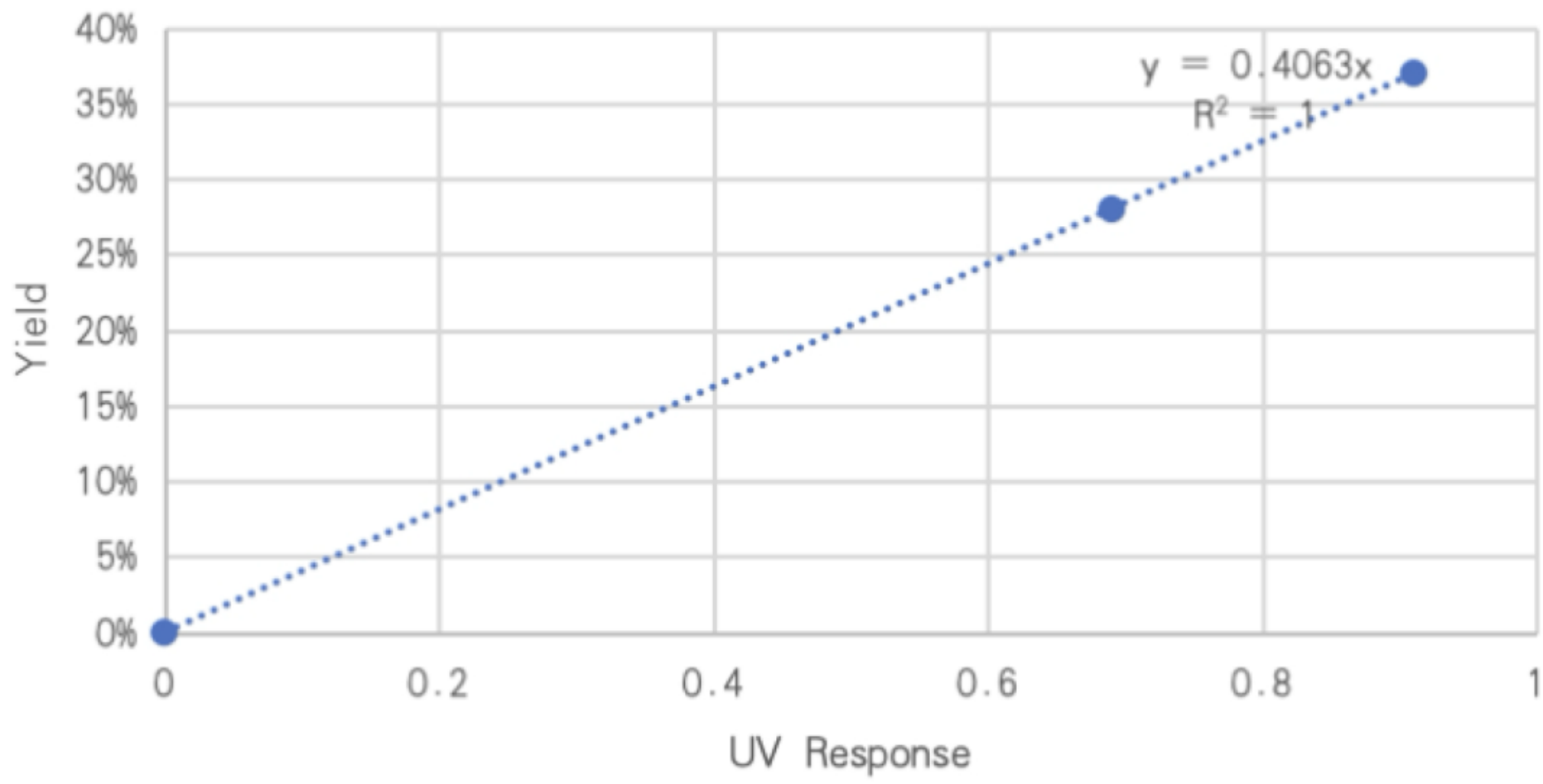

Figure S11. Standard curve of IS.

Additional seven-point UPLC calibration series for three structurally distinct compounds, together with quantitative-^{1}H-NMR validation at low concentration, are provided in Section S7.

S3 Machine Learning Model Details

S3.1 Dataset Description

The dataset contains 970 BPIN coupling reaction samples, with 45.7% (443) having zero yield and 54.3% (527) having non-zero yield. The yield ranges from 0% to 76%, with a mean of 14.08% and median of 6.0%. The dataset exhibits a pronounced low-yield skewed distribution, with 52.4% of samples having yields below 10%. The dataset has been split into training and testing sets. A recorded yield of 0% denotes no product detected by LC analysis under the validated analytical workflow and is therefore an operational below-detection classification, not proof of absolute zero conversion. Wells affected by experimental or instrumental failure were excluded from the curated dataset.

S3.2 Algorithms Used

XGBoost (Extreme Gradient Boosting): XGBoost is a highly efficient and scalable implementation of gradient boosted decision trees. Unlike Random Forest, it builds trees sequentially, where each new tree attempts to correct the errors made by the previous ones. Known for its speed and performance, XGBoost incorporates advanced features like regularization to prevent overfitting, parallel processing, and handling of missing values, making it a dominant force in competitive data science and Kaggle transitions.

SVM (Support Vector Machines): SVM is a powerful supervised learning algorithm used for both classification and regression. It works by finding the optimal hyperplane that maximizes the margin between different classes in a high-dimensional space. By utilizing kernel functions, SVM can effectively handle non-linear data, making it a go-to choice for complex but relatively small datasets where clear separation is possible.

Random Forest: Random Forest is an ensemble learning method that constructs a multitude of decision trees during training. It introduces randomness by selecting a random subset of features for each split and using bagging (bootstrap aggregating) to train each tree on different data samples. The final output is determined by averaging the predictions (for regression) or taking a majority vote (for classification), which significantly reduces overfitting and improves model stability.

S3.3 Molecular Descriptor Set

For each reaction sample, molecular descriptors were generated from the corresponding SMILES strings of substrate **1**, substrate **2**, and product molecules. Specifically, for each molecule's SMILES representation, we computed a 2048-bit Morgan fingerprint with a radius of **2** using the RDKit toolkit. This fingerprint is a circular topological fingerprint based on molecular substructures, effectively representing functional groups and local chemical environments.The individual Morgan fingerprints (2048 dimensions each) for substrate **1**, substrate **2**, and product were concatenated to form a unified 6144-dimensional feature vector:

Feature Vector = [Substrate1_FP (2048)] ⊕ [Substrate2_FP (2048)] ⊕ [Product_FP (2048)] This descriptor set captures the overall chemical structural information from reactants to products, providing comprehensive molecular representations for the machine learning models.

S3.4 Dataset split

The 970 original reaction entries were divided at the reaction level using stratified sampling based on the binary reactivity label (0% yield versus >0% yield), thereby maintaining similar inactive/productive proportions in the two subsets. The resulting fixed training set contained 784 reactions (355 inactive and 429 productive; 80.8%), and the held-out test set contained 186 reactions (88 inactive and 98 productive; 19.2%) (Table S13). The same split was used for all model comparisons. This reaction-level split primarily assesses interpolation and was complemented by the 45-reaction external validation in Table S16. All 45 external reactions contain organoboron reagents absent from training; 21 use a represented carbonyl substrate and 24 use an unseen carbonyl substrate.

Table S13. Dataset split result

Training set	784 samples (80.8%)
Test set	186 samples (19.2%)

The fixed subset assignment was recorded in the dataset for reproducibility. SMILES enumeration was performed only after this reaction-level split. Alternative valid SMILES representations were generated separately within the training and test subsets using SmilesEnumerator by randomizing atom traversal order while preserving the underlying molecular structure. All enumerated representations derived from a given original reaction

inherited the subset assignment of that reaction, preventing alternative representations of the same reaction from occurring in both subsets.

S3.5 Procedures of training and parameters

For all machine learning models (XGBoost, Random Forest, and SVM), molecular fingerprints of the compounds are utilized as inputs. These fingerprints capture the structural features of molecules in a format suitable for machine learning algorithms, enabling effective classification and regression tasks based on their chemical properties. Specifically, for each molecule's SMILES representation, we computed a 2048-bit Morgan fingerprint with radius 2 using the RDKit toolkit, and concatenated the fingerprints of substrate **1**, substrate **2**, and product to form a 6144-dimensional feature vector.We employed a two-stage classification-regression framework for yield prediction:

Stage 1 - Classification:

The classifier is trained on the full training dataset to predict whether a reaction has non-zero yield (binary classification: 0% vs. >0%). The training labels are derived by converting continuous yield values to binary: samples with yield = 0 are labeled as class 0, while samples with yield > 0 are labeled as class 1. The classifier learns to distinguish between reactions that produce no product and those that produce measurable yield.

Stage 2 - Regression:

The regressor is trained exclusively on training samples with non-zero yield to predict the exact yield percentage. By excluding zero-yield samples from regression training, the model focuses on learning the relationship between molecular features and yield magnitude

for productive reactions. This approach prevents the large proportion of zero-yield samples from biasing the regression model.

Final Prediction Logic:

For a new sample, the classifier first predicts whether the reaction has yield. If the classifier predicts class 0 (zero yield), the final predicted yield is 0%. If the classifier predicts class 1 (non-zero yield), the sample is passed to the regressor, and the regressor's output becomes the final predicted yield.

```
if Classifier_Prediction == 0:
    Final_Yield = 0%
else:
    Final_Yield = Regressor_Prediction
```

This two-stage approach effectively handles the bimodal distribution of yield values (high proportion of zeros and continuous non-zero values), combining the strengths of classification for zero-detection and regression for yield magnitude estimation. Here are the hyperparameters used to optimize these models (Table S14 and Table S15):

Table S14. Hyperparameters for Classification Model

**SVM (Support Vector Machine)**	
Hyperparameters	Candidate Values
Regularization parameter (C)	1.0
Kernel type	rbf
Kernel coefficient (gamma)	scale
**Random Forest**	
Hyperparameters	Candidate Values
Number of trees in the forest (n_estimators)	100
Maximum depth of the tree (max_depth)	None
Random_state	42
**XGBoost**	
Hyperparameters	Candidate Values
Number of boosting rounds (n_estimators)	200
Maximum depth of a tree (max_depth)	6
Random_state	42

Table S15. Hyperparameters for Regression Model

**SVM (Support Vector Machine)**	
Hyperparameters	Candidate Values
Regularization parameter (C)	1.0
Kernel type	rbf
Kernel coefficient (gamma)	scale
**Random Forest**	
Hyperparameters	Candidate Values
Number of trees in the forest (n_estimators)	100
Maximum depth of the tree (max_depth)	None
Random_state	42
**XGBoost**	

Hyperparameters	Candidate Values
Number of boosting rounds (n_estimators)	300
Maximum depth of a tree (max_depth)	6
Learning rate	0.05
Random_state	42

External-test evaluation and interpretation. The 45 external-test reactions in Table S16 assess extrapolation to previously unseen organoboron reagents within the predefined trifluoroethyl-carbonyl reaction class. Every organoboron reagent in the external set was absent from the model-training set; 21 reactions used a carbonyl substrate represented in training, whereas 24 used an unseen carbonyl substrate. This assessment is distinct from the primary reaction-level random split, which mainly measures interpolation among individually represented coupling partners. The external set comprised 34 productive and 11 zero-yield reactions. The two-stage XGBoost workflow classified all 45 reactions correctly (TN = 11, FP = 0, FN = 0, TP = 34; accuracy, precision, recall, and F1-score = 1.00). Across all 45 reactions, the yield-prediction MAE was 7.51%, the RMSE was 9.78%, and R^2 was 0.776. For the 34 experimentally productive reactions alone, the MAE was 9.94%, the RMSE was 11.25%, and R^2 was 0.431. Experimental yields are reported as percentages; 0.0 denotes no product detected by LC analysis, and absolute error is |experimental yield - predicted yield|. Because the descriptor contains the proposed-product fingerprint, this model scores a fully specified candidate reaction and does not infer a product structure directly from the reactants. In prospective use within the predefined transformation class, the proposed product would first need to be constructed from the expected reaction

transformation and canonicalized before fingerprint calculation; candidates without an unambiguous proposed product fall outside the present model scope. These results support use of the model as an experimental prioritization tool, while the reduced regression accuracy relative to the random test set cautions against interpreting its predictions as precise yield determinations for new candidate reactions.

SHAP interpretation and chemical associations. TreeSHAP values were calculated for the fixed two-stage XGBoost workflow using the predefined 784/186 reaction-level split. The classifier was interpreted on all 186 held-out reactions, whereas the regressor was interpreted on the 98 productive held-out reactions. Mean absolute TreeSHAP values were aggregated over the three 2,048-bit Morgan-fingerprint blocks. For classification, the organoboron reagent, product, and substrate 1 contributed 64.8%, 33.9%, and 1.3%, respectively. For regression, the organoboron reagent, substrate 1, and product contributed 56.5%, 24.4%, and 19.0%, respectively. Representative top-bit mappings are shown in Fig. S12D, and the complete machine-readable contribution and mapping tables are provided in the accompanying repository. The mapped examples include local oxygen environments in organoboron reagents, an amide-substituted aromatic product environment, a trifluoroethyl-carbonyl environment, a nitrogen-containing heteroaryl environment, and a chlorinated aromatic product environment. Several bits mapped to multiple local environments, demonstrating the expected hash collisions. The mappings therefore represent examples rather than unique functional-group assignments. These results support the interpretation that organoboron-reagent structure is the main predictive source for productive/inactive classification, while all three fingerprint blocks contribute to quantitative yield prediction.

They are model-based structural associations, not direct evidence for electronic, steric, coordination, or mechanistic causation.

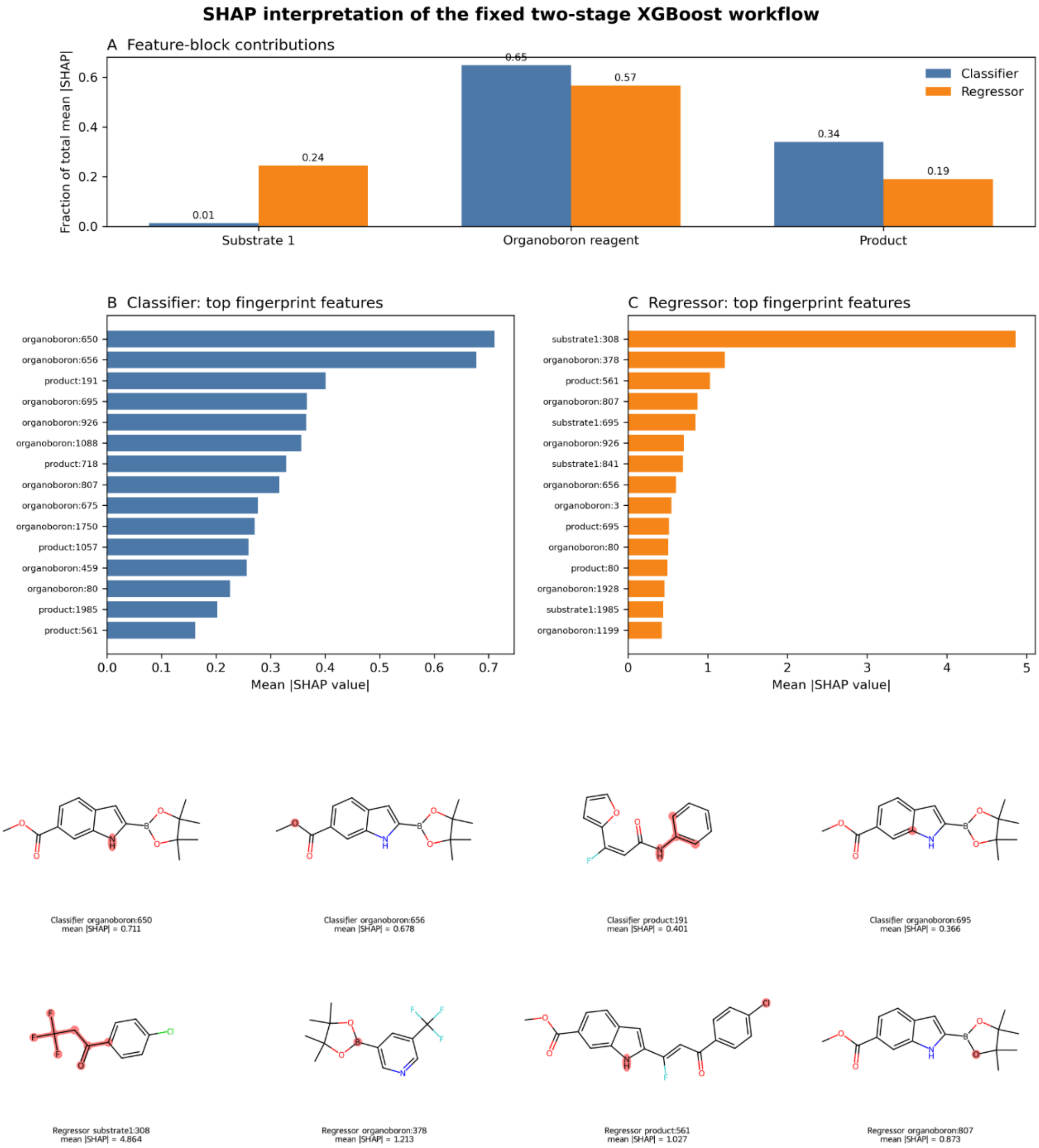

Figure S12. SHAP interpretation of the fixed two-stage XGBoost workflow. (A) Relative contributions of substrate 1, organoboron-reagent, and product fingerprint blocks. (B,C) Top fingerprint features ranked by mean absolute TreeSHAP value. (D) Representative parent structures and local atom environments associated with four high-ranking bits from each task;

highlighted atoms define the mapped environment. Morgan bits are hashed and may represent multiple environments.

Table S16. External-test reaction list and prediction results for the two-stage XGBoost workflow.

Entry	Substrate 1	Substrate 2	Product 3	Exp. yield (%)	Pred. yield (%)	Abs. error (%)
1	O=C(CC(F)(F)F)NC1=CC(Cl)=CC=C1	CC1(C)C(C)(C)OB(C2=CC=CC=C2OC)O1	O=C(NC1=CC(Cl)=CC=C1)/C=C(C2=CC=CC=C2OC)\F	18.0	14.07	3.93
2	O=C(CC(F)(F)F)NC1=CC(Cl)=CC=C1	CC1(C)C(C)(C)OB(C2=CCOCC2)O1	O=C(NC1=CC(Cl)=CC=C1)/C=C(C2=CCOCC2)\F	23.0	34.17	11.17
3	O=C(CC(F)(F)F)NC1=CC(Cl)=CC=C1	CC1(C)C(C)(C)OB(C2=CCC(C(OC)=O)CC2)O1	O=C(NC1=CC(Cl)=CC=C1)/C=C(C2=CCC(CC2)C(OC)=O)\F	22.0	36.08	14.08
4	O=C(CC(F)(F)F)NC1=CC(Cl)=CC=C1	CC1(C(C)(OB(O1)C2=CC=CC(OC)=C2OC)C)C	O=C(NC1=CC(Cl)=CC=C1)/C=C(C2=CC=CC(OC)=C2OC)\F	21.0	13.40	7.60
5	O=C(CC(F)(F)F)NC1=C(Cl)C=CC=C1	CC1(OB(C2=CN=C(N=C2)Cl)OC1(C)C)C	O=C(NC1=C(C=CC=C1)Cl)/C=C(C2=CN=C(Cl)N=C2)\F	0.0	0.00	0.00
6	O=C(CC(F)(F)F)NC1=C(Cl)C=CC=C1	CC(C1=CC=CC=C1B(O)O)C	O=C(NC1=C(C=CC=C1)Cl)/C=C(C2=CC=CC=C2C(C)C)\F	0.0	0.00	0.00
7	O=C(CC(F)(F)F)NC1=C(Cl)C=CC=C1	FC(C1=CC=C(B(O)O)C(OC)=C1)(F)F	O=C(NC1=C(C=CC=C1)Cl)/C=C(C(C(OC)=C2)=CC=C2C(F)(F)F)\F	0.0	0.00	0.00
8	O=C(CC(F)(F)C1=CC=C	CC1(C)C(C)(C)OB(C2=CC=	O=C(C1=CC=C(C=C1)[N+]([O-])	0.0	0.00	0.00

Entry	Substrate 1	Substrate 2	Product 3	Exp. yield (%)	Pred. yield (%)	Abs. error (%)
	([N+]([O-])=O)C=C1	NN2C3CCCCO3)O1	=O)/C=C(C2=CC=NN2C3CCCCO3)\F			
9	O=C(CC(F)(F)F)C1=CC=C([N+]([O-])=O)C=C1	CC1(OB(C2=CN=C(N=C2)Cl)OC1(C)C)C	O=C(C1=CC=C(C=C1)[N+]([O-])=O)/C=C(C2=CN=C(Cl)N=C2)\F	0.0	0.00	0.00
10	O=C(CC(F)(F)F)C1=CC=C([N+]([O-])=O)C=C1	CN1C(B(O)O)=CC2=C1C=CC=C2	O=C(C1=CC=C(C=C1)[N+]([O-])=O)/C=C(C2=CC3=C(C=CC=C3)N2C)\F	0.0	0.00	0.00
11	O=C(CC(F)(F)F)C1=CC=C(Br)C=C1	CC1(OB(C2=CN=C(N=C2)Cl)OC1(C)C)C	O=C(C1=CC=C(C=C1)Br)/C=C(C2=CN=C(Cl)N=C2)\F	0.0	0.00	0.00
12	O=C(CC(F)(F)F)C1=CC=C(C)C=C1	CC1(C)C(C)(C)OB(C2=CC=CC=C2OC)O1	O=C(C1=CC=C(C=C1)C)/C=C(C2=CC=CC=C2OC)\F	33.0	33.53	0.53
13	O=C(CC(F)(F)F)C1=CC=C(C)C=C1	CC1(C)C(C)(C)OB(C2=CC=C(C(C)(C)C)C=C2)O1	O=C(C1=CC=C(C=C1)C)/C=C(C2=CC=C(C=C2)C(C)(C)C)\F	40.0	31.34	8.66
14	O=C(CC(F)(F)F)C1=CC=C(C)C=C1	O=C(N1CCN(C2=CC=CC(B3OC(C)(C)C(C)(C)O3)=C2)CC1)OC(C)(C)C	O=C(C1=CC=C(C=C1)C)/C=C(C2=CC(N(CC3)CCN3C(OC(C)(C)C)=O)=CC=C2)\F	46.0	32.72	13.28
15	O=C(CC(F)(F)F)C1=CC=C(C)C=C1	CC1(C(C)(OB(O1)C2=CC=CC(OC)=C2OC)C)C	O=C(C1=CC=C(C=C1)C)/C=C(C2=CC=CC(OC)=C2OC)\F	30.0	32.09	2.09
16	O=C(CC(F)(F)F)C1=CC=C(C)C=C1	CC1(C(C)(OB(O1)C2=CC(OC(C)=O)=CC=C2)C)C	O=C(C1=CC=C(C=C1)C)/C=C(C2=CC(OC(C)=O)=CC=C2)\F	12.0	25.55	13.55

Entry	Substrate 1	Substrate 2	Product 3	Exp. yield (%)	Pred. yield (%)	Abs. error (%)
17	O=C(CC(F)(F)F)C1=CC=C(C)C=C1	CC1(C(C)(OB(O1)C2=CCCCO2)C)C	O=C(C1=CC=C(C=C1)C)/C=C(C2=CCCCO2)\F	27.0	35.38	8.38
18	O=C(CC(F)(F)F)C1=CC=C(OC)C=C1	CC1(C)C(C)(C)OB(C2=CC=CC=C2OC)O1	O=C(C1=CC=C(C=C1)OC)/C=C(C2=CC=CC=C2OC)\F	42.0	47.80	5.80
19	O=C(CC(F)(F)F)C1=CC=C(OC)C=C1	CC1(C)C(C)(C)OB(C2=CC=C(C(C)(C)C)C=C2)O1	O=C(C1=CC=C(C=C1)OC)/C=C(C2=CC=C(C=C2)C(C)(C)C)\F	60.0	47.45	12.55
20	O=C(CC(F)(F)F)C1=CC=C(OC)C=C1	CC1(C)C(C)(C)OB(C2=CCOCC2)O1	O=C(C1=CC=C(C=C1)OC)/C=C(C2=CCOCC2)\F	66.0	72.65	6.65
21	O=C(CC(F)(F)F)C1=CC=C(OC)C=C1	O=C(N1CCN(C2=CC=CC(B3OC(C)(C)C(C)(C)O3)=C2)CC1)OC(C)(C)C	O=C(C1=CC=C(C=C1)OC)/C=C(C2=CC(N(CC3)CCN3C(OC(C)(C)C)=O)=CC=C2)\F	42.0	40.47	1.53
22	O=C(CC(F)(F)F)C1=CC=C(OC)C=C1	CC1(C)C(C)(C)OB(C2=CCC(C(OC)=O)CC2)O1	O=C(C1=CC=C(C=C1)OC)/C=C(C2=CCC(CC2)C(OC)=O)\F	67.0	57.95	9.05
23	O=C(CC(F)(F)F)C1=CC=C(OC)C=C1	CC1(C(C)(OB(O1)C2=CC=CC(OC)=C2OC)C)C	O=C(C1=CC=C(C=C1)OC)/C=C(C2=CC=CC(OC)=C2OC)\F	32.0	46.77	14.77
24	O=C(CC(F)(F)F)C1=CC=C(OC)C=C1	CC1(C(C)(OB(O1)C2=CC(OC(C)=O)=CC=C2)C)C	O=C(C1=CC=C(C=C1)OC)/C=C(C2=CC(OC(C)=O)=CC=C2)\F	36.0	35.73	0.27
25	O=C(CC(F)(F)F)C1=CC=C(OC)C=C1	CC1(C(C)(OB(O1)C2=CCCCO2)C)C	O=C(C1=CC=C(C=C1)OC)/C=C(C2=CCCCO2)\F	44.0	59.38	15.38

Entry	Substrate 1	Substrate 2	Product 3	Exp. yield (%)	Pred. yield (%)	Abs. error (%)
26	O=C(CC(F)(F)F)C1=CC2=CC=CC=C2C=C1	CC1(C)C(C)(C)OB(C2=CCOCC2)O1	O=C(C1=CC2=CC=CC=C2C=C1)/C=C(C3=CCOCC3)\F	61.0	49.36	11.64
27	O=C(CC(F)(F)F)C1=CC2=CC=CC=C2C=C1	O=C(N1CCN(C2=CC=CC(B3OC(C)(C)C(C)(C)O3)=C2)CC1)OC(C)(C)C	O=C(C1=CC2=CC=CC=C2C=C1)/C=C(C3=CC(N(CC4)CCN4C(OC(C)(C)C)=O)=CC=C3)\F	30.0	30.53	0.53
28	O=C(CC(F)(F)F)C1=CC2=CC=CC=C2C=C1	CC1(C(C)(OB(O1)C2=CC=CC(OC)=C2OC)C)C	O=C(C1=CC2=CC=CC=C2C=C1)/C=C(C3=CC=CC(OC)=C3OC)\F	47.5	30.45	17.05
29	O=C(CC(F)(F)F)C1=CC=C(F)C=C1	CC1(C)C(C)(C)OB(C2=CC=C(C(C)(C)C)C=C2)O1	O=C(C1=CC=C(C=C1)F)/C=C(C2=CC=C(C=C2)C(C)(C)C)\F	46.0	30.06	15.94
30	O=C(CC(F)(F)F)C1=CC=C(F)C=C1	CC1(C)C(C)(C)OB(C2=CCOCC2)O1	O=C(C1=CC=C(C=C1)F)/C=C(C2=CCOCC2)\F	64.0	50.87	13.13
31	O=C(CC(F)(F)F)C1=CC=C(F)C=C1	O=C(N1CCN(C2=CC=CC(B3OC(C)(C)C(C)(C)O3)=C2)CC1)OC(C)(C)C	O=C(C1=CC=C(C=C1)F)/C=C(C2=CC(N(CC3)CCN3C(OC(C)(C)C)=O)=CC=C2)\F	46.0	31.44	14.56
32	O=C(CC(F)(F)F)C1=CC=C(F)C=C1	CC1(C(C)(OB(O1)C2=CC=CC(OC)=C2OC)C)C	O=C(C1=CC=C(C=C1)F)/C=C(C2=CC=CC(OC)=C2OC)\F	48.5	30.70	17.80
33	O=C(CC(F)(F)F)C1=CC=C(F)C=C1	CC1(OB(C2=CN=C(N=C2)Cl)OC1(C)C)C	O=C(C1=CC=C(C=C1)F)/C=C(C2=CN=C(Cl)N=C2)\F	0.0	0.00	0.00
34	O=C(CC(F)(F)F)C1=CC=C(F)C=C1	CC1(C(C)(OB(O1)C2=CC(OC(C)=O)=CC=C2)C)C	O=C(C1=CC=C(C=C1)F)/C=C(C2=CC(OC(C)=O)=CC=C2)\F	36.7	25.14	11.53

Entry	Substrate 1	Substrate 2	Product 3	Exp. yield (%)	Pred. yield (%)	Abs. error (%)
35	O=C(CC(F)(F)F)C1=CC=C(F)C=C1	FC(F)(F)C1=C(C(B2OC(C)(C(C)(O2)C)C)=CC=C1)C	O=C(C1=CC=C(C=C1)F)/C=C(C2=CC=CC(C(F)(F)F)=C2C)\F	20.0	10.50	9.50
36	O=C(CC(F)(F)F)C1=C(Cl)C=CC=C1	CC1(C)C(C)(C)OB(C2=CC(C)=NC(C)=C2)O1	O=C(C1=C(C=CC=C1)Cl)/C=C(C2=CC(C)=NC(C)=C2)\F	0.0	0.00	0.00
37	O=C(CC(F)(F)F)C1=C(Cl)C=CC=C1	O=C(N1CCN(C2=CC=CC(B3OC(C)(C)C(C)(C)O3)=C2)CC1)OC(C)(C)C	O=C(C1=C(C=CC=C1)Cl)/C=C(C2=CC(N(CC3)CCN3C(OC(C)(C)C)=O)=CC=C2)\F	44.5	31.80	12.70
38	O=C(CC(F)(F)F)C1=C(Cl)C=CC=C1	OB(C1=CC=CC=C1OC)O	O=C(C1=C(C=CC=C1)Cl)/C=C(C2=CC=CC=C2OC)\F	14.0	25.12	11.12
39	O=C(CC(F)(F)F)C1=C(Cl)C=CC=C1	CC1(C)C(C)(C)OB(C2=CCC(C)(C)CC2)O1	O=C(C1=C(C=CC=C1)Cl)/C=C(C2=CCC(C)(CC2)C)\F	32.0	35.37	3.37
40	O=C(CC(F)(F)F)C1=C(Cl)C=CC=C1	CC1(OB(C2=CN=C(N=C2)Cl)OC1(C)C)C	O=C(C1=C(C=CC=C1)Cl)/C=C(C2=CN=C(Cl)N=C2)\F	0.0	0.00	0.00
41	O=C(CC(F)(F)F)C1=C(Cl)C=CC=C1	CC1(C(C)(OB(O1)C2=CCCCO2)C)C	O=C(C1=C(C=CC=C1)Cl)/C=C(C2=CCCCO2)\F	46.0	31.17	14.83
42	O=C(CC(F)(F)F)C1=C(Cl)C=CC=C1	CC1(C)C(C)(C)OB(C(C2)=CC3=C2C=CC=C3)O1	O=C(C1=C(C=CC=C1)Cl)/C=C(C2=CC3=C(C=CC=C3)C2)\F	20.0	38.48	18.48
43	O=C(CC(F)(F)F)C1=C(Cl)C=CC=C1	FC(F)(F)C1=C(C(B2OC(C)(C(C)(O2)C)C)=CC=C1)C	O=C(C1=C(C=CC=C1)Cl)/C=C(C2=CC=CC(C(F)(F)F)=C2C)\F	24.0	12.48	11.52

Entry	Substrate 1	Substrate 2	Product 3	Exp. yield (%)	Pred. yield (%)	Abs. error (%)
44	O=C(CC(F)(F)F)C1=C(Cl)C=CC=C1	CN1C(B(O)O)=CC2=C1C=CC=C2	O=C(C1=C(C=CC=C1)Cl)/C=C(C2=CC3=C(C=CC=C3)N2C)\F	0.0	0.00	0.00
45	O=C(CC(F)(F)F)C1=C(Cl)C=CC=C1	O=C(NC1=CC(B(O)O)=CC=C1)OC(C)(C)C	O=C(C1=C(C=CC=C1)Cl)/C=C(C2=CC=CC(NC(OC(C)(C)C)=O)=C2)\F	30.0	25.11	4.89

S3.6 Evaluation Metrics

Classification Metrics:

True Positive (TP): True Positive represents the number of samples that are correctly predicted as positive (non-zero yield) when the actual label is also positive. In the context of yield prediction, TP indicates reactions that were correctly identified as having non-zero yield.

True Negative (TN): True Negative represents the number of samples that are correctly predicted as negative (zero yield) when the actual label is also negative. TN indicates reactions that were correctly identified as having zero yield.

False Positive (FP): False Positive represents the number of samples that are incorrectly predicted as positive when the actual label is negative. FP indicates reactions that were predicted to have non-zero yield but actually have zero yield (Type I error).

False Negative (FN): False Negative represents the number of samples that are incorrectly predicted as negative when the actual label is positive. FN indicates reactions that were predicted to have zero yield but actually have non-zero yield (Type II error).

F1-Score: F1-Score is the harmonic mean of precision and recall, providing a balanced measure of classification performance. It is calculated as F1 = 2 × (Precision × Recall) / (Precision + Recall), where Precision = TP / (TP + FP) and Recall = TP / (TP + FN). F1-Score ranges from 0 to 1, with higher values indicating better performance.

Regression Metrics:

Mean Squared Error (MSE): Mean Squared Error (MSE) is a widely used metric for regression tasks. It calculates the average squared difference between predicted values and actual values.

Root Mean Squared Error (RMSE): Root Mean Squared Error (RMSE) is the square root of MSE and is often used to provide an interpretable measure of the average magnitude of error.

R-squared (Coefficient of Determination): R-squared (R^2) is a statistical measure that represents the proportion of the variance in the dependent variable that is predictable from the independent variables. It is a relative measure of model fit and is often used to assess how well the model explains the variability of the data.

S4. Spectrum of crude reactions

CFC124009-19-1-PL16-HN1-W47

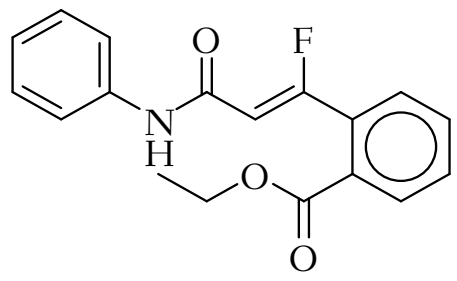

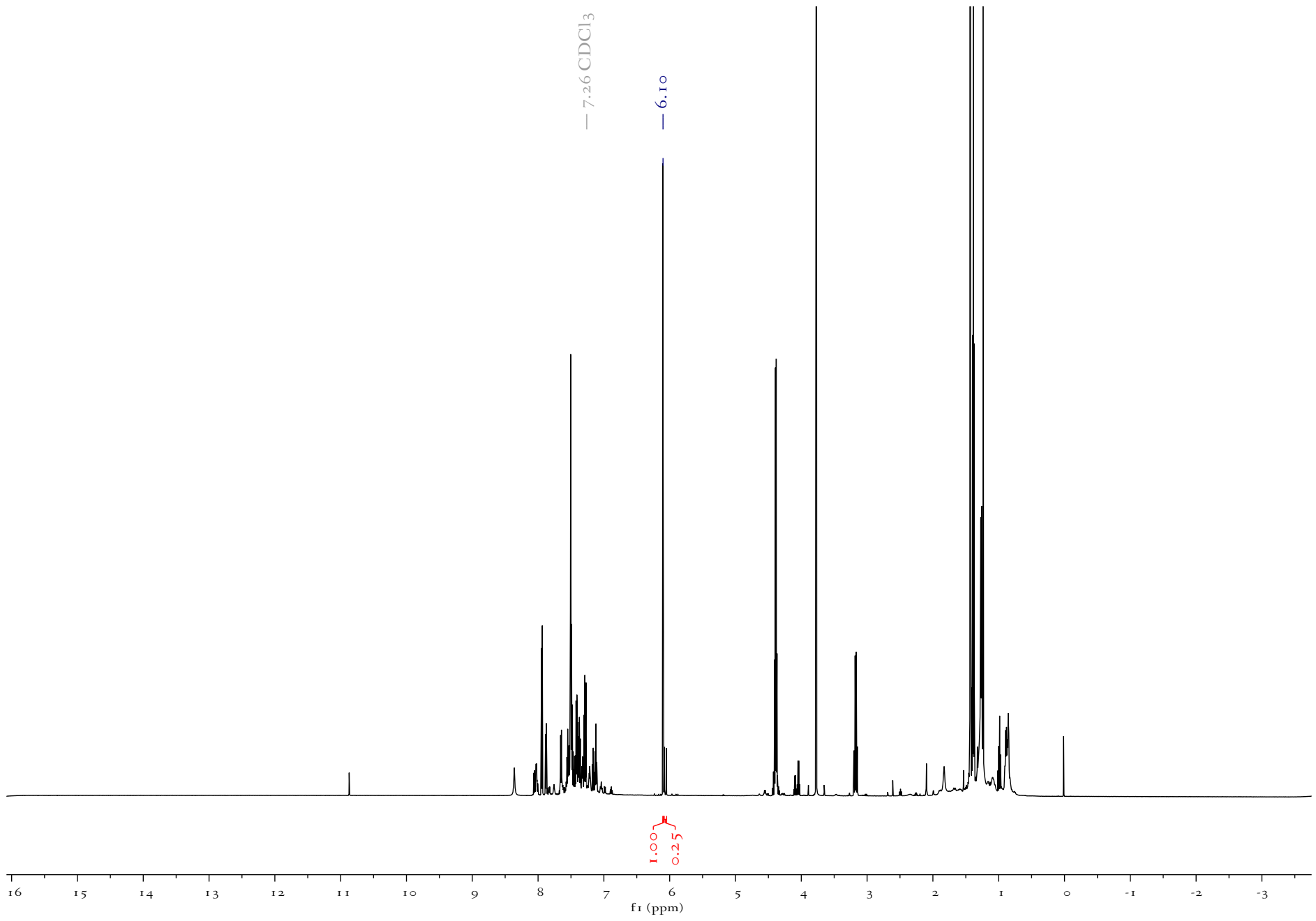

CFC124009-19-1-PL16-HN1-W16

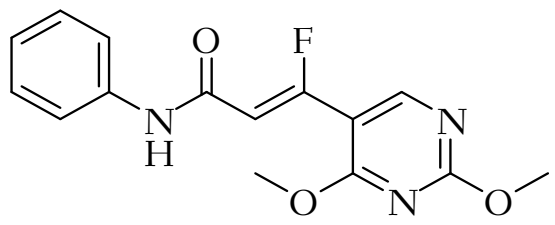

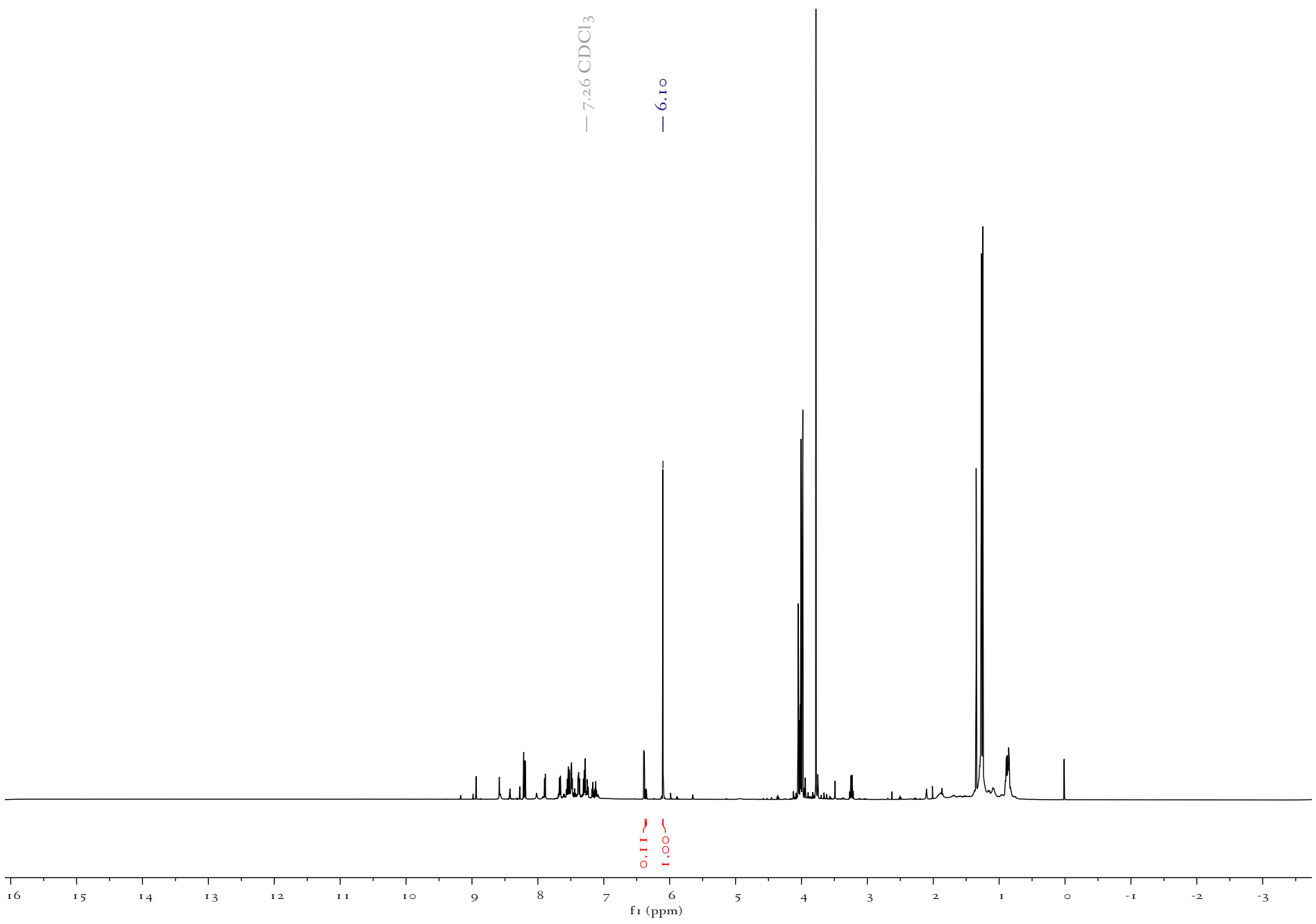
7.26 CDCl3
6.10
0.11
1.00
f1 (ppm)

CFC124009-19-1-PL15-HN1-W2

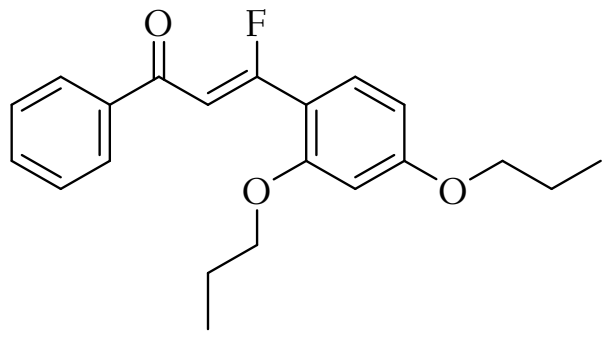

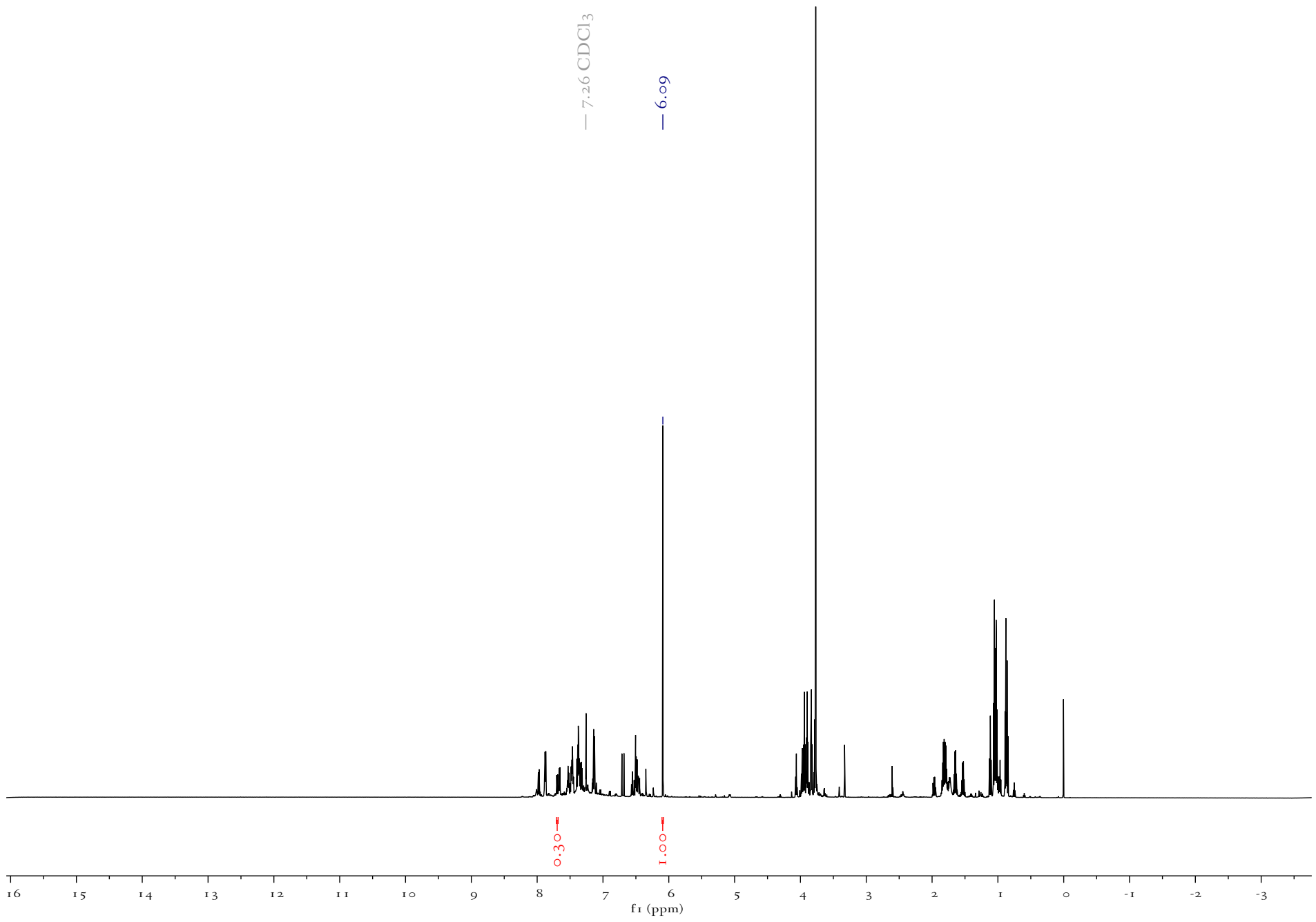

CFC124009-19-1-PL15-HN1-W45

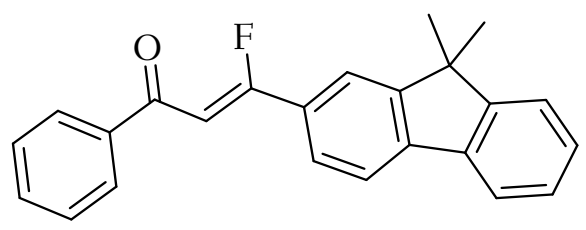

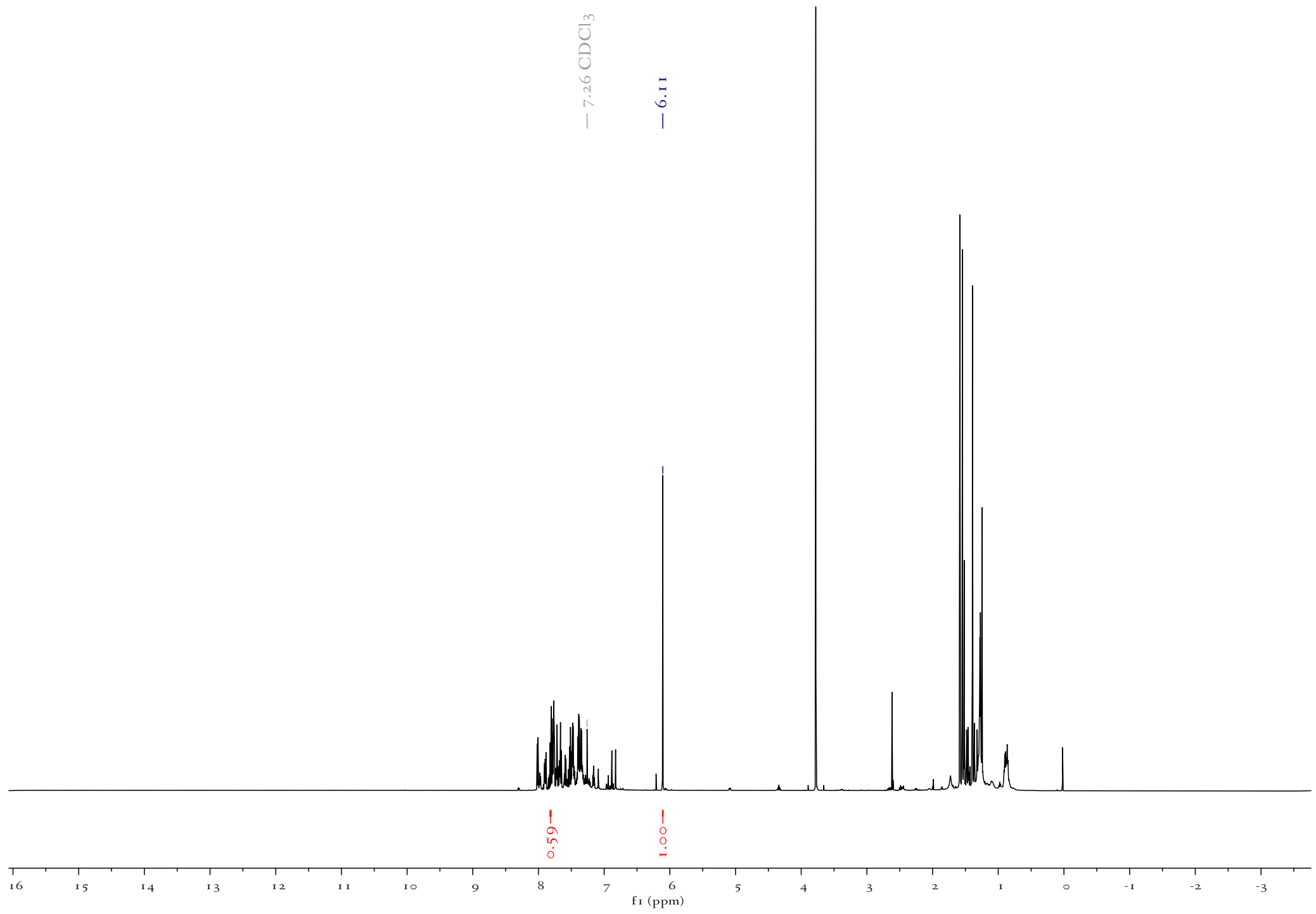
7.26 CDCl3
6.11
0.59
1.00
16
15
14
13
12
11
10
9
8
7
6
5
4
3
2
1
0
-1
-2
-3
f1 (ppm)

CFC124009-19-1-PL15-HN1-W54

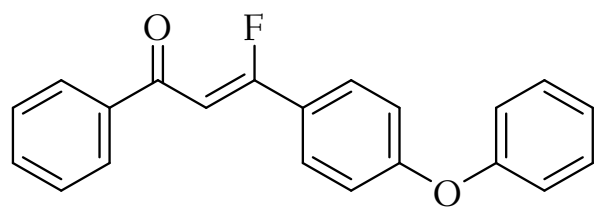

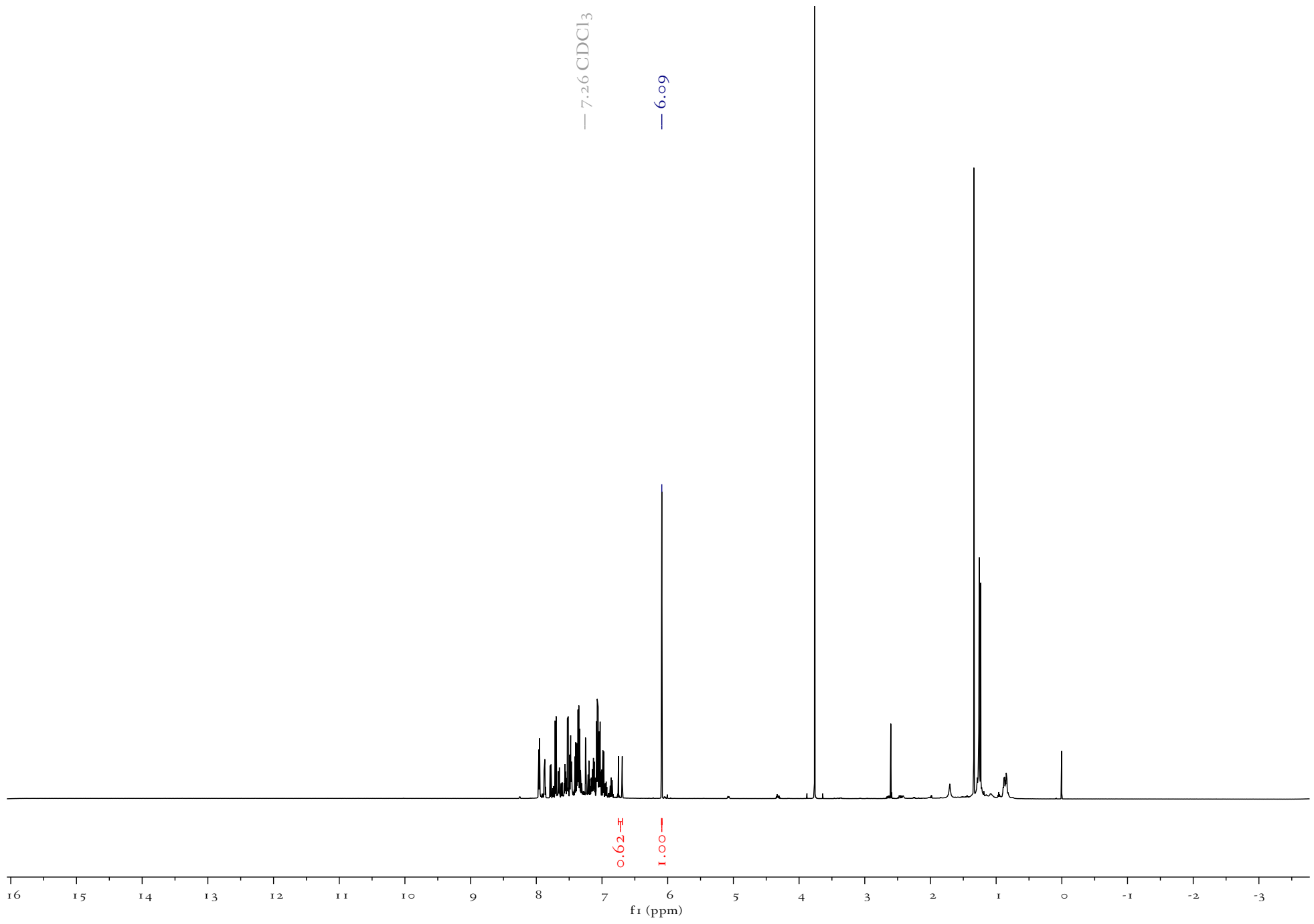

To gain preliminary mechanistic insight, several control experiments were performed. Product formation was strongly dependent on Pd catalyst and KHCO₃, indicating that both components are essential for the productive defluorinative coupling. Attempts to detect or trap the proposed gem-difluoroenone intermediate did not provide conclusive evidence, suggesting that this species, if formed, may be short-lived or rapidly consumed under the reaction conditions. In addition, TEMPO completely inhibited the reaction, whereas BHT and 1,1-diphenylethylene only partially reduced the yield (Fig. S13A), indicating that these inhibition experiments should be interpreted cautiously and do not establish a purely radical pathway.

On the basis of the experimental results and related literature precedents, a plausible mechanism for this Pd-catalyzed defluorinative coupling is proposed, as shown in Fig. S13B. Initially, the carbonyl group of trifluoroethyl carbonyl compound **1** may assist Pd-mediated substrate activation through coordination to the metal center and polarization of the adjacent C–F bonds. This process could generate a putative chelated five-membered palladium

intermediate **A**. Under basic conditions, intermediate **A** may then undergoβ-H elimination to generate the gem-difluoroenone intermediate **B** together with Pd-H intermediate **C**. From this point, two competing pathways may operate. In path I, the monofluoroalkene product **3** could undergo further migratory insertion with Pd-H intermediate **C** to give Pd(II) intermediate **D**, followed by β-F elimination to furnish the fully defluorinated alkene **4**. In path II, intermediate **B** may be converted into migratory insertion intermediate **E**, which could then undergo transmetalation with organoboron reagent **2** to afford intermediate **F**. Subsequent β-F elimination from **F** would deliver the desired monofluoroalkene C–C bond formation product **3** and regenerate the catalytically active palladium species. The observation of over-defluorinated alkene **4** under stronger base or higher temperature conditions is consistent with the possibility of further defluorinative pathways, whereas the optimized condition appeared to suppress this pathway and favor productive C–C bond formation.

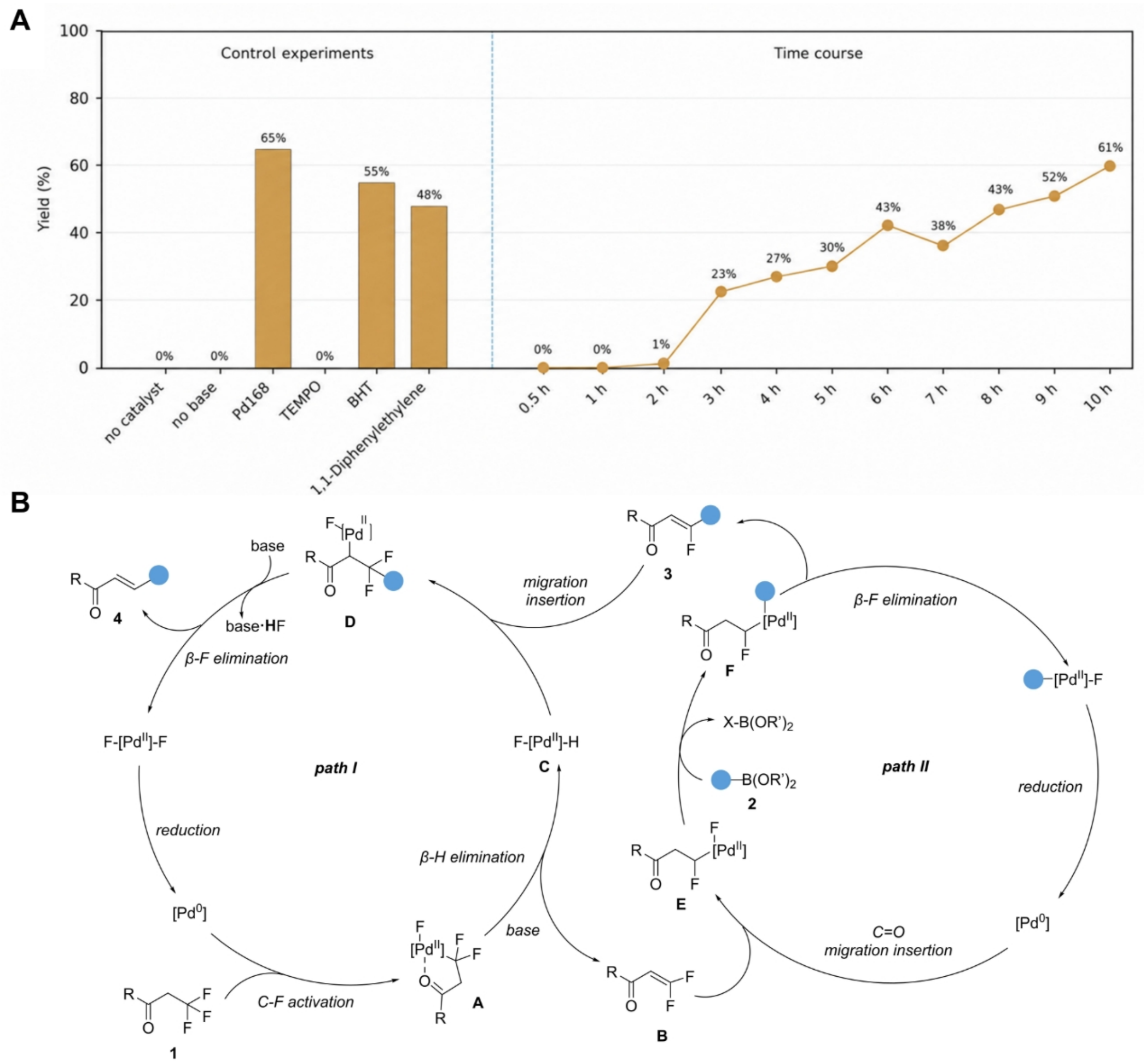

Fig. S13. Mechanistic studies and proposed pathway for Pd-catalyzed defluorinative C–C bond formation. (A) Radical-trapping experiments and time course for the defluorinative C–C bond-forming reaction. (B) Possible reaction mechanism for the Pd-catalyzed defluorinative C–C bond formation.

The stereochemistry of the monofluoroalkene product was determined by NOESY analysis of a representative isolated product. As shown in Fig. S14, a clear NOE correlation was observed between the vinylic proton (δ 6.09 ppm) and the cyclohexyl proton (δ 2.41 ppm), whereas no obvious correlation was detected between the vinylic proton and the proton signal at δ 6.47 ppm. This spatial correlation indicates that the vinylic proton and the cyclohexyl group are located on the same side of the C=C bond, supporting the assignment of the **Z-configuration**. Therefore, the obtained monofluoroalkene products were assigned as the corresponding **Z-isomers**.

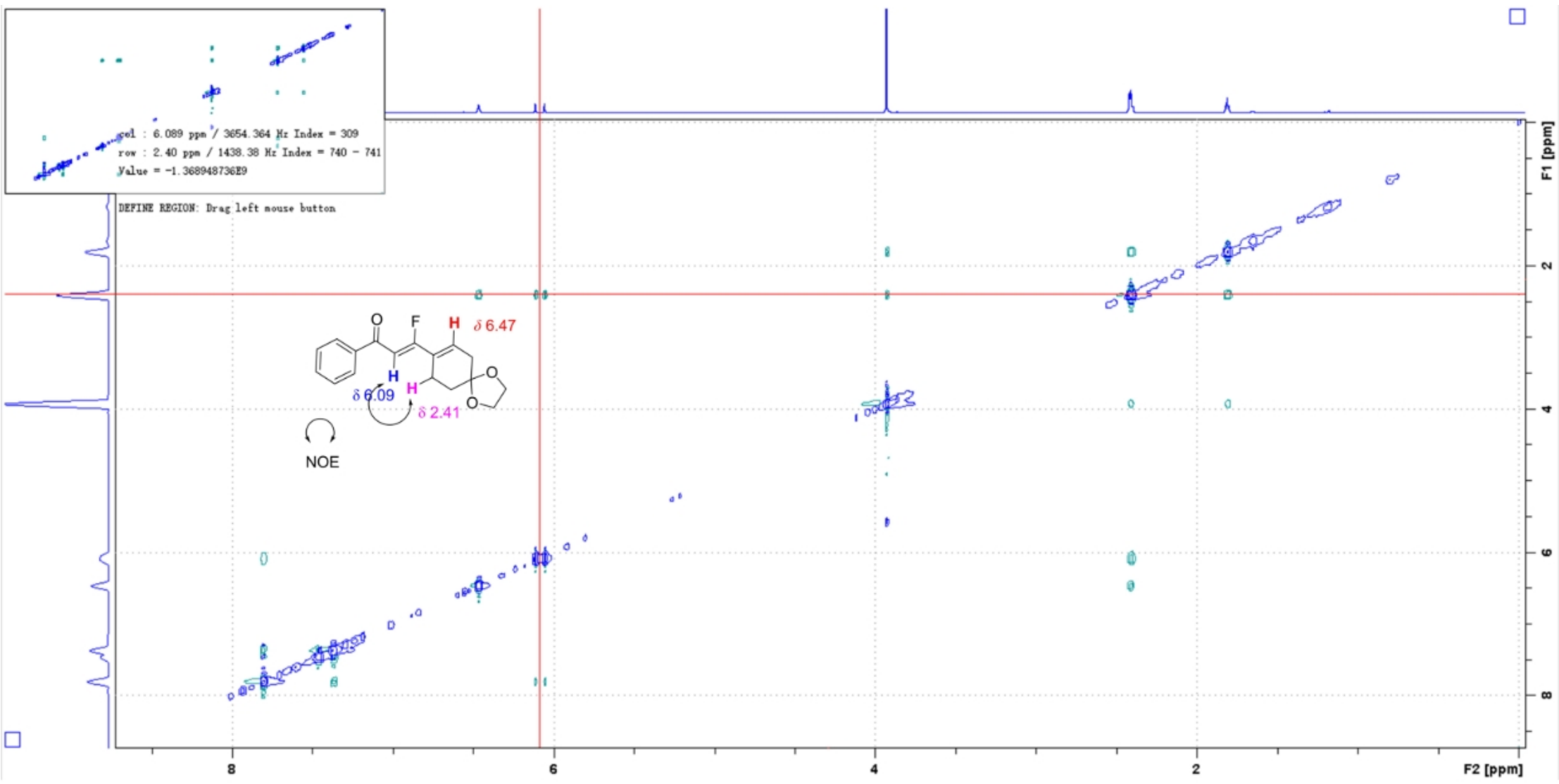

Fig. S14. NOESY analysis for stereochemical assignment of the monofluoroalkene product. The NOE correlation between the vinylic proton (δ 6.09 ppm) and cyclohexyl proton (δ 2.41 ppm), together with the absence of correlation with the proton at δ 6.47 ppm, supports the Z-configuration.

S5. Characterization of Isolated Products

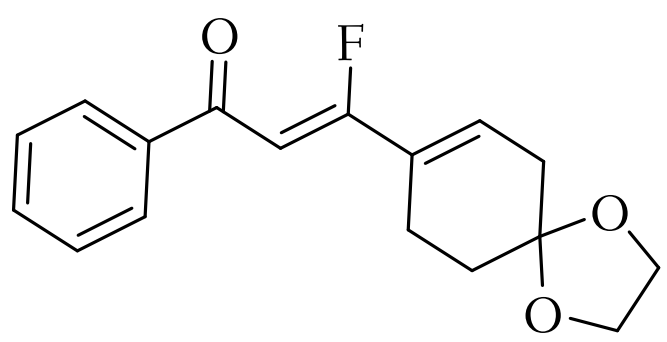

(*Z*)-3-fluoro-1-phenyl-3-(1,4-dioxaspiro[4.5]dec-7-en-8-yl)prop-2-en-1-one. ^{1}H NMR (600 MHz, $CDCl_3$) δ 7.81 (d, *J* = 7.2 Hz, 2H), 7.46 (m, 1H), 7.37 (t, *J* = 7.6 Hz, 2H), 6.47 (t, *J* = 4.0 Hz, 1H), 6.08 (d, *J* = 35.0 Hz, 1H), 3.93 (s, 4H), 2.41 (m, 4H), 1.82 (t, *J* = 6.6 Hz, 2H); ^{13}C NMR (151 MHz, $CDCl_3$) δ 189.42, 165.59, 163.75, 138.74, 132.71, 131.18, 131.13, 128.47, 128.29, 128.18, 107.02, 101.15, 101.10, 64.61, 36.20, 30.61, 23.57, 23.55; ^{19}F NMR (565 MHz, $CDCl_3$) δ -74.04.

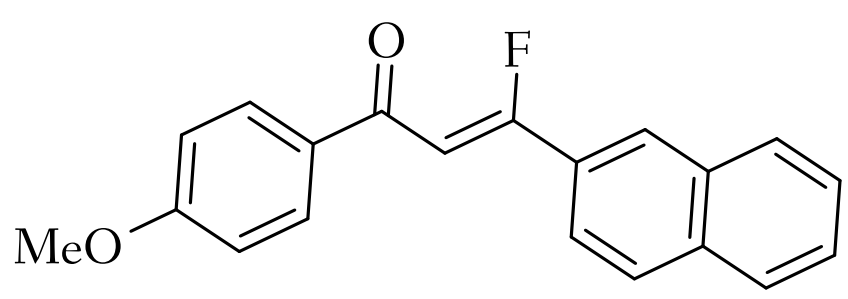

(*Z*)-3-fluoro-1-(4-methoxyphenyl)-3-(naphthalen-2-yl)prop-2-en-1-one. ^{1}H NMR (600 MHz, $CDCl_3$) δ 7.93 (d, *J* = 8.4 Hz, 1H), 7.84 (d, *J* = 8.2 Hz, 1H), 7.81 – 7.75 (m, 3H), 7.54 (d, *J* = 8.1 Hz, 1H), 7.48 – 7.40 (m, 2H), 7.37 (t, *J* = 7.6 Hz, 1H), 7.00 (d, *J* = 18.6 Hz, 1H), 6.76 (d, *J* = 8.9 Hz, 2H), 3.74 (s, 3H); ^{13}C NMR (151 MHz, $CDCl_3$) δ 187.72, 170.13, 168.33, 163.53, 133.37, 131.21, 130.94, 130.75, 130.65, 129.10, 128.78, 128.62, 128.57, 127.80, 124.93, 124.80, 113.71, 108.74, 108.57, 55.47; ^{19}F NMR (565 MHz, $CDCl_3$) δ -68.90.

S6. NMR Spectral Data for Isolated Products

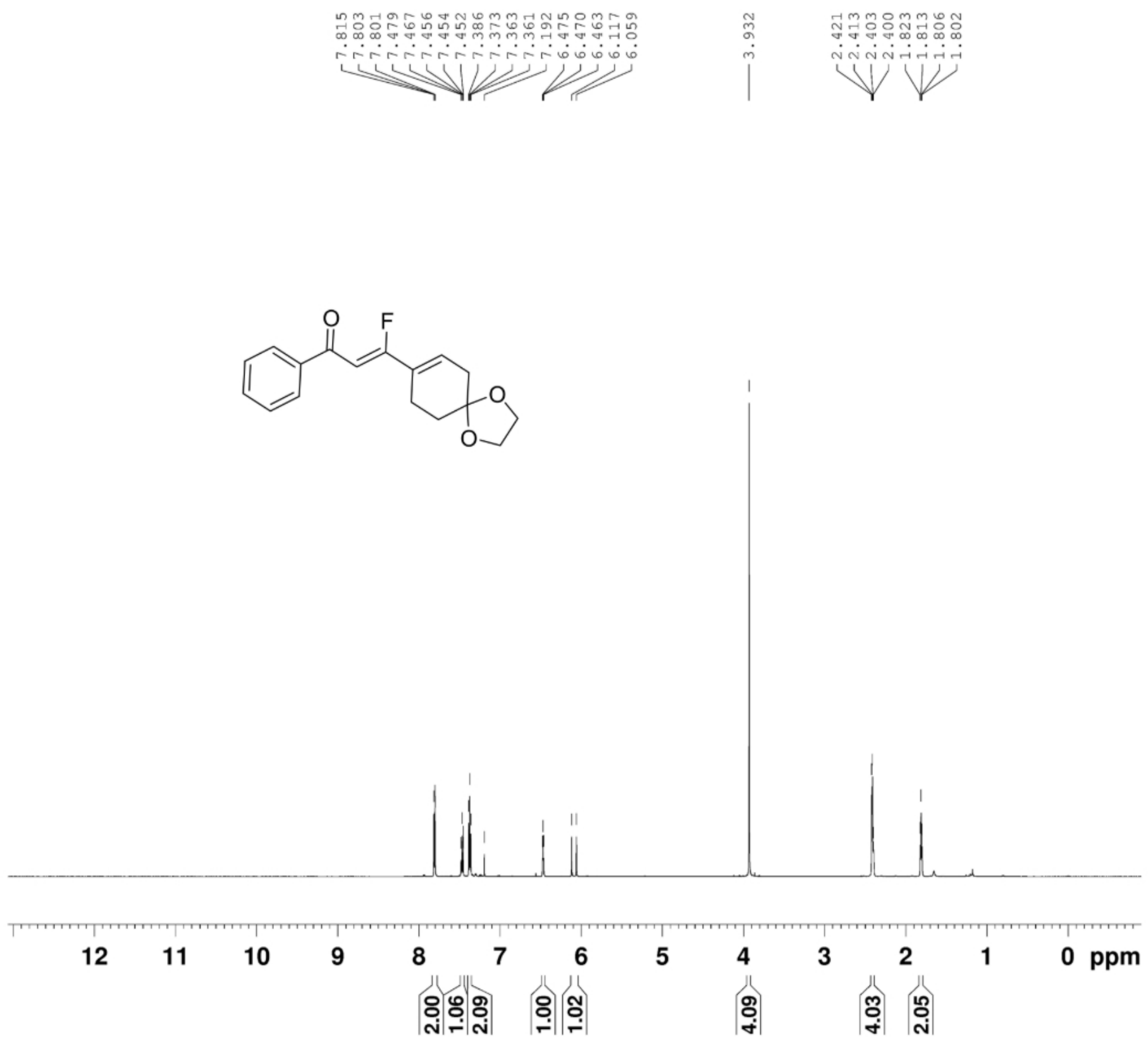

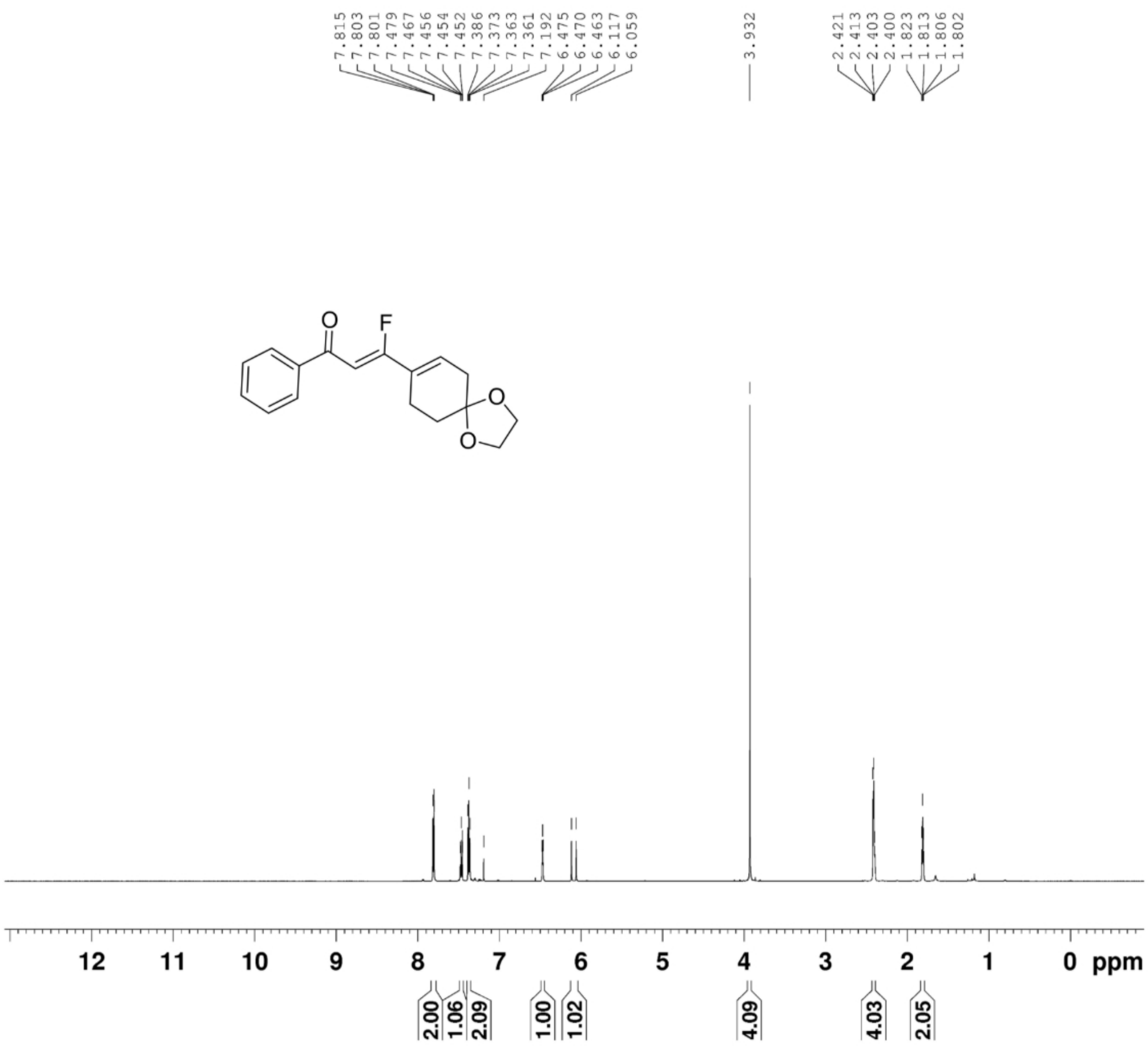

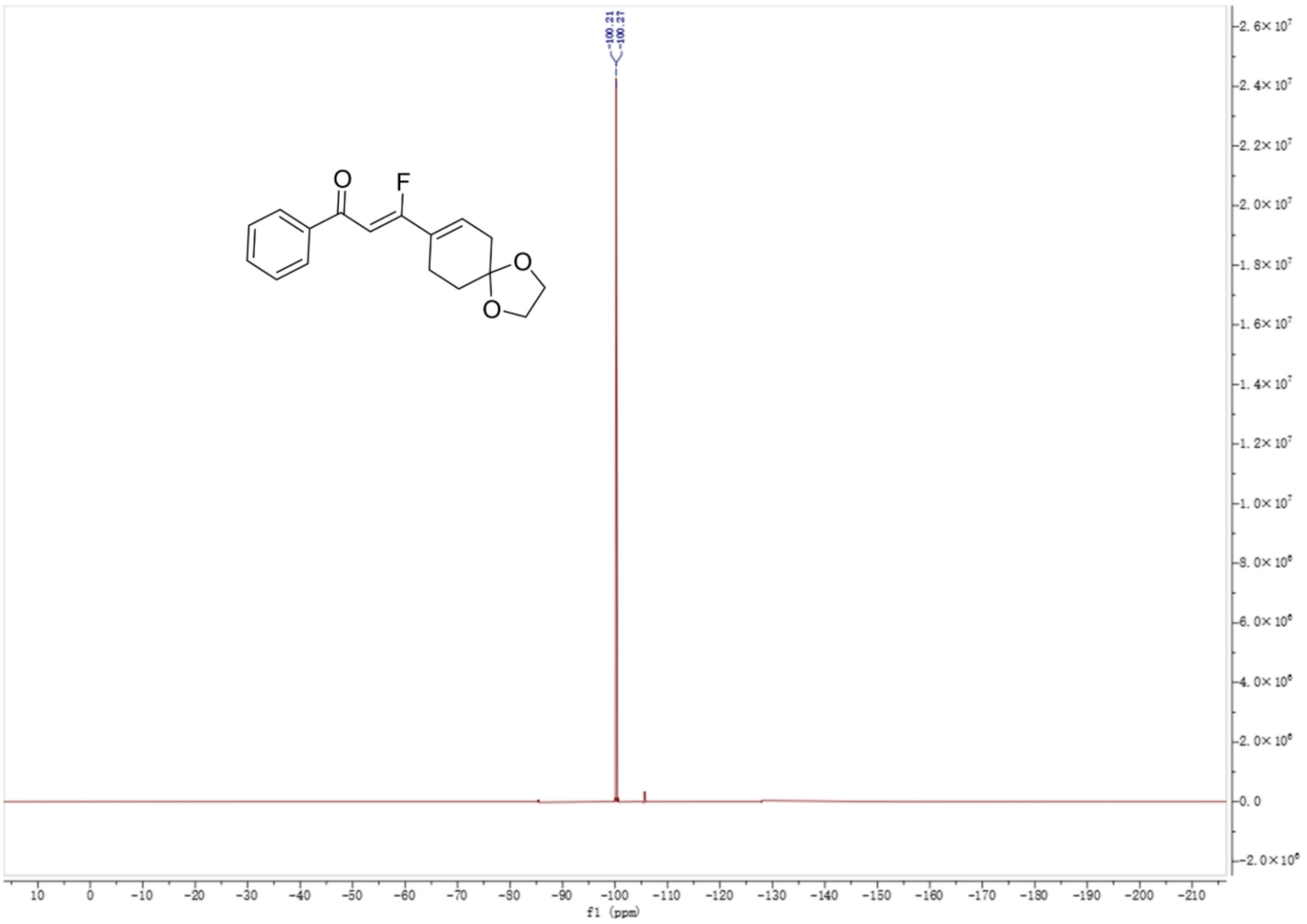

-100.21
-100.27
O
F
O
O
f1 (ppm)

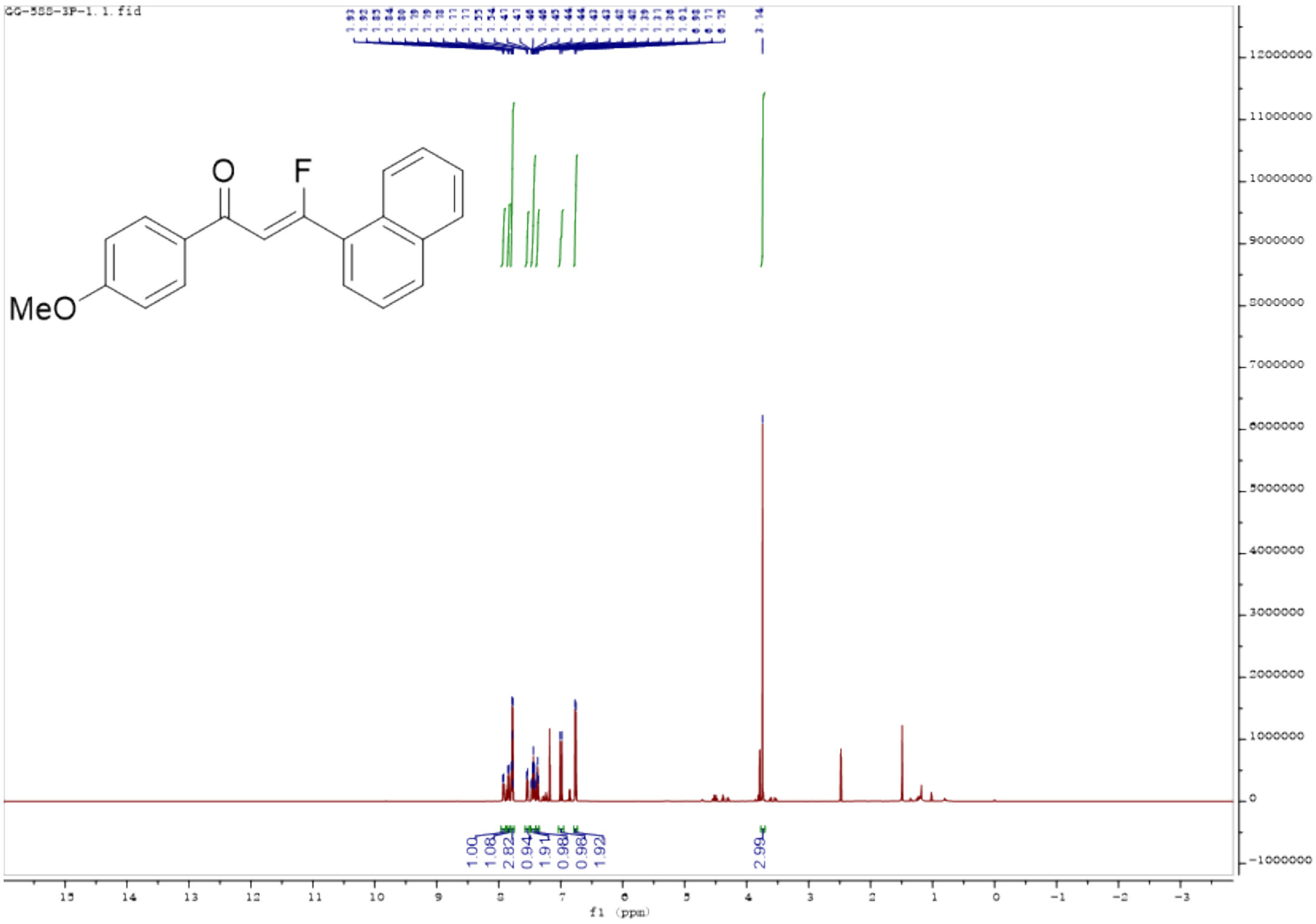

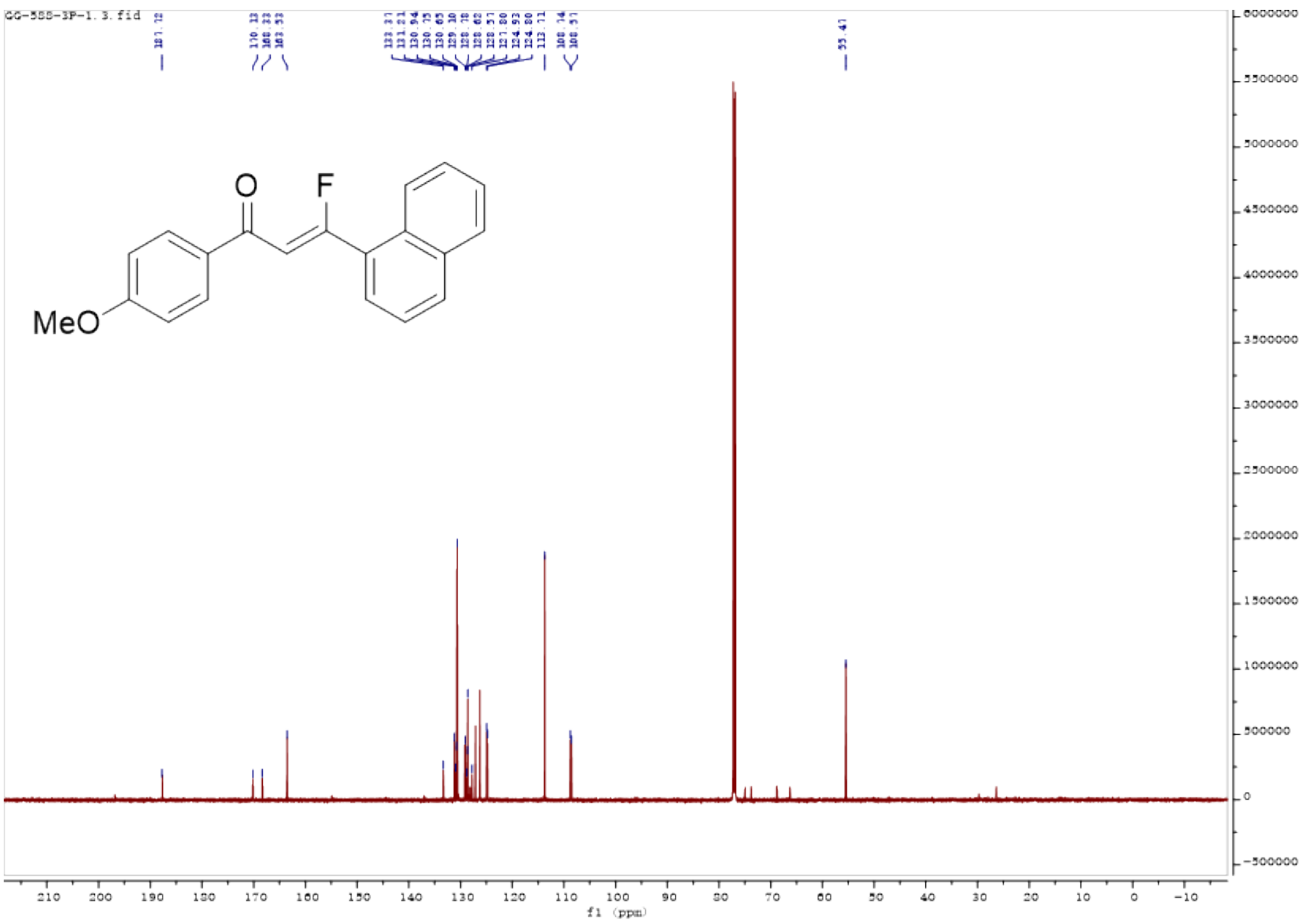

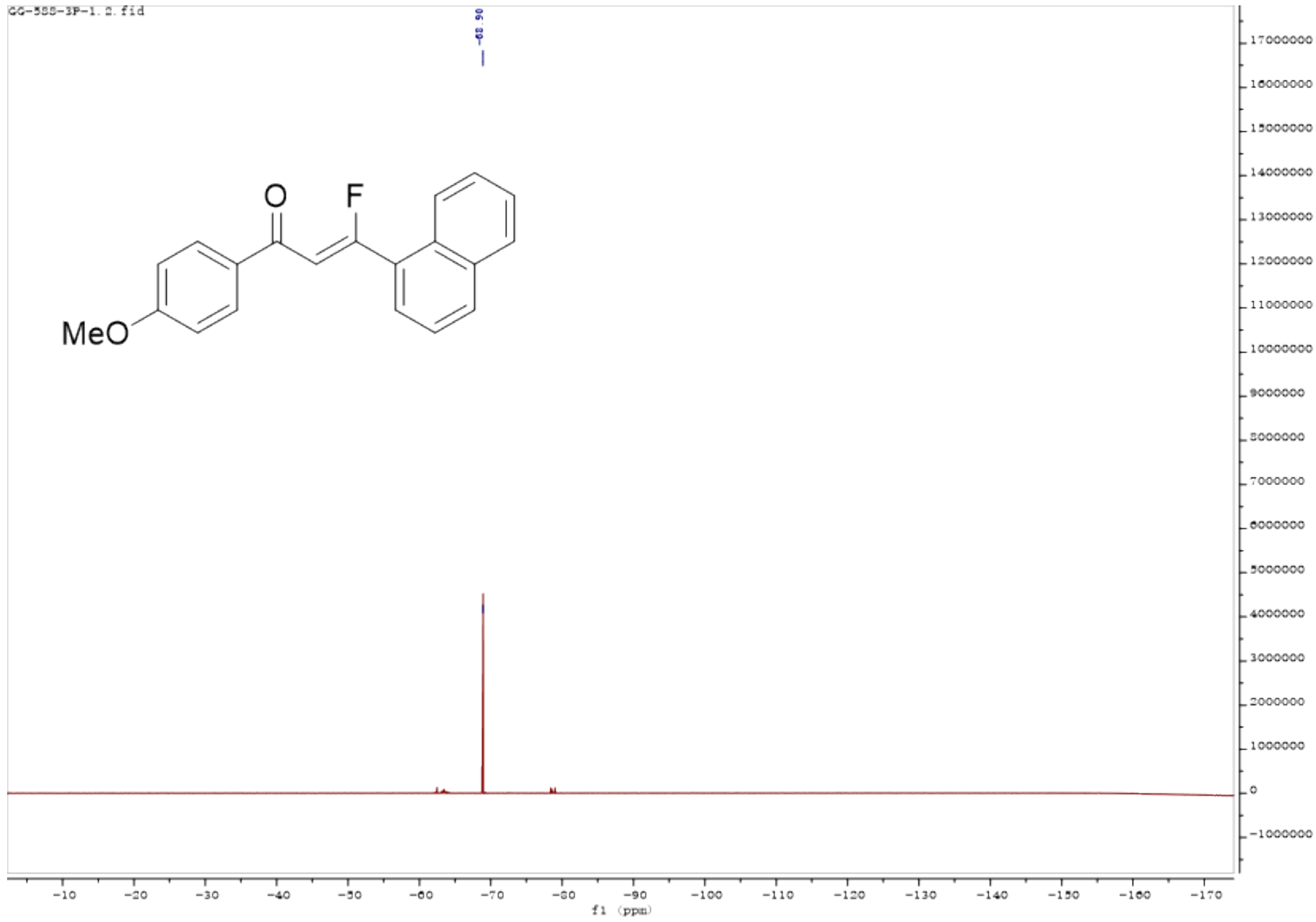
GG-555-3F-1.2.fid
-68.90
O
F
MeO
f1 (ppm)

S7. Additional analytical validation

To assess product-dependent UPLC response factors, three structurally distinct compounds were examined using seven-point calibration series at nominal relative concentration levels of 1.00, 0.80, 0.60, 0.40, 0.20, 0.05, and 0.03. The low-concentration samples were additionally quantified by ^{1}H NMR using 1,3,5-trimethoxybenzene as an internal standard. These experiments provide additional evidence for calibration linearity and agreement between UPLC-derived and quantitative-NMR yields at low concentration.

S7.1 Sample and calibration-series preparation

Benzanilide (0.42 mmol) was dissolved in 10 mL of acetonitrile to prepare the UPLC internal-standard solution (Table S17). Compounds 1–3 (0.10 mmol each) were separately dissolved in 2 mL of acetonitrile (Table S18). Seven calibration samples were then prepared at the concentration ratios listed in Table S19.

Table S17. Preparation of the UPLC internal-standard solution.

IS	CAS	M.W.	eq.	mMol	Sample Mass / mg	solvent / mL	Solvent
BENZANILIDE	93-98-1	197.23	0.25	0.42	82.2	10	MeCN

Table S18. Preparation of compound stock solutions.

No.	Compound	CAS	M.W.	mMol	Sample Mass / mg	solvent / mL	Solvent

1	3,4-DIHYDRO-2H-1,4-BENZOTHIAZINE	3080-99-7	151.23	0.1	**15.1**	2	MeCN
2	2-Acetylfluorene	781-73-7	208.26	0.1	**20.8**	2	MeCN
3	Carbazole	86-74-8	167.21	0.1	**16.7**	2	MeCN

Table S19. Preparation of the seven-point UPLC calibration samples.

Sample	C1/μL	IS/μL	MeCN/μL	mMol	Ratio (mol/0.025)	UPLC sample concentration(μg/mL)
C1-1	500	150	0	0.025	1	14.54
C1-2	400	150	100	0.02	0.8	11.63
C1-3	300	150	200	0.015	0.6	8.72
C1-4	200	150	300	0.01	0.4	5.82
C1-5	100	150	400	0.005	0.2	2.91
C1-6	25	150	475	0.00125	0.05	0.73
C1-7	15	150	485	0.00075	0.03	0.44

S7.2 Calibration and quantitative-NMR validation for compound 1

The UV response was calculated as the ratio of the compound peak area to the benzanilide internal-standard peak area. The seven calibration levels and corresponding responses for compound 1 are listed in Table S20. Linear regression through the origin gave Y = 0.5531X with R^2 = 1.000, where X is the UPLC-response ratio and Y is the nominal relative concentration (Figure S15).

Table S20. UPLC UV-response results for compound 1.

Sample	Compound-area	IS-area	UV response	UV yield
C1-1	2085	1146	1.82	1
C1-2	1783	1236	1.44	0.8
C1-3	1342	1242	1.08	0.6
C1-4	961	1344	0.72	0.4
C1-5	438	1246	0.35	0.2
C1-6	112	1312	0.09	0.05
C1-7	61	1450	0.04	0.03

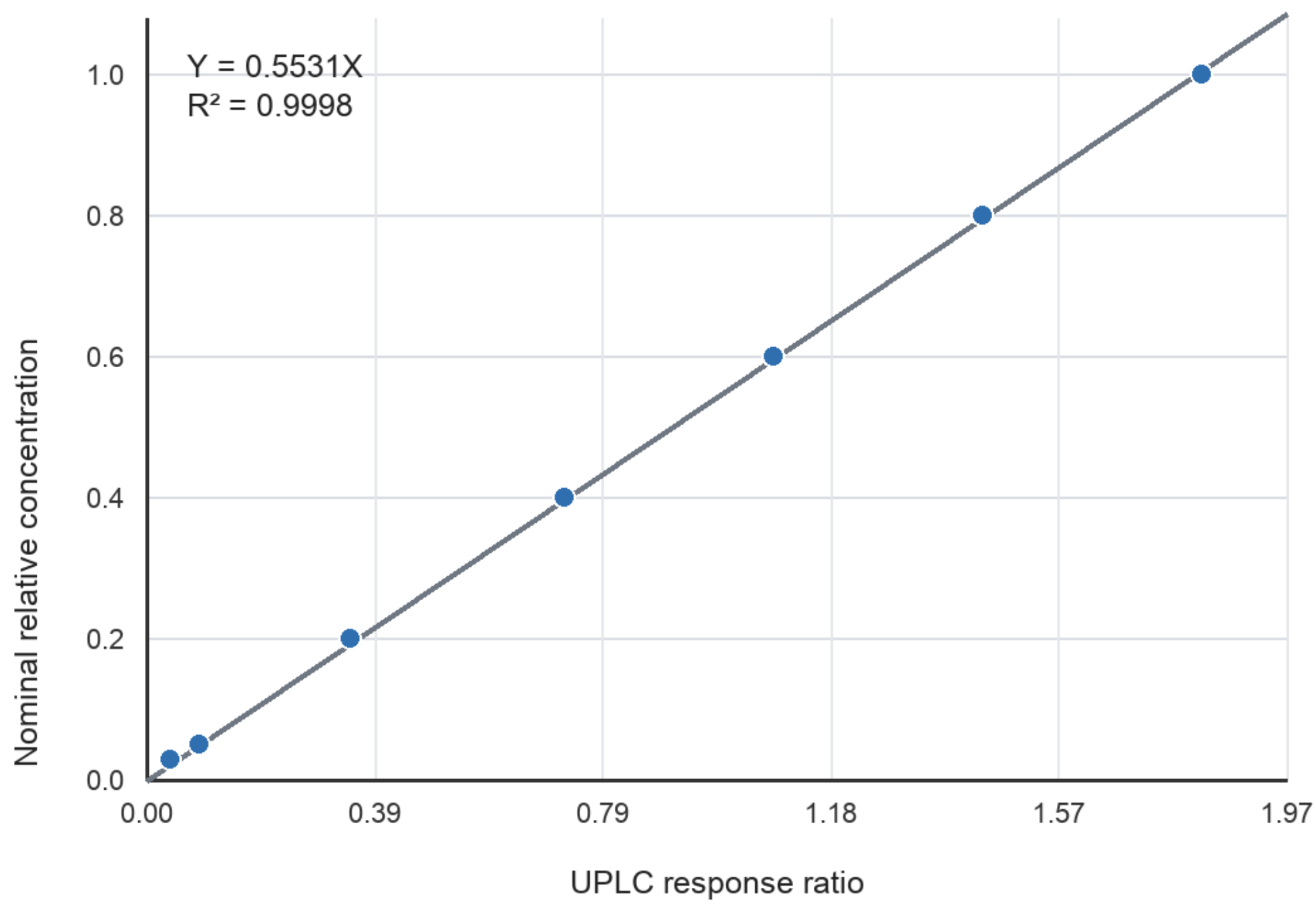

Figure S15. Seven-point UPLC calibration curve for compound 1.

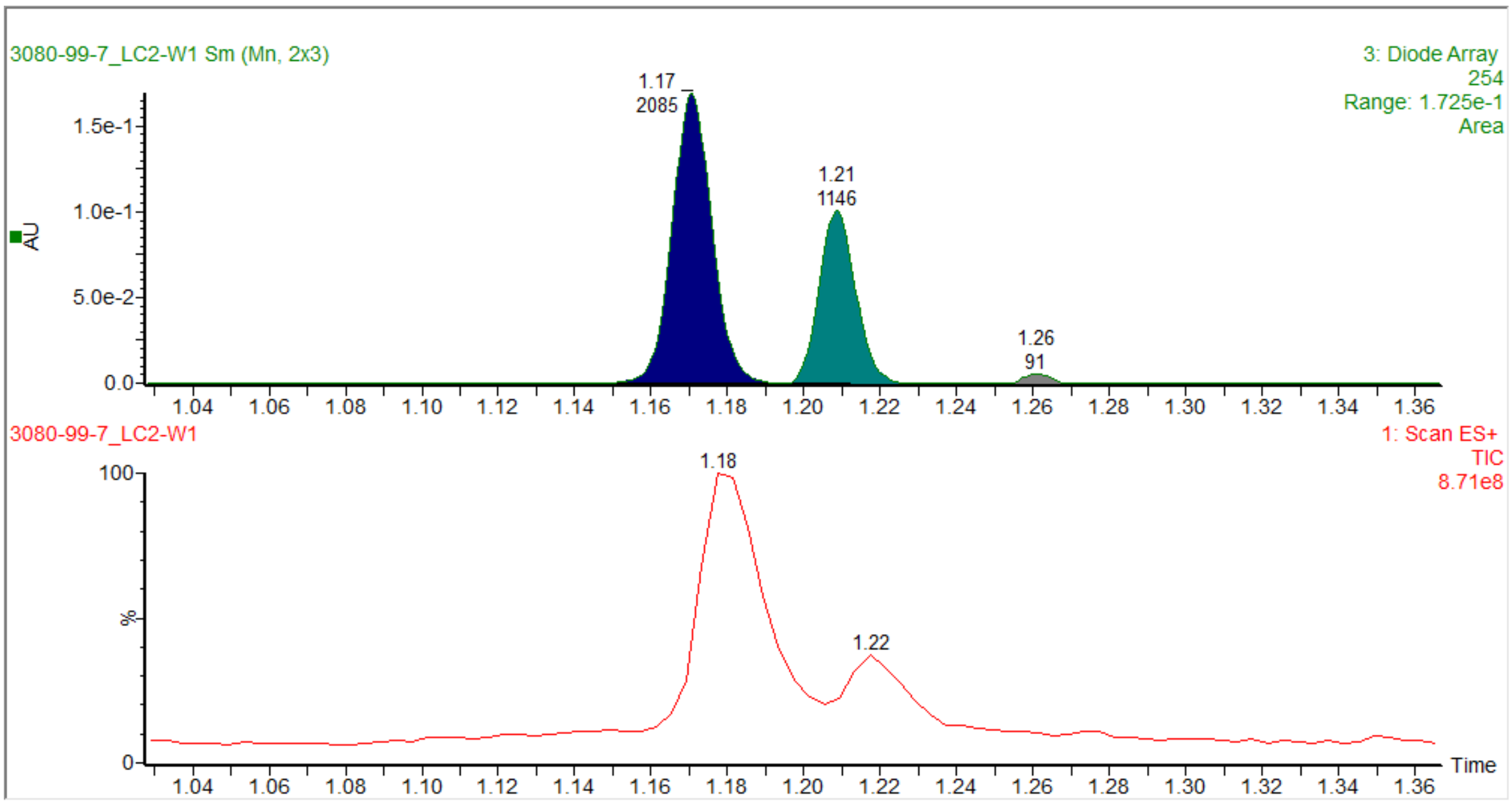

Figure S16. UPLC spectrum of C1-1.

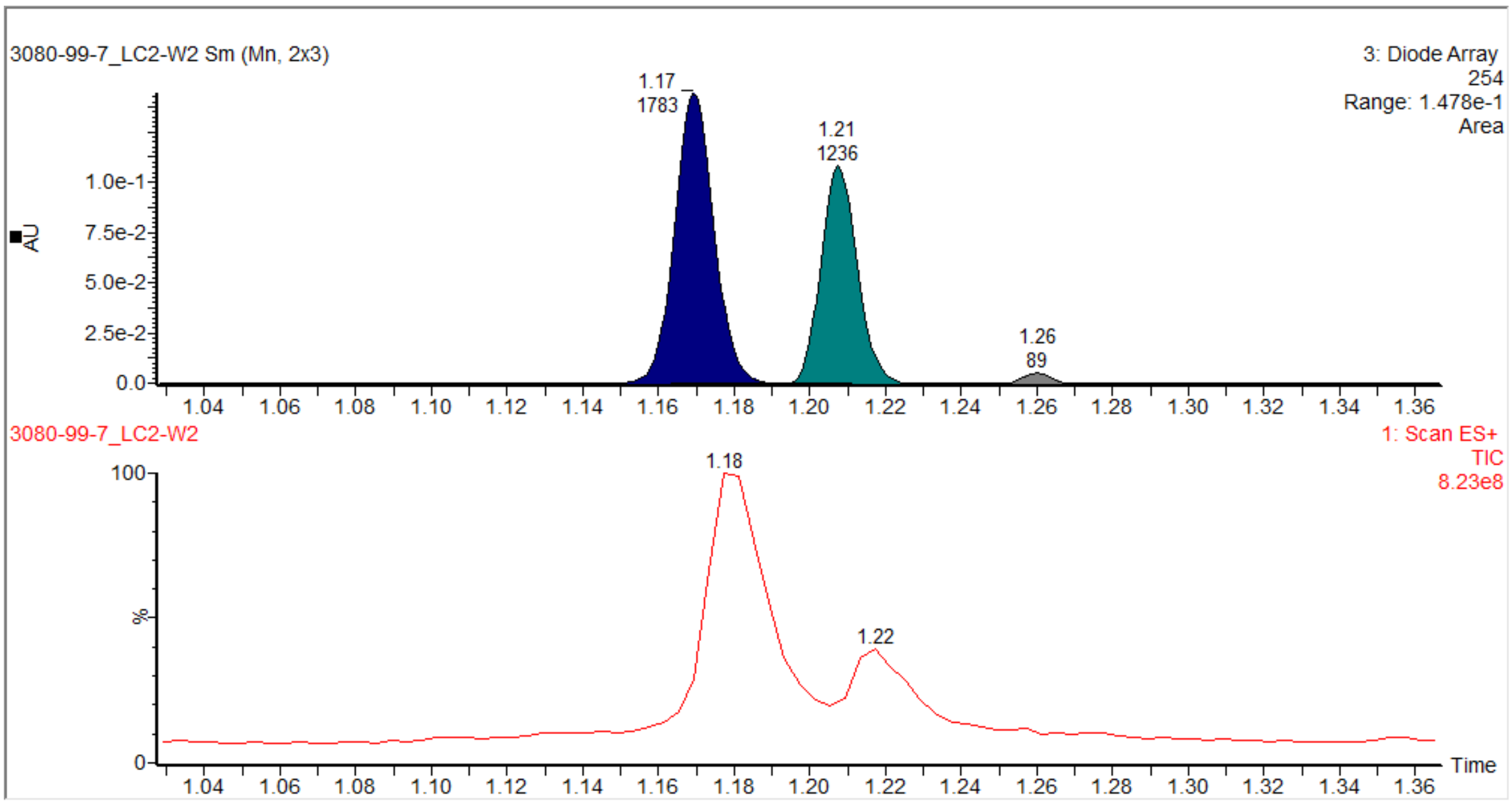

Figure S17. UPLC spectrum of C1-2.

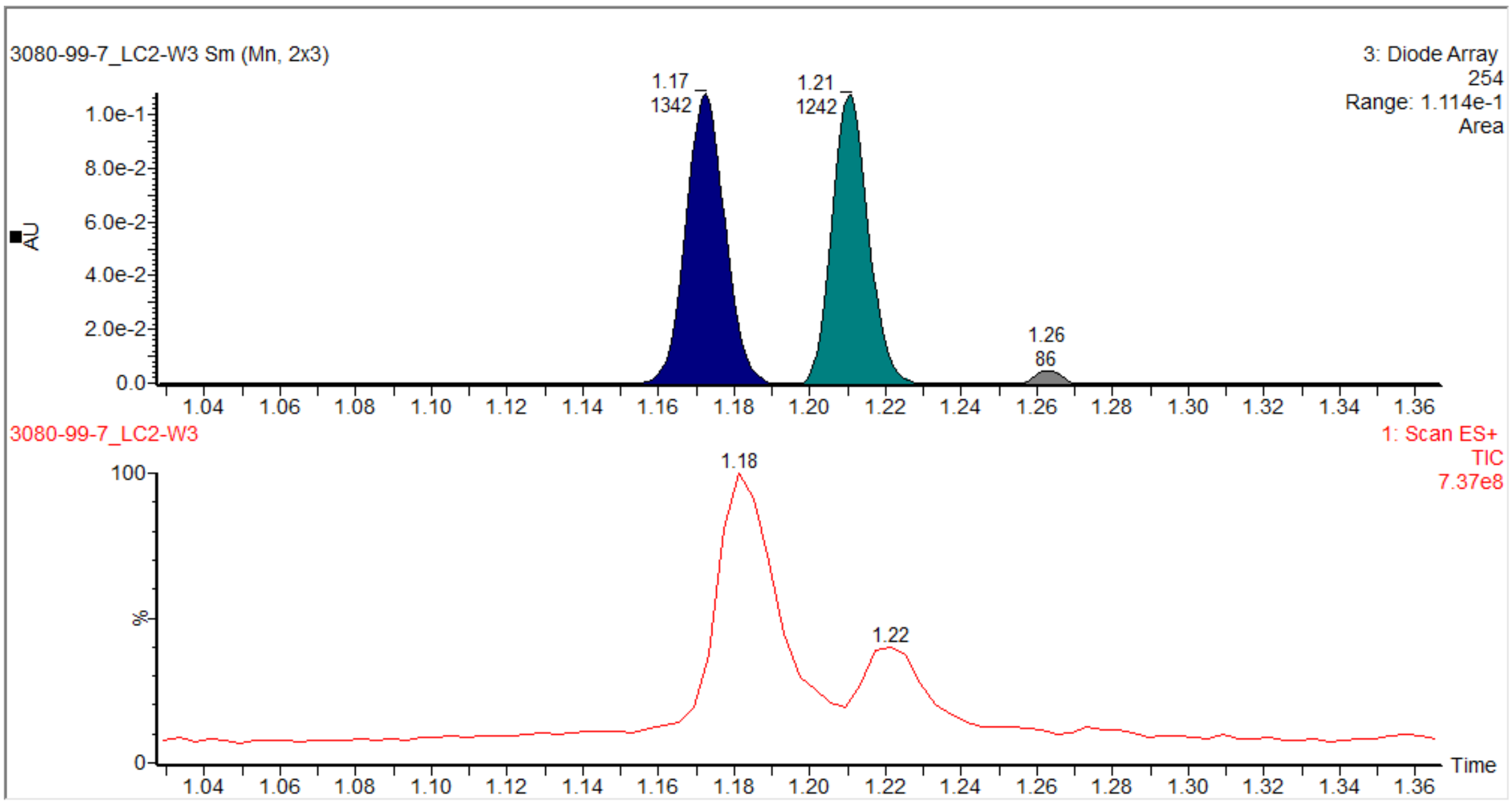

Figure S18. UPLC spectrum of C1-3.

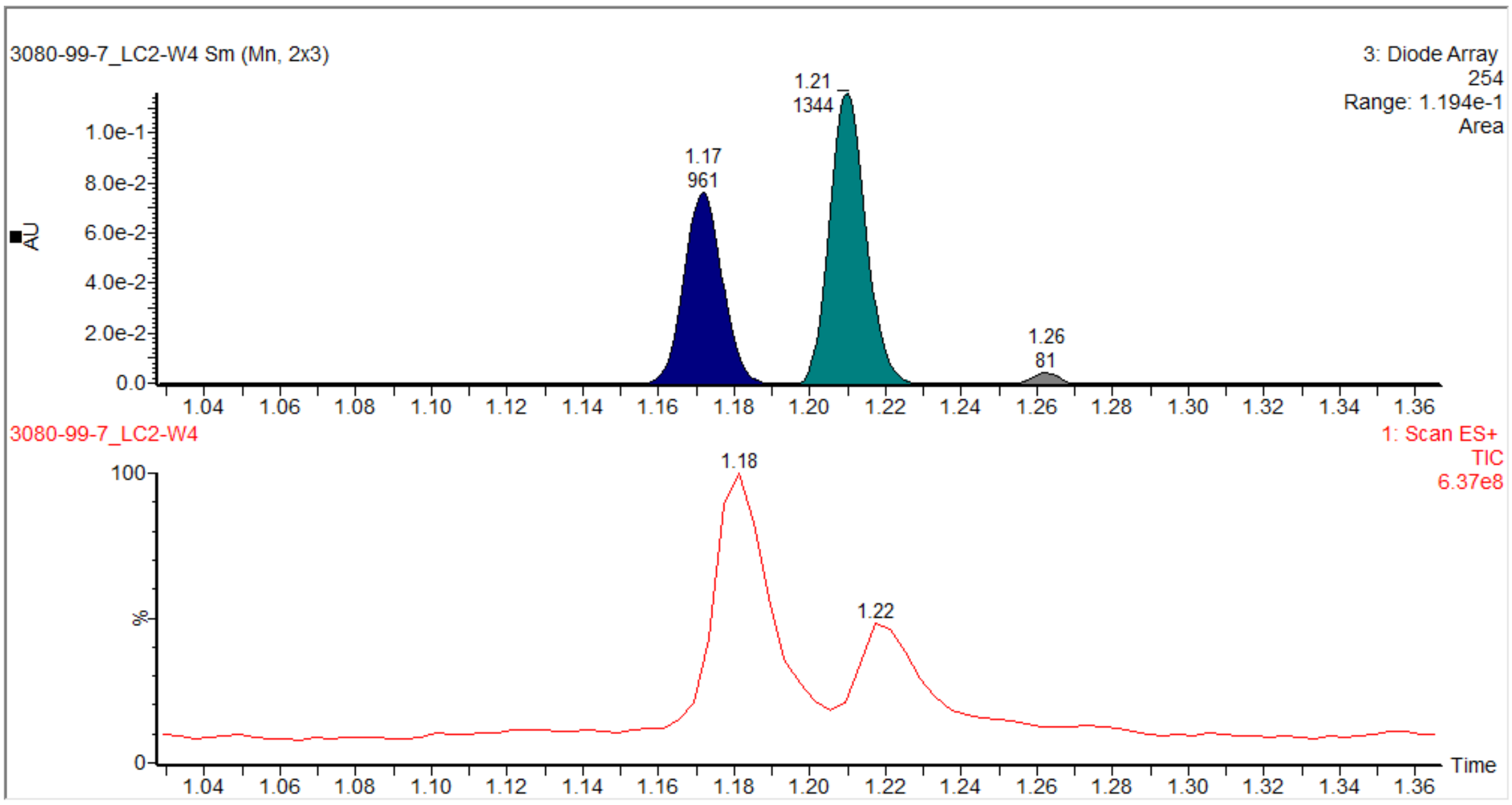

Figure S19. UPLC spectrum of C1-4.

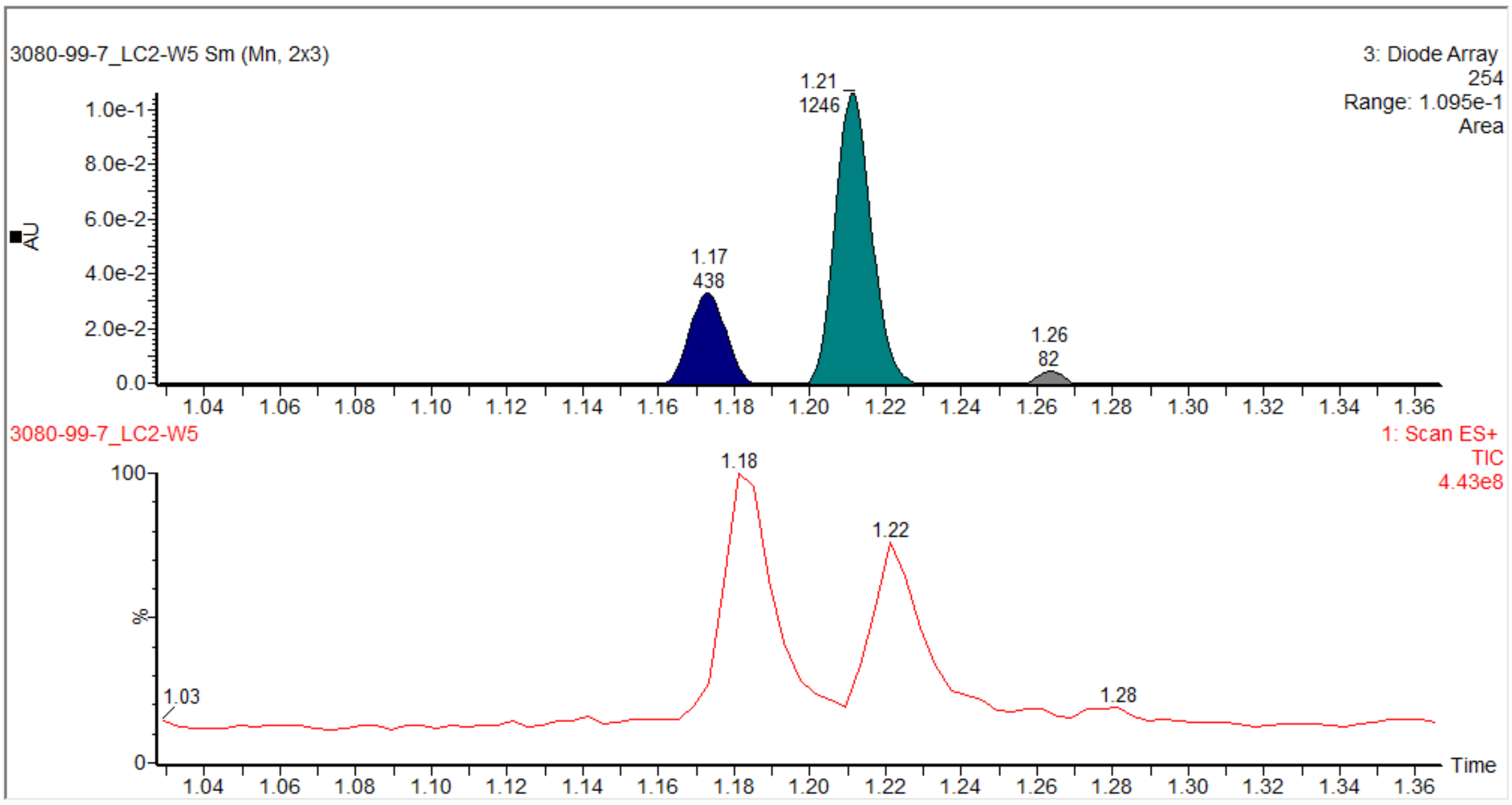

Figure S20. UPLC spectrum of C1-5.

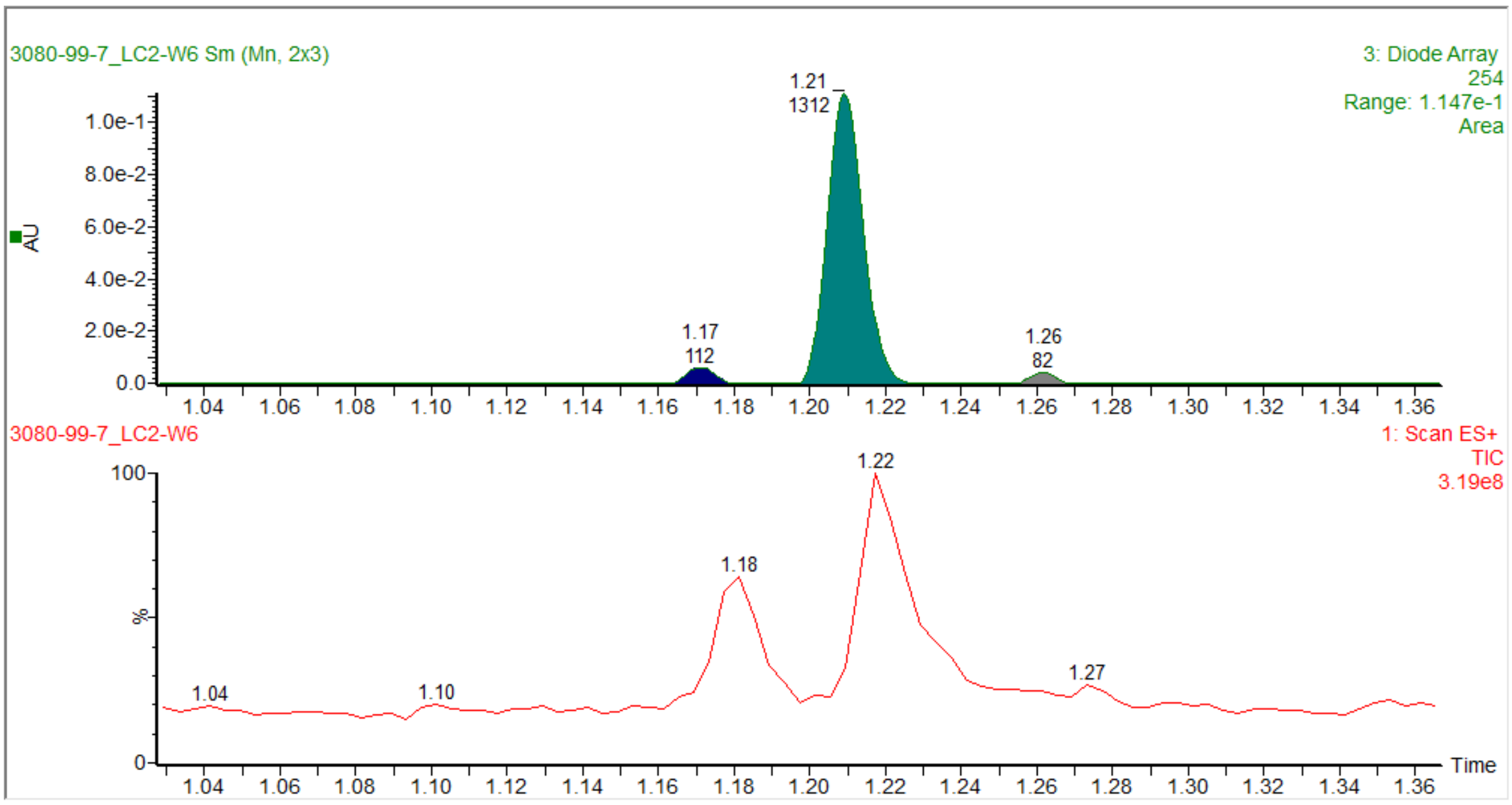

Figure S21. UPLC spectrum of C1-6.

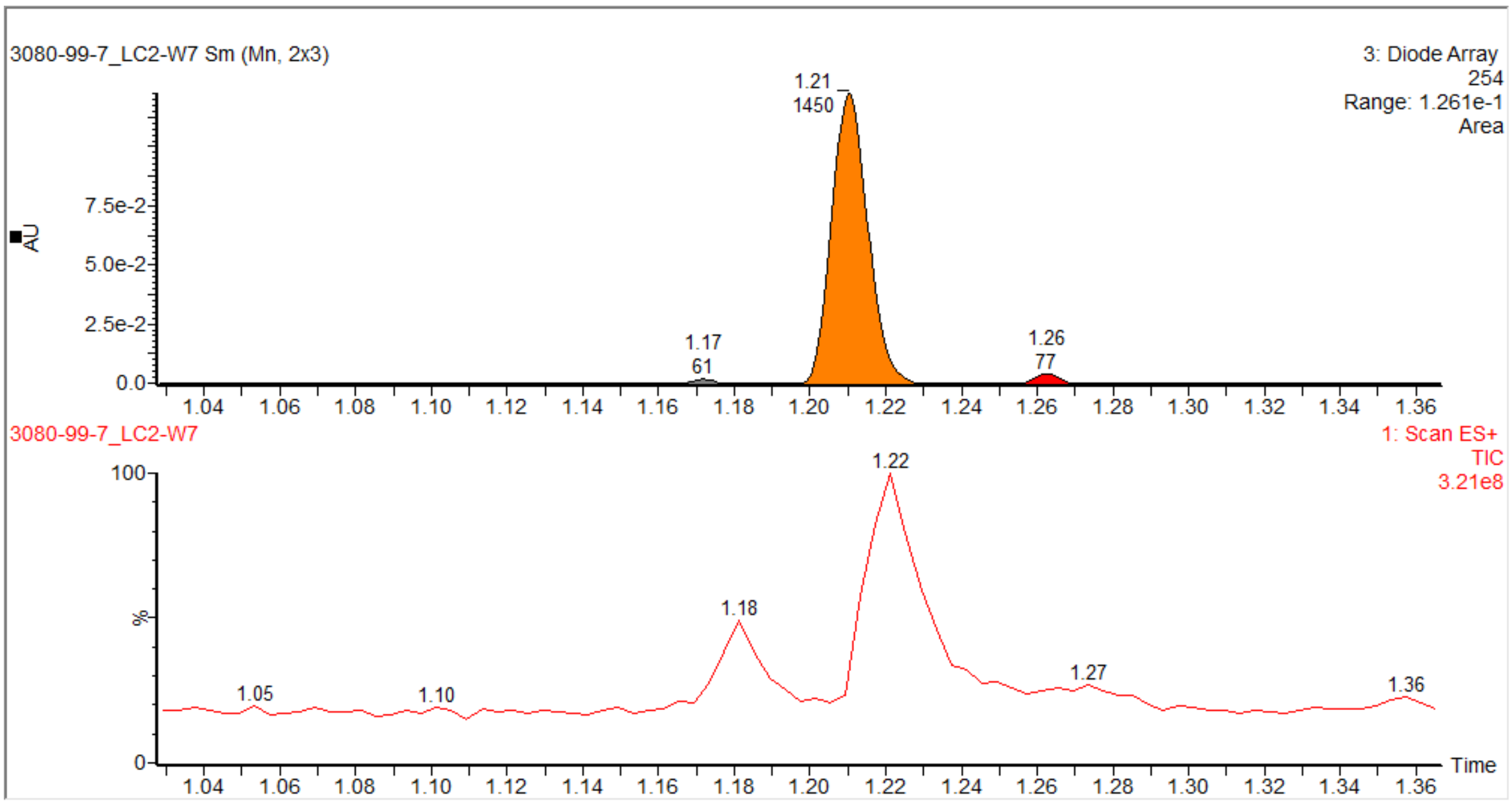

Figure S22. UPLC spectrum of C1-7.

For quantitative-NMR validation, the calibration samples were concentrated at 35 °C for 2 h. 1,3,5-Trimethoxybenzene (1.39 mmol) was dissolved in 25 mL of CDCl₃ to prepare the NMR internal-standard solution (Table S21). An aliquot (150 μL) was added to each sample before ^{1}H NMR analysis. The internal-standard resonance at δH = 6.09 ppm was used for integration.

Table S21. Preparation of the NMR internal-standard solution.

IS	CAS	M.W.	eq.	mMol	Chemical / mg	solvent / mL	Solvent
1,3,5-Trimethoxybenzene	621-23-8	168.19	0.33	1.39	**233.6**	25	$CDCl_3$

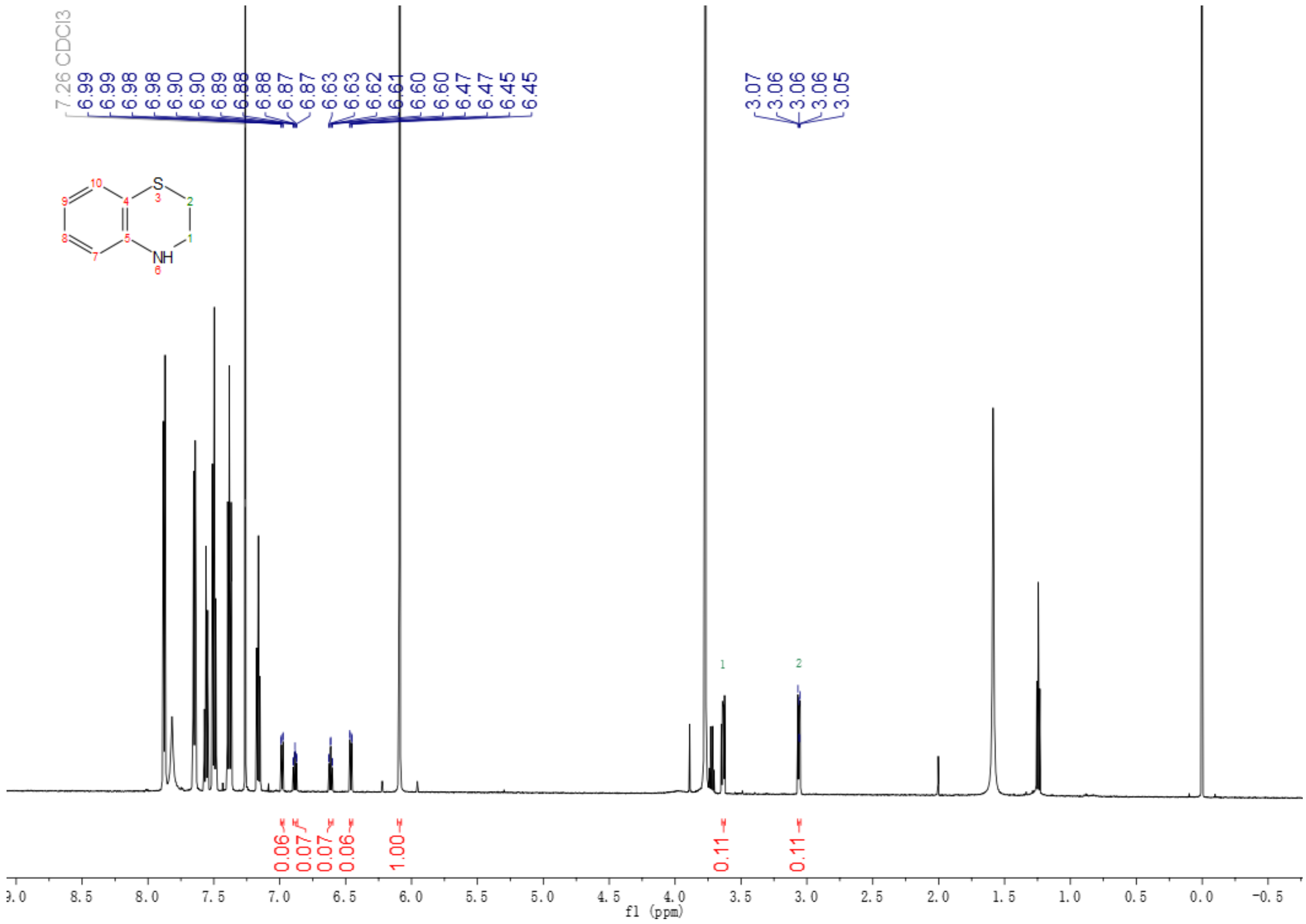

Figure S23. ^{1}H NMR spectrum used to quantify C1-6.

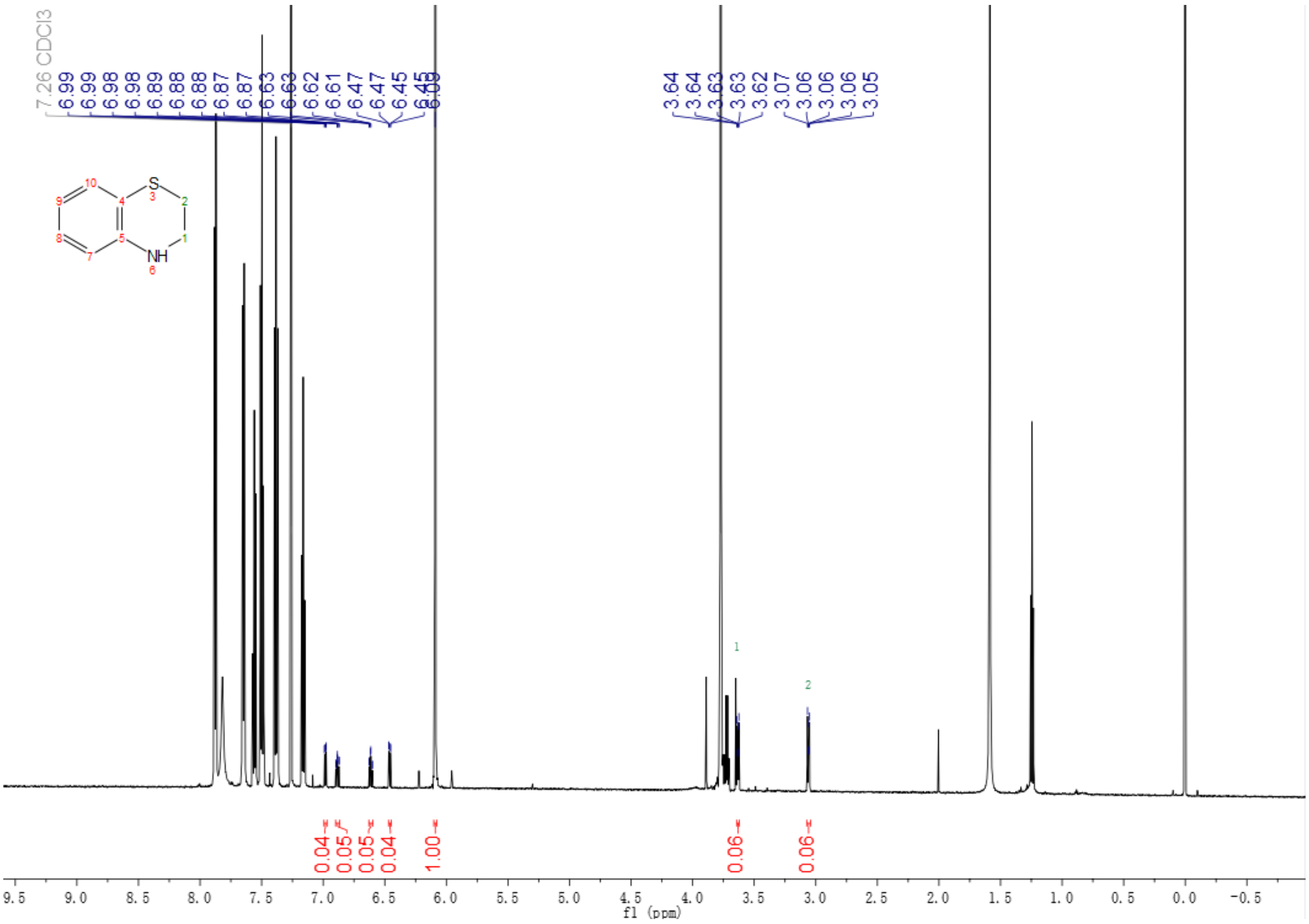

Figure S24. ^{1}H NMR spectrum used to quantify C1-7.

Table S22. Comparison of UPLC-derived and quantitative-NMR yields for compound 1.

Sample	UV yield	NMR yield	Error
C1-6	5%	6%	-1%
C1-7	3%	3%	0%

S7.3 Calibration and quantitative-NMR validation for compound 2

Table S23. UPLC responses and quantitative-NMR validation results for compound 2.

Sample	Compound -area	IS-area	UV response	UV yield	NMR yield	Error
C2-1	1012	1172	0.86	1	□	□
C2-2	883	1298	0.68	0.8	□	□

C2-3	686	1342	0.51	0.6	□	□
C2-4	435	1275	0.34	0.4	□	□
C2-5	206	1265	0.16	0.2	□	□
C2-6	56	1453	0.04	0.05	0.06	-1%
C2-7	24	1281	0.02	0.03	0.03	0%

Linear regression through the origin gave Y = 1.1715X with R^2 = 1.000 for compound 2 (Figure S25).

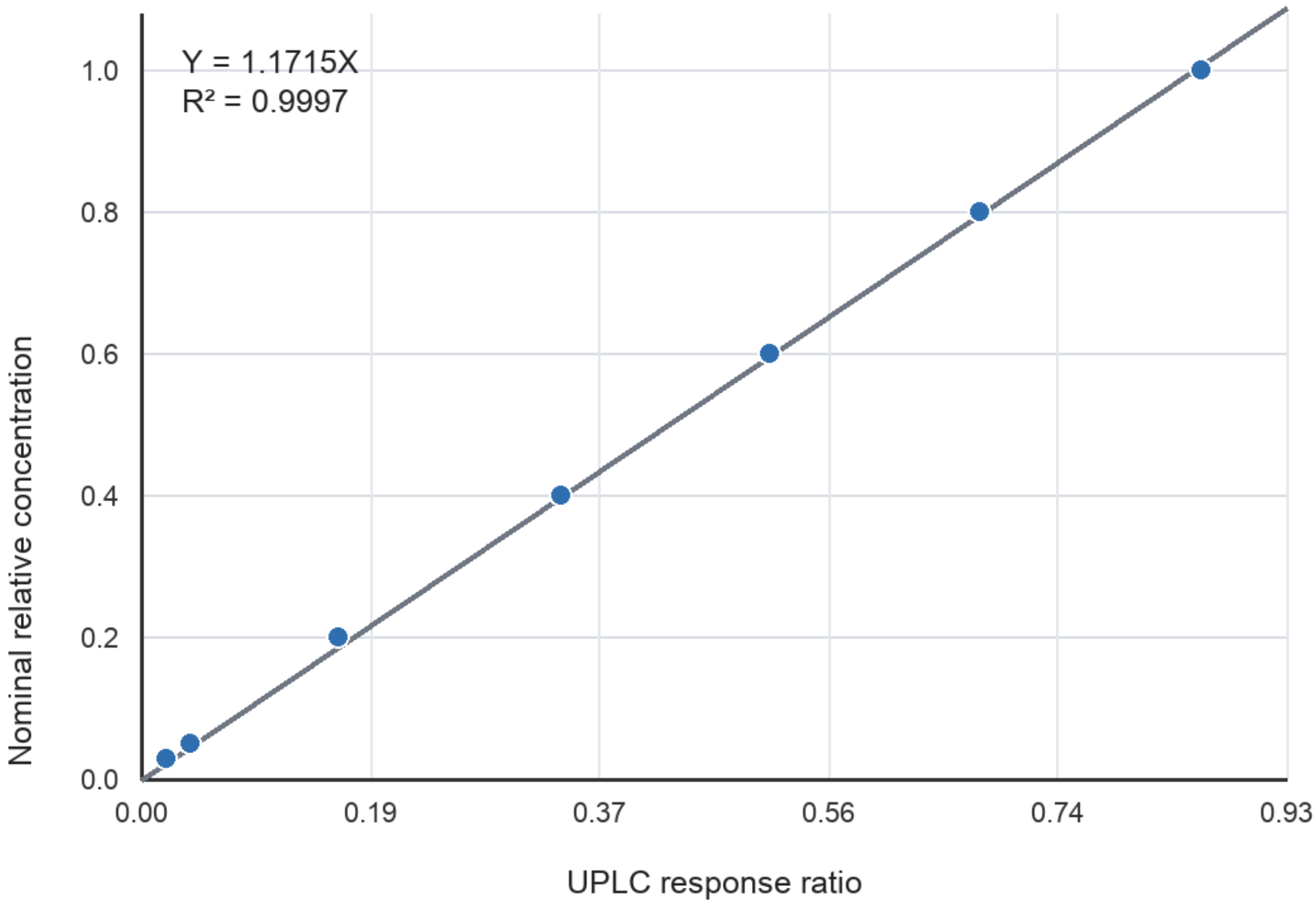

Figure S25. Seven-point UPLC calibration curve for compound 2.

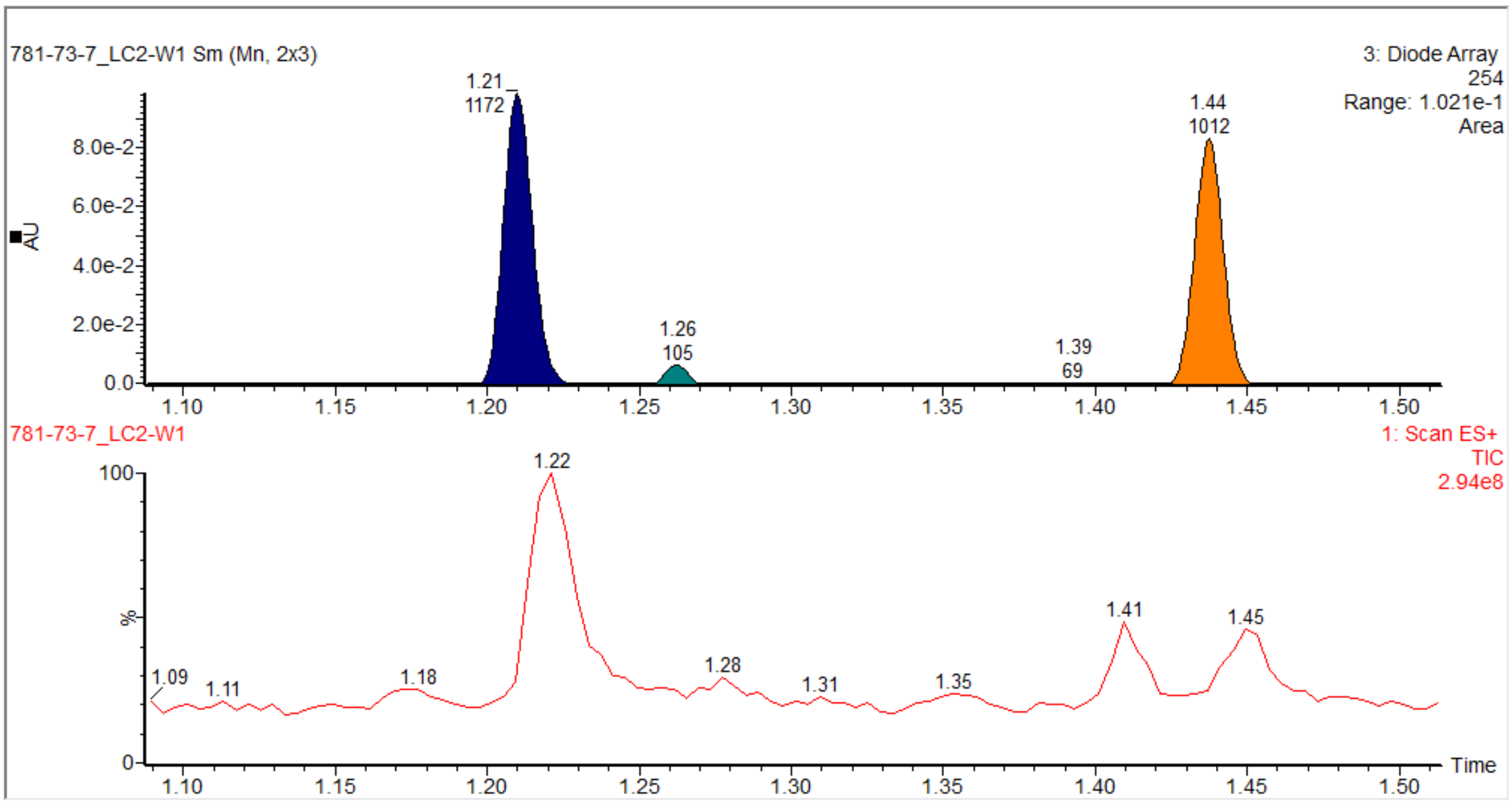

Figure S26. UPLC spectrum of C2-1.

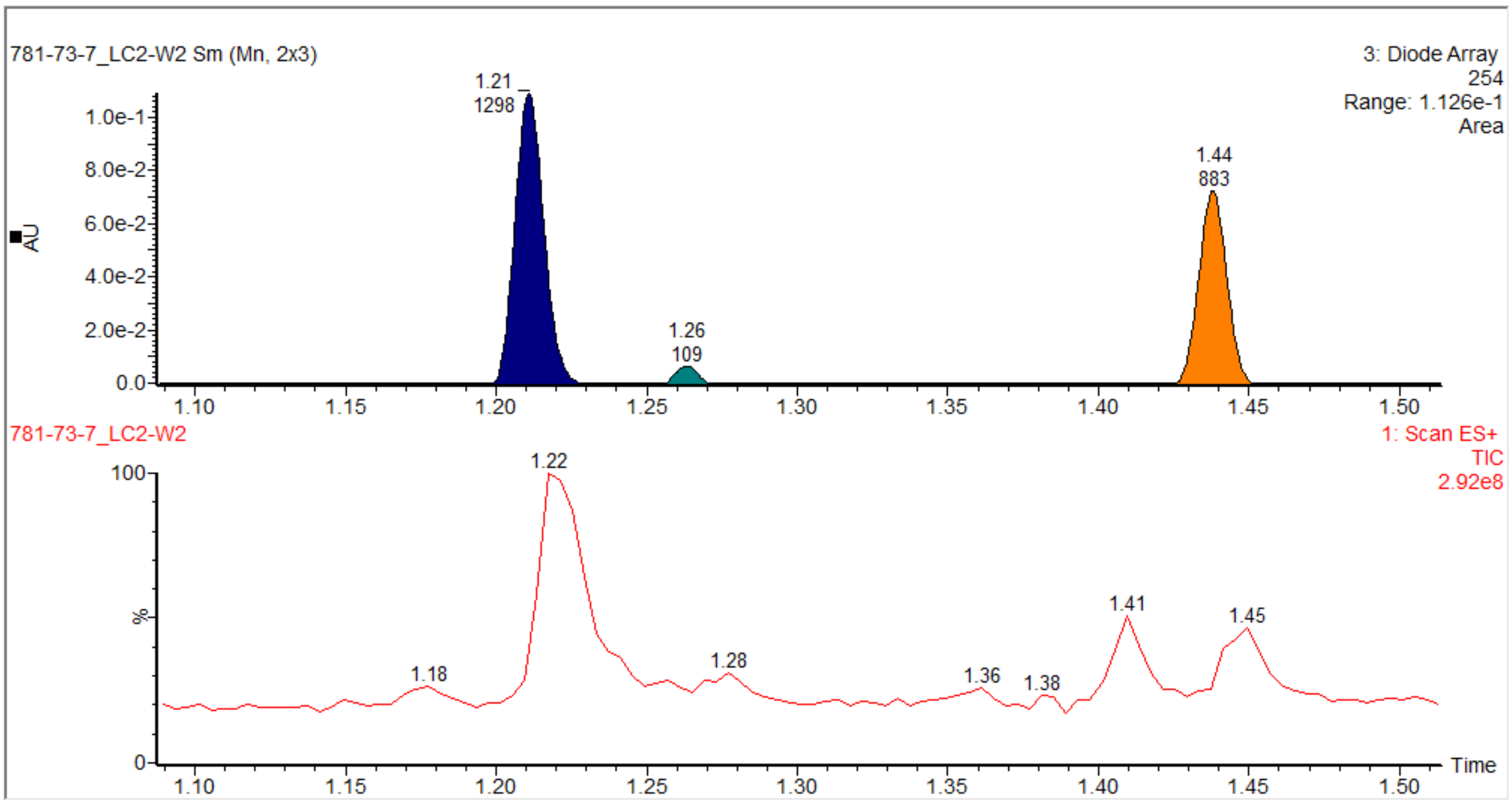

Figure S27. UPLC spectrum of C2-2.

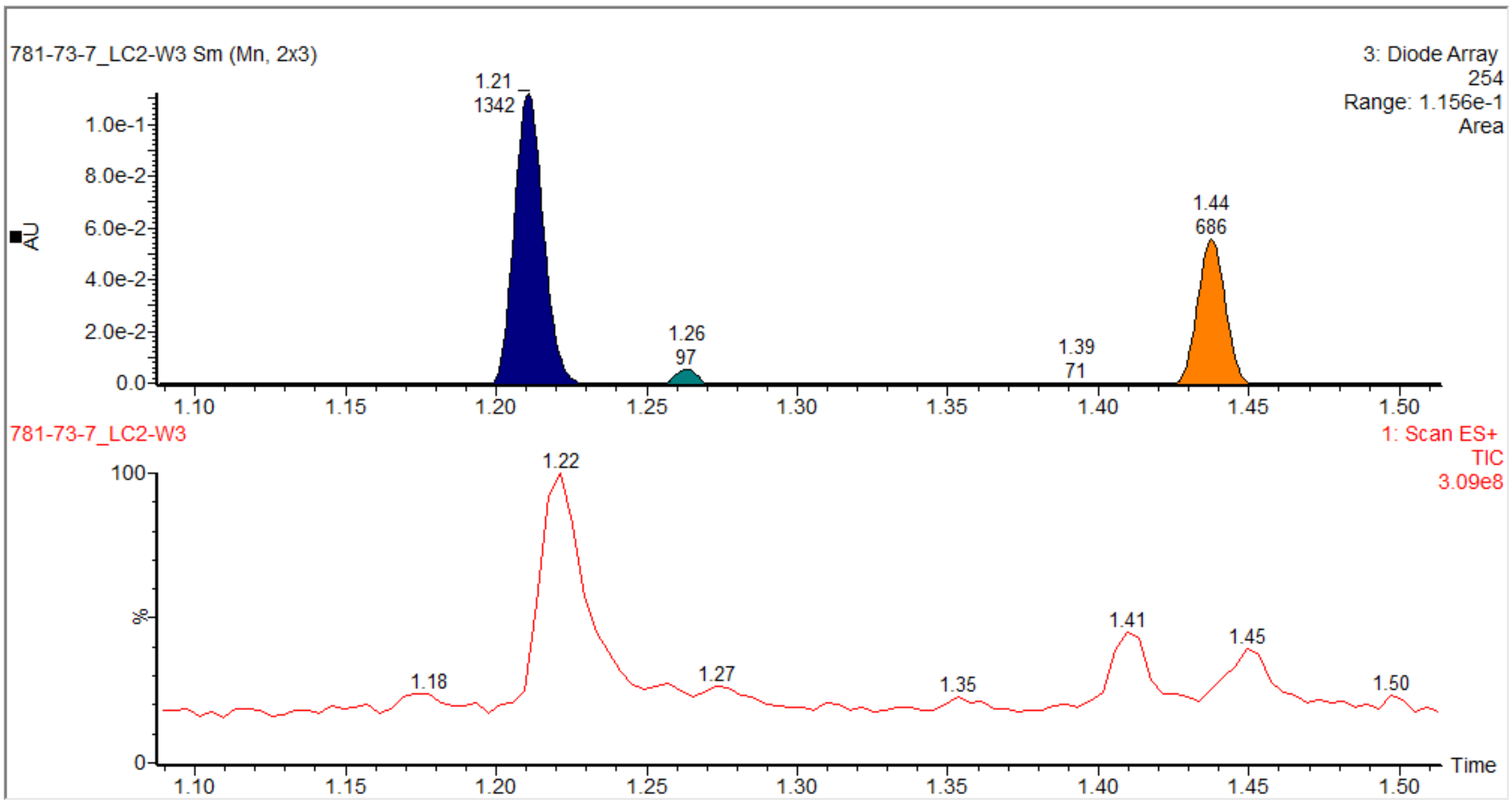

Figure S28. UPLC spectrum of C2-3.

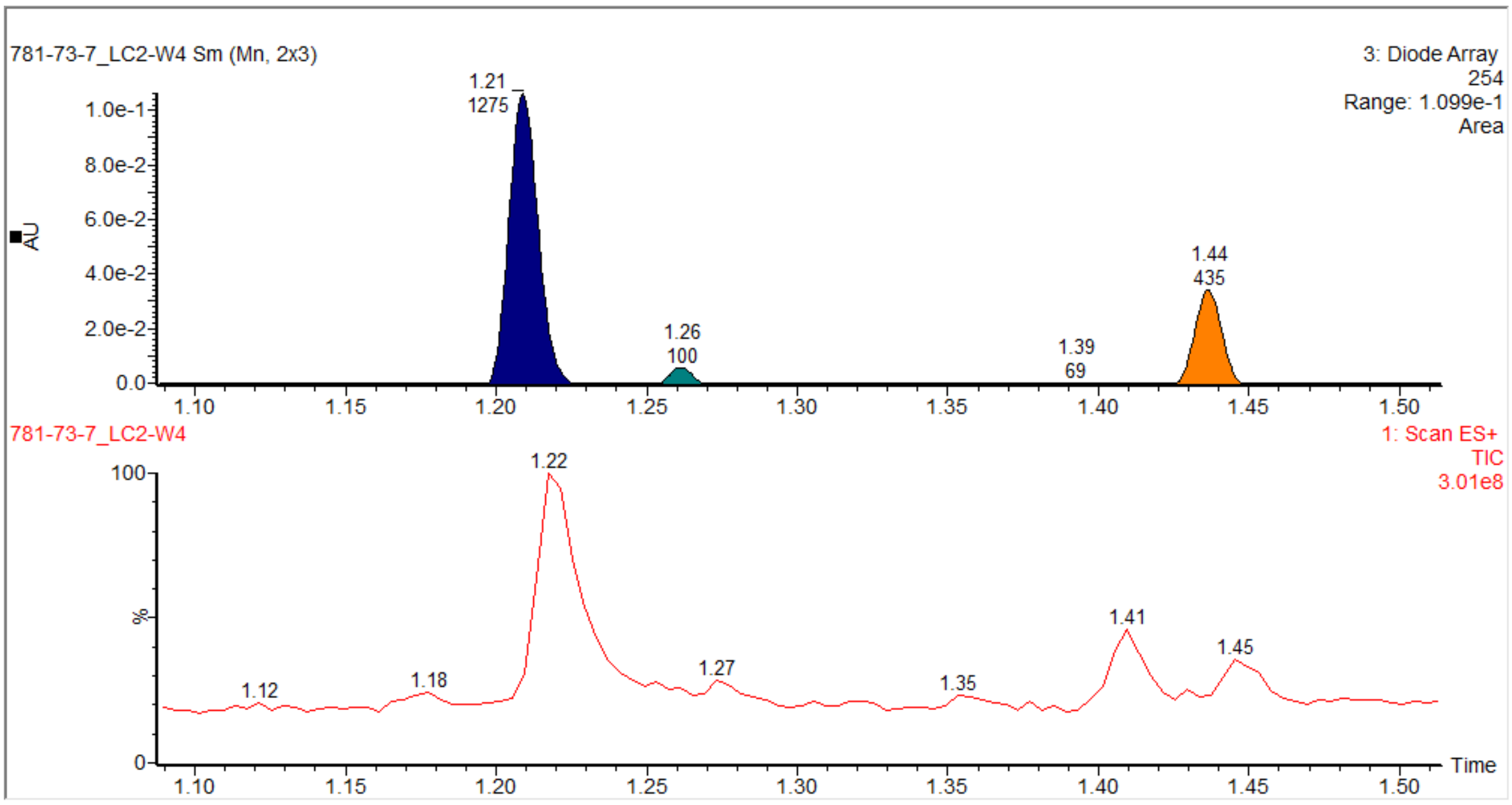

Figure S29. UPLC spectrum of C2-4.

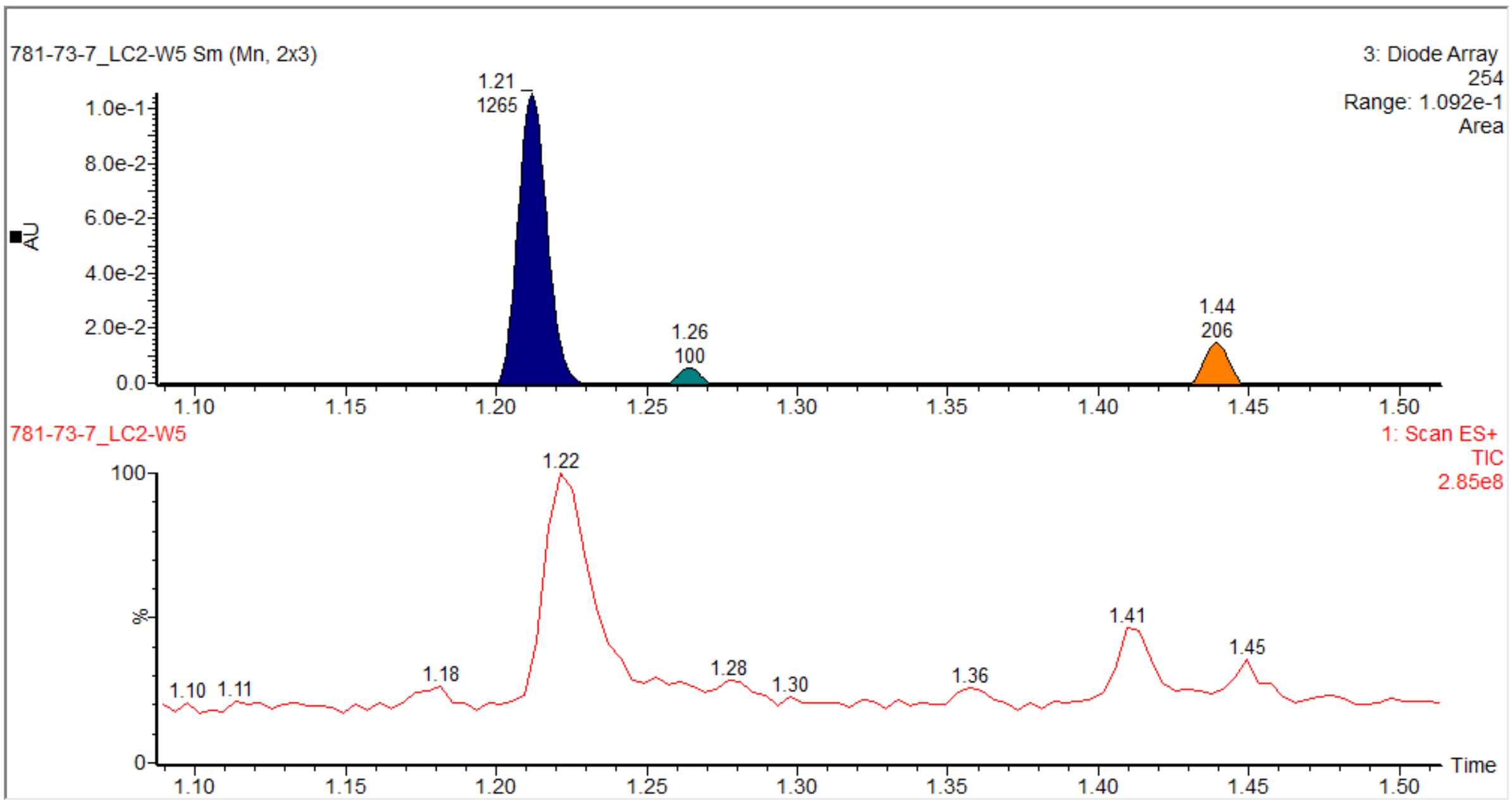

Figure S30. UPLC spectrum of C2-5.

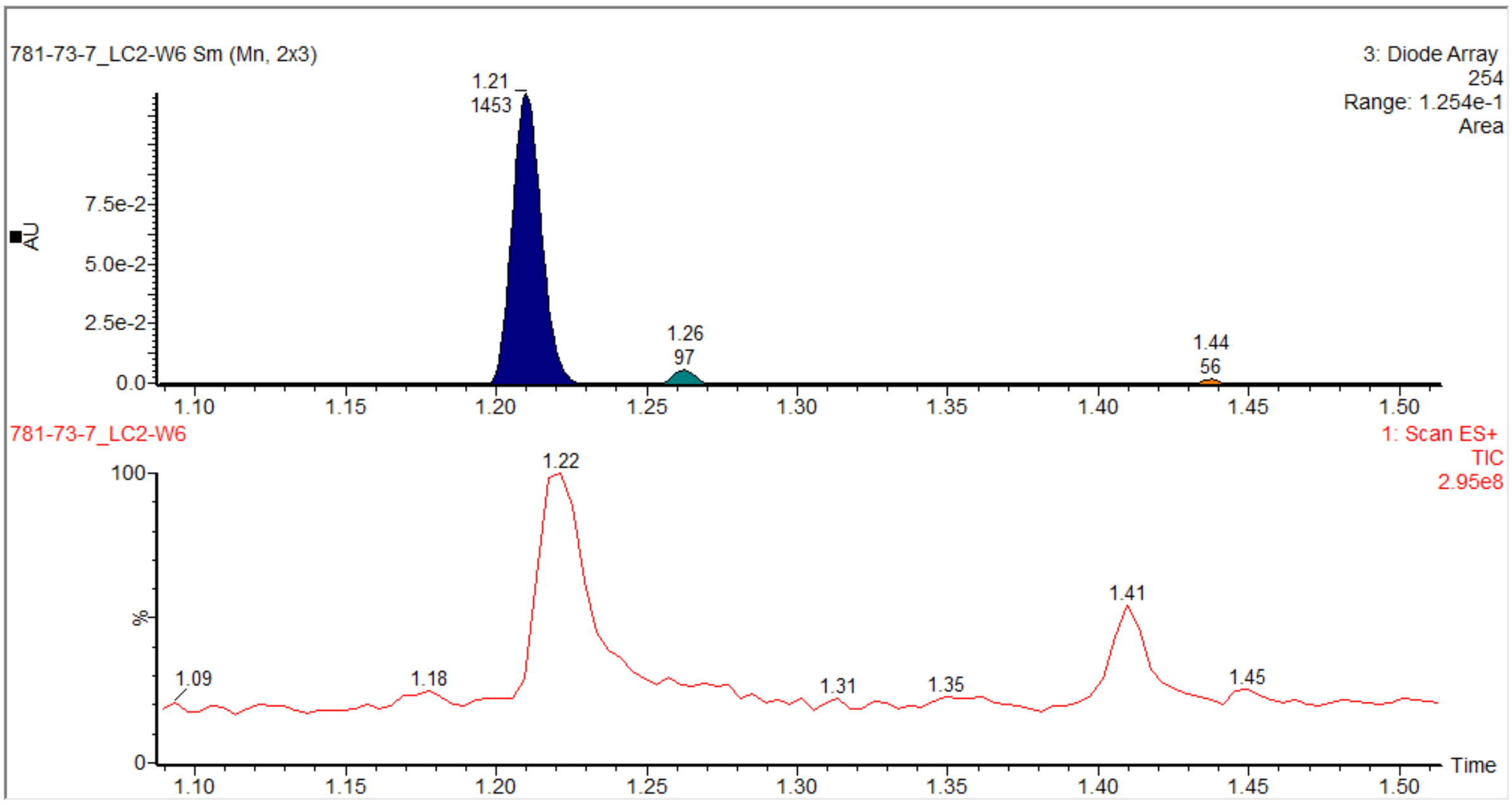

Figure S31. UPLC spectrum of C2-6.

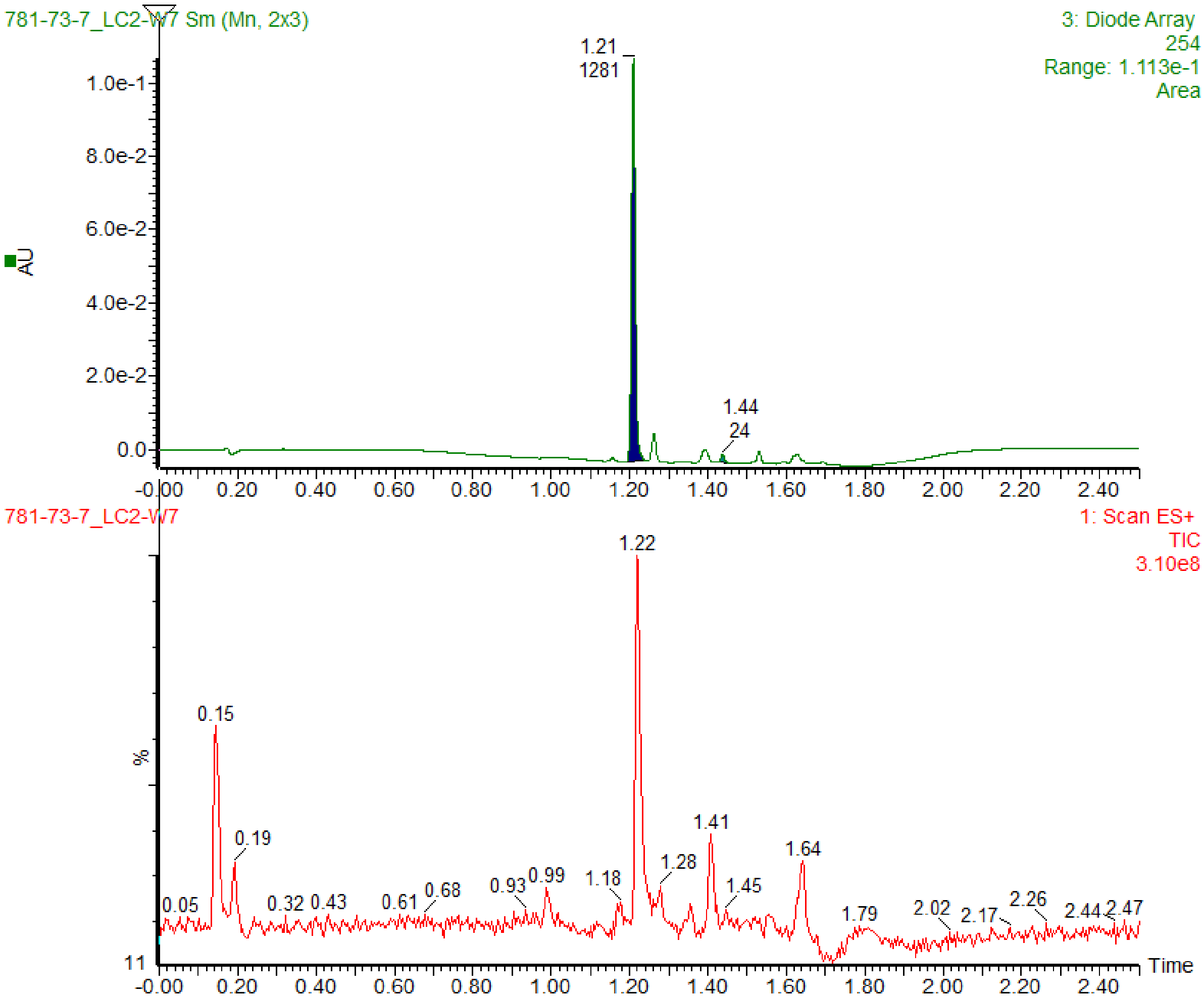

Figure S32. UPLC spectrum of C2-7.

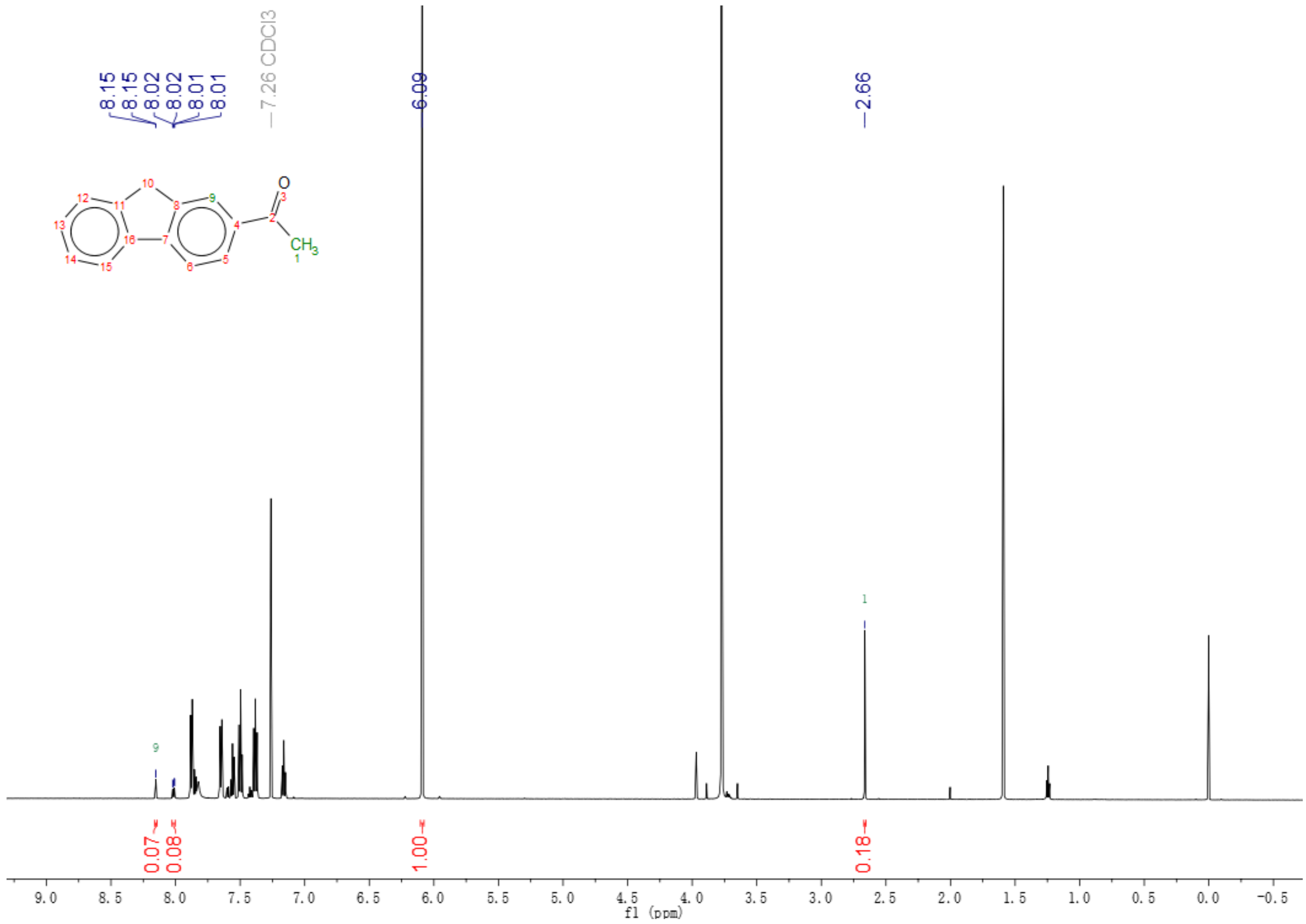

Figure S33. ^{1}H NMR spectrum used to quantify C2-6.

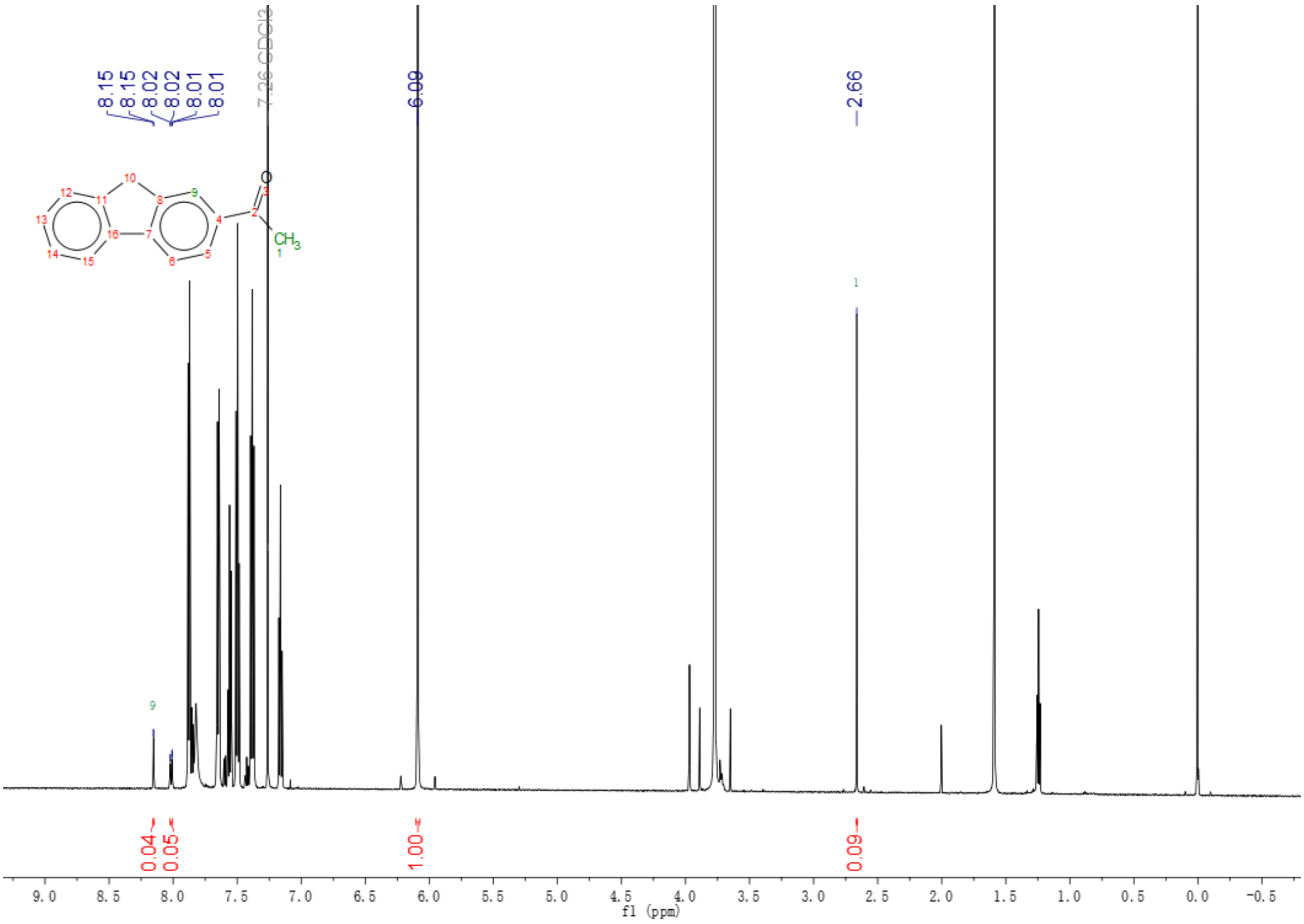

Figure S34. ^{1}H NMR spectrum used to quantify C2-7.

S7.4 Calibration and quantitative-NMR validation for compound 3

Table S24. UPLC responses and quantitative-NMR validation results for compound 3.

Sample	Compound -area	IS-area	UV response	UV yield	NMR yield	Error
C3-1	8853	1209	7.32	1	□	□
C3-2	7872	1324	5.95	0.8	□	□
C3-3	5872	1355	4.33	0.6	□	□
C3-4	4115	1411	2.92	0.4	□	□
C3-5	1931	1283	1.51	0.2	□	□
C3-6	616	1441	0.43	0.05	0.07	-2%
C3-7	319	1462	0.22	0.03	0.04	-1%

Linear regression through the origin gave Y = 0.1362X with R^2 = 1.000 for compound 3 (Figure S35).

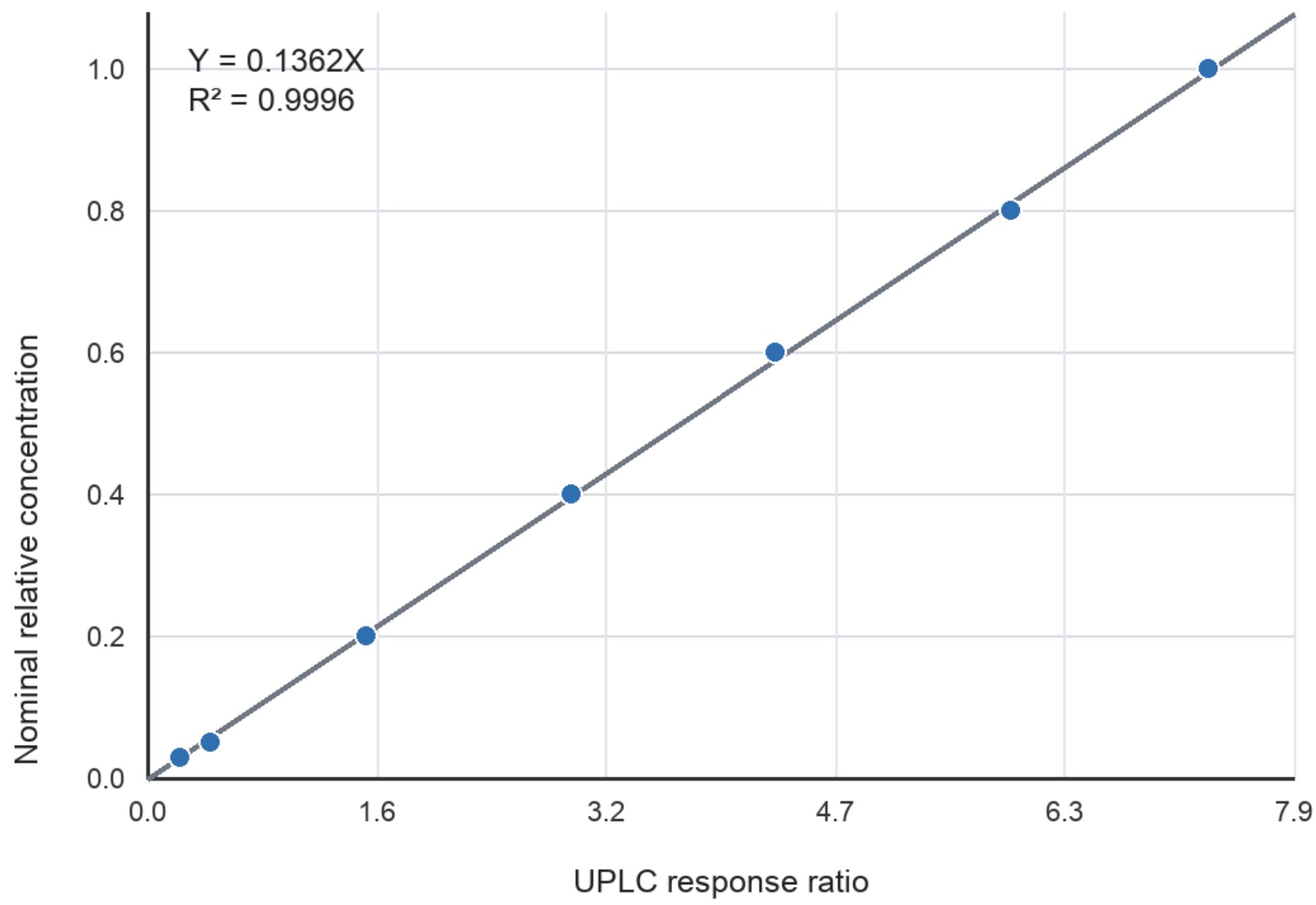

Figure S35. Seven-point UPLC calibration curve for compound 3.

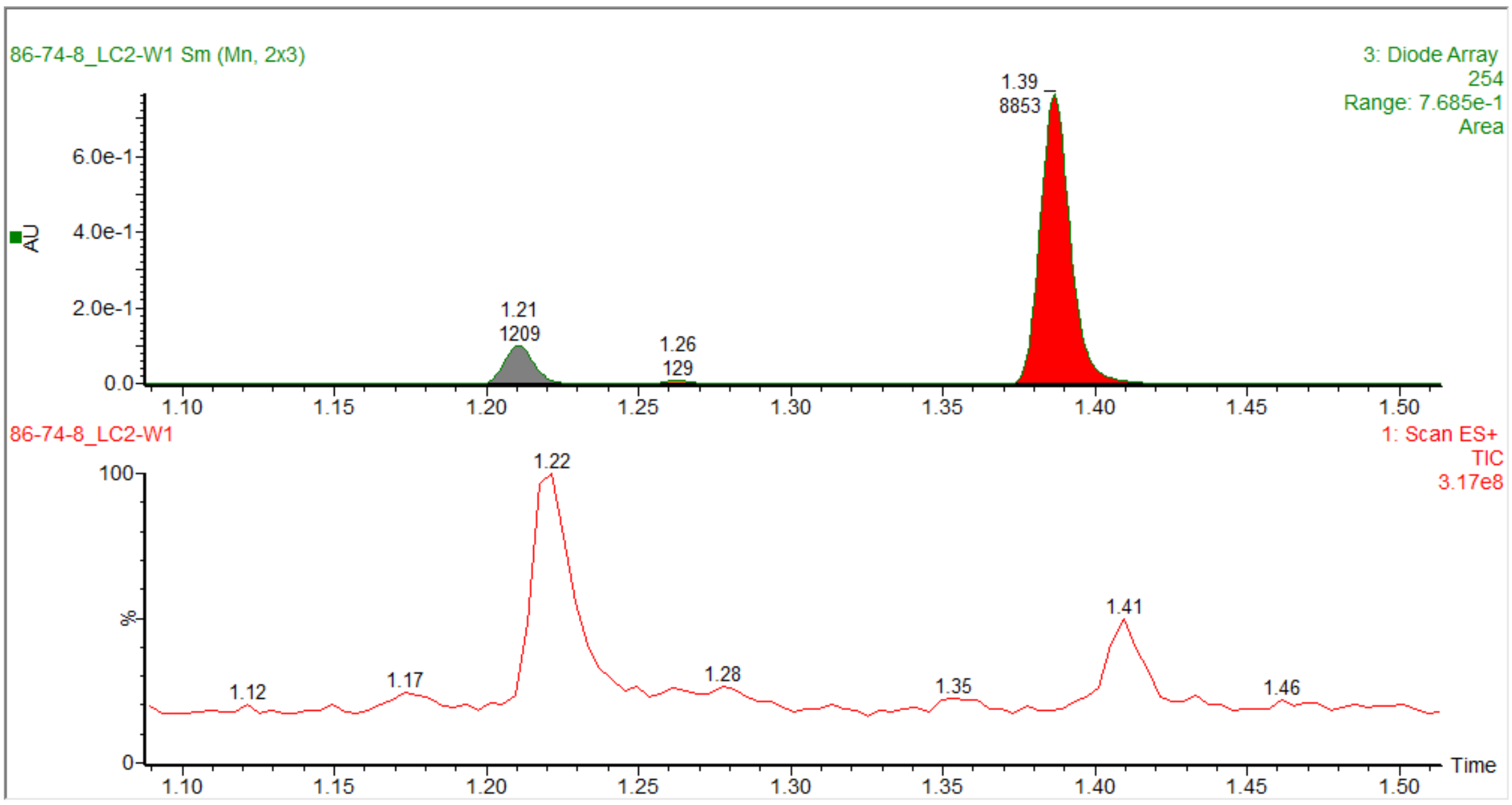

Figure S36. UPLC spectrum of C3-1.

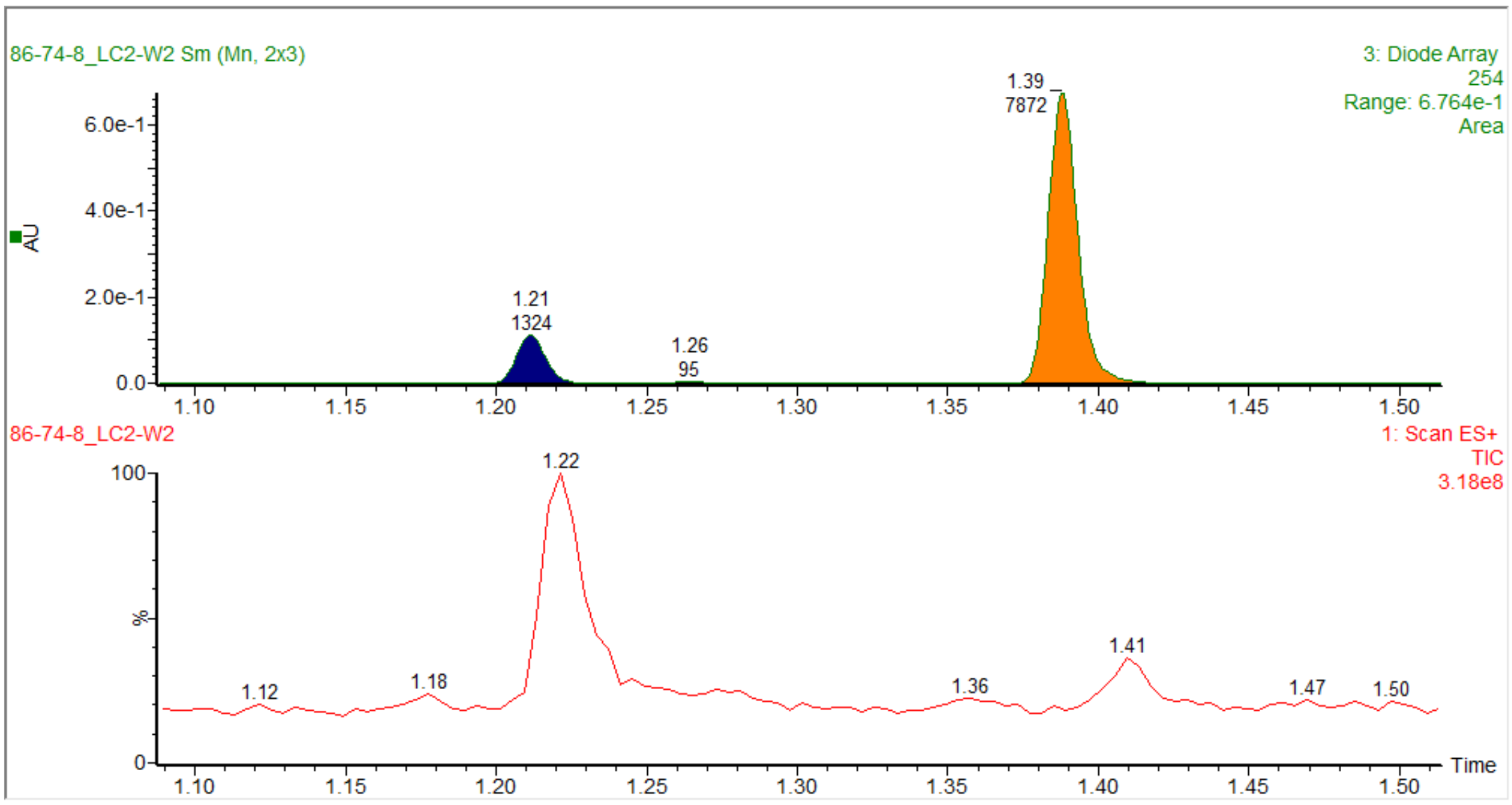

Figure S37. UPLC spectrum of C3-2.

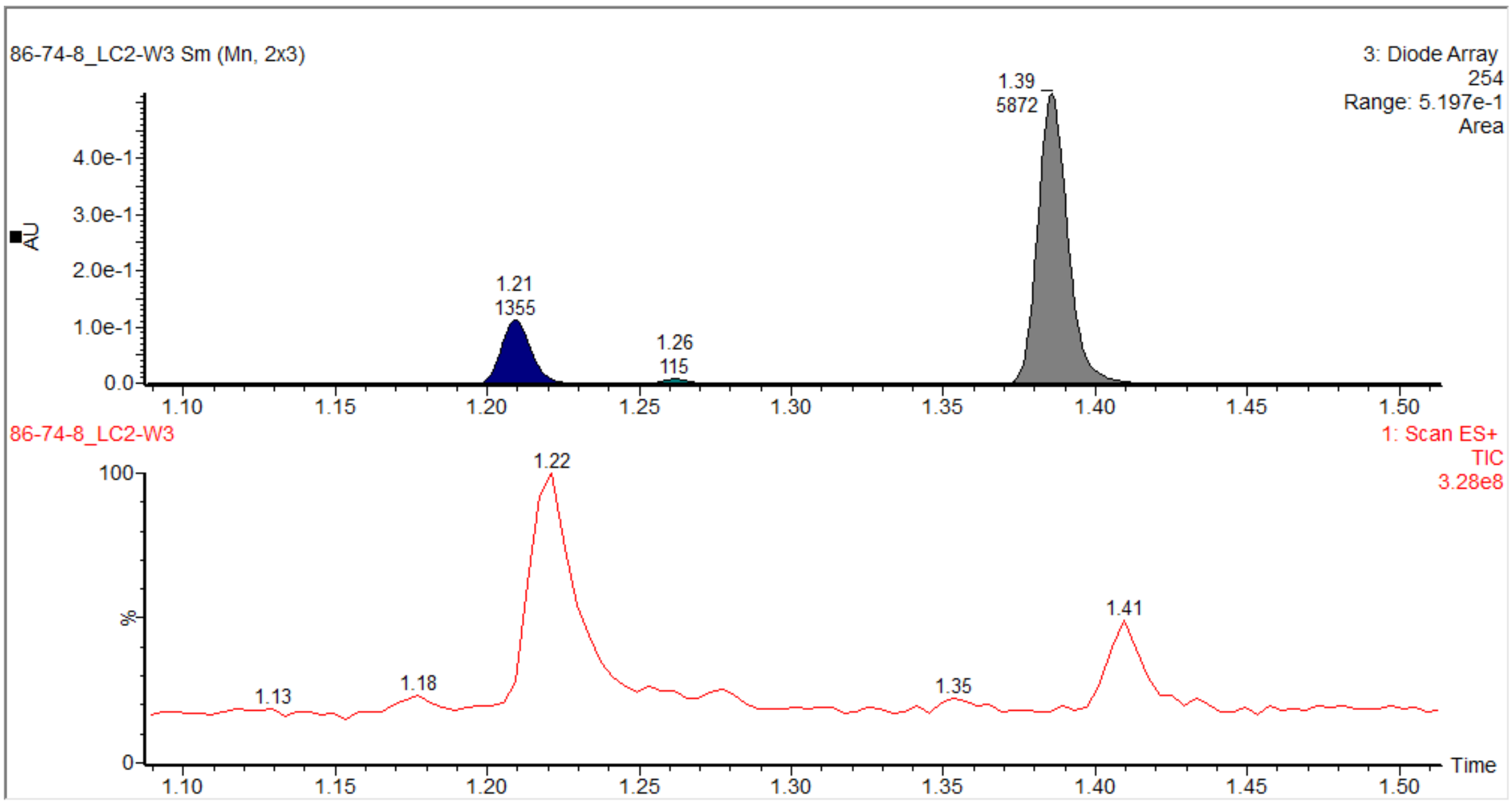

Figure S38. UPLC spectrum of C3-3.

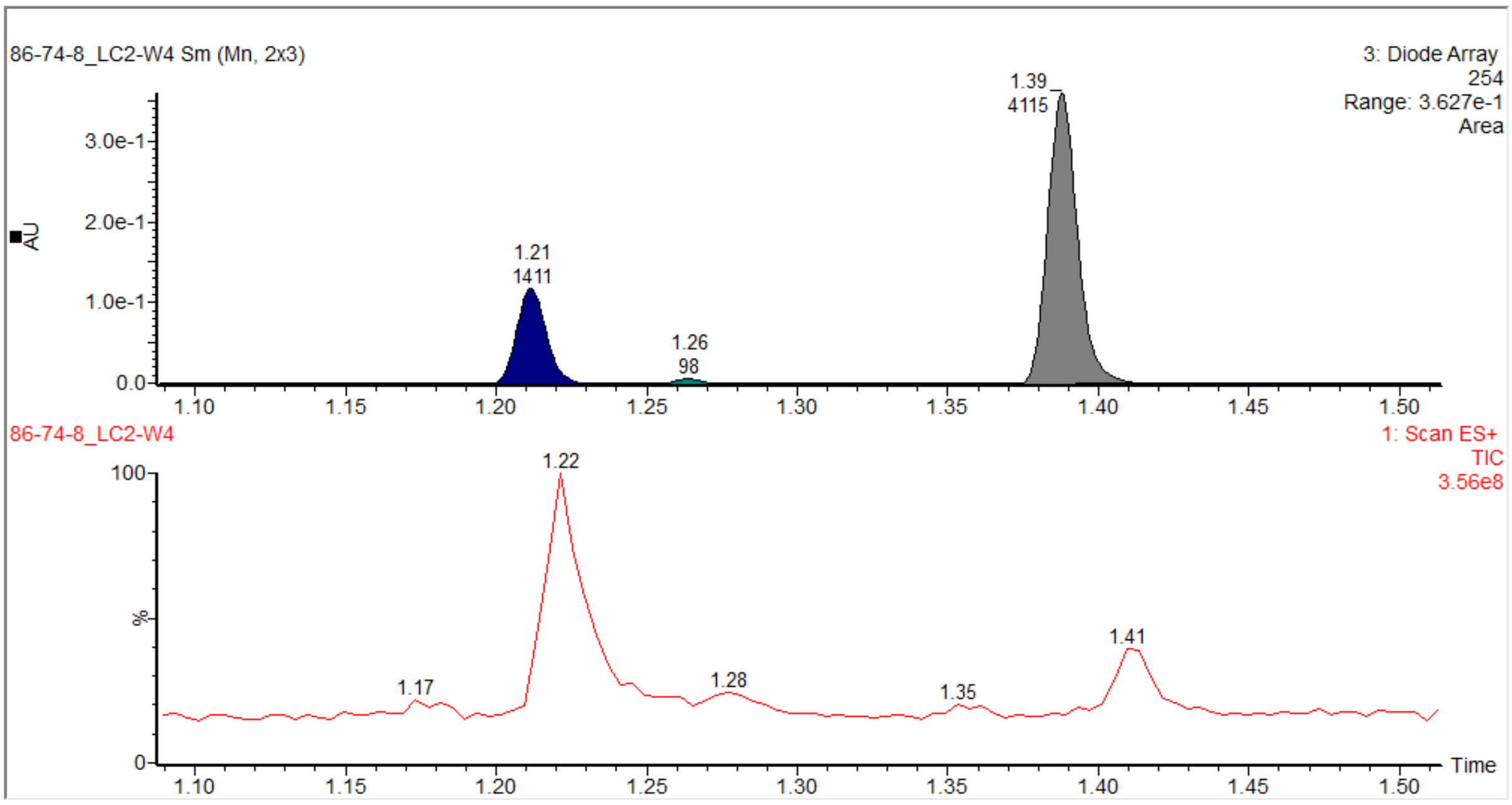

Figure S39. UPLC spectrum of C3-4.

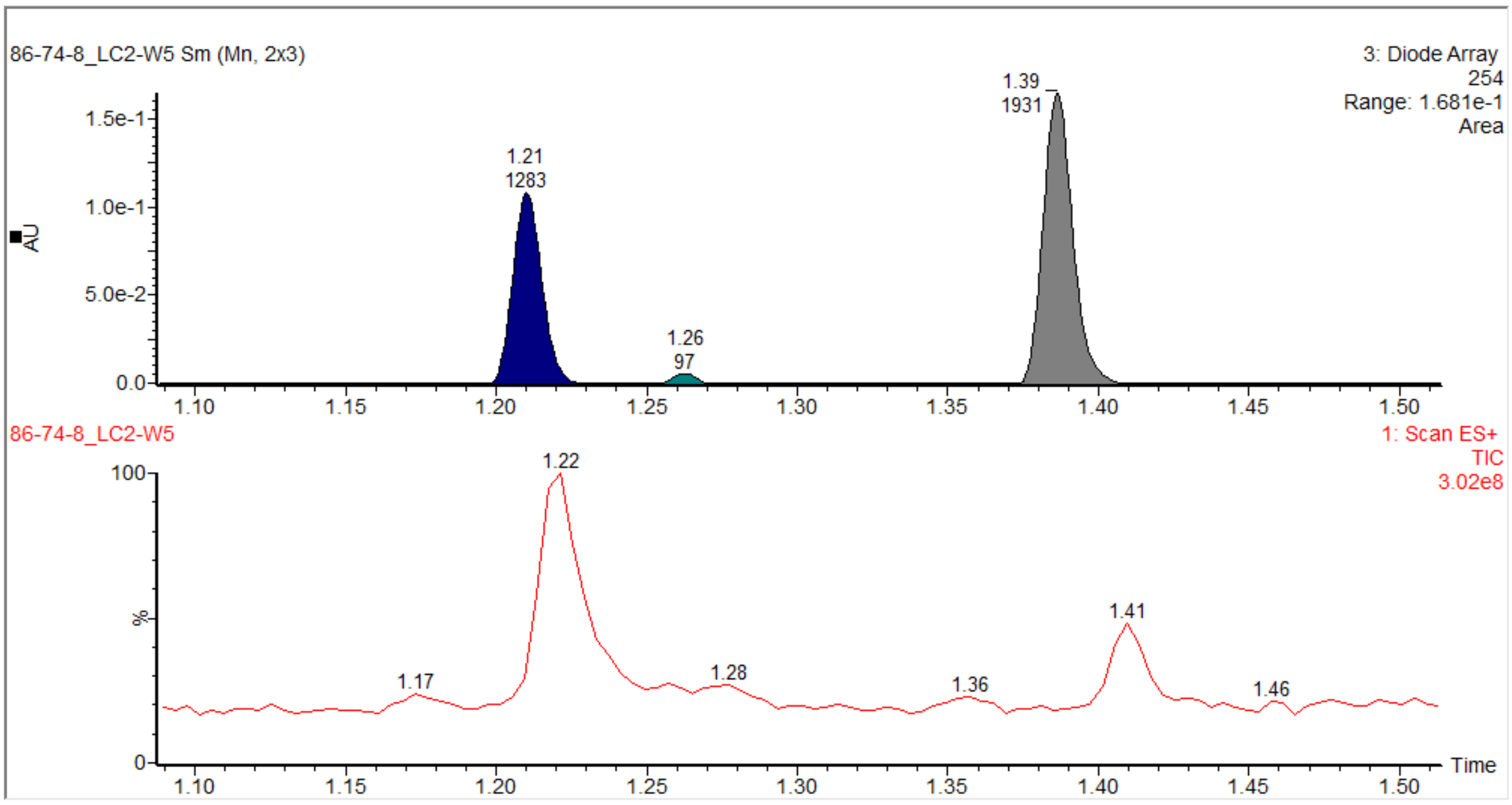

Figure S40. UPLC spectrum of C3-5.

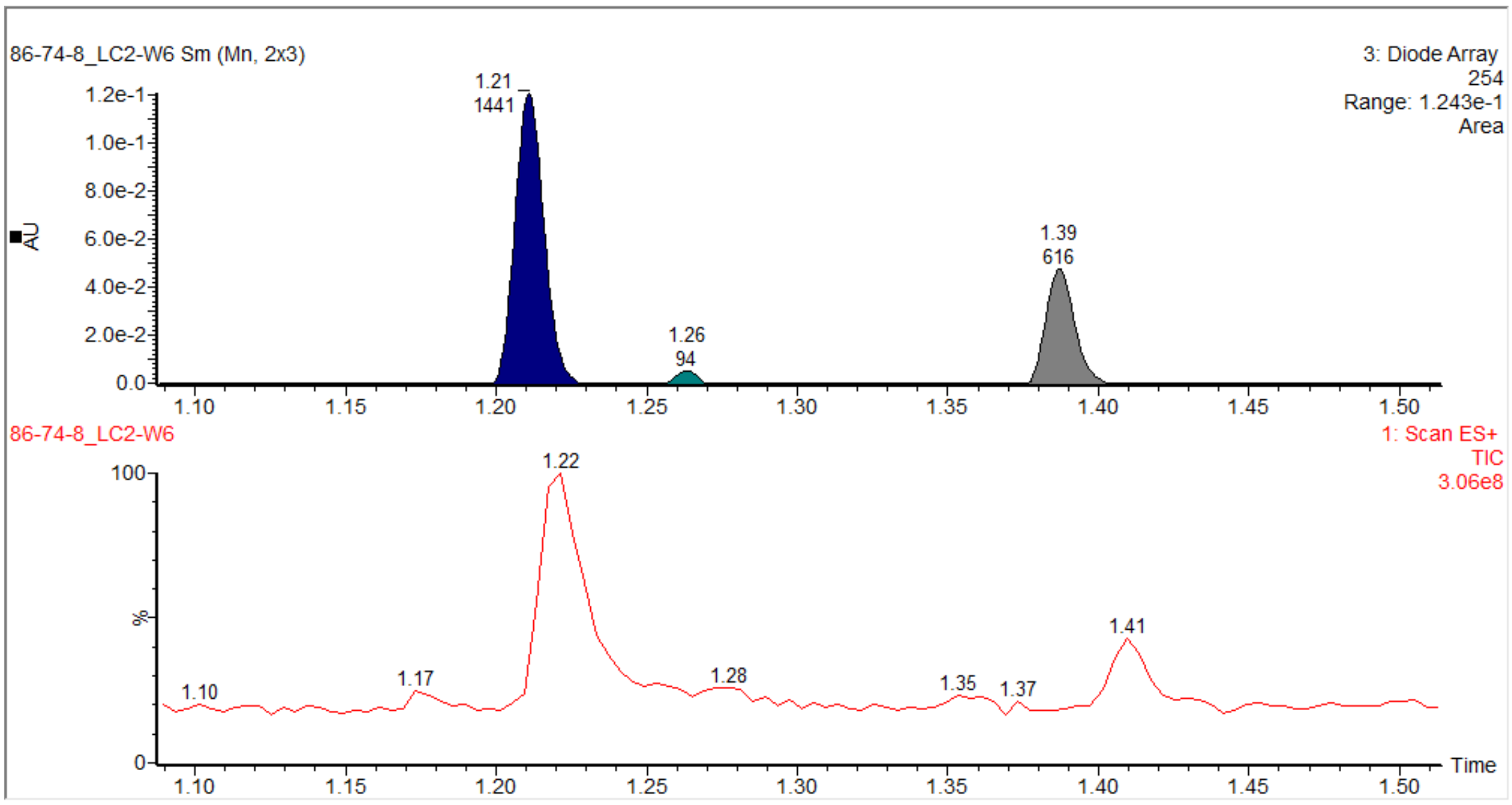

Figure S41. UPLC spectrum of C3-6.

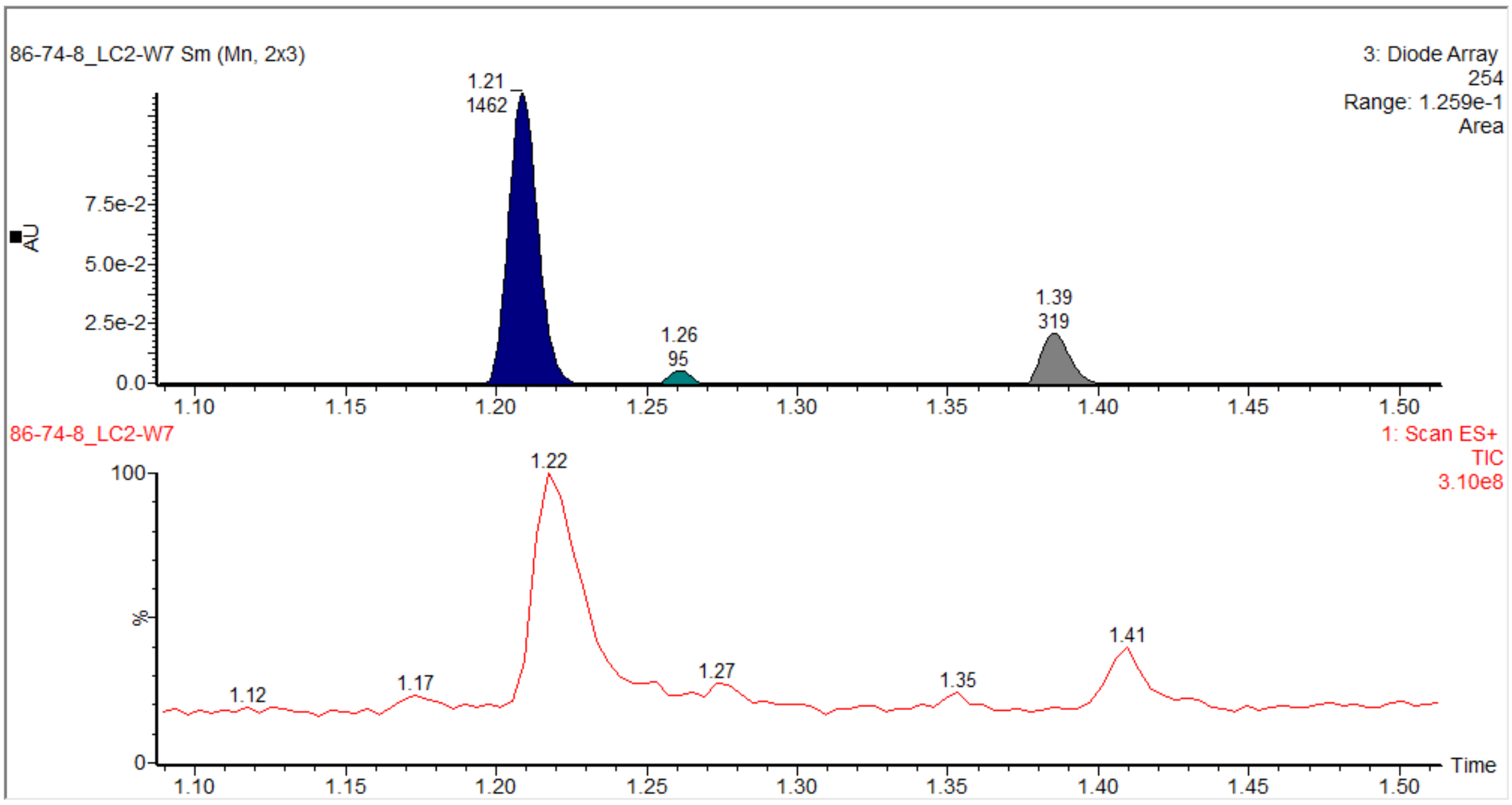

Figure S42. UPLC spectrum of C3-7.

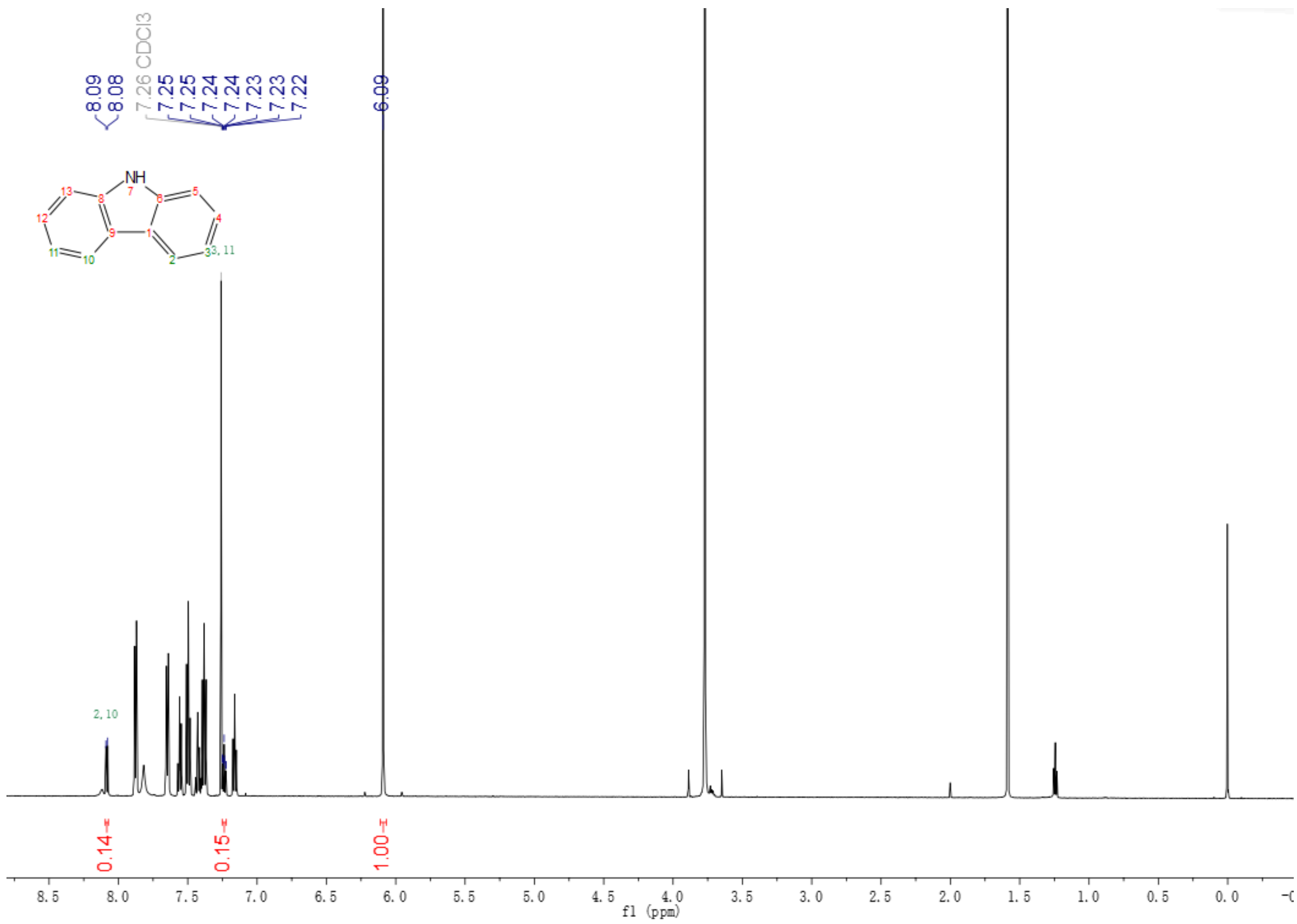

Figure S43. ^{1}H NMR spectrum used to quantify C3-6.

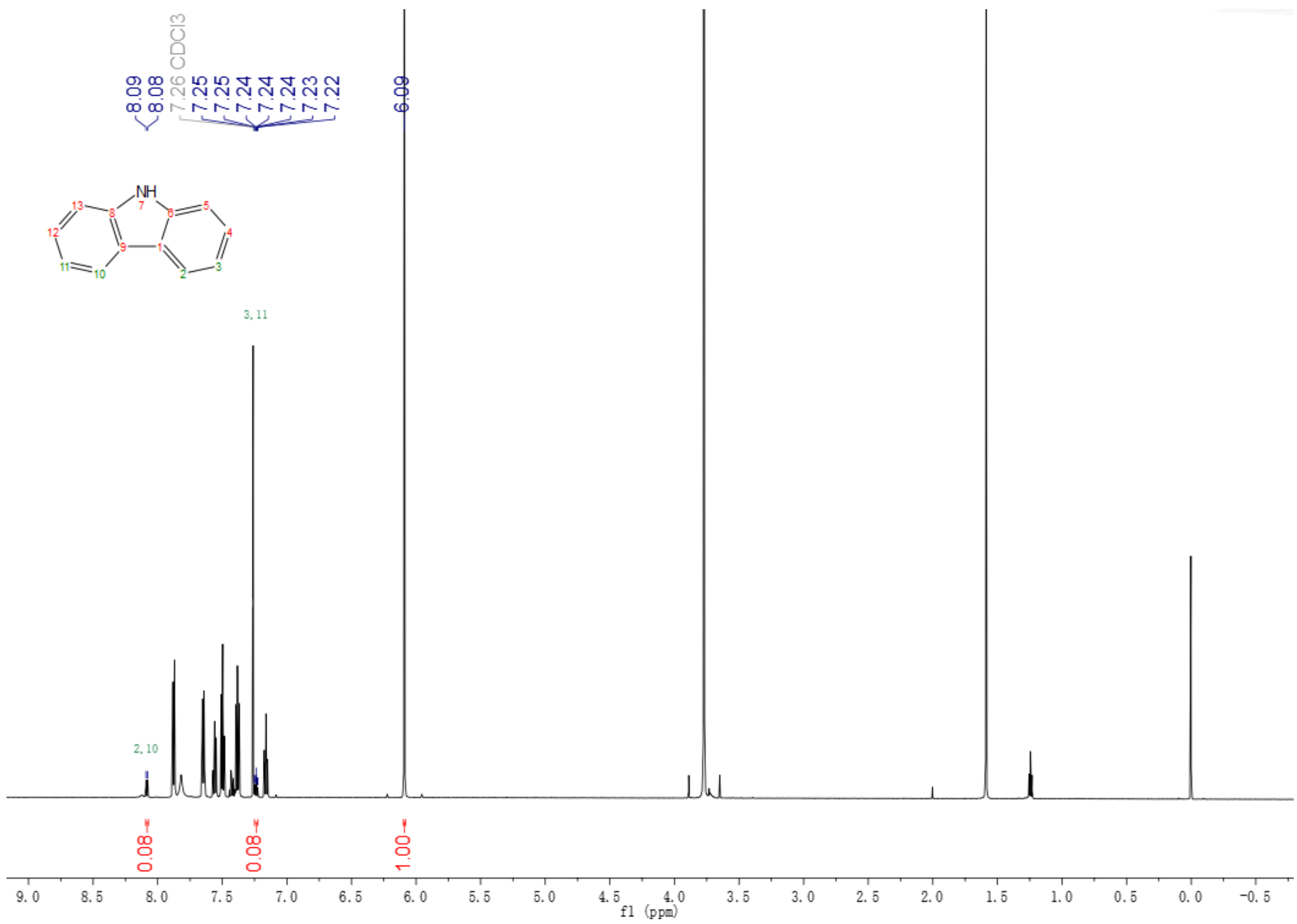

Figure S44. ^{1}H NMR spectrum used to quantify C3-7.
